# Ciclopirox drives growth arrest and autophagic cell death through STAT3 in gastric cancer cells

**DOI:** 10.1038/s41419-022-05456-7

**Published:** 2022-11-28

**Authors:** Lingyan Chen, Dejian Chen, Jiwei Li, Lipeng He, Ting Chen, Dandan Song, Shuang Shan, Jiaxin Wang, Xiaoang Lu, Bin Lu

**Affiliations:** 1 Protein Quality Control and Diseases Laboratory, Zhejiang Provincial Key Laboratory of Medical Genetics, Key Laboratory of Laboratory Medicine, Ministry of Education, School of Laboratory Medicine and Life Sciences, Wenzhou Medical University, Wenzhou, Zhejiang 325035 China; 2 The Affiliated Nanhua Hospital and Department of Biochemistry and Molecular Biology, School of Basic Medical Sciences, Hengyang Medical School, University of South China, Hengyang, Hunan 421001 China

**Keywords:** Drug development, Chemotherapy

## Abstract

Ciclopirox (CPX), an antifungal drug, has recently been identified as a promising agent for cancer treatment. However, the effects and underlying mechanism of CPX as an antitumor agent of gastric cancer (GC) remain largely unknown. Here, we found that CPX dramatically suppresses GC xenograft growth in vitro via inhibiting proliferation and stimulating autophagic cell death rather than apoptosis. Moreover, CPX (20 mg/kg, intraperitoneally) substantially inhibits GC xenograft tumor growth in vivo. Mechanistically, CPX promotes growth arrest and autophagic cell death through suppressing the phosphorylation of signal transducers and activators of transcription 3 (STAT3) at tyrosine 705 (Tyr705) and serine 727 (Ser727) sites, respectively. Additionally, CPX induces STAT3 ubiquitination, which subsequently leads to a decrease in the p-STAT3 (Ser727) level. On the other hand, CPX represses the p-STAT3 (Tyr705) level via p-Src (Tyr416) inhibition. Collectively, our findings unmask a novel mechanism by which CPX regulates growth and autophagic cell death in GC cells via regulating the phosphorylation of STAT3 both at Tyr705 and Ser727 residues, and suggest that CPX may be a potential treatment for GC.

## Introduction

Gastric cancer (GC), a malignant tumor that originates from gastric mucosal, is the third leading cause of cancer-related mortality worldwide [1]. Surgery and/or chemotherapy are the main therapeutic strategies for GC, however, the recurrence and metastasis ratio of GC continues to mount [2]. Drugs, such as trastuzumab and apatinib, are only suitable for patients with advanced or refractory GC, but the cure rate is still low [3, 4]. Therefore, it is urgent to find novel treatments for GC.

Increasing evidence shows that antifungal drugs have potential anticancer activity in various cancers, such as GC and non-small cell lung cancer [5, 6]. Our previous studies showed that CPX induces colorectal cancer (CRC) and non-small cell lung cancer (NSCLC) cell death and CPX in synergy with bortezomib retards glioblastoma multiforme (GBM) growth [7–9]. CPX, a broad-spectrum fungicide used against dermatophytes, yeast, and filamentous fungi for over two decades, had significant antitumor activity in several cancers [10, 11]. A noteworthy clinical study showed that CPX shows satisfactory safety, tolerability, and pharmacodynamic activity in patients with advanced hematologic malignancies, suggesting that CPX may be a potential antitumor agent [12]. However, the activity and molecular mechanisms of CPX in GC have not yet been defined.

Activation of STAT3 induces growth, inflammatory infiltration, and vascularization in mouse models of gastric tumorigenesis, and mediates poor prognosis in GC [13, 14]. Additionally, the IL-6-STAT3-NEK9 pathway regulates metastasis of GC cells via targeting the phosphorylation of Rho/Rac guanine nucleotide exchange factor 2 [15]. Moreover, the STAT3/epigenetic kinase mitogen- and stress-activated protein kinase 1 signaling significantly promotes GC cell proliferation and tumor growth of xenografts [16]. Importantly, *Helicobacter pylori* infection, one of the risk factors of GC, can activate STAT3 in human and mouse gastric epithelial cells to accelerate the progression of GC [17]. Therefore, numerous studies support STAT3 as a promising therapeutic target for human cancer treatment, especially in GC [18, 19].

In eukaryotic cells, STAT3 is regulated by the Janus kinases (JAKs), tyrosine kinase, and non-receptor tyrosine kinase [20]. Phosphorylation of STAT3 at Tyr705 is critical for its homodimerization, nuclear localization, and transactivation, which can promote tumor cell proliferation via enhancing cell cycle-related protein expression [21, 22]. While phosphorylation of STAT3 at Ser727 is mediated by extracellular regulated protein kinases (ERK), inhibition of ERK effectively triggers autophagic cell death via an mTOR-dependent pathway in GBM cells [23, 24]. Therefore, phosphorylation of the Ser727 and Tyr705 residues is critical for STAT3 in promoting tumorigenesis. However, the regulatory mechanism of STAT3 in GC, especially p-STAT3 (Tyr705) and p-STAT3 (Ser727) has not been fully elucidated.

This study aims to investigate the impact of CPX on regulating GC tumor growth. Here, we identified that CPX induces cell cycle arrest at the G1/S phase in GC cells through inhibiting p-Src (Tyr416)/p-STAT3 (Tyr705) signaling. In addition, we found that CPX led to the autophagic death of GC cells by targeting p-STAT3 (Ser727). Interestingly, CPX induces STAT3 ubiquitination, which results in decreased phosphorylation of Ser727, rather than Tyr705. Collectively, our findings provide new insight into STAT3 as an important target underlying CPX antitumor activity and suggest the potential of CPX as a therapeutic agent to treat GC patients.

## Results

### CPX dramatically inhibits GC cell proliferation in vitro

**Fig. 1:**
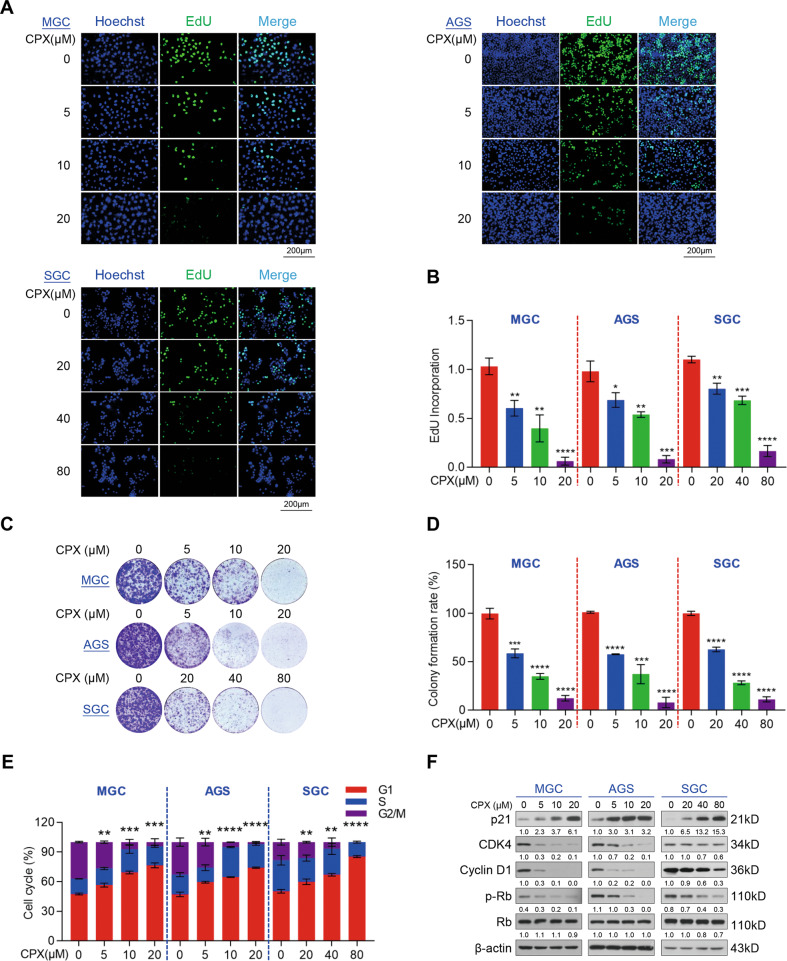
CPX remarkably inhibits GC cell proliferation in vitro. **A**, **B** Proliferation of MGC, AGS, and SGC cells treated with various concentrations of CPX for 24 h. Cell proliferation was detected using an EdU cell proliferation kit with Alexa Fluor 488 (Scale bar, 200 µm) (**A**). EdU incorporation was quantified using ImageJ Plus software (**B**). Data were shown as mean ± SD (*n* = 3, *P < 0.05, **P < 0.01, ***P < 0.001, ****P < 0.0001). **C**, **D** Colony formation of MGC, AGS, and SGC cells treated with various concentrations of CPX for 1–2 weeks. Cell colonies were stained with 0.1% crystal violet solution (**C**). Colony numbers were counted using ImageJ Plus software (**D**). Data were shown as mean ± SD (*n* = 3, ***P < 0.001, ****P < 0.0001). **E** Cell cycle arrest assay analysis of MGC, AGS, and SGC cells treated with a serial dose of CPX for 24 h. The percentage of cell cycle distribution was analyzed using flow cytometry. Data were shown as mean ± SD (*n* = 3, **P < 0.01, ***P < 0.001, ****P < 0.0001). **F** The protein levels of cell cycle-related proteins were examined using Western blotting analysis.

To evaluate the antitumor activity of CPX in vitro, we performed cellular proliferation and viability assays. GC cell lines, such as MGC, AGS, and SGC cells, were treated with a serial dilution of CPX as indicated for 24 h, and cell proliferation and viability were assessed using EdU incorporation and MTT assay, respectively. CPX markedly inhibited the proliferation of all tested cell lines and reduced their viability in a dose-dependent manner (Fig. 1A, B). Compared to MGC, AGS, and SGC cells, the normal human gastric epithelial cell, GES-1, displayed higher tolerance to CPX (Fig. S1a). CPX also markedly suppressed the colony-forming ability of GC cells in a dose-dependent manner (Fig. 1C, D). In addition, we treated GC cell lines with the indicated concentration of CPX for 1, 2, 3, 4, and 5 days and found that CPX also reduced cell viability in a time-dependent manner (Fig. S1b). To further investigate the underlying mechanism of CPX-inhibited GC cell proliferation, we assessed the cell cycle distribution of GC cells treated with CPX using flow cytometry. As shown in Fig. 1E and Fig. S2, CPX induced G1/S arrest in MGC, AGS, and SGC cells. Furthermore, the protein levels of CDK4, Cyclin D1, and p-Rb/Rb were all decreased, while p21, an inhibitor of cyclin-dependent kinases, was remarkably increased in CPX-treated cells (Fig. 1F). Taken together, these findings indicate that CPX may arrest the cell cycle of GC cells and thus suppress their proliferation and viability in vitro.

### CPX induces autophagic death in GC cells

**Fig. 2:**
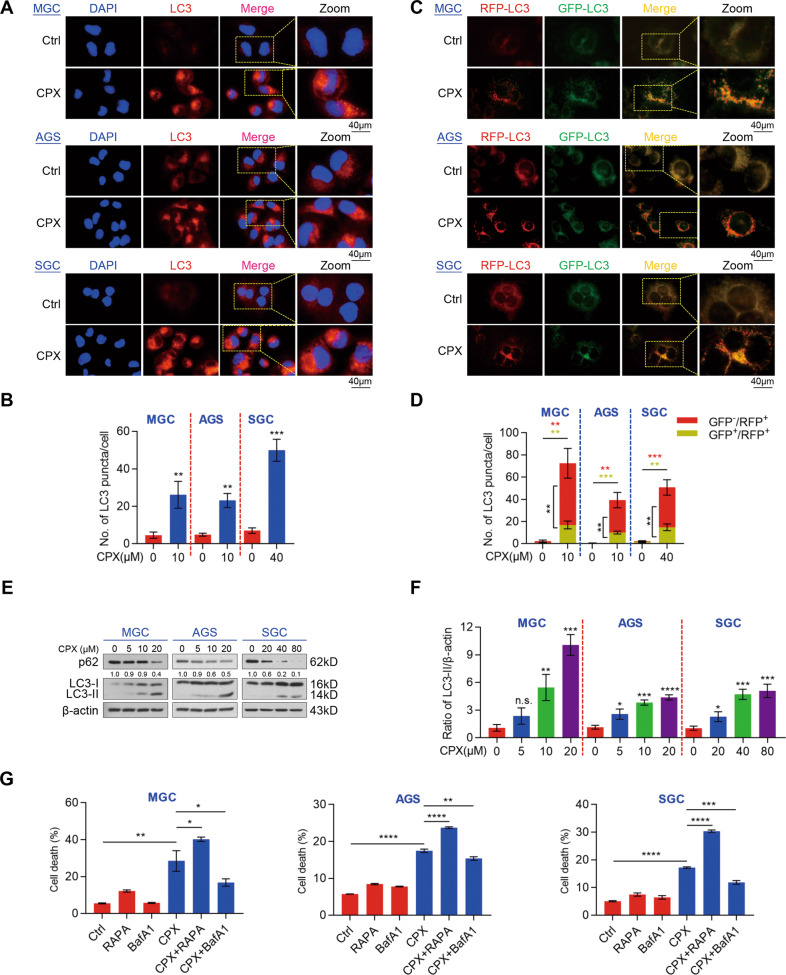
CPX induces autophagic death of GC cells. **A**, **B** The LC3 fluorescence in GC cells treated with CPX at the indicated concentration (MGC: 10 µM; AGS: 10 µM; SGC: 40 µM) for 24 h. Representative images of endogenous LC3 puncta (Scale bar, 40 µm) (**A**). The relative ratio of LC3 puncta to cell number was quantified using ImageJ Plus software (**B**). Data were shown as mean ± SD (*n* = 3, **P < 0.01, ***P < 0.001).**C**, **D** GC cells were transiently transfected mRFP-GFP-tagged LC3 and treated with CPX at the indicated concentration (MGC: 10 µM; AGS: 10 µM; SGC: 40 µM) for 24 h. Representative images of red or green LC3 puncta (Scale bar, 40 µm) (**C**). The relative ratio of red puncta (autolysosome, GFP^-^/RFP^+^) to yellow puncta (autophagosome, GFP^+^/RFP^+^) was quantified using ImageJ Plus software (**D**). Data were shown as mean ± SD (*n* = 3, **P < 0.01, ***P < 0.001). **E**, **F** Western blotting analysis of autophagy-related protein LC3 and p62 in MGC, AGS, and SGC cells treated with various concentrations of CPX for 24 h. Representative protein levels of LC3 and p62 were shown (**E**) and the relative ratio of LC3-II to β-actin from three independent experiments (**F**). Data were shown as mean ± SD (*n* = 3, *P < 0.05, ** *P* < 0.01, ***P < 0.001, ****P < 0.0001, or n.s., not significant by unpaired Student’s *t* test). **G** Cell death of GC cells co-treated with CPX (MGC: 10 µM; AGS: 10 µM; SGC: 40 µM) and RAPA (MGC: 50 nM; AGS: 50 nM; SGC: 100 nM) or BafA1 (20 nM) for 24 h and stained with PI was analyzed using flow cytometry. Data were shown as mean ± SD (*n* = 3, *P < 0.05, **P < 0.01, ***P < 0.001, ****P < 0.0001).

Previous studies have highlighted the crucial activity of CPX in regulating autophagy [25, 26]. We thus evaluated the autophagy of GC cells treated with CPX and observed the accumulation of endogenous LC3 puncta (autophagic vesicles) in CPX-treated GC cells (Fig. 2A, B). In addition, we expressed a tandem monomeric RFP-GFP-tagged LC3 in GC cells and found that CPX-treated cells contained abundant GFP^-^/RFP^+^-LC3 and GFP^+^/RFP^+^-LC3 compared to the control treatment, implying the formation of autophagosomes and autolysosomes in CPX-treated GC cells (Fig. 2C, D). To further clarify whether CPX promoted autophagy, we examined the levels of autophagy-related proteins, LC3 and p62. Our data showed that CPX treatment dramatically increased the ratio of LC3-II to β-actin, but reduced the levels of p62, a well-known autophagic substrate [27], in all treated GC cells. This occurred in a dose-dependent manner, indicating the enhanced formation of autophagosomes (Fig. 2E, F). Previous studies have reported that CPX induces apoptosis of CRC cells and that autophagy can drive apoptosis [7, 25, 28]. We stained cells with annexin V-fluorescein isothiocyanate (FITC)/ propidium iodide (PI) to determine whether the cytotoxicity of CPX-induced autophagy in GC cells depended on apoptosis. Surprisingly, data from the annexin V-FITC/PI apoptosis assay revealed no significant increase in CPX-treated cells (Fig. S3). Given that prolonged autophagy activation eventually leads to autophagic death, known as type II cell death [29], we then examined whether CPX-induced autophagy was a protective response or nonapoptotic cell death and found that bafilomycin A1 (BafA1), an autophagy inhibitor, significantly reduced CPX-induced cell death, while rapamycin (RAPA), an autophagy activator, dramatically increased CPX-induced cell death (Fig. 2G). These data suggest that CPX appears to reduce GC cell viability through autophagic death rather than apoptosis.

### CPX degrades STAT3 via the ubiquitin-proteasome pathway to trigger proliferation inhibition and autophagic death of GC cells

**Fig. 3:**
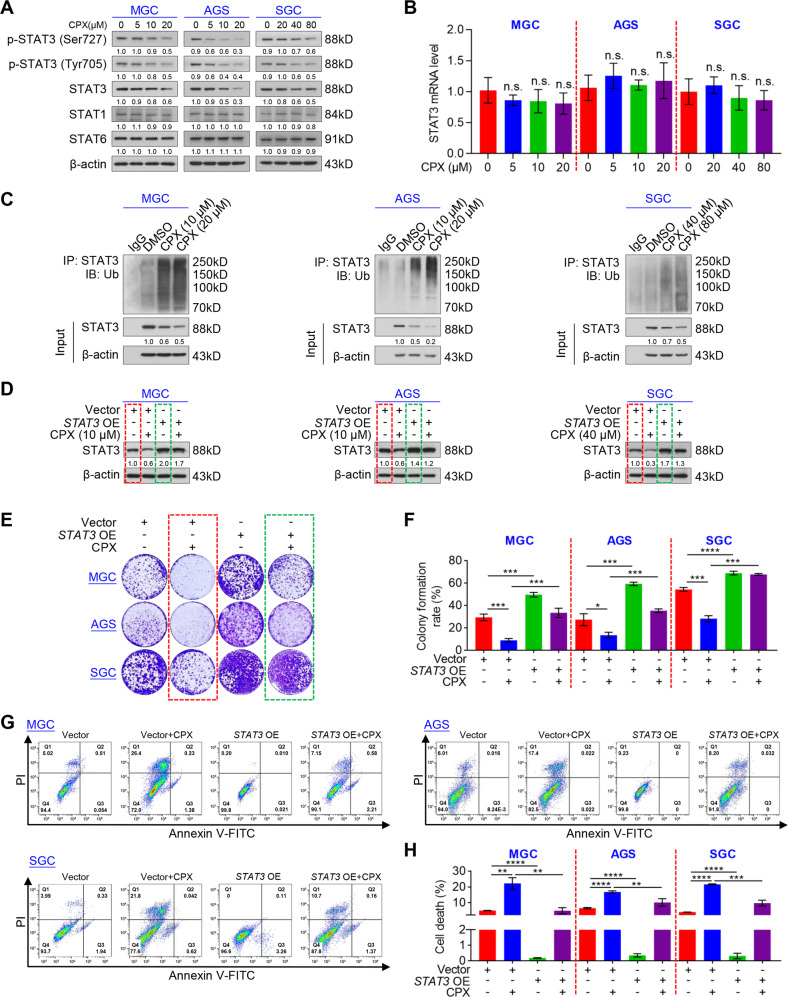
CPX induces proliferation inhibition and autophagic death by promoting STAT3 ubiquitination in GC cells. **A** Western blotting analysis of p-STAT3 (Ser727), p-STAT3 (Tyr705). STAT3, STAT1, and STAT6 in GC cells treated with a serial dose of CPX for 24 h. **B** RT-qPCR analysis of STAT3 mRNA levels in GC cells treated with a different dose of CPX for 24 h. Data were shown as mean ± SD (*n* = 3, n.s., not significant by unpaired Student’s *t* test). **C** Ubiquitination of STAT3 in GC cells treated with a serial dose of CPX for 24 h was detected using immunoprecipitation. **D** Western blotting analysis demonstrating STAT3 overexpression in the indicated GC cells with or without CPX for 24 h. **E**, **F** Colony formation of *STAT3*-overexpressing cell lines treated with CPX for 1–2 weeks. Cell colonies were stained with 0.1% crystal violet solution (**E**). Colony numbers were counted using ImageJ Plus software (**F**). Data were shown as mean ± SD (*n* = 3, *P < 0.05, ***P < 0.001, ****P < 0.0001). **G**, **H** Cells treated with CPX (MGC: 10 µM; AGS: 10 µM; SGC: 40 µM) for 24 h and then stained with annexin V-FITC/PI were determined using flow cytometry (**G**). The cell death rate was plotted followed by statistical analysis (**H**). Data were shown as mean ± SD (*n* = 3, **P < 0.01, *** *P* < 0.001, ****P < 0.0001).

Considering that activation of STAT3 is associated with poor prognosis in GC [13, 14], we investigated whether CPX could regulate STAT3 in GC cells. As expected, CPX dramatically reduced the levels of total STAT3, p-STAT3 (Ser727), and p-STAT3 (Tyr705) in GC cells, but failed to downregulate STAT3 mRNA levels (Fig. 3A, B). However, CPX decreased the levels of total STAT3 in a time-dependent manner, implying that CPX may induce the degradation of STAT3 protein (Fig. S4a). In eukaryotic cells, the lysosome and ubiquitin-proteasome pathways are the two main routes for protein degradation [30, 31]. It has been reported that CPX can lead to ROS accumulation, which is involved in protein degradation via the ubiquitin-proteasome pathway [7, 32]. To dissect how CPX promoted STAT3 degradation in GC cells, we treated GC cells with CPX and a ROS scavenger N-acetyl-cysteine (NAC), an autophagy inhibitor 3-Methyladenine (3-MA), or a proteasome inhibitor Z-Leu-Leu-Leu-al (MG132) and Carfilzomib. As shown in Fig. S4b, MG132 and Carfilzomib, but not NAC or 3-MA, blocked CPX-induced reduction in STAT3. Furthermore, treatment of GC cells with three different autophagy inhibitors, 3-MA, BafA1, or chloroquine (CQ), failed to increase STAT3 expression, whereas MG132 increased STAT3 expression in a dose-dependent manner (Fig. S4c, d). In addition, the ubiquitination of STAT3 was markedly increased in CPX-treated cells (Fig. 3C). These data indicate that CPX degrades STAT3 likely through the ubiquitin-proteasome rather than the lysosome pathway.

To check further verify whether CPX-induced downregulation of STAT3 was involved in GC suppression, we constructed a lentivirus-packaged overexpression plasmid targeting STAT3 (*STAT3* OE) and a negative control plasmid (Vector). GC cell lines MGC, AGS, and SGC were infected with lentivirus-packaged plasmids and the endogenous STAT3 was successfully overexpressed (Fig. 3D). Subsequently, we evaluated cell proliferation and autophagic death in GC cells stably overexpressing STAT3 under CPX treatment. As shown in Fig. 3E, the colony formation ability of *STAT3*-overexpressing cells was stronger than that of vector cells when treated with CPX (Fig. 3E, F). Furthermore, overexpressed STAT3 significantly inhibited CPX-induced autophagic death in GC cells (Fig. 3G, H). These results suggest that CPX induces proliferation inhibition and autophagic death by suppressing STAT3 protein levels.

### CPX inhibits the p-Src (Tyr416)/p-STAT3 (Tyr705) pathway to hinder GC cell proliferation

**Fig. 4:**
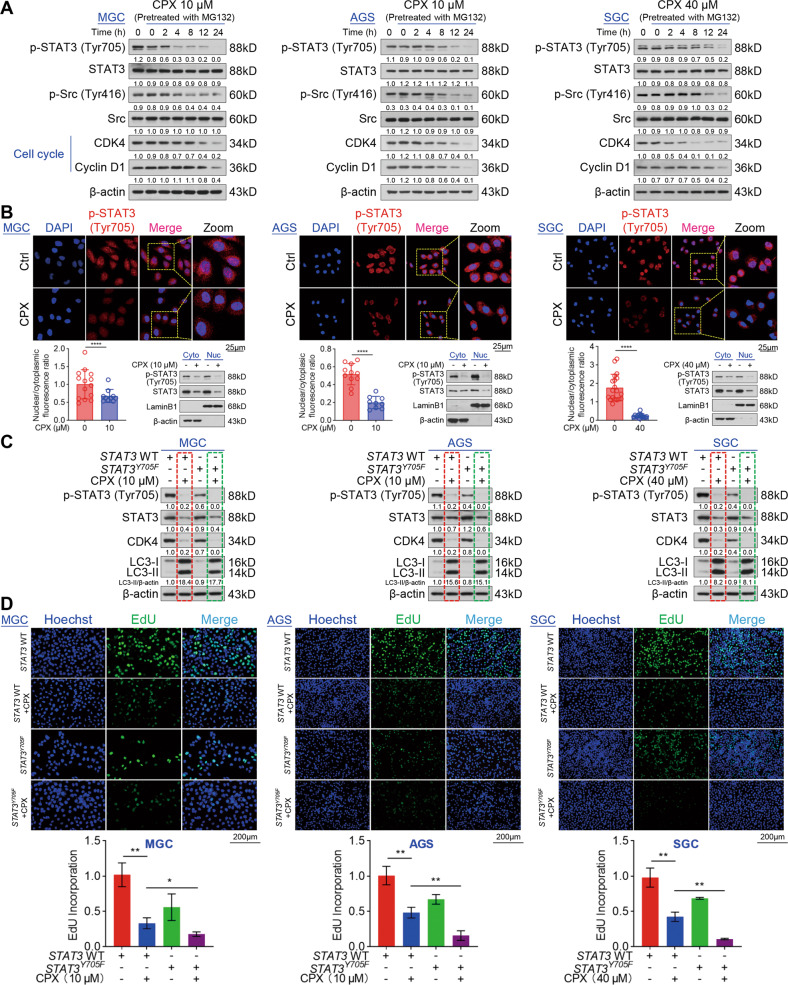
CPX decreases p-STAT3 (Tyr705) to promote proliferation inhibition in GC cells. **A** The protein levels of p-STAT3 (Tyr705), STAT3, p-Src (Tyr416), Src, CDK4, and Cyclin D1 in GC cells pretreated with MG132 (MGC: 100 nM; AGS: 100 nM; SGC: 200 nM) for 24 h and then treated with CPX at different times. **B** The levels of p-STAT3 (Tyr705) in the nucleus and cytoplasm of GC cells treated with CPX (MGC: 10 µM; AGS: 10 µM; SGC: 40 µM) for 24 h. Representative images of p-STAT3 (Tyr705) and the ratio of nuclear to cytoplasmic fluorescence were shown (Scale bar, 25 µm). The protein levels of p-STAT3 (Tyr705) in the nucleus and cytoplasm of GC cells were analyzed by nuclear/cytosolic fractionation and Western blotting. Data were shown as mean ± SD (*n* = 3, ****P < 0.0001). **C** Western blotting analysis of p-STAT3 (Tyr705), STAT3, CDK4, and LC3 protein levels in *STAT3*^*Y705F*^ cell lines treated with CPX for 24 h. **D** The proliferation of *STAT3*^*Y705F*^ GC cells after being treated with CPX for 24 h was detected using an EdU Cell Proliferation kit with Alexa Fluor 488. EdU incorporation was quantified using ImageJ Plus software (Scale bar, 200 µm). Data were shown as mean ± SD (*n* = 3, *P < 0.05, **P < 0.01).

To further study the role of CPX in the expression of STAT3 and its two phosphorylation sites, we treated GC cells with MG132 and CPX at different times. Surprisingly, MG132 blocked the effect of CPX on the expression of total STAT3 and p-STAT3 (Ser727), but not p-STAT3 (Tyr705), which suggested that p-STAT3 (Ser727) levels may be dependent on STAT3 protein level, while other mechanisms may be responsible for CPX-reduced reduction in p-STAT3 (Tyr705) levels in GC cells (Fig. S5a). Previous studies have reported that blocking Src tyrosine kinase activity can inhibit STAT3 signaling in melanoma and breast carcinoma cells [33, 34]. Consistently, we found that CPX decreased the levels of p-STAT3 (Tyr705), p-Src (Tyr416), and the cell cycle-related proteins CDK4 and Cyclin D1 without any effect on total STAT3 and Src levels in GC cells pretreated with MG132, indicating that CPX may decrease p-STAT3 (Tyr705) level via inhibiting p-Src (Tyr416) (Fig. 4A). It was known that p-STAT3 (Tyr705) in the nucleus can promote tumor growth [35]. Indeed, we observed that CPX treatment decreased the protein levels of p-STAT3 (Tyr705) in the nucleus and cytoplasm (Fig. 4B). To investigate whether CPX-inhibited GC cell proliferation via the p-Src (Tyr416)/p-STAT3 (Tyr705) pathway, we analyzed their expression under different conditions. As shown in Fig. S5b, interleukin-4 (IL-4) upregulated the expression of p-STAT3 (Tyr705), p-Src (Tyr416), and total Src in a dose-dependent manner, but did not change the protein levels of total STAT3 and p-STAT3 (Ser727). Furthermore, IL-4 remarkedly reversed the reduction in the levels of p-STAT3 (Tyr705), p-Src (Tyr416), and CDK4 induced by CPX (Fig. S5c). Consistently, pretreatment of GC cells with IL-4 significantly rescued GC cells from CPX-induced inhibition in cell proliferation (Fig. S5d). We then constructed *STAT3*^*Y705F*^ mutant GC cell lines to further determine the effects of p-STAT3 (Tyr705) on GC cell proliferation. As shown in Fig. 4C, D, treatment of CPX in non-phosphorylated *STAT3*^*Y705F*^ mutant cell lines promoted CPX-induced suppression of CDK4 and EdU incorporation, but not LC3 protein, suggesting that p-STAT3 (Tyr705) is not involved in CPX-induced autophagy in GC cells. These results indicate that CPX suppresses GC cell proliferation probably via the p-Src (Tyr416)/p-STAT3 (Tyr705) pathway.

### CPX induces autophagy by targeting p-STAT3 (Ser727)

**Fig. 5:**
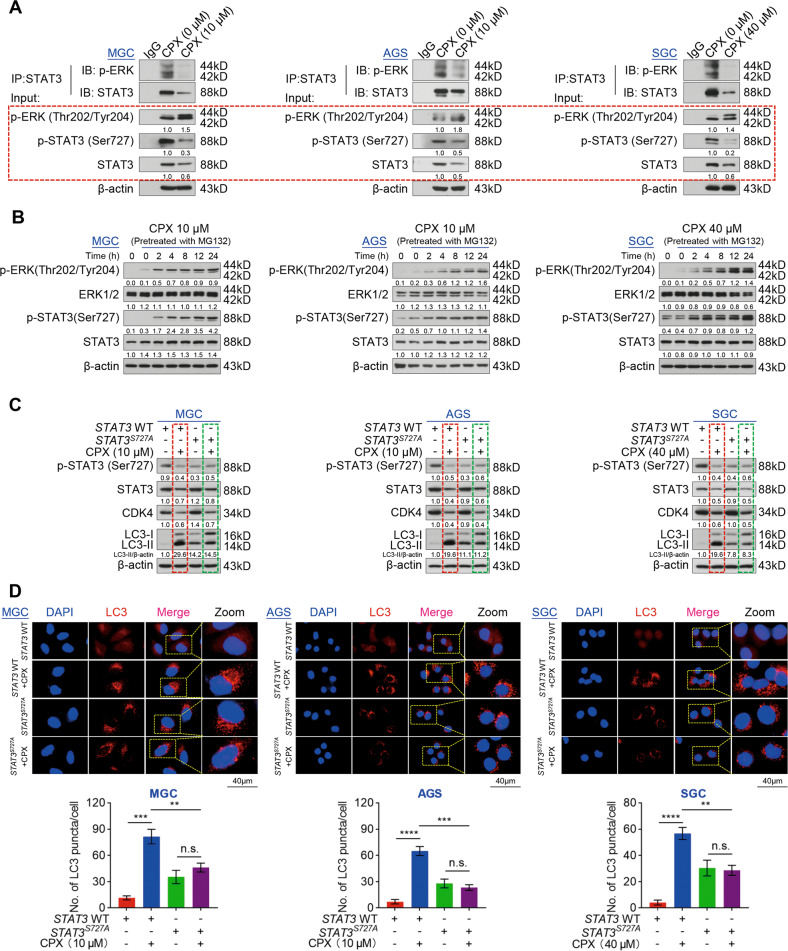
CPX induces autophagy of GC cells through targeting p-STAT3 (Ser727). **A** The interaction between p-ERK (Thr202/Tyr204) and STAT3 in GC cells treated with CPX for 24 h. **B** The protein levels of p-ERK (Thr202/Tyr204), ERK1/2, p-STAT3 (Ser727), and STAT3 in GC cells pretreated with MG132 (MGC: 100 nM; AGS: 100 nM; SGC: 200 nM) for 24 h and then treated with CPX at different times. **C** Western blotting analysis of p-STAT3 (Ser727), STAT3, CDK4, and LC3 protein levels in *STAT3*^*S727A*^ GC cells treated with CPX for 24 h. **D** The endogenous LC3 puncta fluorescence in *STAT3*^*S727A*^ GC cells treated with CPX for 24 h. The ratio of LC3 puncta to cell number was quantified using ImageJ Plus software (Scale bar, 40 µm). Data were shown as mean ± SD (*n* = 3, **P < 0.01, *** *P* < 0.001, **** *P* < 0.0001, or n.s., not significant by unpaired Student’s *t* test).

Next, we sought to identify the role of p-STAT3 (Ser727) in CPX-induced autophagy and inhibition of proliferation in GC cells. Previous studies have shown that p-ERK could promote the phosphorylation of STAT3 at Ser727 [36, 37]. Therefore, we investigated whether p-ERK (Thr202/Tyr204) could regulate the p-STAT3 (Ser727) level in GC cells. We found that endogenous STAT3 was coimmunoprecipitated with p-ERK (Thr202/Tyr204), while the interaction of STAT3 with p-ERK (Thr202/Tyr204) was drastically decreased by CPX treatment (Fig. 5A). We also observed that CPX increased p-ERK (Thr202/Tyr204) levels but decreased the levels of total STAT3 and p-STAT3 (Ser727) (Fig. 5A). Considering that p-STAT3 (Ser727) levels in GC cells were dependent on total STAT3 protein level (Fig. S5a), we examined p-ERK (Thr202/Tyr204) and p-STAT3 (Ser727) levels with or without MG132. As shown in Fig. S6a, CPX increased p-ERK (Thr202/Tyr204) levels but reduced the levels of total STAT3 and p-STAT3 (Ser727). However, in GC cells pretreated with MG132, CPX did not alter STAT3 levels, but increased the protein levels of both p-ERK (Thr202/Tyr204) and p-STAT3 (Ser727) in a time-dependent manner (Fig. 5B). These results suggest that p-ERK could regulate p-STAT3 (Ser727) levels in GC cells. Given that p-ERK plays an essential role in autophagy [38, 39], we used PD98059, an inhibitor of ERK1/2 signaling, to treat GC cells and found that PD98059 led to a prominent reduction in the phosphorylation of STAT3 at Ser727 without any effects on the phosphorylation of STAT3 at Tyr705 and total STAT3 protein levels (Fig. S6b). Interestingly, the combined treatment of CPX and PD98059 reduced phosphorylated STAT3 at Ser727, enhanced the conversion of LC3-I to LC3-II, and reduced p62 levels compared with the treatment of CPX alone (Fig. S6c). Consistently, endogenous LC3 puncta were significantly increased in the combined treatment (Fig. S6d).

To further determine whether p-STAT3 (Ser727) is involved in CPX-triggered autophagy of GC cells, we then constructed *STAT3*^*S727A*^ mutant GC cell lines. Unexpectedly, we found that CPX treatment significantly reduced LC3-mediated autophagy in cells expressing the non-phosphorylated *STAT3*^*S727A*^ mutant compared to the *STAT3* WT cells (Fig. 5C, D). Moreover, in *STAT3*^*S727A*^ mutant GC cell lines, CPX did not markedly regulate the ratio of LC3-II to β-actin and LC3 puncta, suggesting that p-STAT3 (Ser727) is the target of CPX-induced autophagy in GC cells (Fig. 5C, D). Simultaneously, *STAT3*^*S727A*^ mutant did not change CPX-induced inhibition of CDK4 and EdU incorporation (Fig. 5C and Fig. S7). Together, these findings reveal that CPX induces autophagy through targeting p-STAT3 (Ser727).

### CPX inhibits GC xenograft tumor growth in vivo

**Fig. 6:**
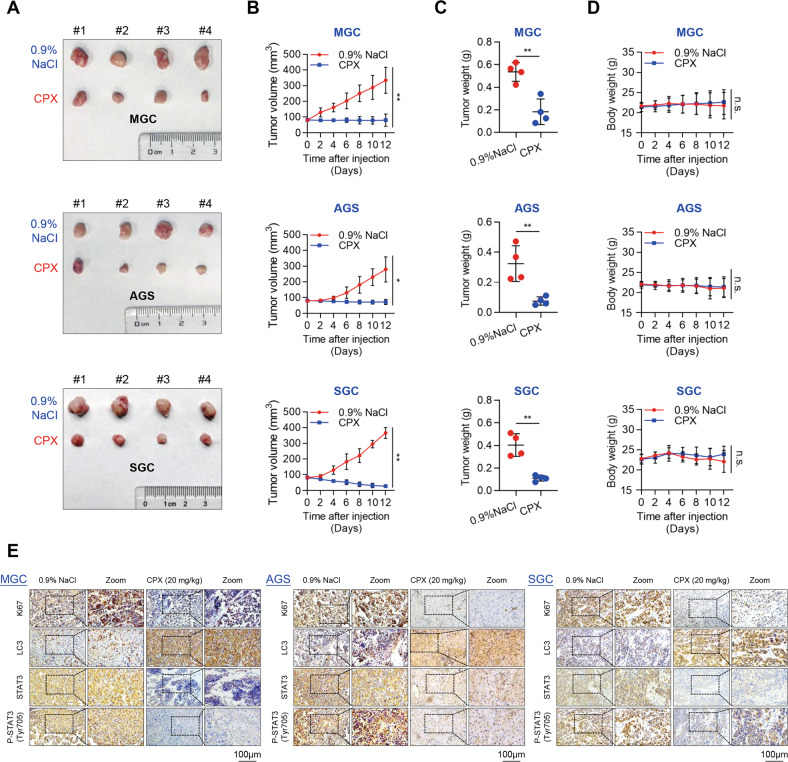
CPX inhibits tumor growth in a mouse xenograft model of GC. **A**–**D** Tumor-bearing nude mice of MGC, AGS, and SGC cells (4 mice per group) were intraperitoneally injected with 0.9% NaCl or CPX (20 mg/kg), respectively. Images of dissected tumors from tumor-bearing mice were shown (**A**). Tumor volume was measured once in 2 days (**B**). Tumor weight was measured after 12 days of consecutive injections (**C**). Changes in the mean body weight from mice treated with 0.9% NaCl or CPX (**D**). Data were shown as mean ± SD (*n* = 4, *P < 0.05, **P < 0.01, or n.s., not significant by unpaired Student’s *t* test). **E** Images of immunohistochemistry staining of Ki67, LC3, STAT3, and p-STAT3 (Tyr705) in the tumor of GC mouse xenograft model intraperitoneally injected with 0.9% NaCl or CPX (Scale bar, 100 µm).

To evaluate the antitumor tumor growth potential of CPX in vivo, we used a mouse xenograft model of MGC, AGS, and SGC cells to explore the tumor-suppressive properties of CPX. We found that CPX significantly suppressed GC tumor growth when compared to the control group (Fig. 6A, B). Moreover, CPX also remarkably decreased the tumor weight (Fig. 6C). However, the mice tolerated the CPX treatment well, without notable body weight loss (Fig. 6D). We also confirmed that CPX treatment dramatically decreased proliferation (Ki67 staining) and increased autophagy (LC3 staining) compared to 0.9% NaCl treatment, while decreasing total STAT3 and p-STAT3 (Tyr705) levels in GC xenograft tumors (Fig. 6E). Collectively, these data suggest that CPX remarkably inhibits GC xenograft tumor growth in vivo.

## Discussion

CPX, an antifungal agent off-patent for several decades, was recently found to have remarkable antitumor effects via growth arrest or cell death induction in various cancers [8, 9, 25, 40]. Here, we demonstrated the role of CPX in regulating proliferation inhibition and autophagic death of GC cells. Specifically, CPX reduces p-STAT3 (Tyr705) expression via inhibiting upstream kinase p-Src (Tyr416) rather than STAT3, leading to the proliferation inhibition of GC cells. We also found that CPX induces the degradation of STAT3 via the ubiquitin-proteasome pathway, leading to the inhibition of p-STAT3 (Ser727) and subsequent autophagy provocation of GC cells. Our findings provide evidence that loss of STAT3 and its two phosphorylation sites contributes to the antitumor effect of CPX treatment, namely promoting growth arrest and autophagic cell death.

CPX has been considered a potential antitumor agent due to its therapeutic potential in preclinical models of various cancers. For instance, CPX inhibits tumorigenesis by inducing apoptosis and protective autophagy in multiple cancers, such as colorectal cancer, glioma, and cervical carcinoma [7, 9, 25, 41]. However, our study showed that CPX triggers autophagic death rather than apoptotic death in GC cells. Similar to our findings, it has been reported that SH003, a powerful herbal formula, activates autophagic cell death under hypoxia via the STAT3-G9a axis in GC [42]. Another study demonstrated that kaempferol, a flavonoid, induces autophagic death via the HDAC-G9a axis in GC [43]. Thus, GC cells appear to prefer to undergo autophagic death rather than apoptosis when treated with antitumor agents. Although both autophagy and apoptosis can induce programmed cell death, their underlying molecular mechanisms are widely divergent. Further studies are needed to elucidate why CPX induces autophagic death rather than apoptosis in GC cells.

Autophagy is a dynamic cellular process involving the lysosomal degradation of cellular materials, which prevents intracellular toxic protein accumulation and clears damaged organelles. However, various cancer cell lines, such as breast cancer cells, glioma cells, and GC cells, undergo elongated or intensive autophagy, which leads to remarkable autophagic cell death [42, 44]. Therefore, autophagy is considered to be a temporary stress survival process, but it can also promote cell death, which suggests that excessive or prolonged autophagy triggered by antitumor drugs may be a useful strategy for the treatment of tumors. In this study, we observed the increased autophagic vacuoles of autophagosomes or autolysosomes in CPX-treated GC cells. Accumulating evidence showed that autophagy could trigger apoptosis, another type of programmed cell death [28, 45]. However, we did not observe annexin-V-FITC-stained apoptosis in CPX-treated GC cells. We found about a 2% apoptosis rate of CPX-treated MGC cells, but it is still below the 15% cutoff criterion for effective apoptosis ratio, which indicates that CPX-induced autophagy cannot trigger apoptosis in GC cells. Furthermore, autophagy inhibitor BafA1 suppresses, whereas autophagy activator RAPA exacerbates CPX-induced autophagic cell death, which confirms that autophagy inhibitor protects GC cells from CPX-induced autophagic cell death. It has been reported that inhibition of STAT3 stimulates autophagic flux and induces autophagic rather than apoptotic cell death in malignant glioma [40, 46, 47], suggesting that STAT3 may be the main factor responsible for CPX-induced autophagic death rather than apoptosis in GC cells. Kroemer’s team revealed that STAT3 in the cytoplasm suppresses autophagy by sequestering the protein kinase R (PKR), thus preventing the phosphorylation of eukaryotic translation initiation factor 2α (eIF2α) and activating the autophagic process, downstream the transcription factor (ATF4) [40, 48, 49]. Wegrzyn et al. pointed out the ability of STAT3 to promote the interaction with complexes I and II of the electron transport chain (ETC) to regulate their activity and reduce ROS production [50]. Our previous studies found that CPX impairs the mitochondrial respiration chain and induces ROS production in CRC and NSCLC cells, which leads to the activation of the protein kinase RNA-like endoplasmic reticulum kinase (PERK)-eIF2α-ATF4 pathway [7, 8]. Therefore, CPX may regulate mitochondrial function, cellular bioenergetics, and endoplasmic reticulum stress-associated cell death by targeting STAT3 in GC cells.

STAT3, a latent transcription factor, is associated with tumorigenesis [51–53]. It is activated in various tumors and then facilitates tumor cell proliferation, angiogenesis, metastasis, and anti-apoptosis [54]. Therefore, inhibition of STAT3 is a promising therapeutic strategy for cancer treatment. However, the structure of the STAT family is highly conserved, making it very challenging to develop highly selective STAT3 inhibitors. Recently, Bai et al. reported that SD-36, a small-molecule degrader of STAT3, exhibits a great immunologic tolerance and promotes durable tumor regression in xenograft models [55]. SD-36 is designed based on proteolysis targeting chimera technology. It can simultaneously bind to STAT3 and E3 ubiquitin ligase, leading to STAT3 degradation through the ubiquitin-proteasome pathway [55]. Proteasome inhibitor carfilzomib and NEDD8-activating E1 enzyme inhibitor MLN4924 block SD-36-induced degradation of STAT3 [55]. Here, we also found that CPX promotes STAT3 ubiquitination, and MG132 and carfilzomib significantly suppress CPX-induced reduction in STAT3. However, CPX-induced STAT3 degradation only decreases p-STAT3 (Ser727) levels and simultaneously CPX reduces p-STAT3 (Tyr705) levels via inhibiting p-Src (Tyr416). Similarly, Liu *et al*. reported that STAT3 is activated by phosphorylation on Ser727, but not on Tyr705 to maintain macrophage survival [56]. Several recent reports also indicate that the pro-tumorigenesis activity of STAT3 depends on p-STAT3 (Ser727), but not p-STAT3 (Tyr705). For instance, abrogation of STAT3 phosphorylation at Ser727 rather than at Tyr705 hinders K-Ras-dependent hematopoietic neoplasia growth [57]. Conversely, activating phosphorylation of STAT3 at Ser727 significantly triggers hepatoma cell survival, and p-STAT3 (Ser727) accelerates breast cancer growth via exacerbating ROS accumulation [37, 58]. We found that CPX inhibits STAT3 to decrease its interaction with p-ERK (Thr202/Tyr204) and p-STAT3 (Ser727) levels, which promotes autophagy through activating LC3-II and suppressing p62 expression. Moreover, *STAT3*^*S727A*^ mutant is resistant to CPX-induced autophagy, indicating that p-STAT3 (Ser727) is important for CPX-induced autophagic death of GC cells. As another critical STAT3 phosphorylation site, p-STAT3 (Tyr705) interacts with programmed death ligand 1 to facilitate tumor necrosis [59, 60]. Furthermore, p-STAT3 (Tyr705) is a major kinase-independent target of sorafenib in hepatocellular carcinoma, and dephosphorylation at Tyr705 enhances the therapeutic efficacy of sorafenib in glioblastoma [61]. In our study, although CPX decreases p-STAT3 (Tyr705) levels in a STAT3-independent manner, CPX drastically suppresses phosphorylation of Src at Tyr416 to reduce p-STAT3 (Tyr705) levels, thereby inhibiting GC cell proliferation. These studies suggest that CPX may be a new therapeutic strategy for treating GC via targeting STAT3 and its phosphorylation at Ser727 and Tyr705 sites.

**Fig. 7:**
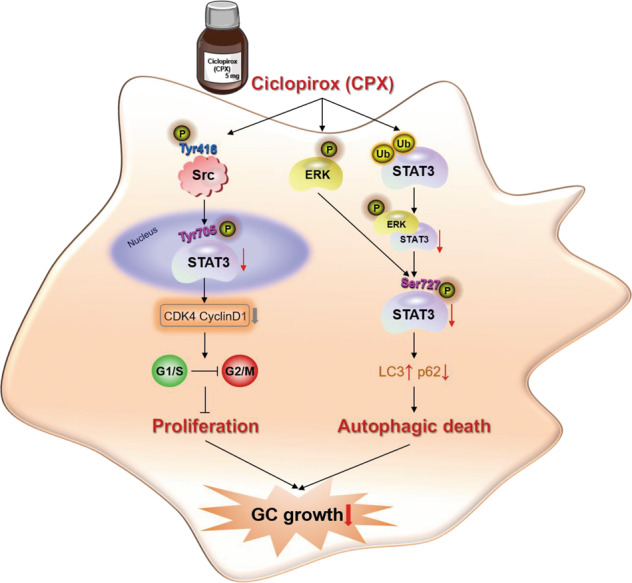
The proposed antitumorigenic mechanism of CPX on GC growth. CPX treatment induces cell cycle arrest and then inhibits GC cell proliferation by regulating the p-Src (Tyr416)/p-STAT3 (Tyr705) pathway. Moreover, CPX enhances autophagic death by inducing total STAT3 ubiquitination and decreasing p-STAT3 (Ser727) levels in GC cells.

In summary, we demonstrated that CPX inhibits GC tumorigenesis via suppressing proliferation and promoting autophagic cell death (Fig. 7). Furthermore, our study reveals a connection between STAT3 phosphorylation and autophagic cell death rather than apoptosis in GC cells. These findings provide important insights into the antitumor mechanism of CPX in GC and suggest that CPX may be a potential antitumor agent for GC treatment.

## Materials and methods

### Cell lines and cell culture

Human gastric cancer cells were kindly donated by Prof. Xiangyang Xue, Wenzhou Medical University. MGC, SGC, and GES-1 cells were cultured in Dulbecco’s modified Eagle’s medium (DMEM, Life Technologies, NY, USA), while AGS cells were cultured in RPMI-1640 medium (Life Technologies, NY, USA). All culture media were supplemented with 10% fetal bovine serum (FBS, ExCell Bio, Shanghai, China) and 1% penicillin-streptomycin (Beyotime Biotechnology, Shanghai, China) at 37 °C in a humidified incubator with 5% CO_2_. Cell lines were authenticated by short tandem repeats (STR) and routinely checked and confirmed to be mycoplasma free during this study.

### Reagents and antibodies

MTT Cell Proliferation and Cytotoxicity Assay Kit, Crystal violet solution, and NAC were purchased from Beyotime Biotechnology (Shanghai, China). EdU Cell Proliferation kit with Alexa Fluor 488, cell cycle analysis kit, and annexin V-FITC/PI apoptosis detection kit were purchased from meilunbio® (Dalian, China). HiScript II Q RT SuperMix for qPCR (+gDNA wiper) and ChamQTM SYBR qPCR Master Mix were purchased from Vazyme Biotech (Nanjing, China). CPX olamine was purchased from Dibo Biotechnology Co. (Shanghai, China). 3-MA, MG132, BafA1, RAPA, and CQ were purchased from MedChemExpress (New Jersey, USA). The Pierce BCA^TM^ protein assay kit was purchased from Thermo Fisher Scientific (MA, USA). Recombinant human IL-4 protein was purchased from R&D Systems (MN, USA). PD98059 was purchased from GLPBIO (CA, USA). Carfilzomib was purchased from Sigma-Aldrich (MO, USA). Alexa Fluor Plus 555 and 4′,6-diamidino-2-phenylindole (DAPI) were purchased from Invitrogen (Thermo Fisher Scientific, MA, USA). 3, 3-diaminobenzidine (DAB) Kit was purchased from Abcam (Cambridge, UK).

Primary antibodies used in this study were: anti-STAT3 (10253-2-AP), anti-STAT6 (51073-1-AP) anti-β-actin (66009-1-AP), anti-Rb (17218-1-AP), anti-Cyclin D1 (26539-1-AP), anti-p21 (10355-1-AP) and anti-Lamin B1 (12987-1-AP). These antibodies were purchased from ProteinTech (Wuhan, China). Anti-LC3B (ab192890), and anti-p62 (ab56416) were purchased from Abcam (Cambridge, UK). Anti-p-STAT3(Tyr705) (9145L), anti-STAT1 (9172S), anti-p-Src (Tyr416) (2101S), anti-Src (2108S), anti-p-ERK (Thr202/Tyr204) (4377S), anti-p-Rb (8516T), and ERK1/2 (4696S) were purchased from Cell Signaling Technology (MA, USA). Anti-Ubiquitin (sc-8017) was purchased from Santa Cruz Biotechnology (TX, USA). Anti-p-STAT3(Ser727) (612543) was purchased from BD Biosciences (NJ, USA) and anti-p-STAT3(Ser727) (TA3294) was purchased from Abmart (Shanghai, China). Anti-CDK4 (R23889) was purchased from Zen Bioscience (Chengdu, China). Alexa Fluor Plus 555 was purchased from Thermo Fisher Scientific (MA, USA). Horseradish peroxidase (HRP)-conjugated secondary antibodies (Goat anti-rabbit and goat anti-mouse) were purchased from Beyotime Biotechnology (Shanghai, China).

### Cell proliferation and viability analysis

Cell proliferation and viability were determined using an MTT assay kit. Briefly, MGC, AGS, and SGC cells were seeded into 96-well plates at a density of 5 × 10^3^ cells/per well and incubated overnight. The cell number was counted by flow cytometry (BD Accuri^TM^ C6 plus flow cytometer). Next day, cells were cultured with or without a serial dilution of CPX (MGC: 0, 5, 10, and 20 μM; AGS: 0, 5, 10, and 20 μM; SGC: 0, 20, 40, and 80 μM) for 1, 2, 3, 4, and 5 days. Cells were then stained with 10 μL of MTT reagent (5 mg/ml) for 4 h at the endpoint. After the crystals were dissolved completely, 100 μL of samples were transferred to a 96-well plate and measured at 570 nm by a plate reader (Molecular Devices, USA).

Cell proliferation was also detected by an EdU assay kit (Meilunbio, Dalian, China). MGC, AGS, and SGC cells were seeded on coverslips in 24-well plates and cultured overnight. Cells were then treated with CPX for 24 h and stained with EdU for 12 h at 37 °C in a humidified incubator with 5% CO_2_. Finally, the EdU assay kit analyzed cell proliferation according to the manufacturer’s instructions and observed using fluorescence microscopy (Nexcope, China).

### Colony formation assay

MGC, AGS, and SGC cells were seeded into 6-well plates at a density of 1000 cells per well and cultured at 37 °C in an atmosphere containing 5% CO_2_ for 1–2 weeks until colonies were visible to the naked eye. Cells were treated with CPX (MGC: 0, 5, 10, and 20 μM; AGS: 0, 5, 10, and 20 μM; SGC: 0, 20, 40, and 80 μM) for another 7 days and then stained with crystal violet solution. The same number of dimethyl sulfoxide (DMSO)-treated cells were used as vehicle control. Only clusters with over 50 cells were photographed and counted using ImageJ Plus software.

### Cell cycle distribution and apoptosis analysis

MGC, AGS, and SGC cells were seeded in 60 mm cell culture dishes at 37 °C in an atmosphere containing 5% CO_2_ overnight, followed by treatment with various concentrations of CPX or DMSO for 24 h. Cells were harvested and fixed with 70% ethanol/H_2_O(v/v) overnight and then incubated with PI for 30 min in the dark. Stained cells were analyzed for cell cycle distributions using flow cytometry on a BD LSRFortessa^TM^ X-20 (BD Biosciences, NJ, USA).

MGC, AGS, and SGC cells were seeded in 60 mm cell culture dishes at 37 °C in an atmosphere containing 5% CO_2_ overnight, followed by treatment with CPX, MG132, and/or BafA1 or RAPA for 24 h. Cells were collected, washed with 1 × PBS, and fixed with 1 × binding buffer, followed by incubating with annexin V-FITC/PI in the dark at room temperature for 15 min. Apoptosis and cell death were immediately measured on a BD LSRFortessa^TM^ X-20. A minimum of 10,000 cells were detected per condition.

### Western blotting analysis

Cells were lysed in Triton X-100 cell lysis buffer containing phosphate inhibitor cocktail (APExBIO, Houston, USA) for 20 min on ice, and then centrifuged at 12,000 rpm for 25 min at 4 °C. Aliquots of whole cell lysate (15 μg per lane) were separated by 10% to 12% SDS-polyacrylamide gel electrophoresis and transferred onto a nitrocellulose membrane (Bio-Rad, CA, USA) in Tris-glycine buffer. Blots were blocked at room temperature for 2 h in a blocking buffer (5% non-fat milk in TBST) on a shaker and then incubated with primary antibodies overnight at 4 °C. The membrane was washed in TBST for 3 × 5 min and then incubated with HRP-conjugated secondary antibodies at room temperature for 60–90 min. Immunoreactive proteins were visualized using an ECL reagent (Thermo Fisher Scientific, MA, USA). Images were exposed by BioMax X-ray film (Carestream, Xiamen, China) and quantified using ImageJ Plus software.

### RNA extraction and RT-qPCR

Total RNA from cell samples was extracted using TRIzol Reagent (Thermo Fisher Scientific, MA, USA) following the manufacturer’s instructions, as described [7]. Primers for gene amplification are shown in Table S1.

### Co-immunoprecipitation assay

MGC, AGS, and SGC cells were treated with a serial concentration of CPX for 24 h. Cells were lysed with 1 × Co-IP cell lysis buffer (Cell Signaling Technology, MA, USA) supplemented with a protease inhibitor cocktail for 30 min on ice. The cell lysate was centrifuged at 12,500 rpm for 40 min at 4 °C. The concentration of the supernatant was determined using a Pierce^TM^ BCA Protein Assay Kit (Thermo Fisher Scientific, MA, USA). To clear the lysate, 30 µl of Protein A/G PLUS-Agarose (Santa Cruz Biotechnology, TX, USA) was added into a 1000 μg supernatant for 4 h with rotation at 4 °C. The supernatant was collected after centrifugation for 5 min at 12,000 rpm and then incubated with 4.0 μg of STAT3 antibody (Proteintech, Wuhan, China) overnight at 4 °C. After, 50 µl of Protein A/G PLUS-Agarose was added to the samples. The mixture was rotated for 4 h at 4 °C, followed by centrifugation for 5 min at 12,000 rpm. The supernatant was discarded, and pellets were washed three times with 1 × Co-IP cell lysis buffer. The immunoprecipitated proteins were eluted in 2 × SDS-Loading buffer and then analyzed with Western blotting analysis.

### Constructing GC cell lines

The cDNAs of *STAT3*-overexpressing, *STAT3* WT, and its dominant negative mutants *STAT3*^*Y705*F^ and *STAT3*^*S727A*^ were purchased from Wuhan Yikeyue Biotechnology Co., Ltd (Wuhan, China). Lentiviruses were made in 60 mm dishes by transfecting HEK 293T cells with 3 μg plasmid/dish by using PEI. These lentiviruses were used to transfect MGC, AGS, and SGC cells for 48 h, and then stable transformants were selected to be checked by immunoblot analysis after applying 1.0 μg/ml puromycin (Calbiochem, San Diego, CA) for 5–7 days.

### Preparation of cytoplasmic and nuclear extracts

Cells in 1 × 100 mm cell culture dish were collected, washed, and suspended in 400 µl of ice-cold 1.5% citric acid solution. To prepare cytoplasmic, 200 µl of cell suspensions were then centrifuged at 14,000 × *g* for 30 min at 4 °C and the supernatants were collected and temporarily stored on ice. To prepare nuclear extracts, 200 µl of cell suspensions were homogenized with a pre-cooled glass homogenizer (50 times per sample) and then centrifuged at 800 × *g* for 15 min at 4 °C. The pellets were resuspended in 1 ml of 0.25 M sucrose citric acid solution (0.25 M sucrose, 3.3 mM CaCl_2_, and 0.078 M citric acid solution) and then slowly added to another fresh tube containing 4 ml of 0.88 M sucrose citric acid solution (0.88 M sucrose, 3.3 mM CaCl_2_, and 0.078 M citric acid solution), followed by centrifugation at 3000 × *g* for 10 min at 4 °C. The pellets were washed with 5 mM Tris-HCl-NaCl solution 2 times and centrifuged at 2000 × *g* for 10 min at 4 °C. The protein concentrations of cytoplasmic and nuclear extracts were determined using the Pierce^TM^ BCA Protein Assay Kit.

### Immunofluorescence staining

Immunofluorescence staining was performed using a protocol as described previously [7].

### Animal experiments

The animal experiments were performed by the guidelines of the Institutional Animal Ethics Committee and the University of South China Animal Care Guidelines for the Use of Experimental Animals. Male nude mice were housed under specific pathogen-free conditions. MGC (5 × 10^7^), AGS (2 × 10^7^), and SGC (5 × 10^6^) cells were subcutaneously injected into the left flank of 5-week-old BALB/c athymic nude mice (*n* = 8) (SJA, Hunan, China). When the average tumor volume reached almost ~75 mm^3^, mice were randomized into two groups of four (0.9% NaCl- and CPX-treated group). The mice were then administered an intraperitoneal injection of CPX (20 mg/kg) dissolved in 0.9% NaCl, or 0.9% NaCl alone, once a day for 12 days. Tumor growth was monitored every 2 days by measuring the length and width of the tumor, and tumor volume was calculated by the following formula: volume = (length × width^2^)/2. After 12 days, the mice were sacrificed and photographed, and tumors were dissected, weighed, and fixed.

### Immunohistochemistry assay

Immunohistochemistry assay was performed using a protocol as described previously [8].

### Statistical analysis

Data were repeated at least three times and expressed as mean ± SD. Statistical analysis was performed using SPSS software version 22.0 (SPSS Inc., Chicago, IL, USA), and a *P* value of less than 0.05 was considered statistically significant (*P < 0.05, **P < 0.01, ***P < 0.001, ****P < 0.0001; ns, no significant difference). Graphs were made using GraphPad Prism 8.0 software (GraphPad Software Inc., San Diego, CA, USA).

## Supplementary information

SUPPLEMENTAL MATERIAL

Original Data File

AJ Checklist-CDDIS-22-2047R

Author-contribution-form

## Data Availability

Data is contained within the article or supplementary material.
