# Supplementary material for: Ciclopirox drives growth arrest and autophagic cell death through STAT3 in gastric cancer cells
Source: Cell Death Dis. 2022 Nov 28;13(11):1007. doi: 10.1038/s41419-022-05456-7 (PMC9705325; doi:10.1038/s41419-022-05456-7)
Supplement: Supplementary file 1 — SUPPLEMENTAL MATERIAL [file 41419_2022_5456_MOESM1_ESM.docx]

**Supplementary figures
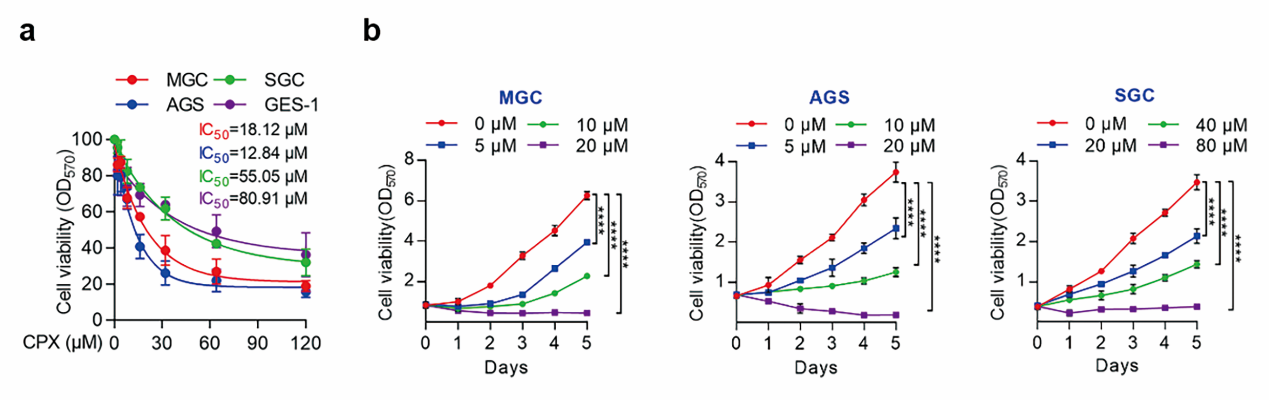
**

**Fig. S1 CPX suppresses GC cell proliferation *in vitro*. a** The IC_50_ values of MGC, AGS, SGC, and GES-1 cells treated with CPX for 24 h were evaluated using MTT cellular proliferation and cytotoxicity assay kit. **b** Proliferation of MGC, AGS, and SGC cells treated with CPX for 5 days was measured by MTT cellular proliferation and cytotoxicity assay kits. Data were shown as mean ± SD (n=3, *****P* < 0.0001).

**
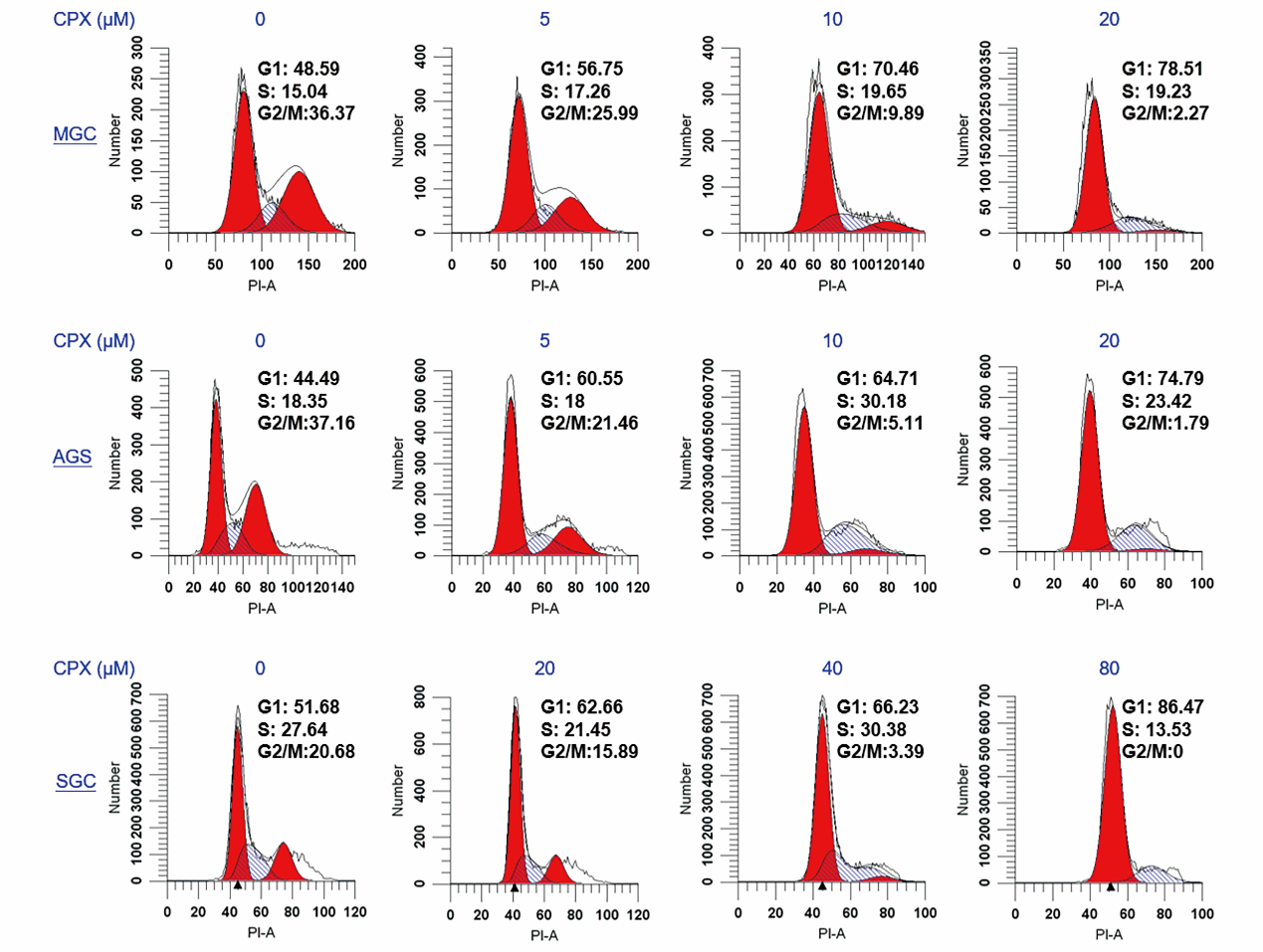
**

**Fig. S2 CPX induces G1/S arrest in GC cells.** Cell cycle distribution of MGC, AGS, and SGC cells treated with a serial dose of CPX for 24 h was analyzed using flow cytometry.

**
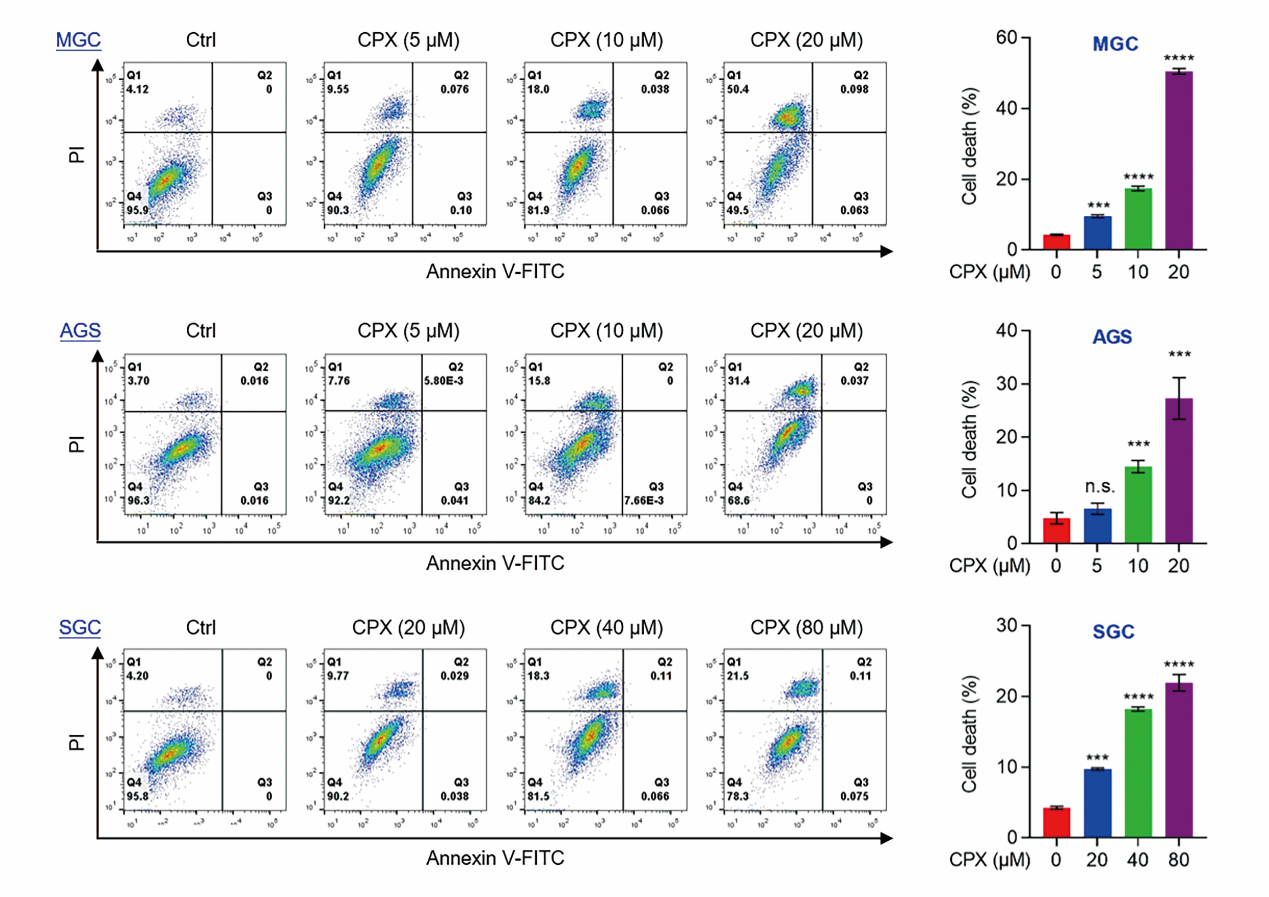
**

**Fig. S3 CPX does not induce apoptosis in GC cells.** Cells treated with a serial dose of CPX for 24 h and stained with annexin V-FITC/PI were determined using flow cytometry. The cell death rate was plotted followed by statistical analysis. Data were shown as mean ± SD (n=3, ****P* < 0.001, *****P* < 0.0001, or n.s., not significant by unpaired Student’s *t* test).


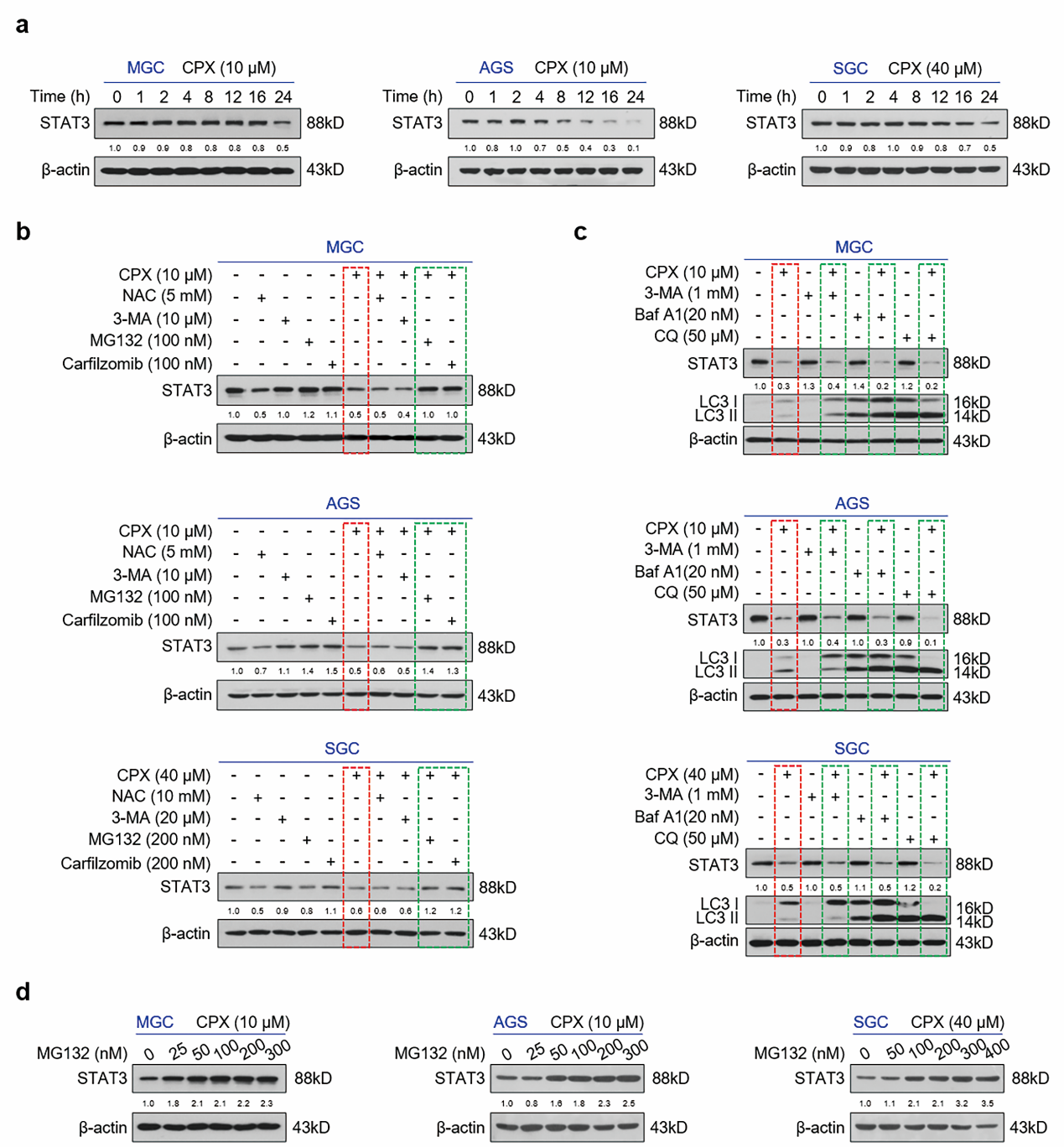


**Fig. S4 CPX degrades STAT3 through the ubiquitin-proteasome rather than the lysosome pathway. a** Western blotting analysis of STAT3 expression in GC cells treated with CPX at different times. **b** Western blotting analysis of STAT3 expression in GC cells co-treated with CPX and NAC, 3-MA, MG132, or Carfilzomib at the indicated concentration for 24 h. **c** Western blotting analysis of STAT3 expression in GC cells co-treated with CPX and three autophagy inhibitors 3-MA, Baf A1, or CQ for 24 h. **d** Western blotting analysis of STAT3 expression in GC cells co-treated with CPX and a serial dose of MG132 for 24 h.

**
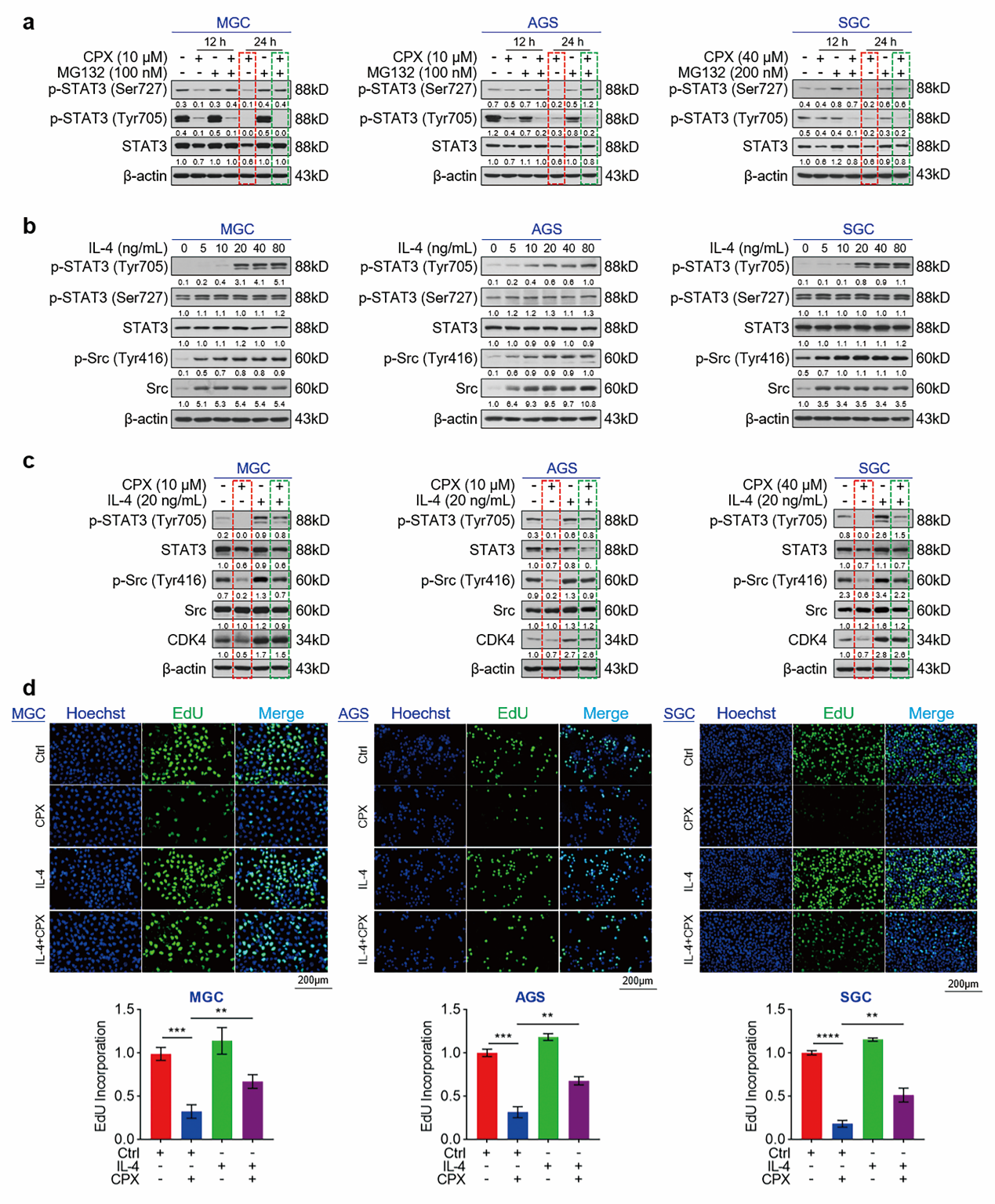
**

**Fig. S5** **CPX inhibits the p-Src (Tyr416)/p-STAT3 (Tyr705) pathway to hinder the proliferation of GC cells. a** Western blotting analysis of STAT3, p-STAT3 (Ser727), and p-STAT3 (Tyr705) in GC cells co-treated with CPX and MG132 for 12 or 24 h. **b** Western blotting analysis of p-STAT3 (Tyr705), p-STAT3 (Ser727), STAT3, p-Src (Tyr416), and Src in GC cells treated with various concentrations of IL-4 for 24 h. **c,d** The protein levels of p-STAT3 (Tyr705), STAT3, p-Src (Tyr416), Src, and CDK4 (**c**) and proliferation of GC cells (**d**) co-treated with CPX and IL-4 for 24 h. EdU incorporation was quantified using ImageJ Plus software (Scale bar, 200 µm). Data were shown as mean ± SD (n=3, ** *P* <0.01, ****P* < 0.001, *****P* < 0.0001).

**
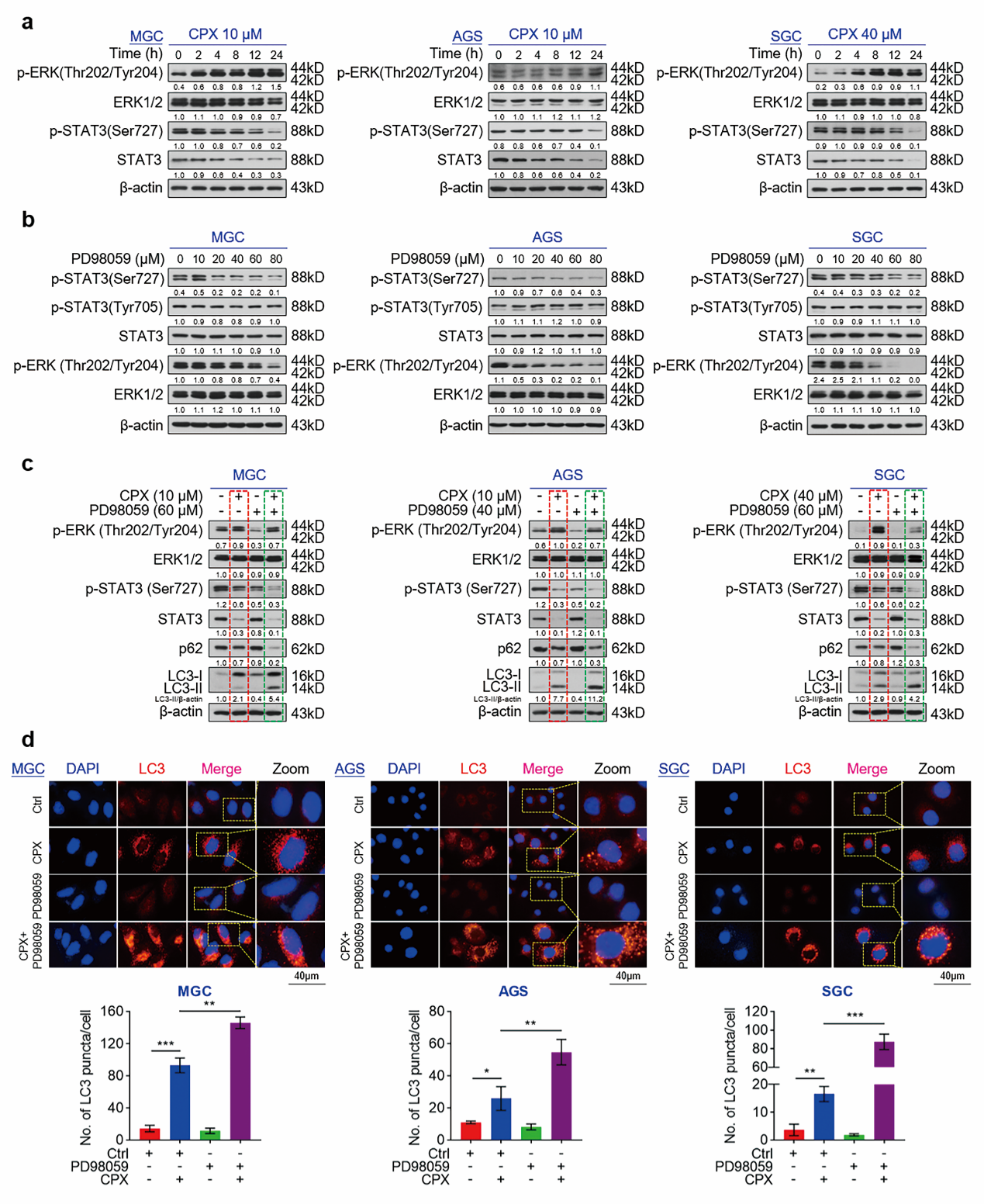
**

**Fig. S6 Inhibition of p-STAT3 (Ser727) augments the effects of CPX on the autophagy of GC cells.** **a** Western blotting analysis of p-ERK (Thr202/Tyr204), ERK1/2, p-STAT3 (Ser727), and STAT3 in GC cells treated with CPX at different times. **b** Western blotting analysis of p-STAT3 (Ser727), p-STAT3 (Tyr705), STAT3, p-ERK (Thr202/Tyr204), and ERK1/2 in GC cells treated with a serial concentration of PD98059 for 24 h. **c,d** The protein levels of p-ERK (Thr202/Tyr204), ERK1/2, p-STAT3 (Ser727), STAT3, p62, and LC3 (**c**) and the endogenous LC3 puncta fluorescence (**d**) in GC cells treated with CPX and PD98059 for 24 h. The ratio of LC puncta to cell number was quantified using ImageJ Plus software (Scale bar, 40 µm). Data were shown as mean ± SD (n=3, **P* < 0.05, ***P* < 0.01, *** *P* < 0.001).

**
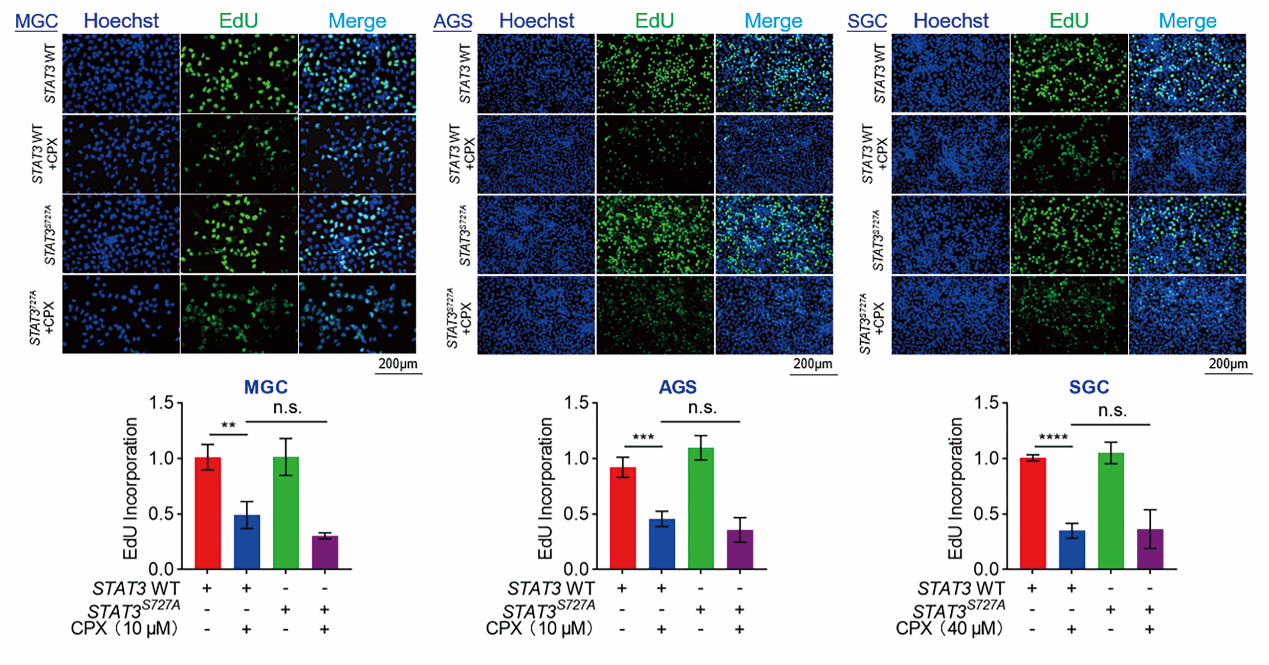
**

**Fig. S7 CPX failed to inhibit the proliferation of *STAT3^S727A^* GC cells.** EdU cell proliferation kit analysis of *STAT3^S727A^* GC cell proliferation treated with CPX for 24 h. EdU incorporation was quantified using ImageJ Plus software (Scale bar, 200 µm). Data were shown as mean ± SD (n=3, ***P*<0.01, ****P* < 0.001, *****P* < 0.0001, or n.s., not significant by unpaired Student’s *t* test).

**Supplementary Table**

**Table S1 Primers for gene amplification in the process of RT-qPCR.**

| **Primer name** | **The sequence of primers from 5’ to 3’** |
| --- | --- |
| Human-*STAT3* | Forward: CGGAGAAGCATCGTGAGTGAGC  Reverse: GTTGCCGCCTCTTCCAGTCAG |
| Human-*β-ACTIN* | \| Forward: AGCACAGAGCCTCGCCTTTG \| \| --- \| \| Reverse: AAGCCGGCCTTGCACATG \| |
