# Supplementary material for: Ciclopirox drives growth arrest and autophagic cell death through STAT3 in gastric cancer cells
Source: Cell Death Dis. 2022 Nov 28;13(11):1007. doi: 10.1038/s41419-022-05456-7 (PMC9705325; doi:10.1038/s41419-022-05456-7)
Supplement: Supplementary file 2 — Original Data File [file 41419_2022_5456_MOESM2_ESM.pdf]

Figure. 1F

MGC cells

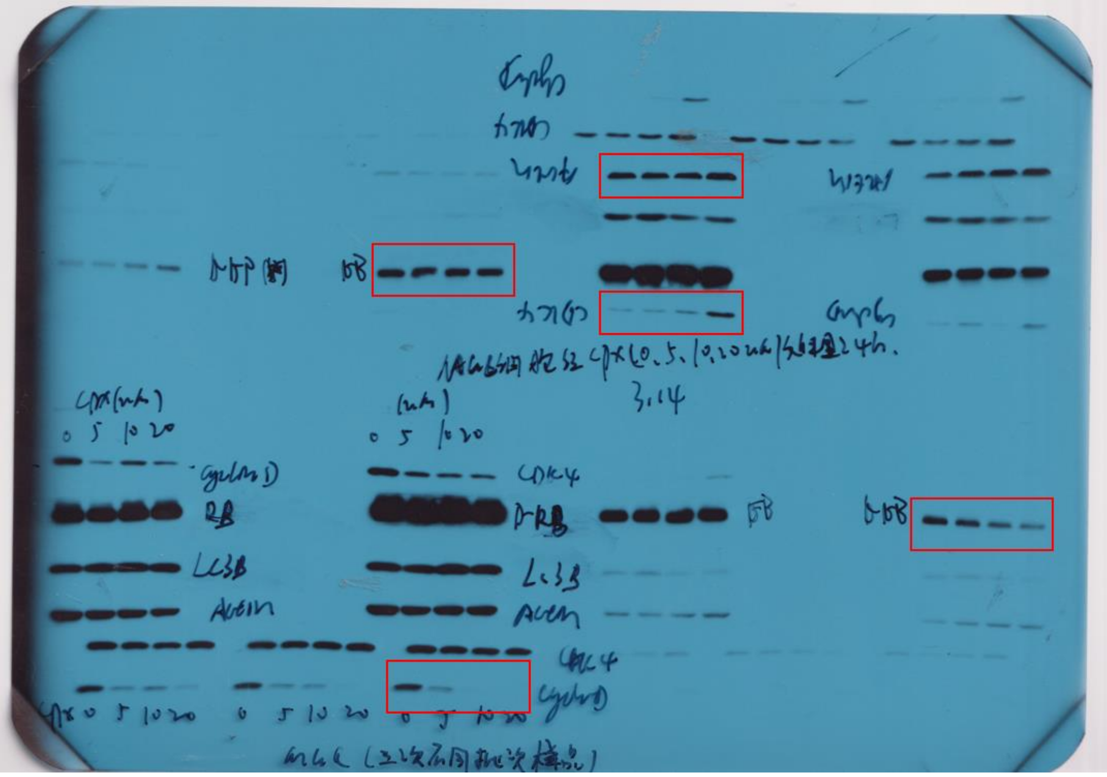

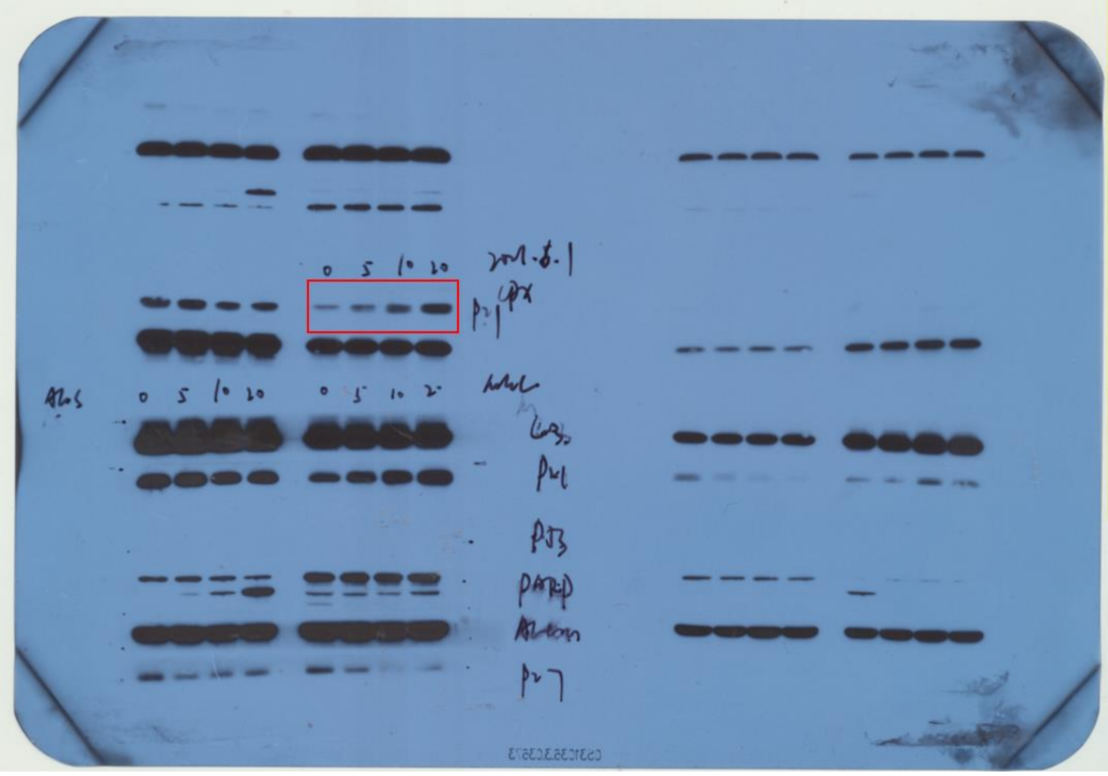

AGS cells

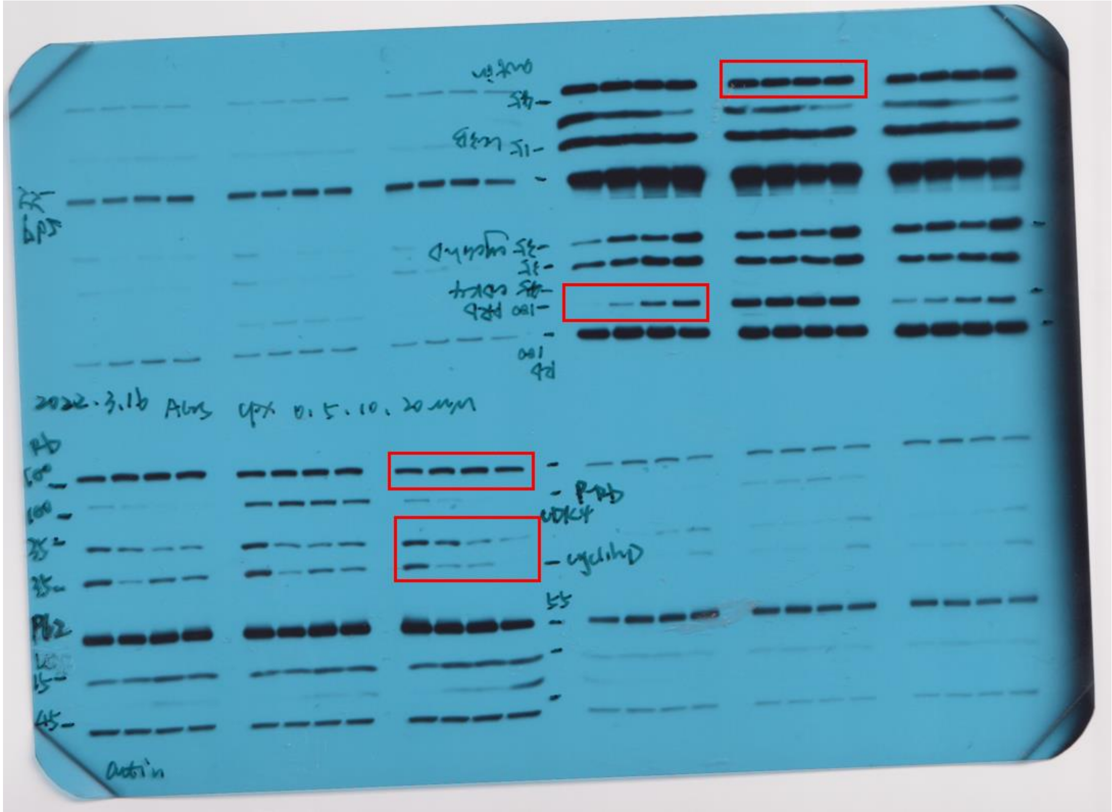

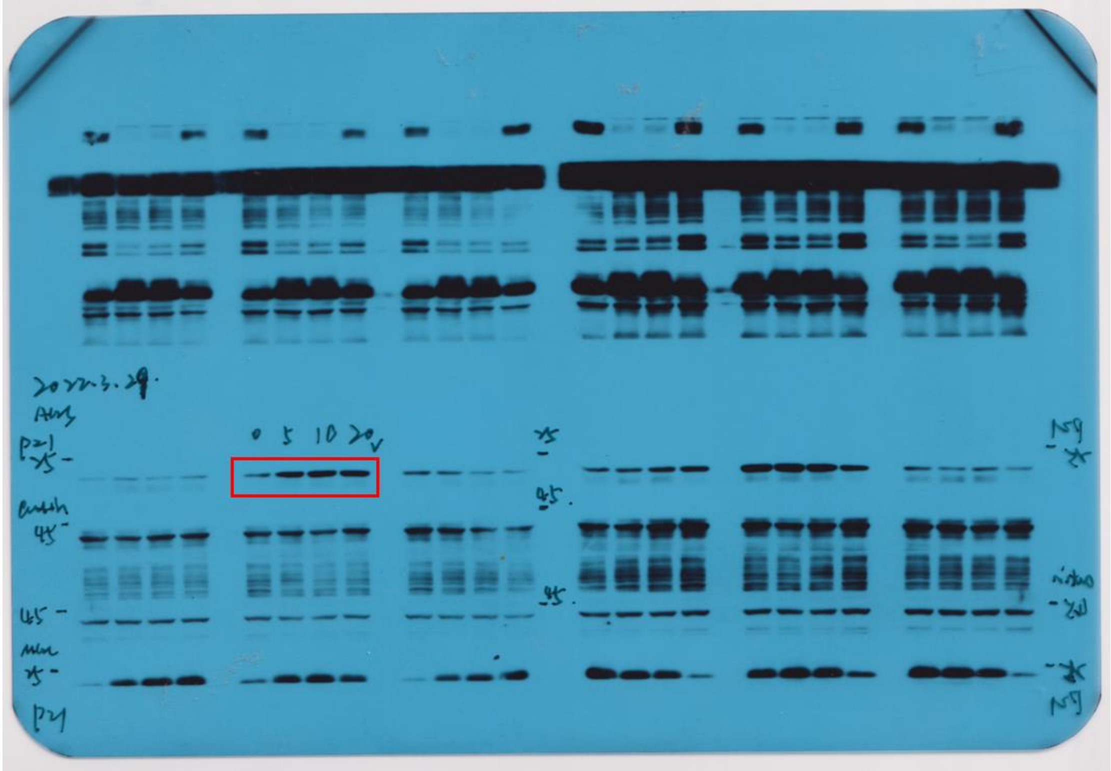

SGC cells

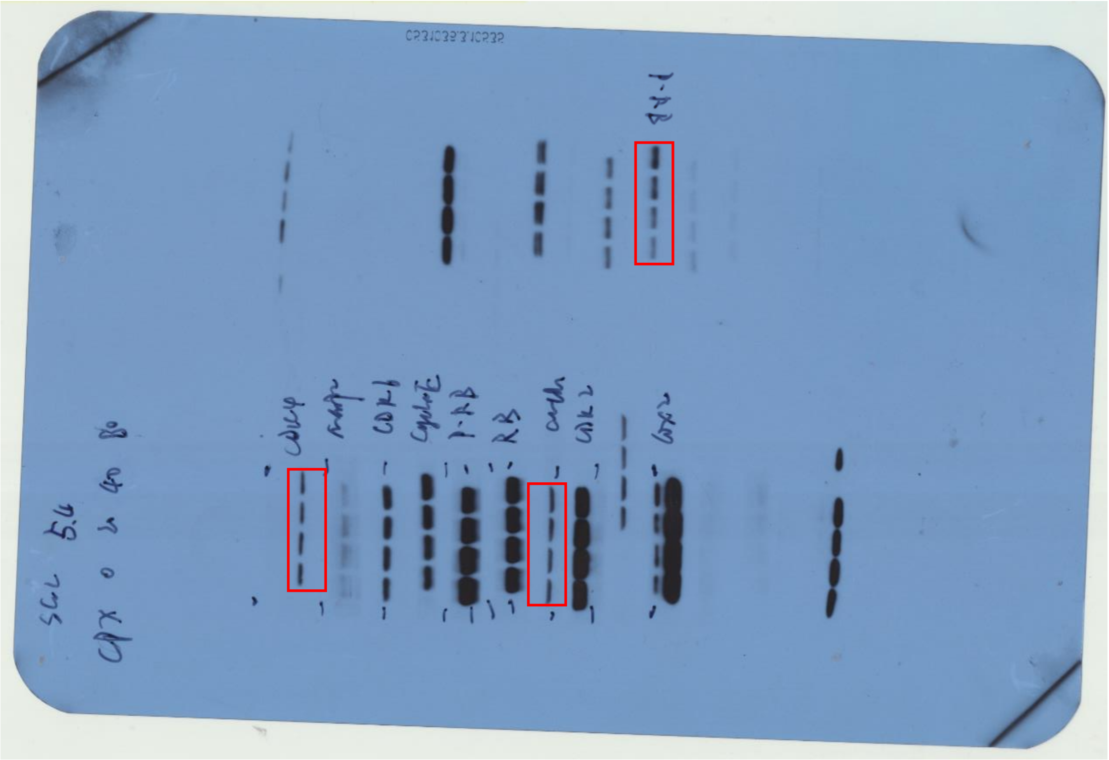

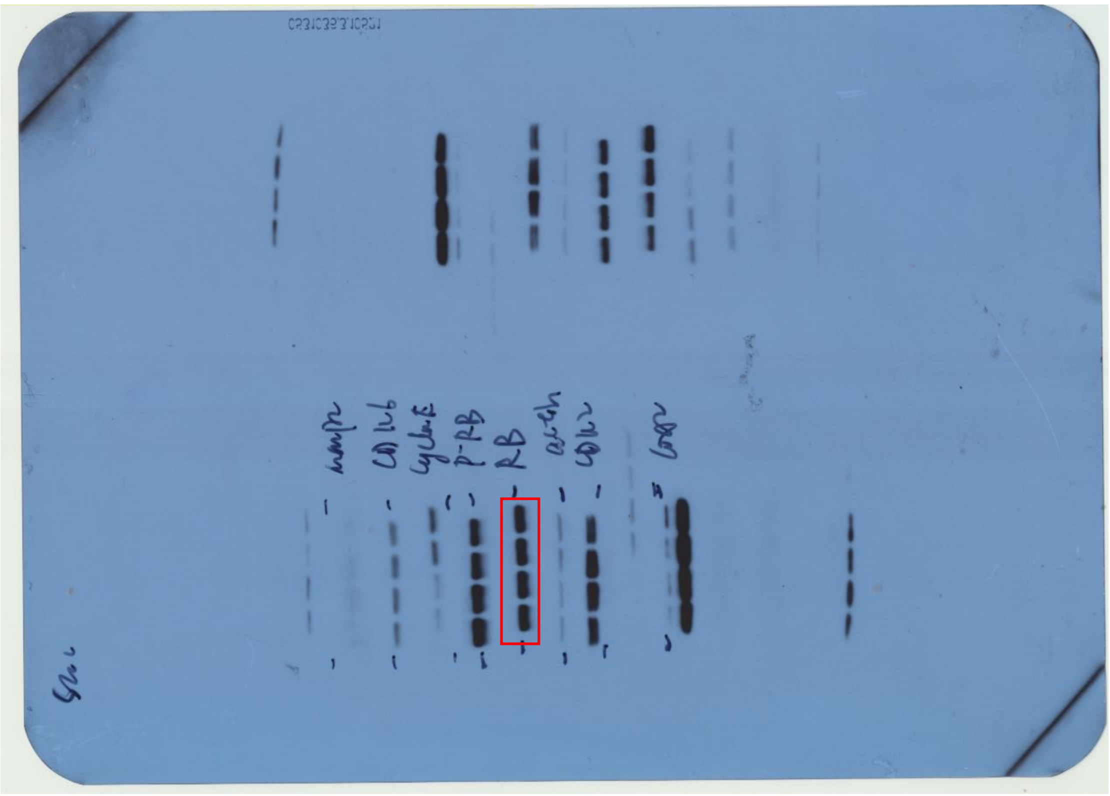

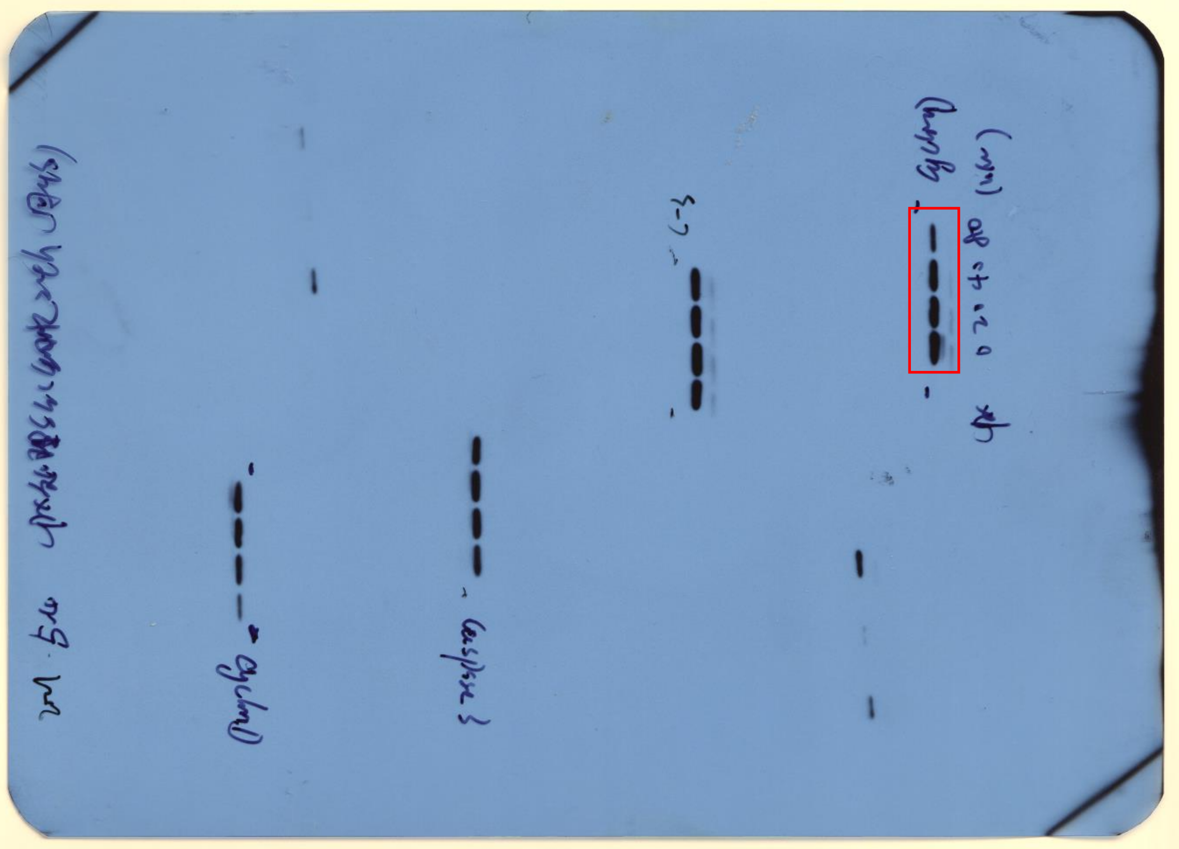

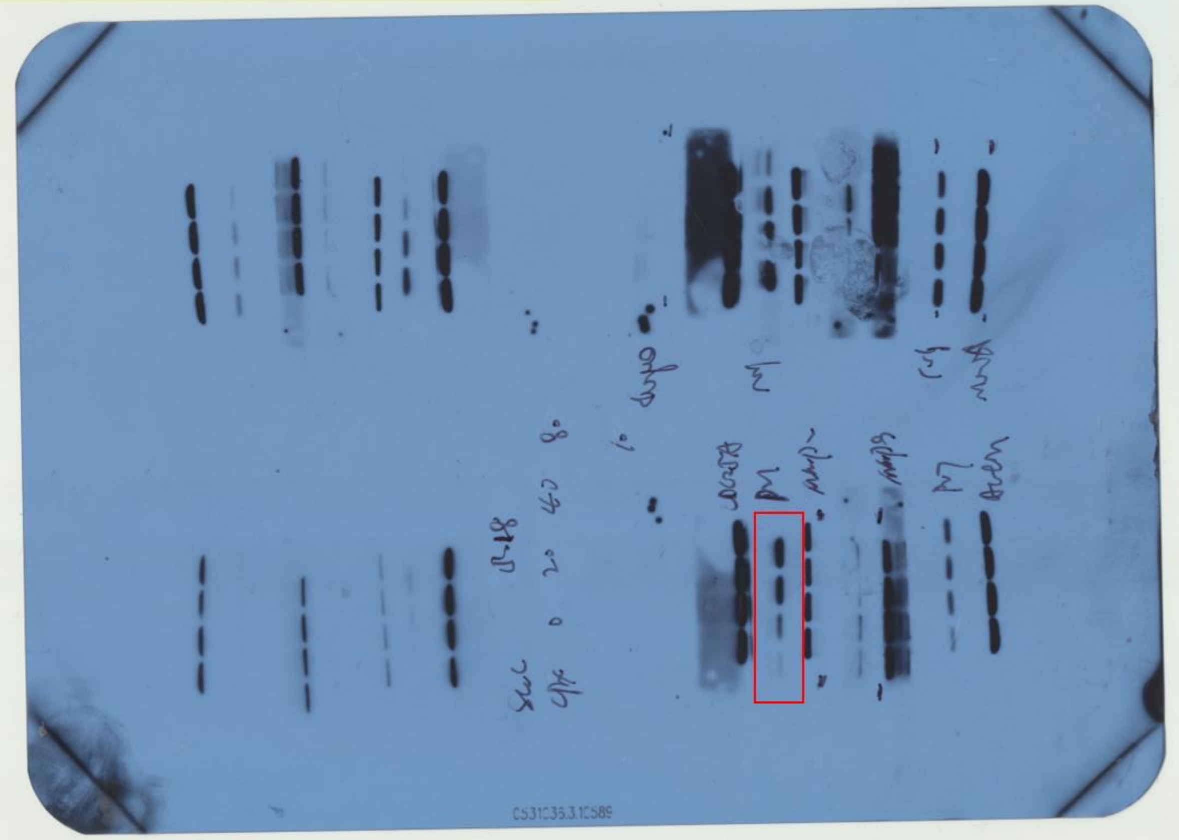

Figure. 2E

MGC cells

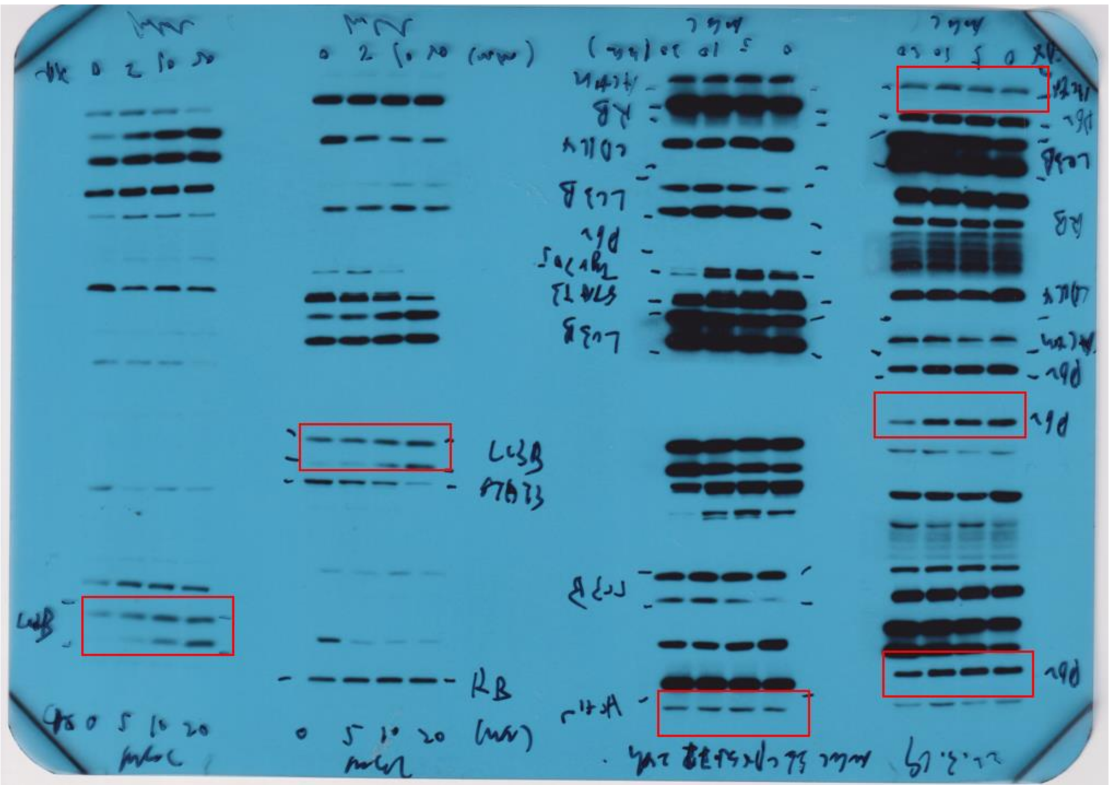

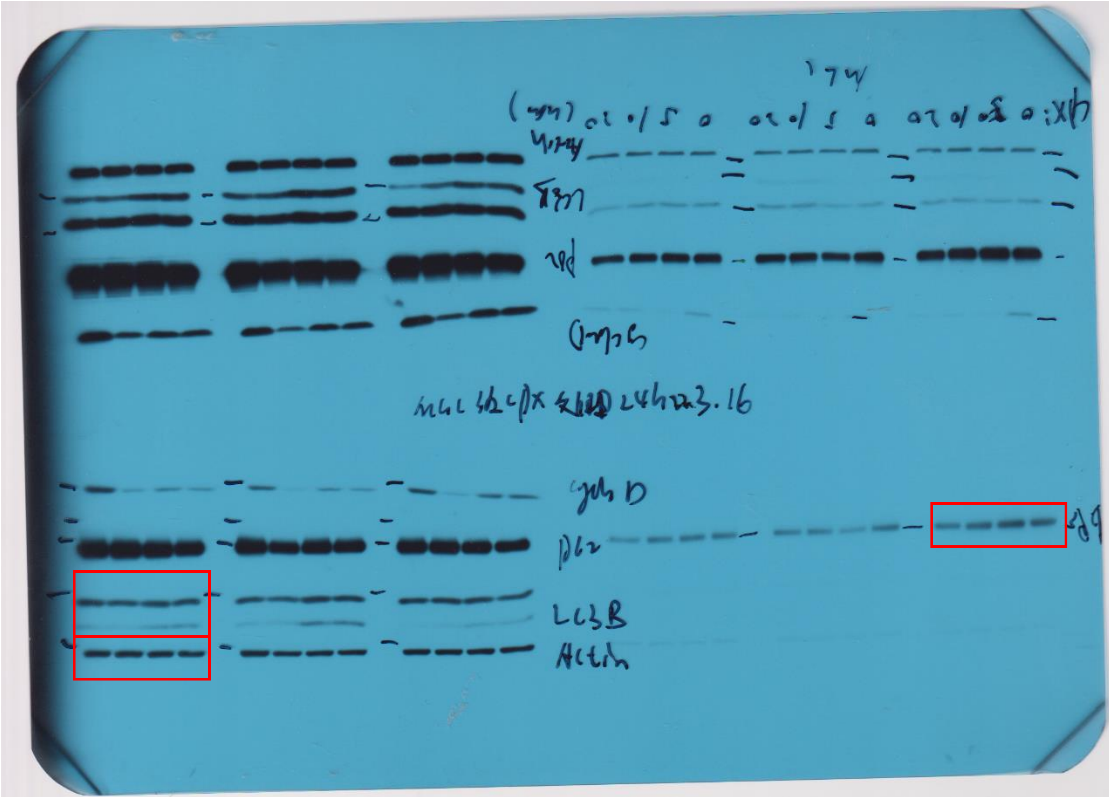

AGS cells

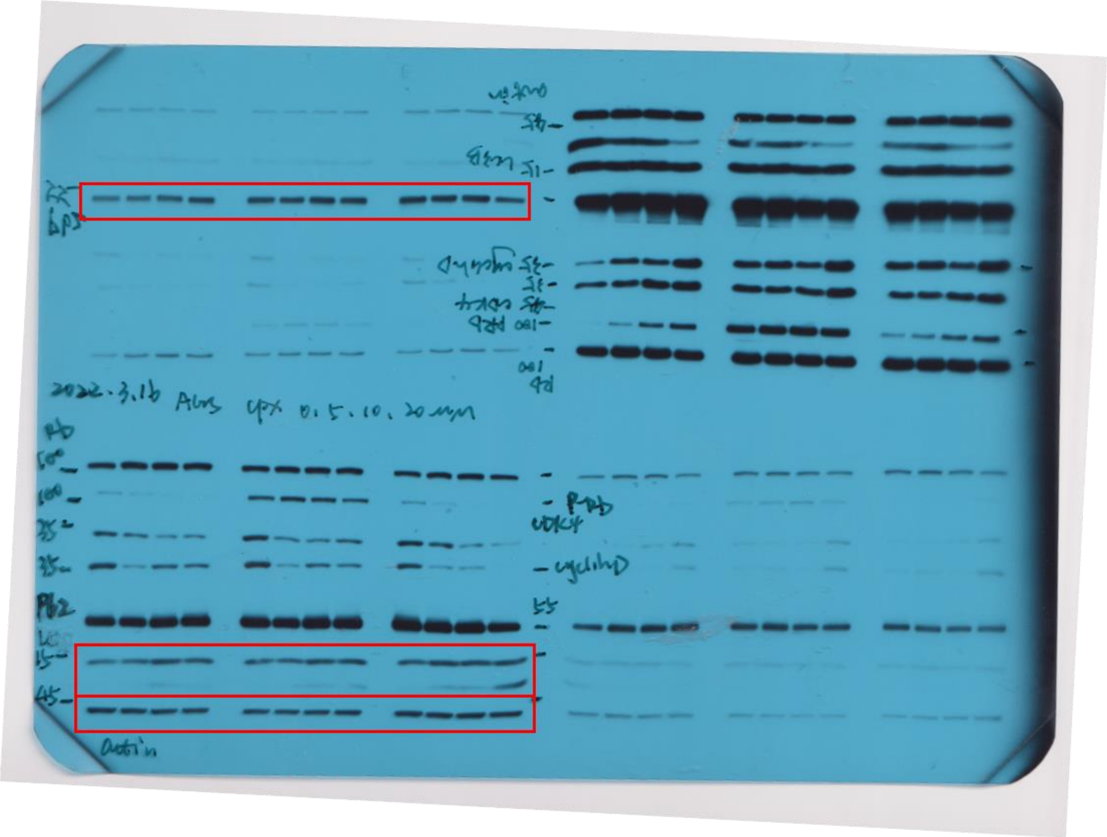

SGC cells

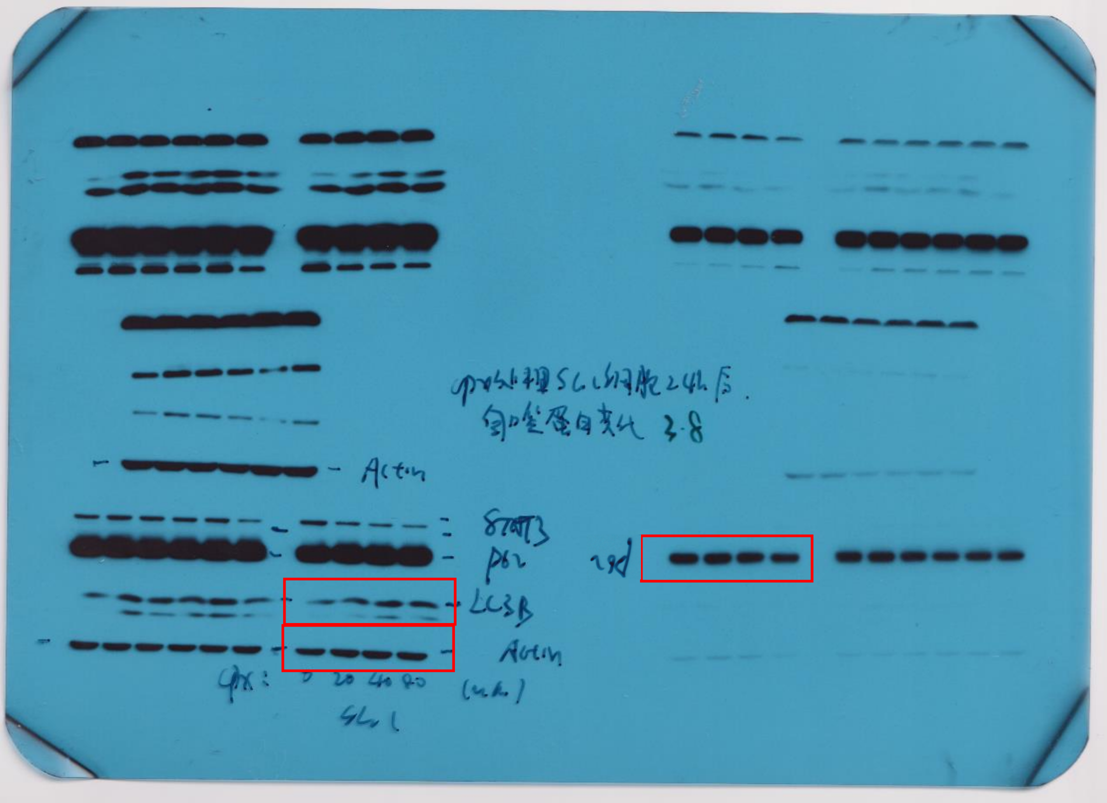

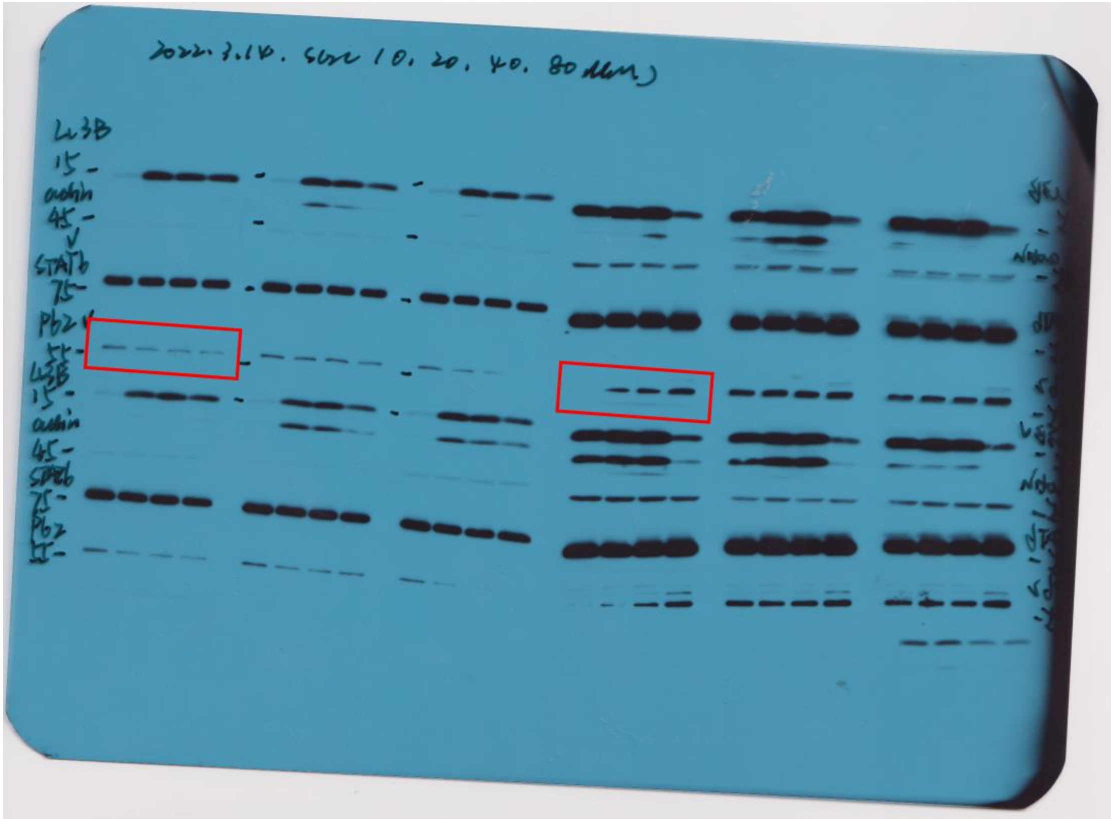

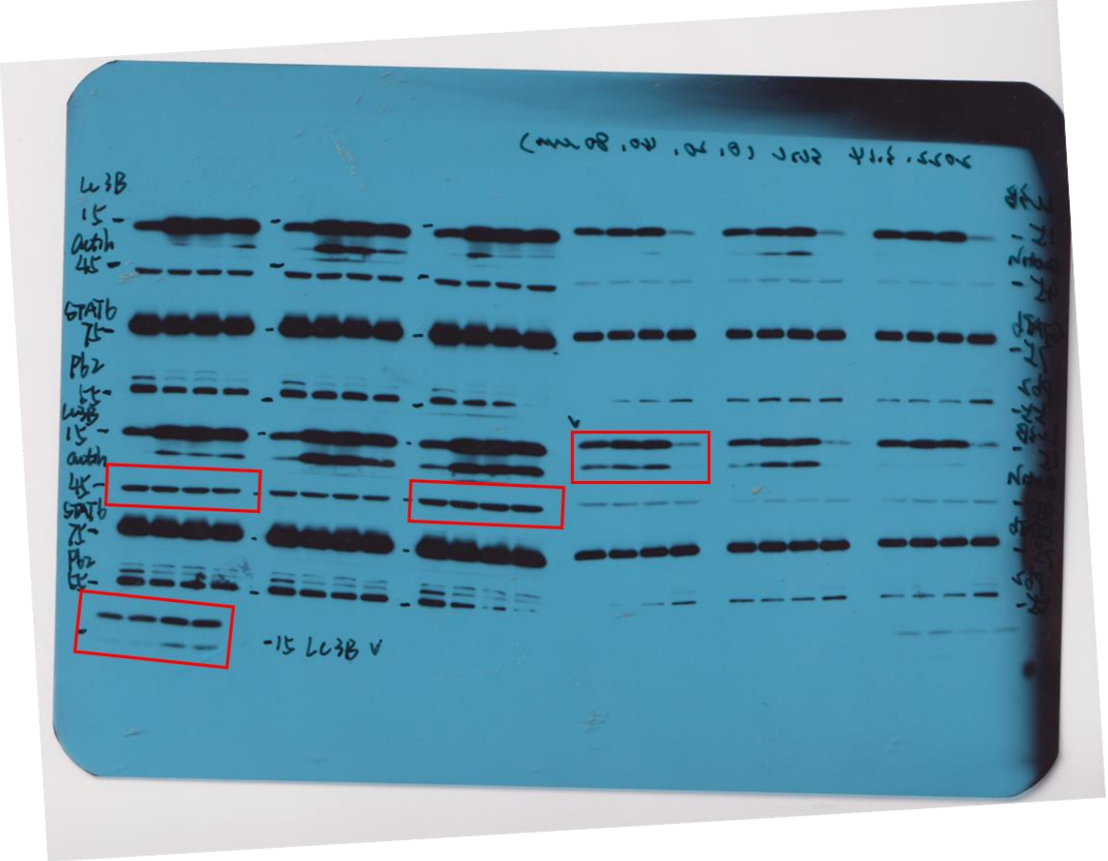

Lc3B
15
actin
45
STAT6
75
-15 Lc3B v

Figure. 3A

MGC, AGS, and SGC cells

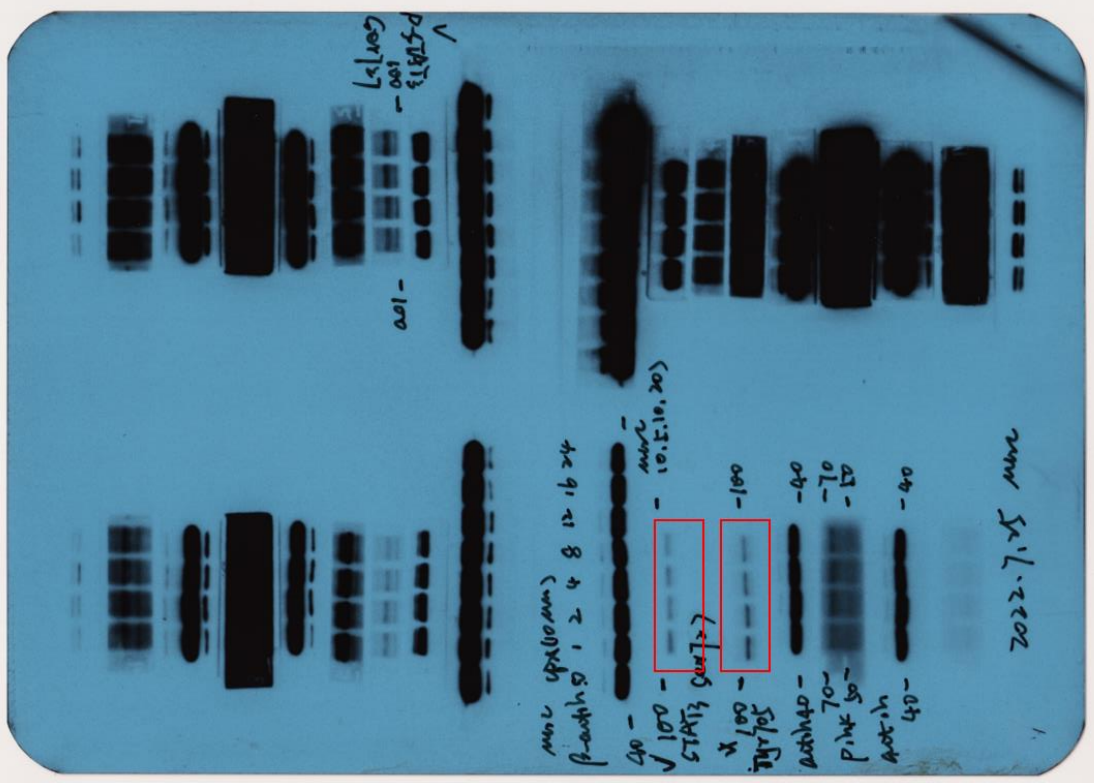

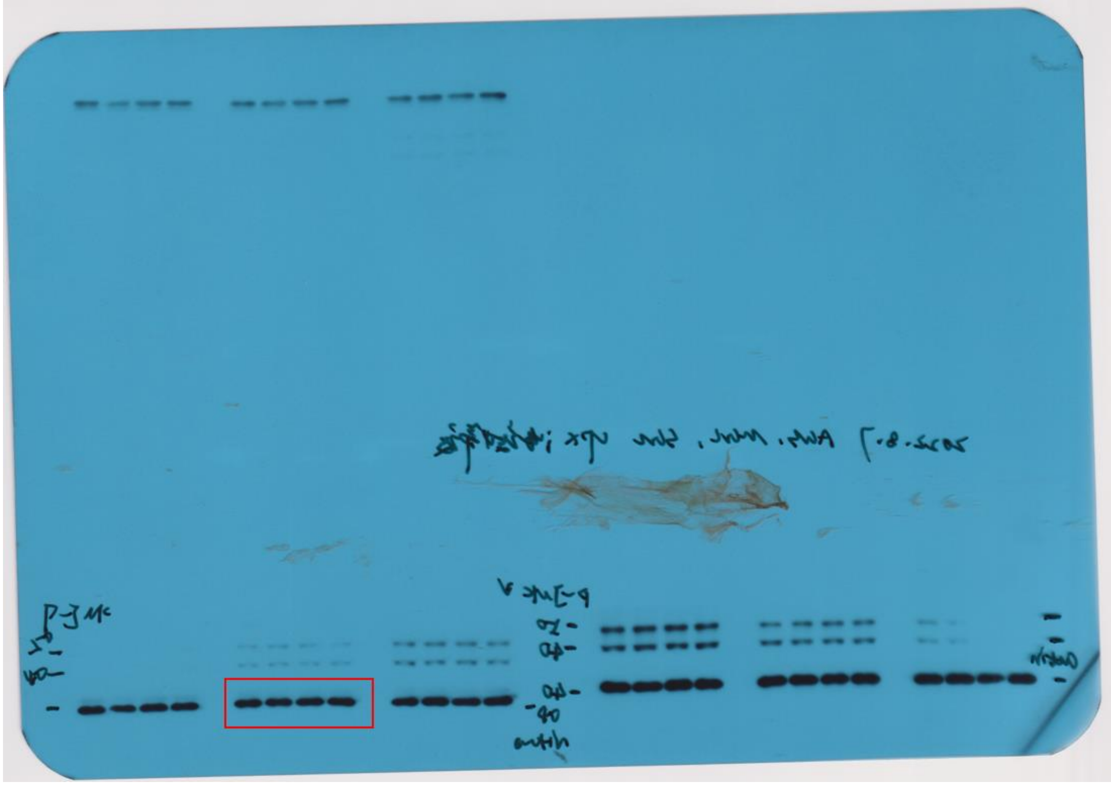

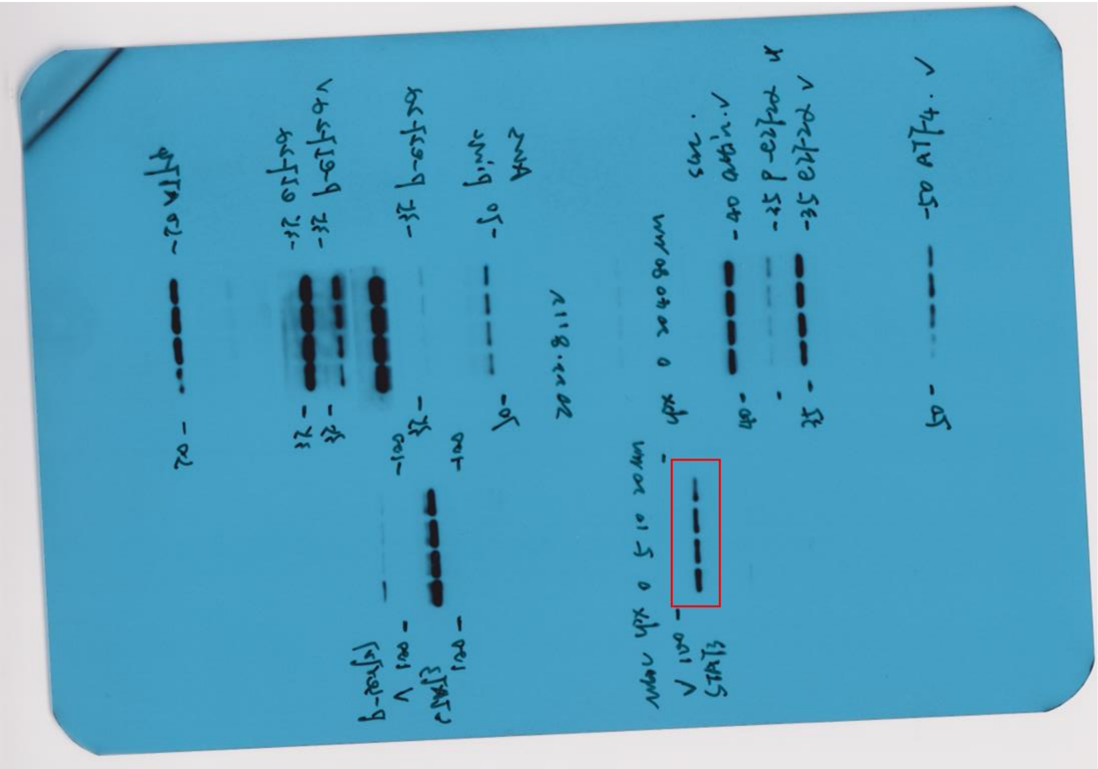

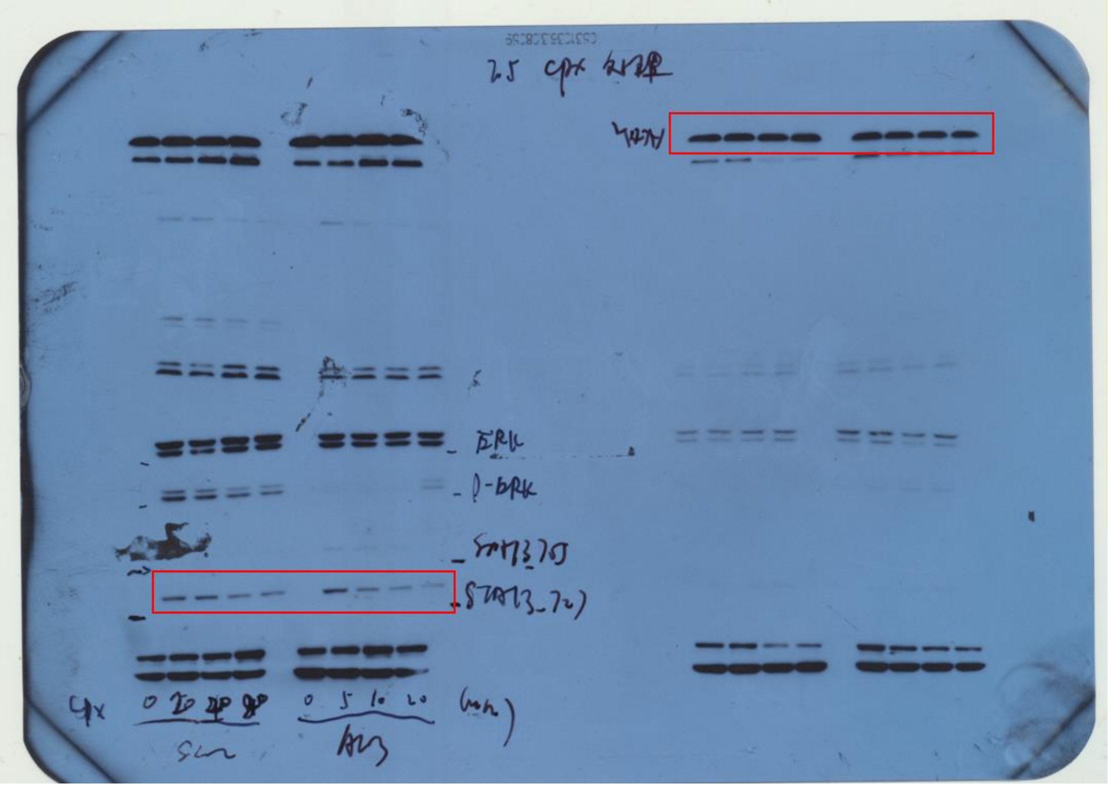
ERK
p-ERK

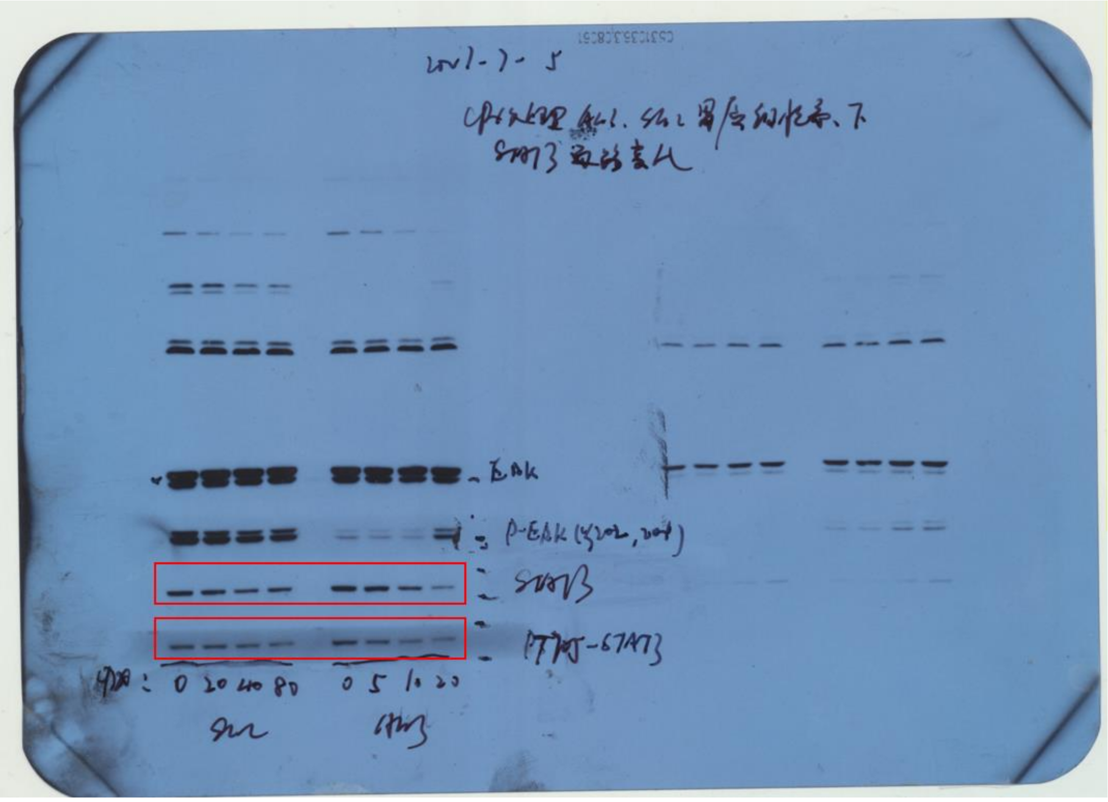
0 20 40 80
0 5 10 20

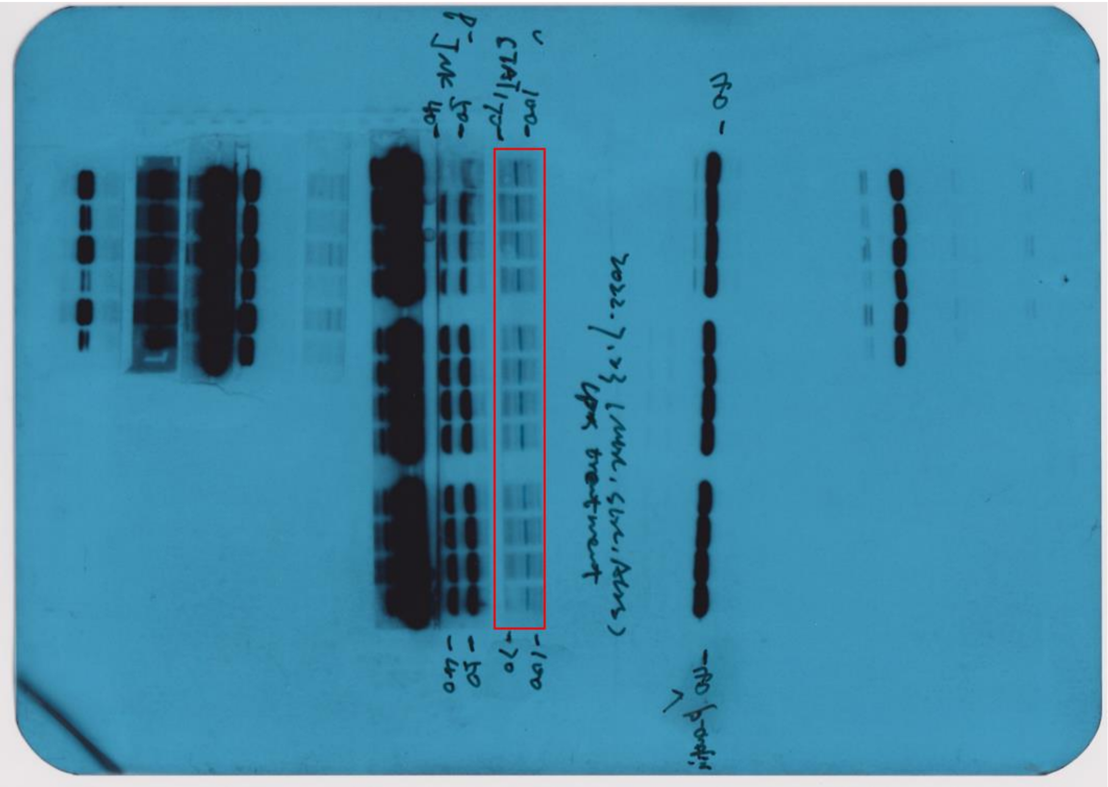

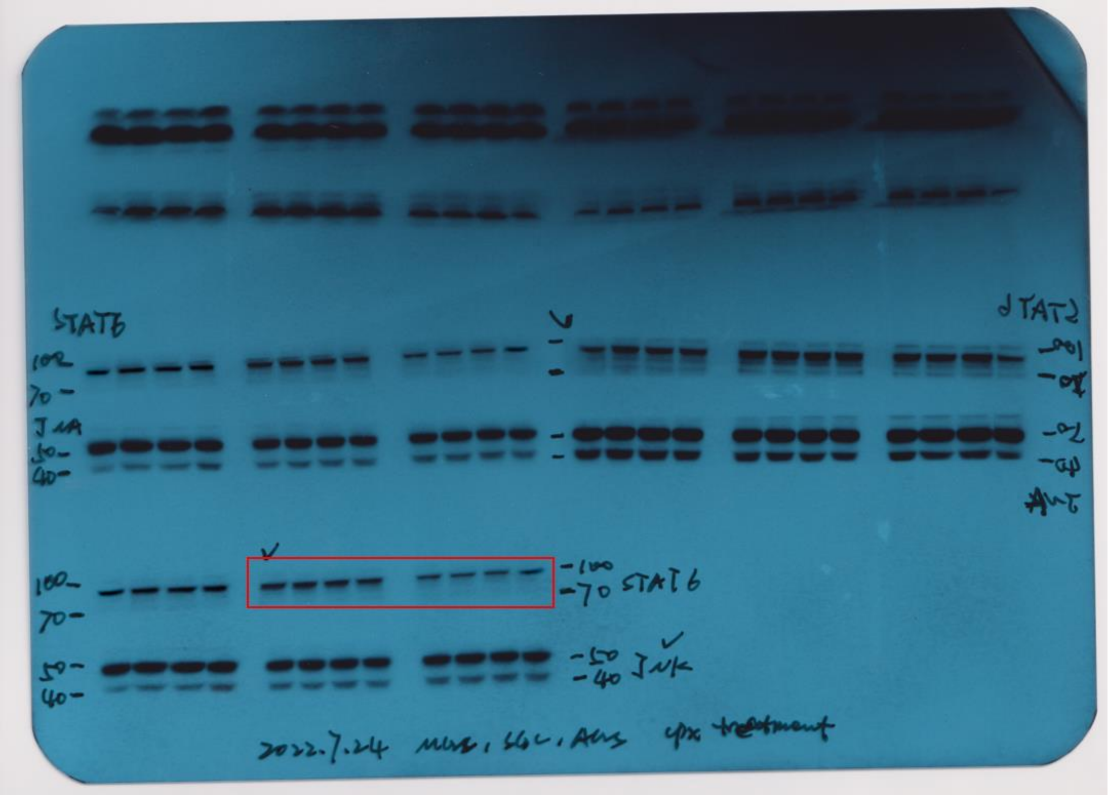
STAT6
-100
-70 STAT6
-50
-40 JNK
2022.7.24
cpx treatment

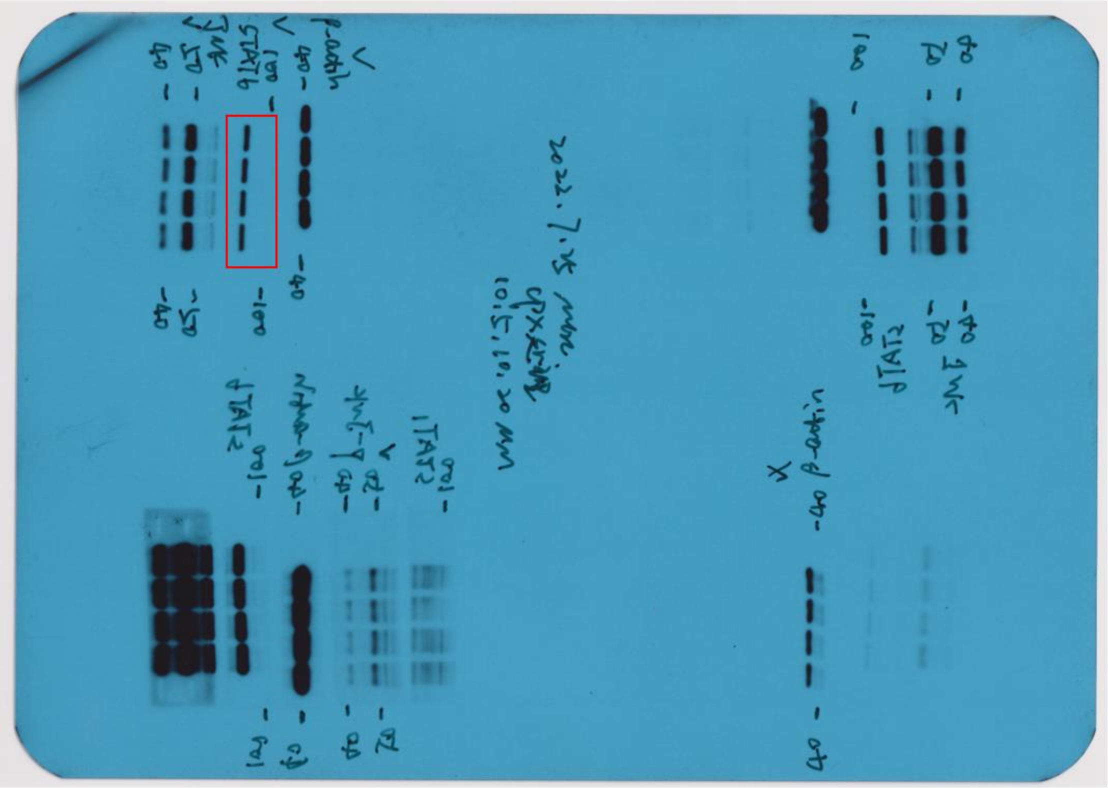

Figure. 3C

MGC cells

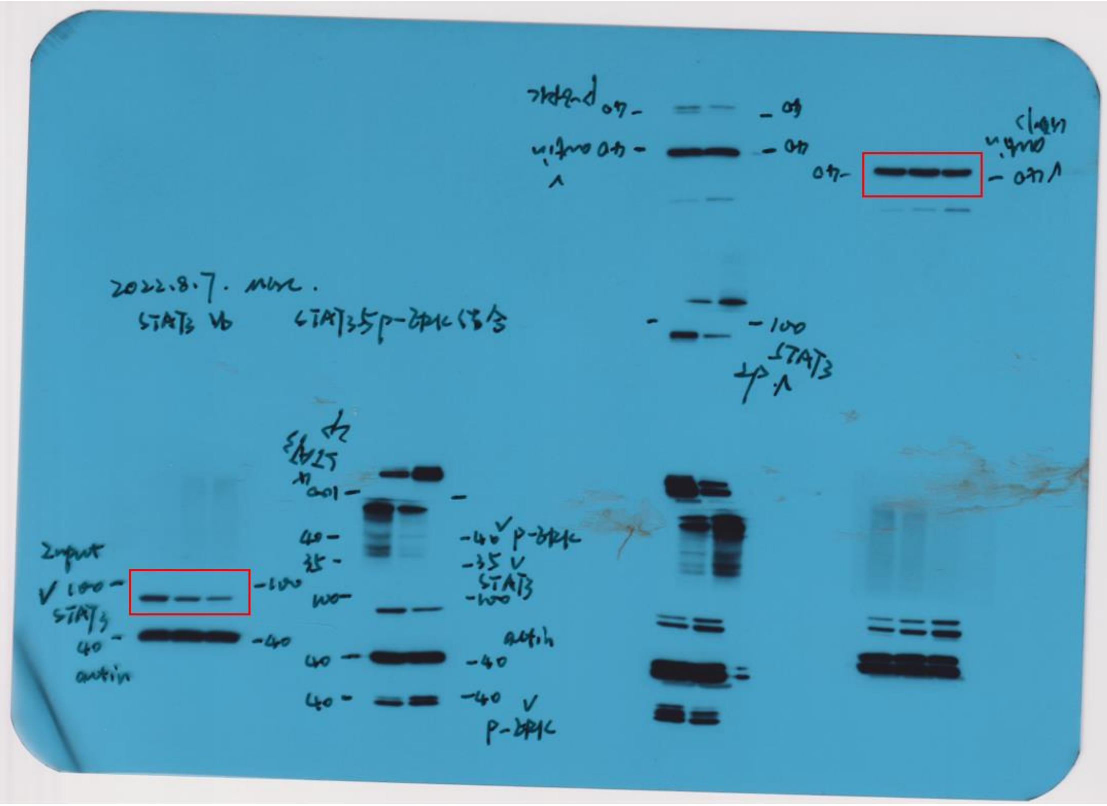

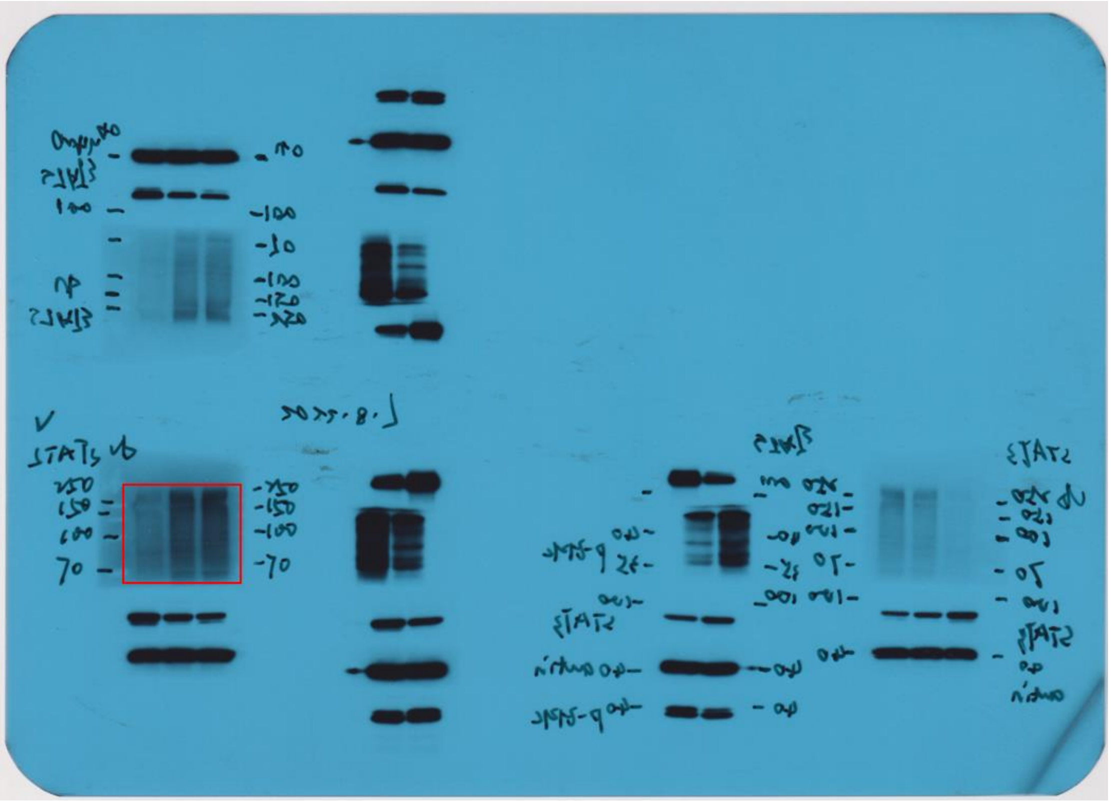

Figure. 3C

AGS cells

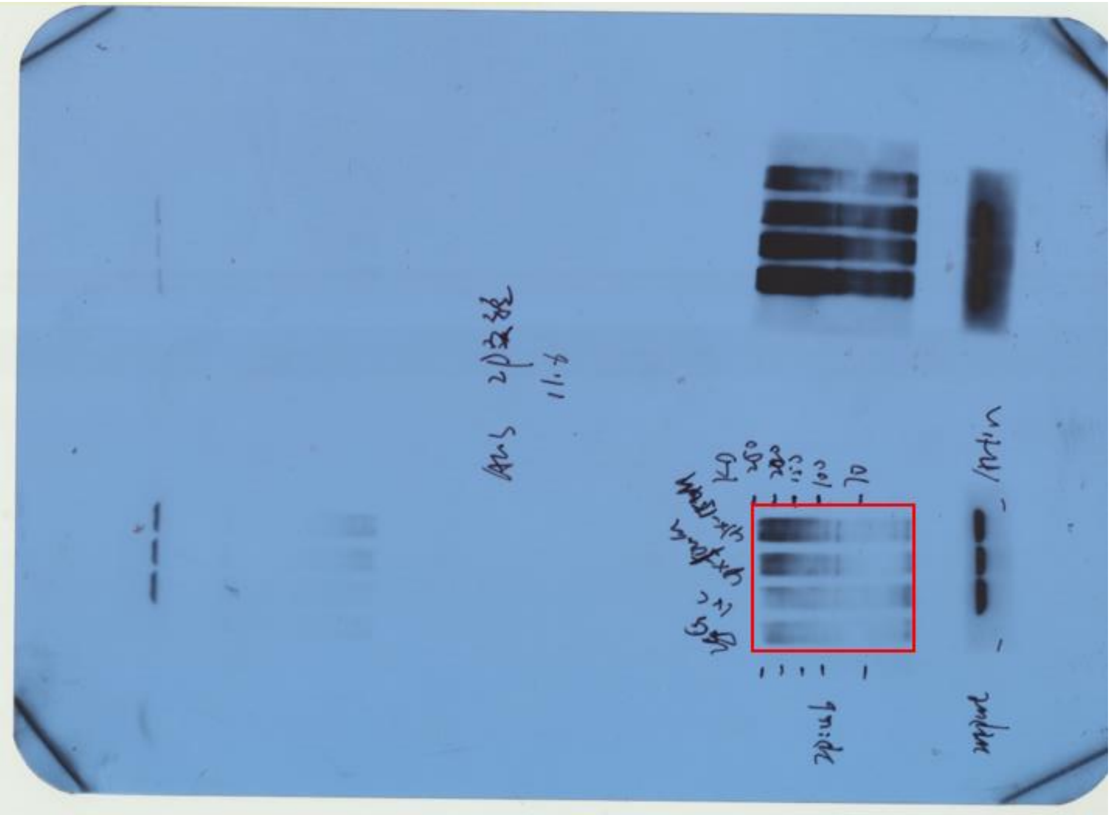

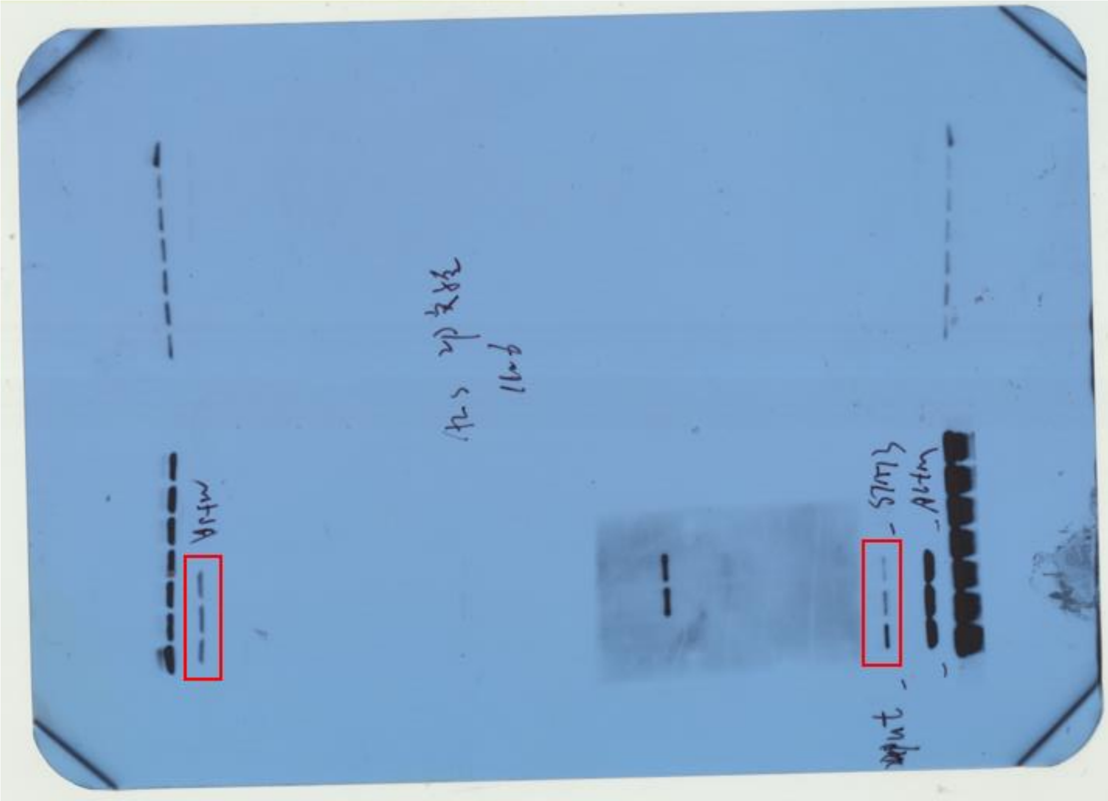

Figure. 3C

SGC cells

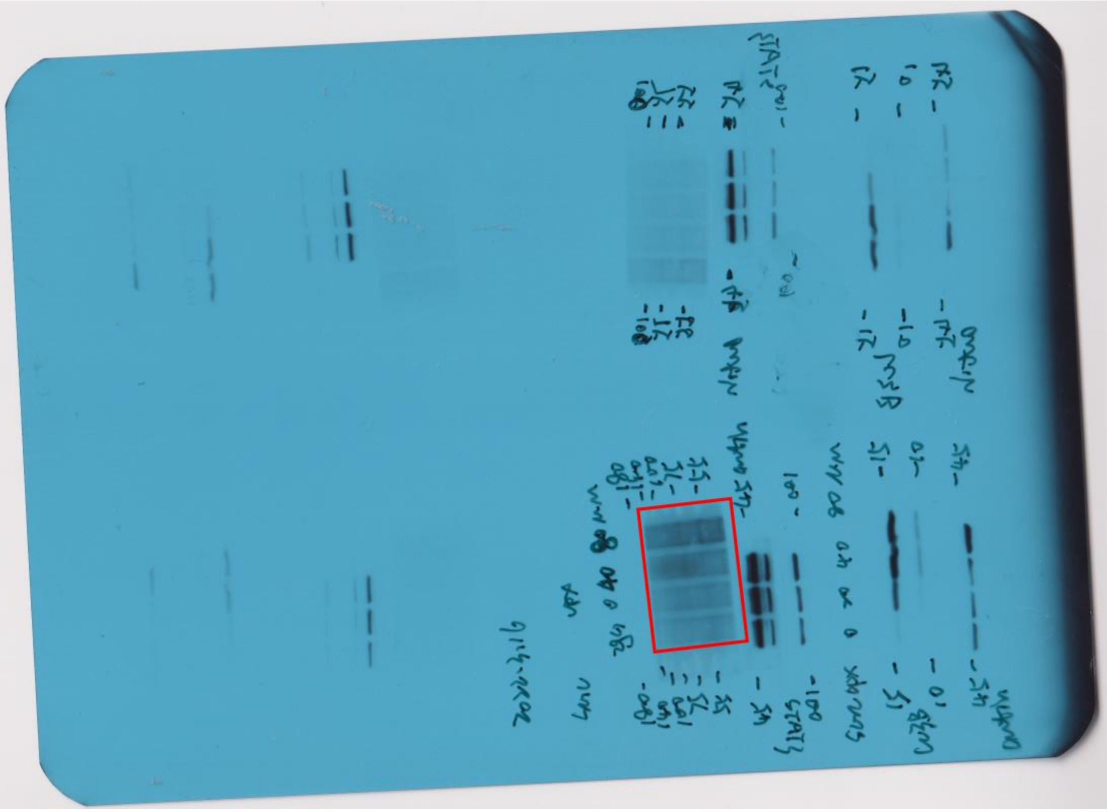

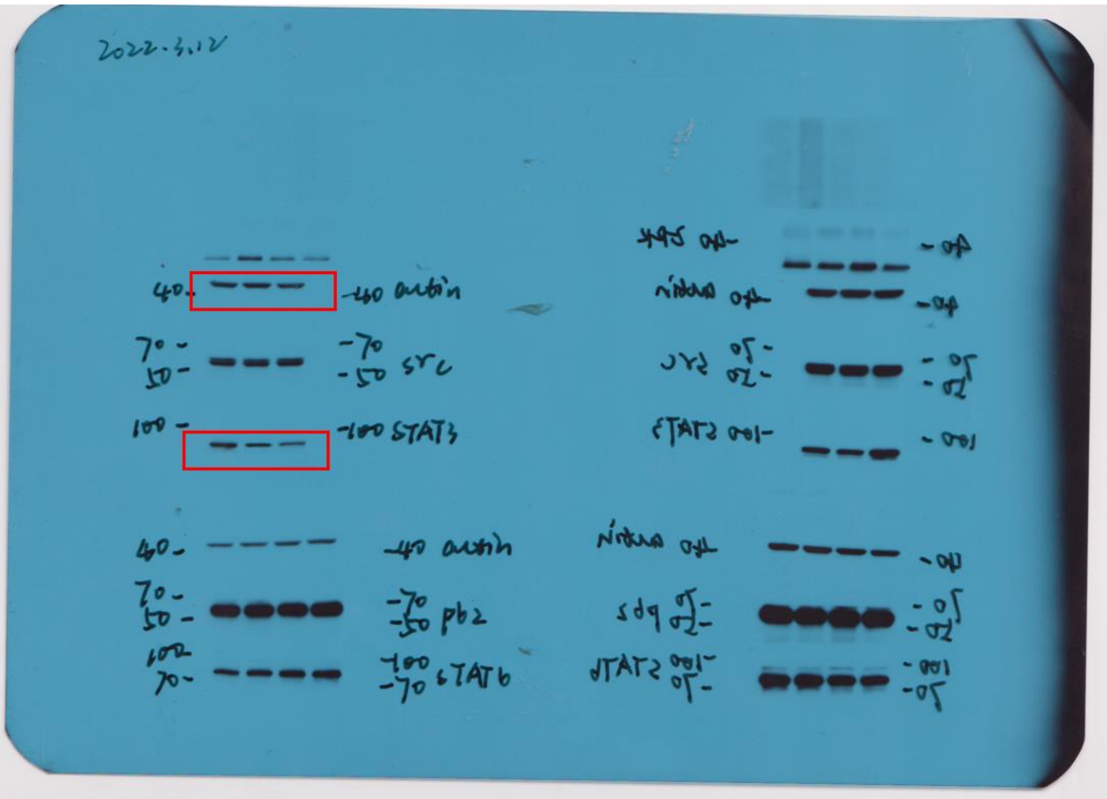

Figure. 3D

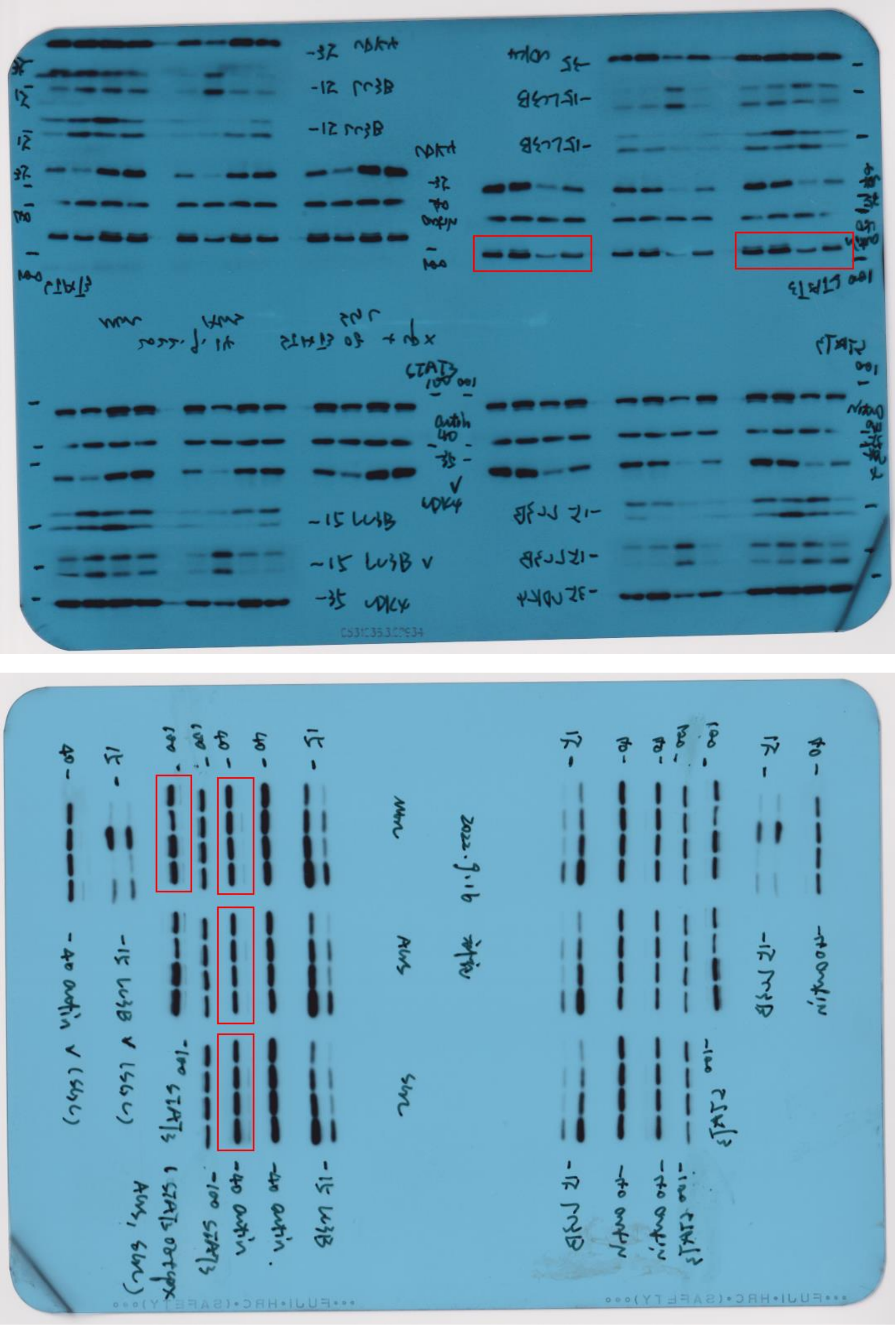

Figure. 4A

MGC cells

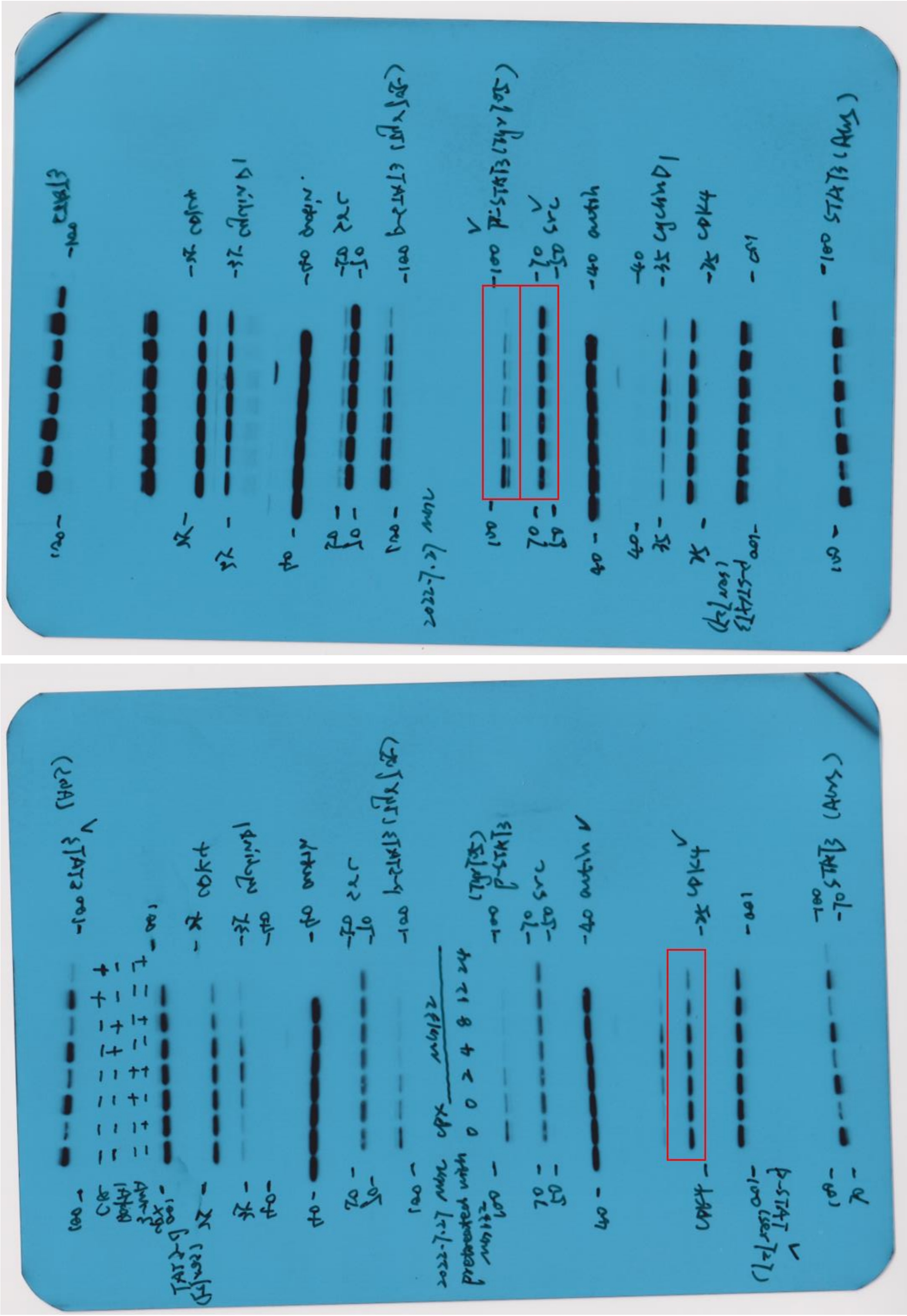

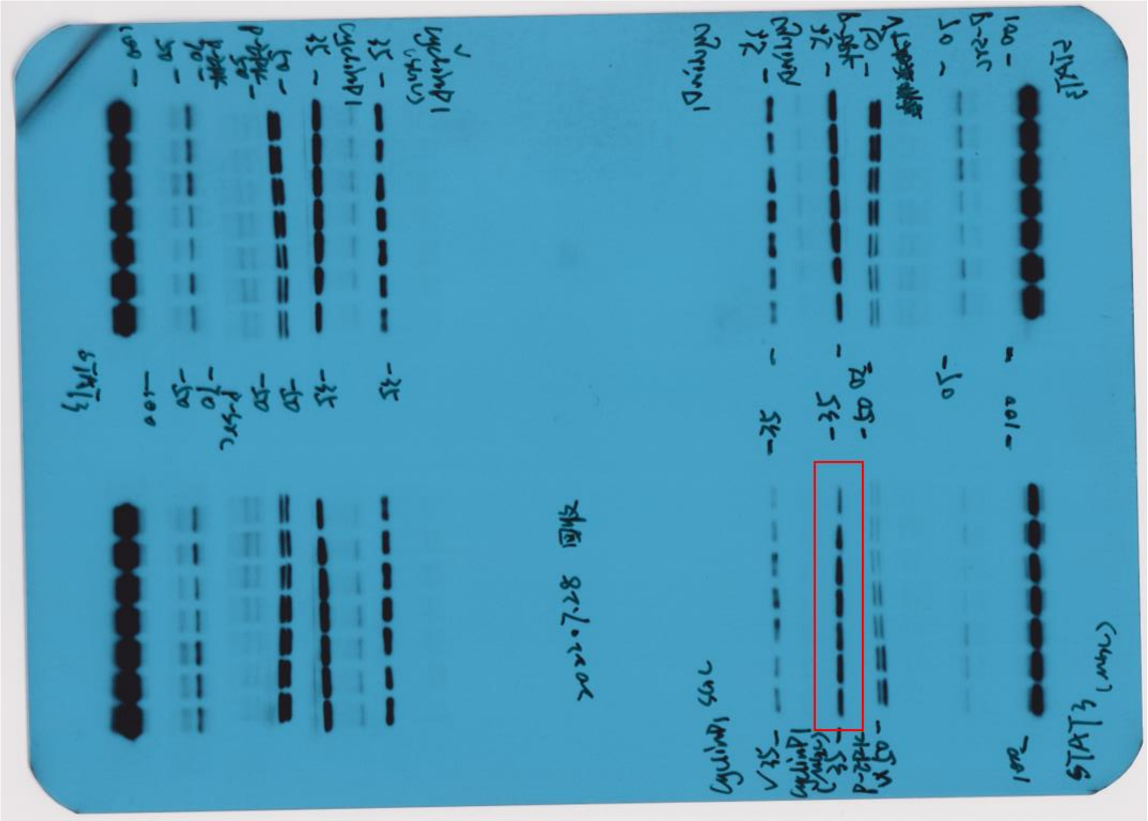

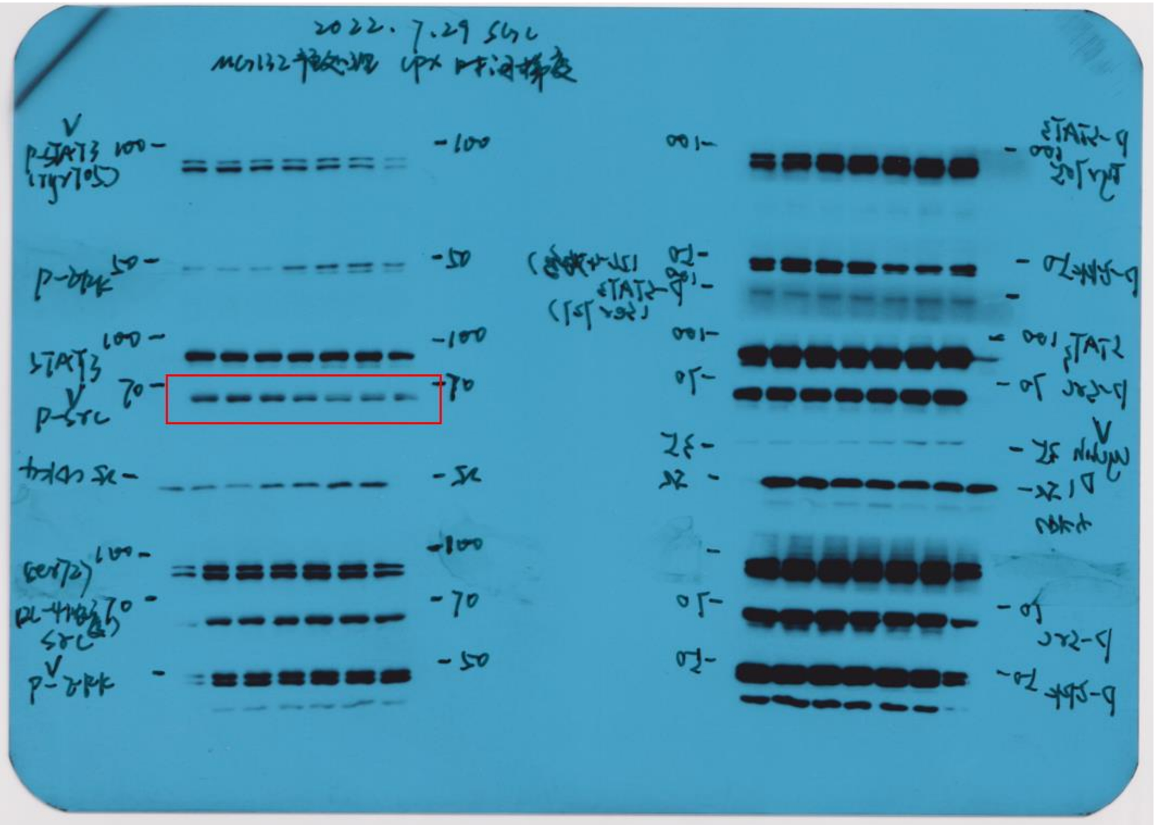
2022.7.29 SGC
p-STAT3
p-ERK
STAT3
p-src
p-ERK

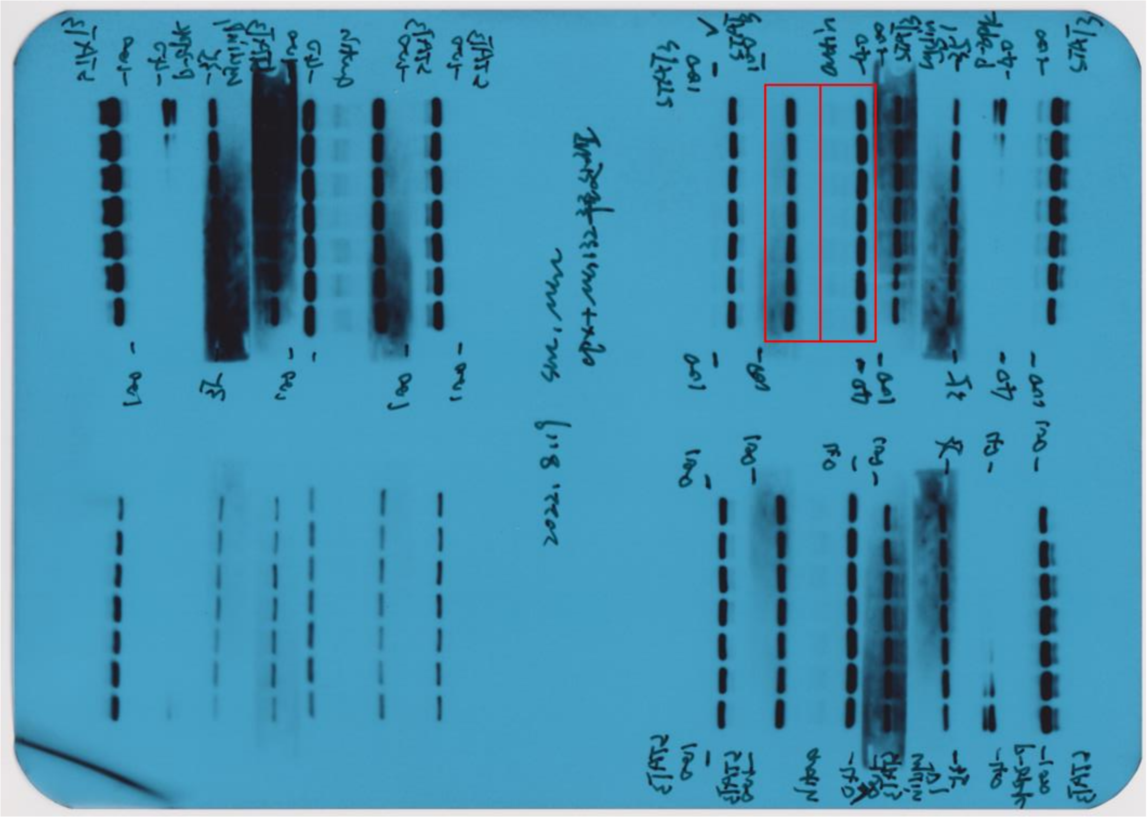

AGS cells

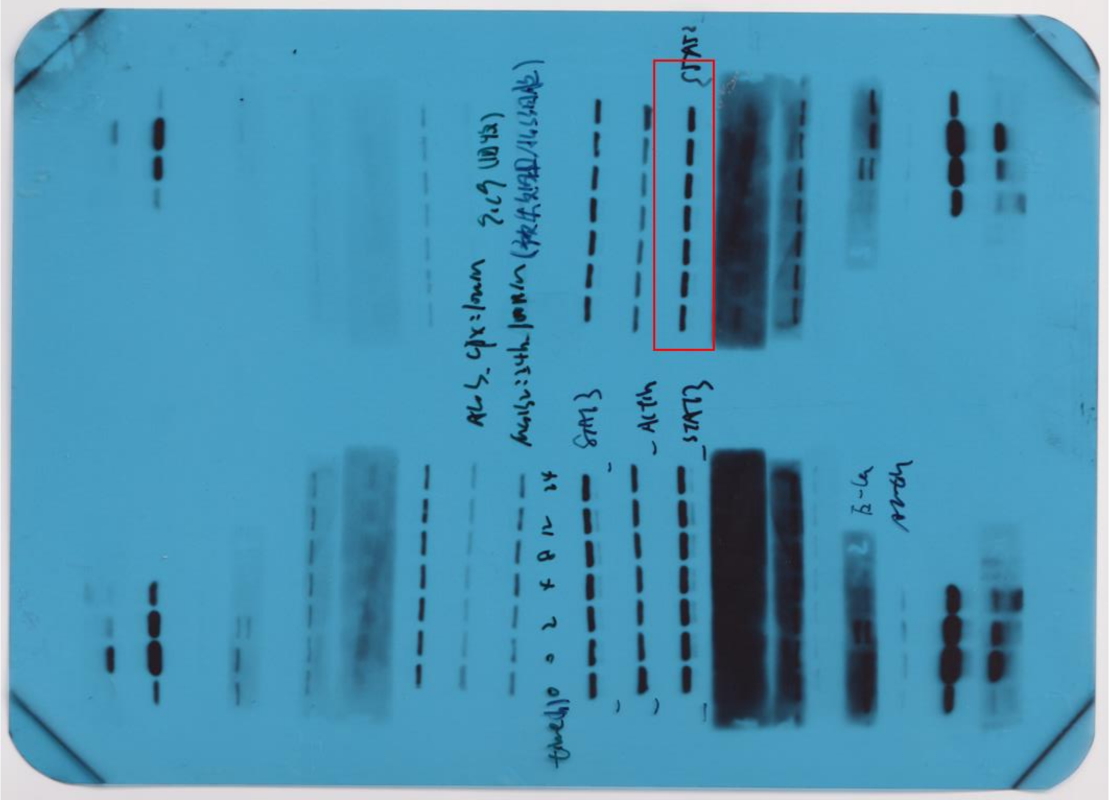

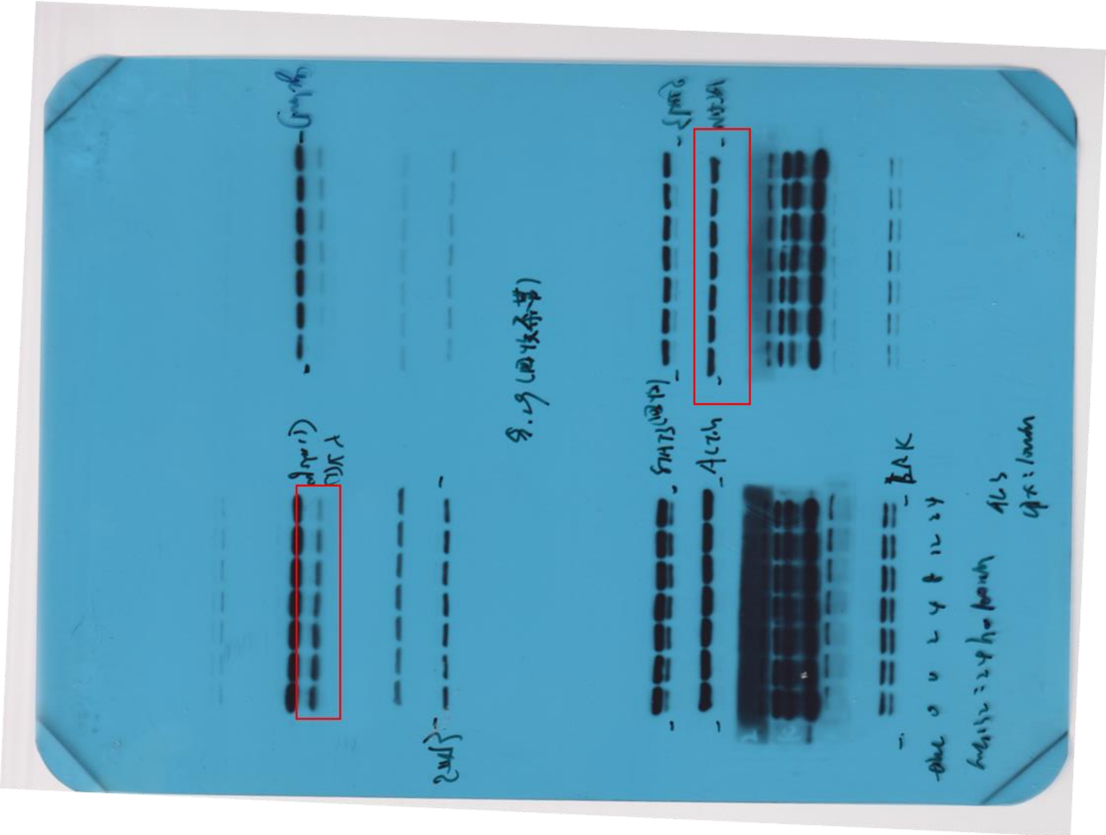

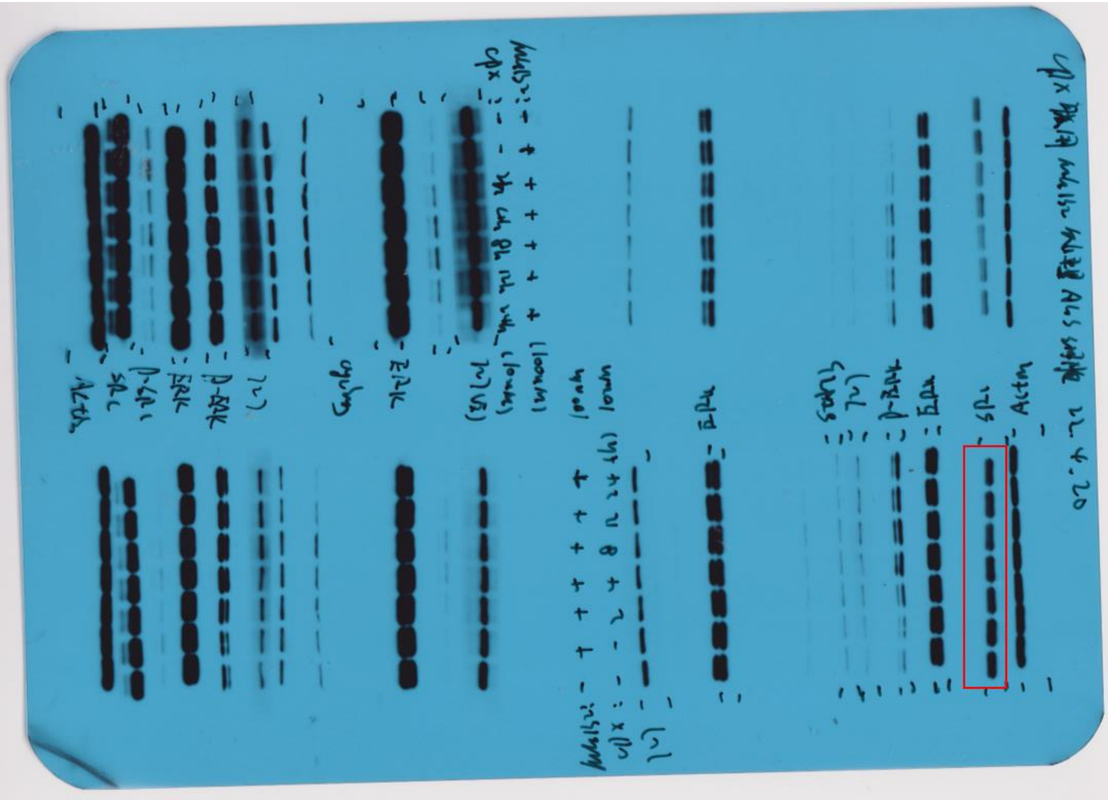

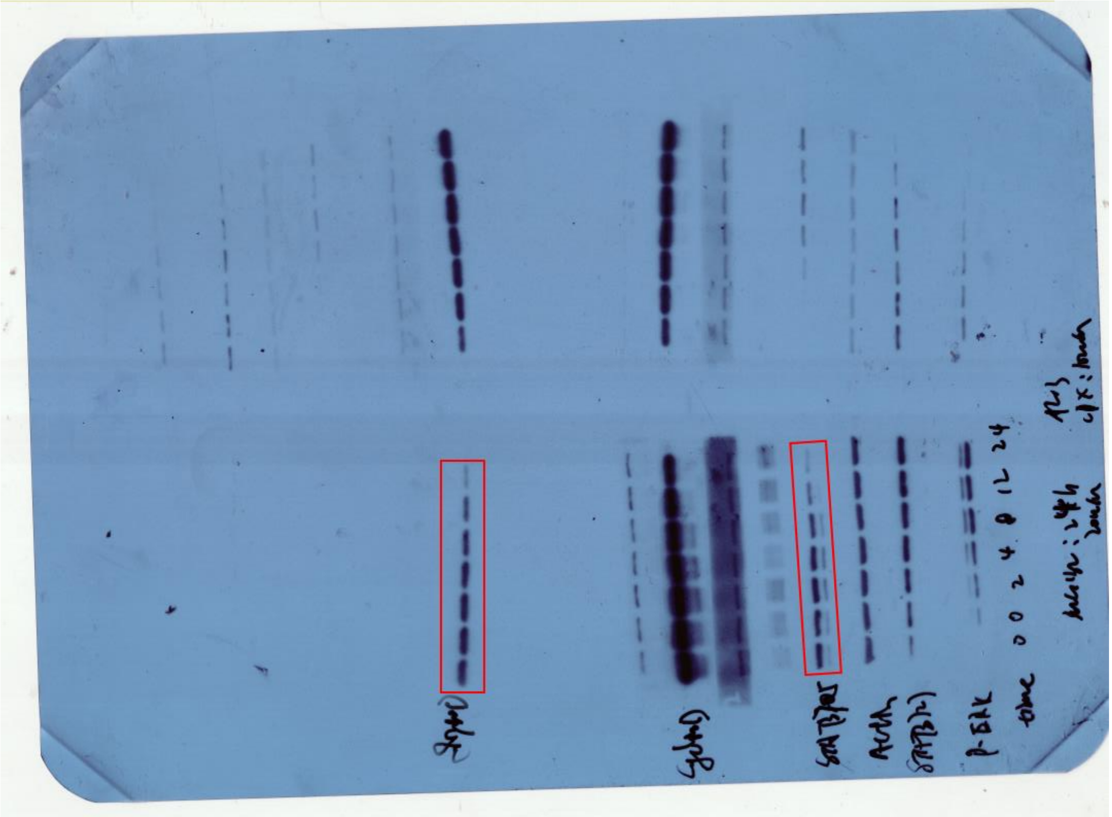
p-ERK
time
0 0 2 4 8 12 24

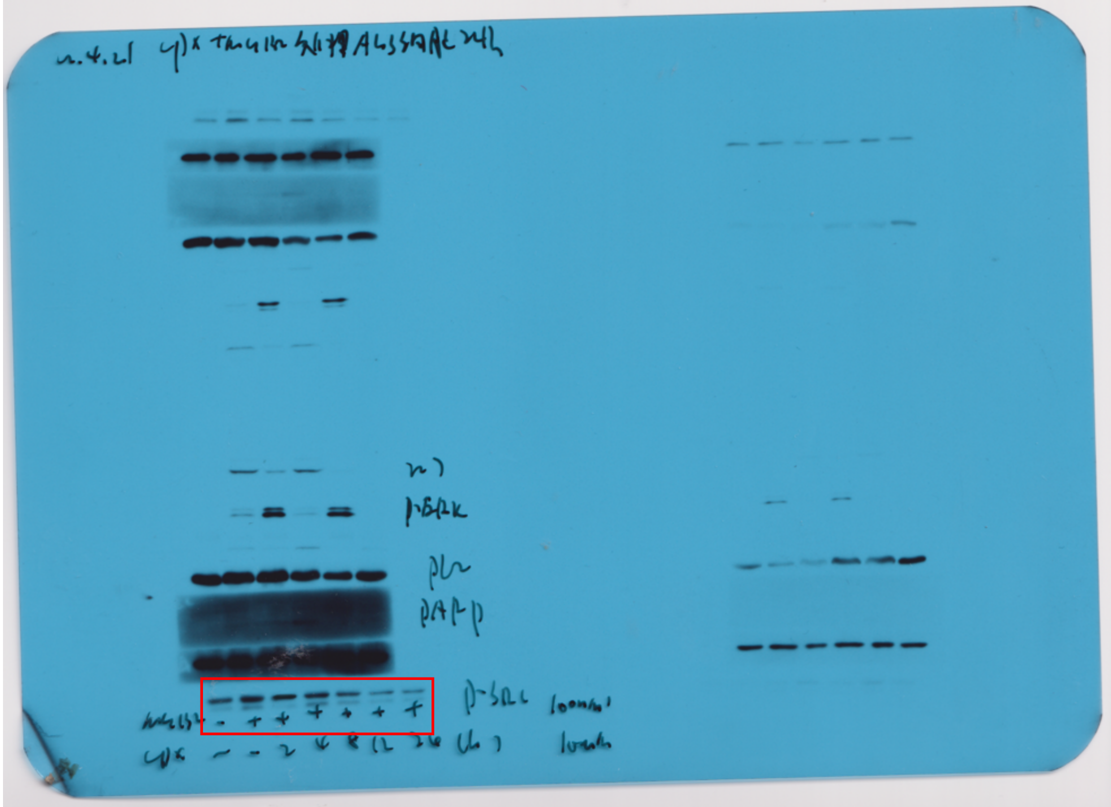
p-ERK
p-SRC

SGC cells

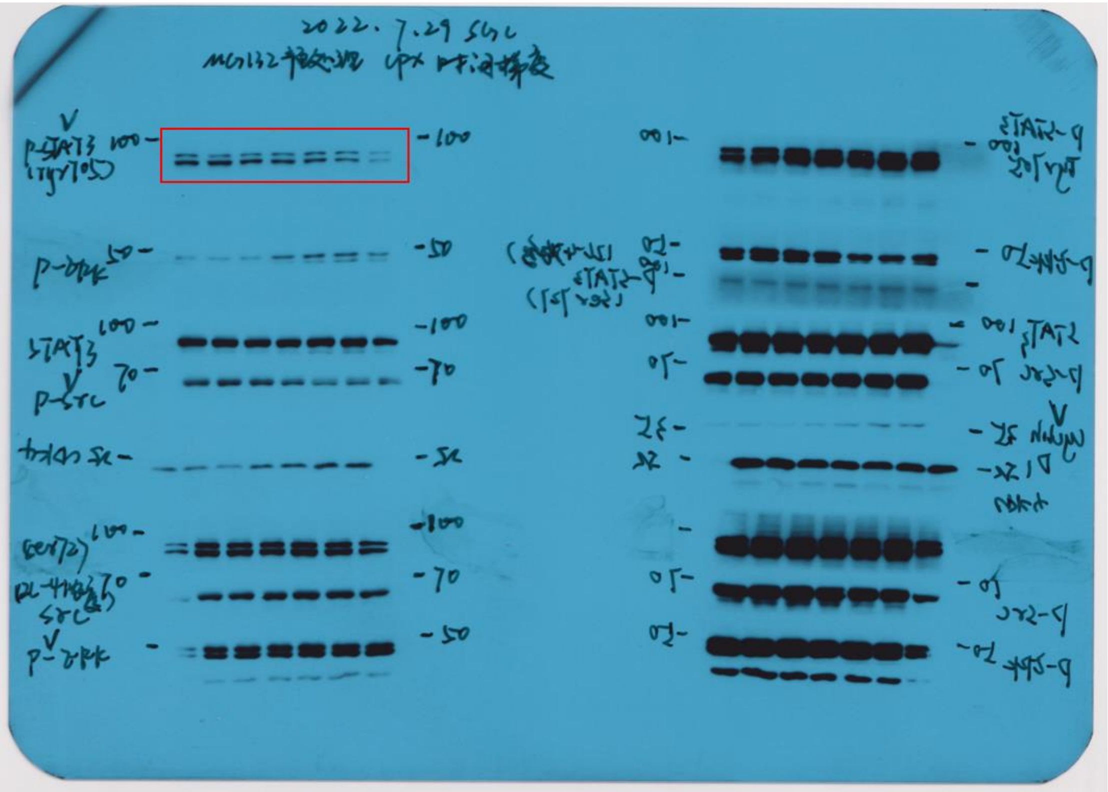
2022.7.29 SGC
p-STAT3 (Tyr705)
p-ERK
STAT3
p-Src
(Ser727)
Src
p-ERK
100
50
70

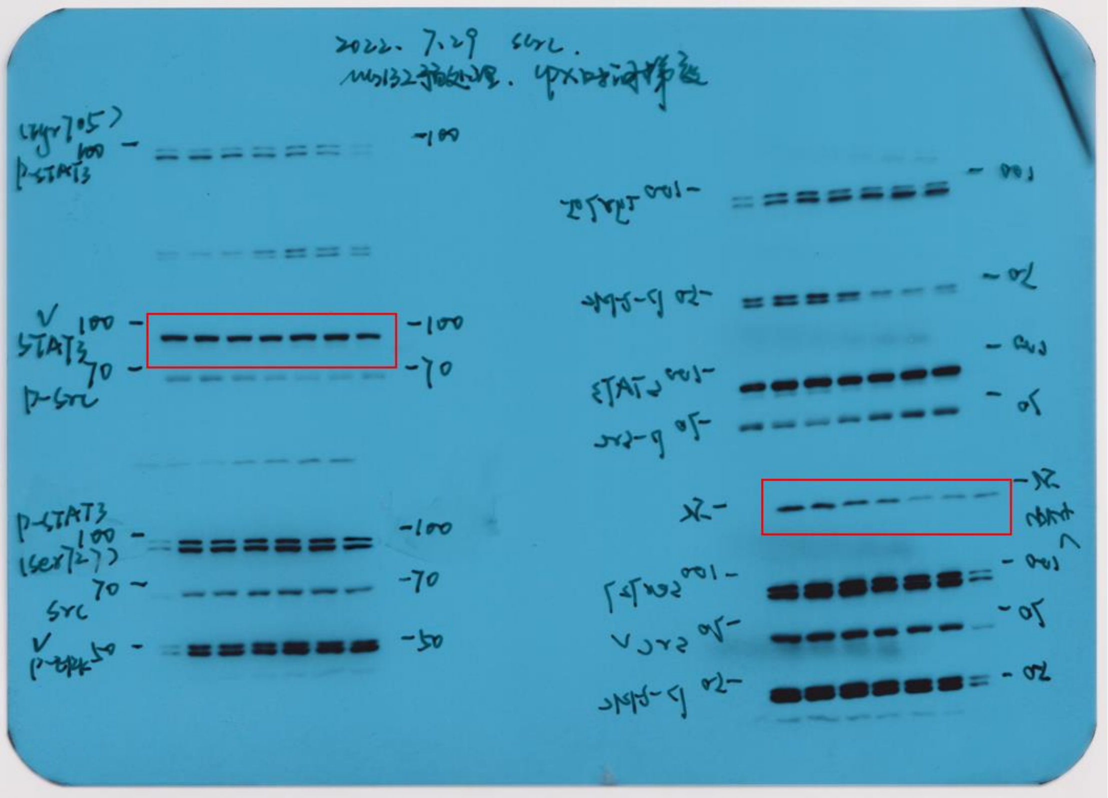
2022.7.29 SGC.
(Tyr705)
p-STAT3
STAT3
p-Src
p-STAT3
(Ser727)
Src
p-ERK
100
70
50

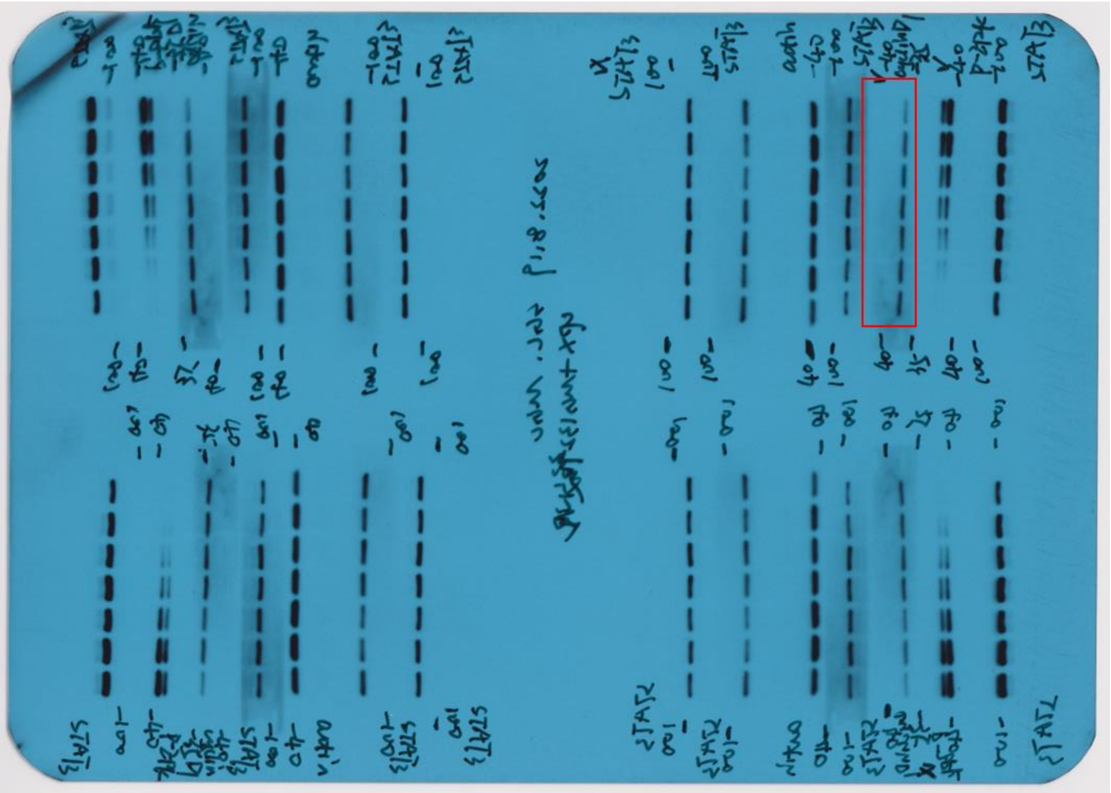

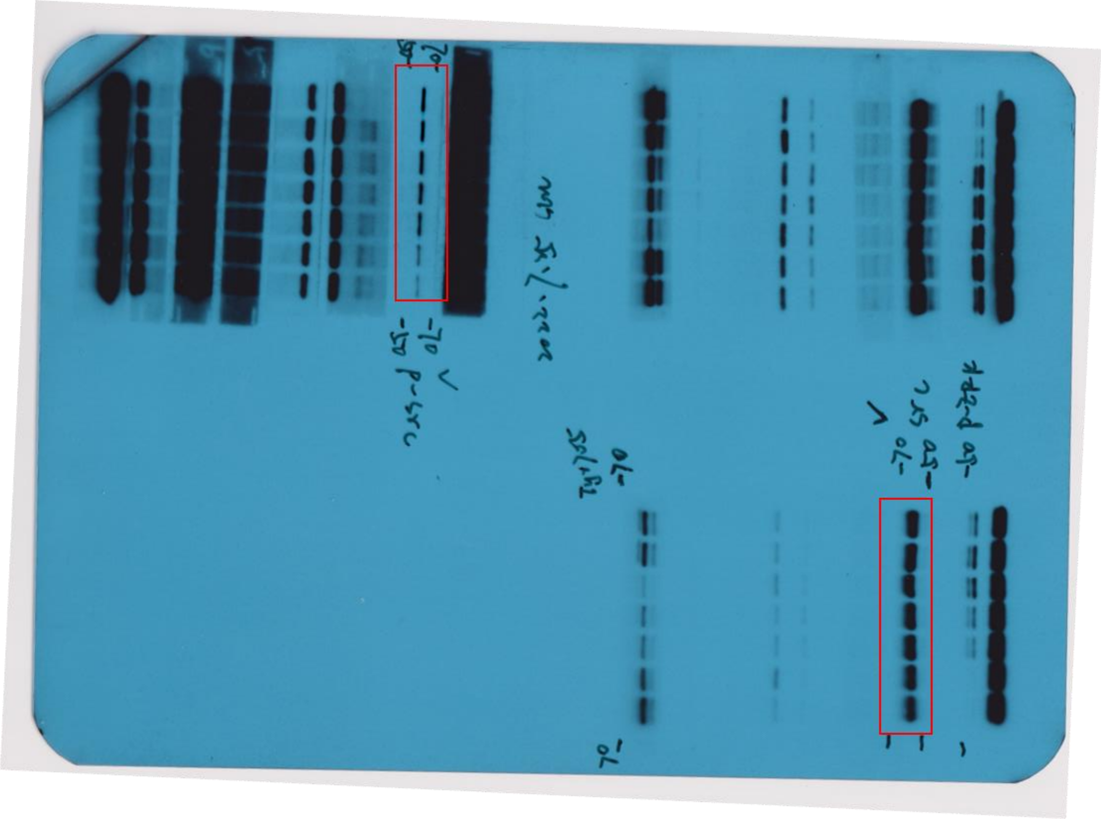

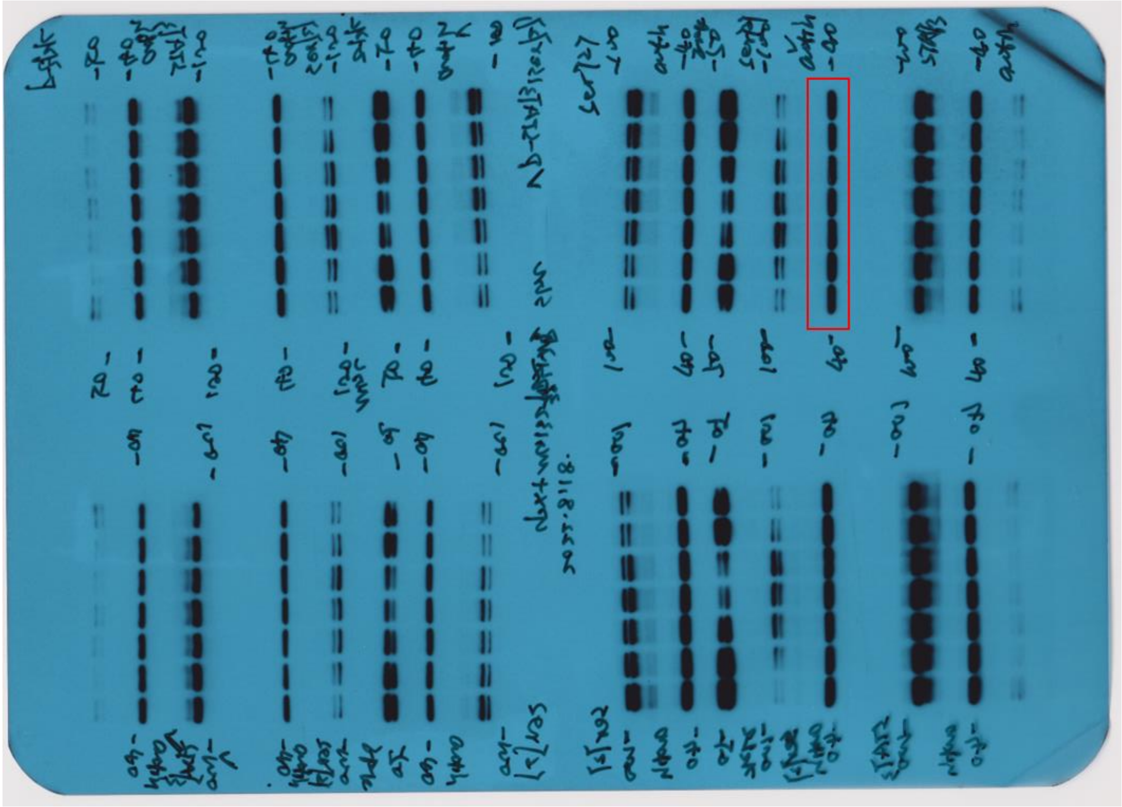

Figure. 4B

AGS cells

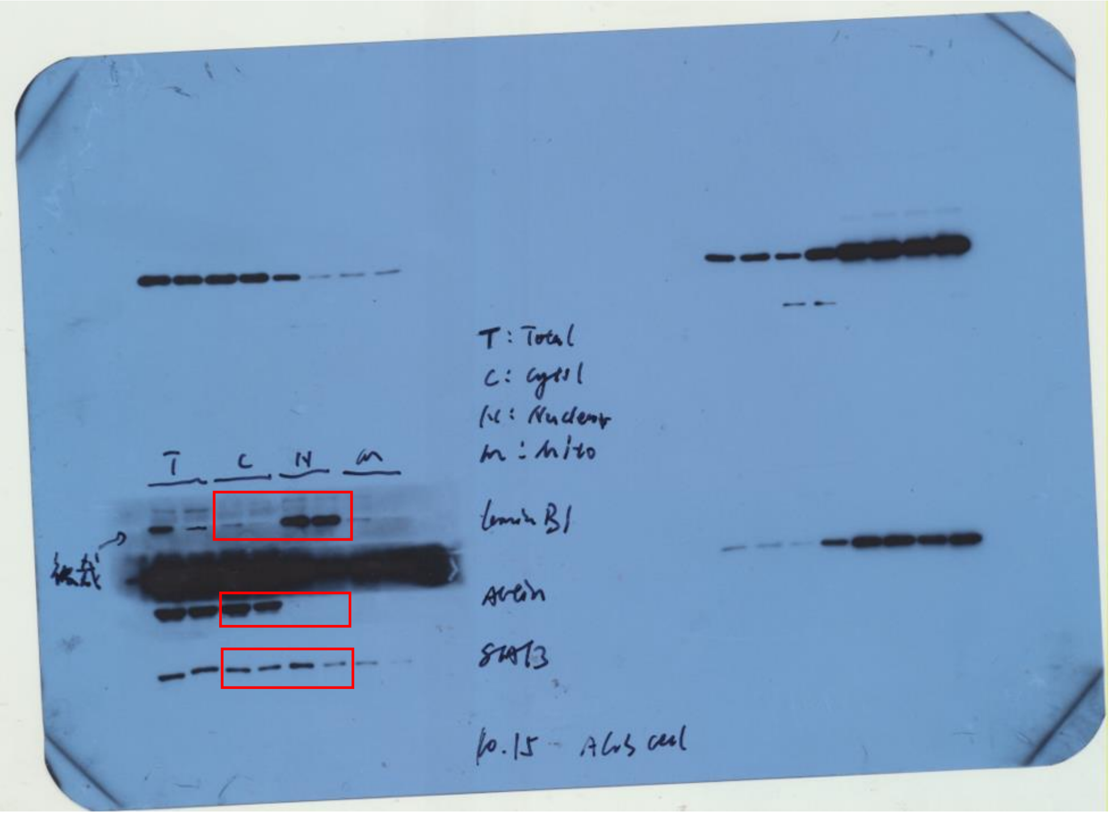

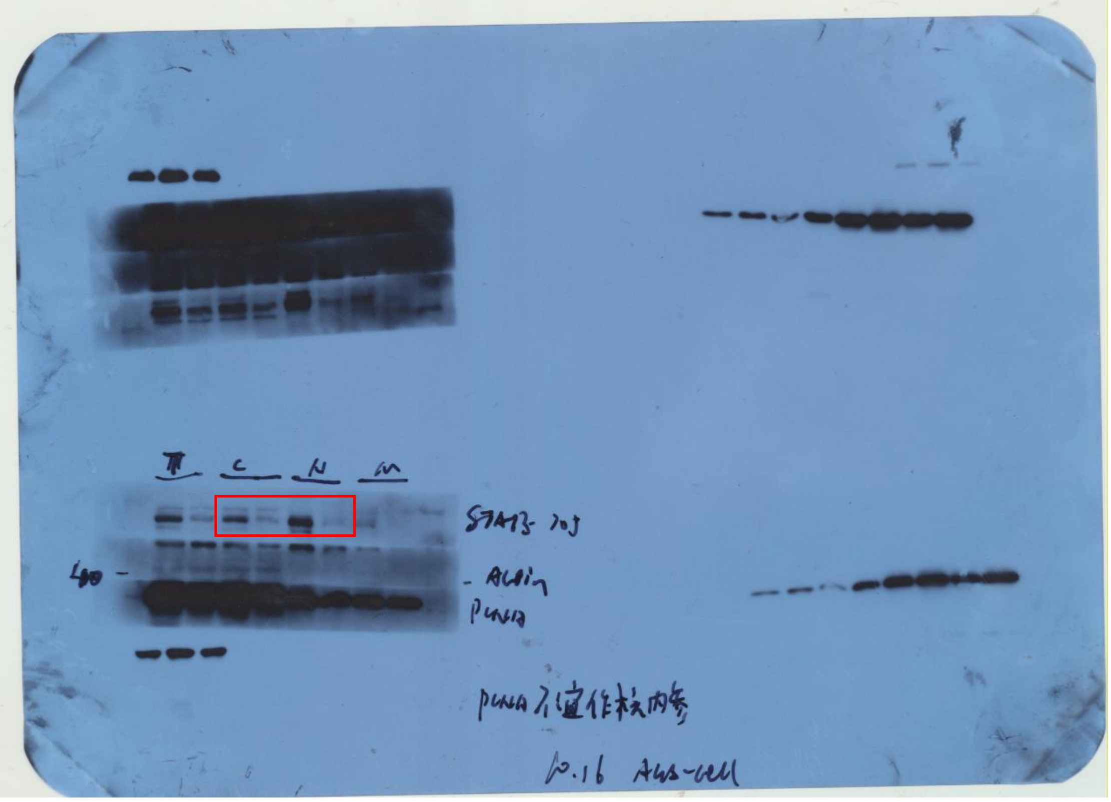

MGC and SGC cells

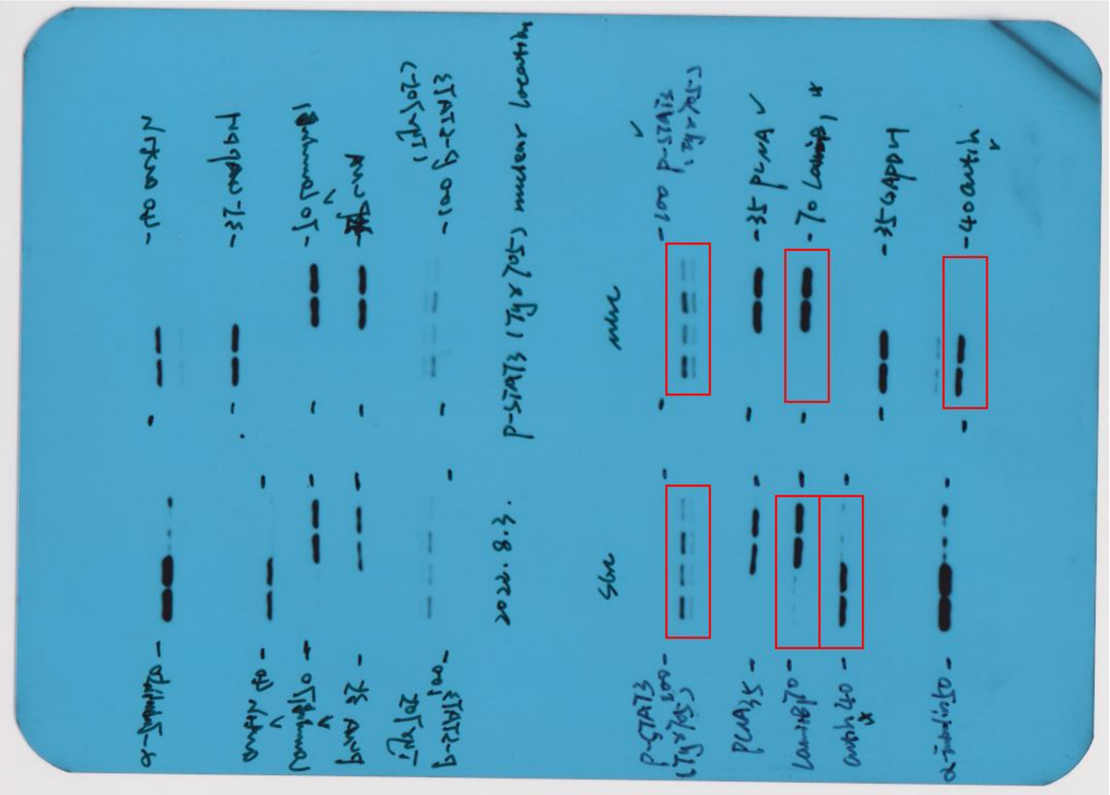

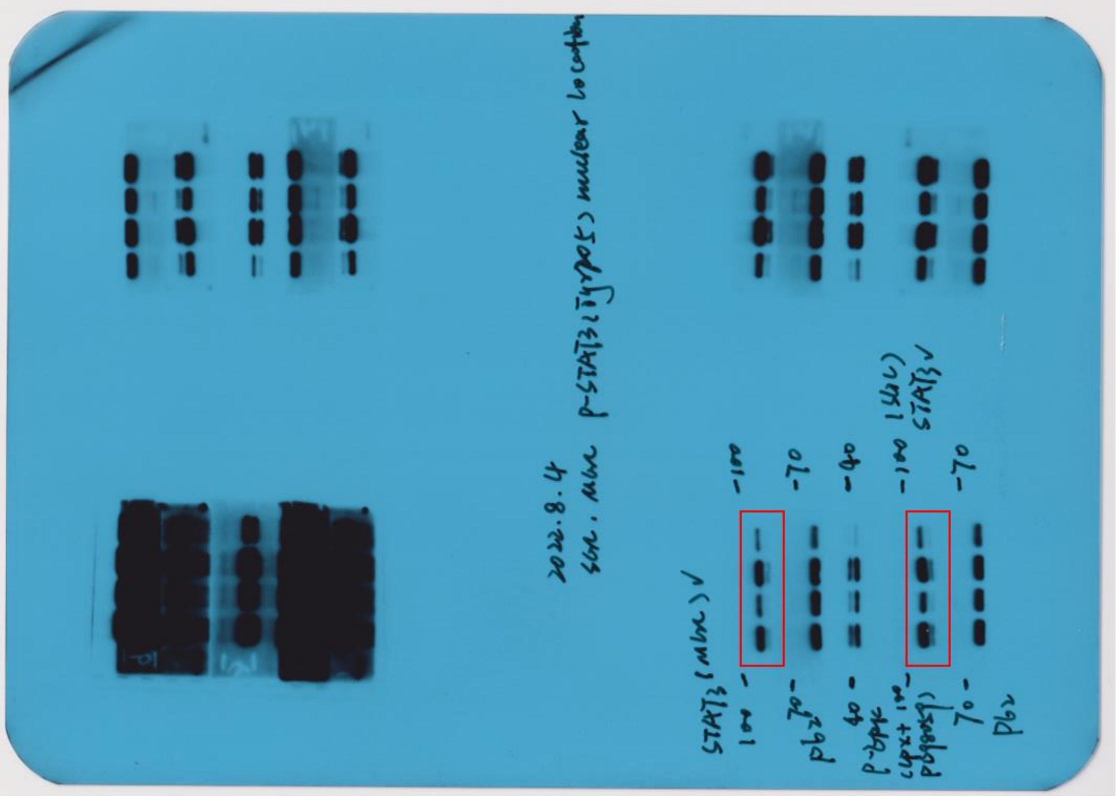

Figure. 4C

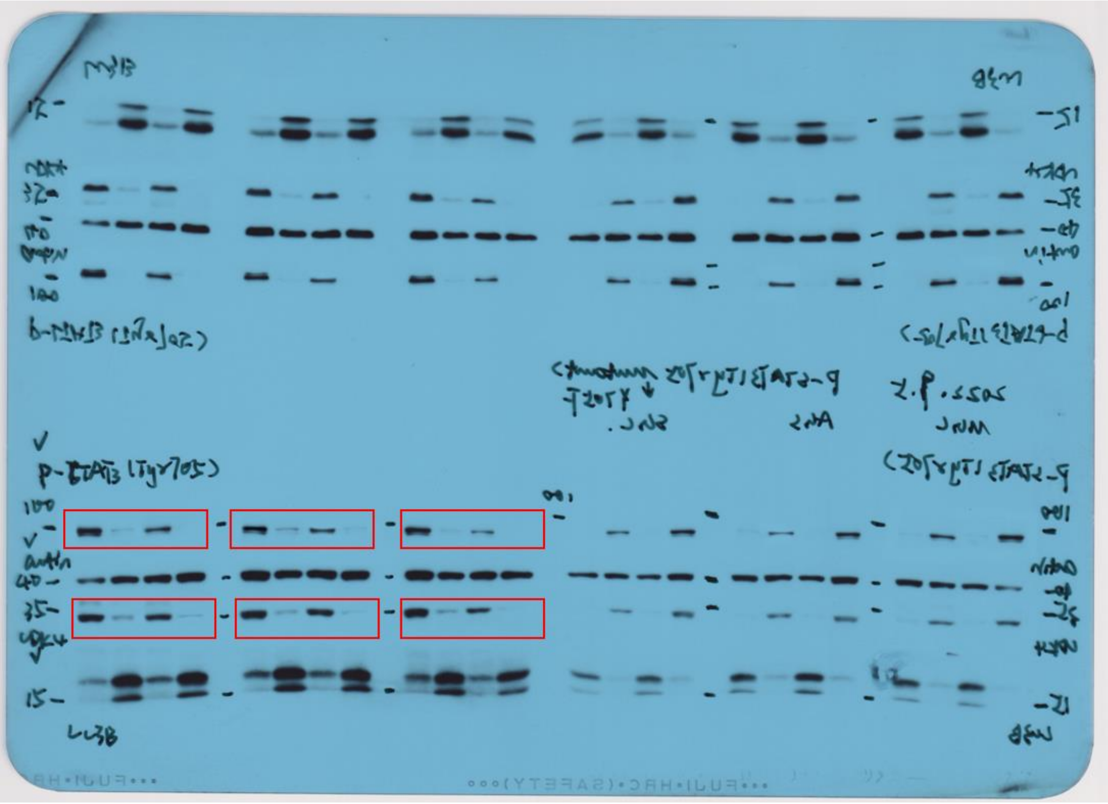

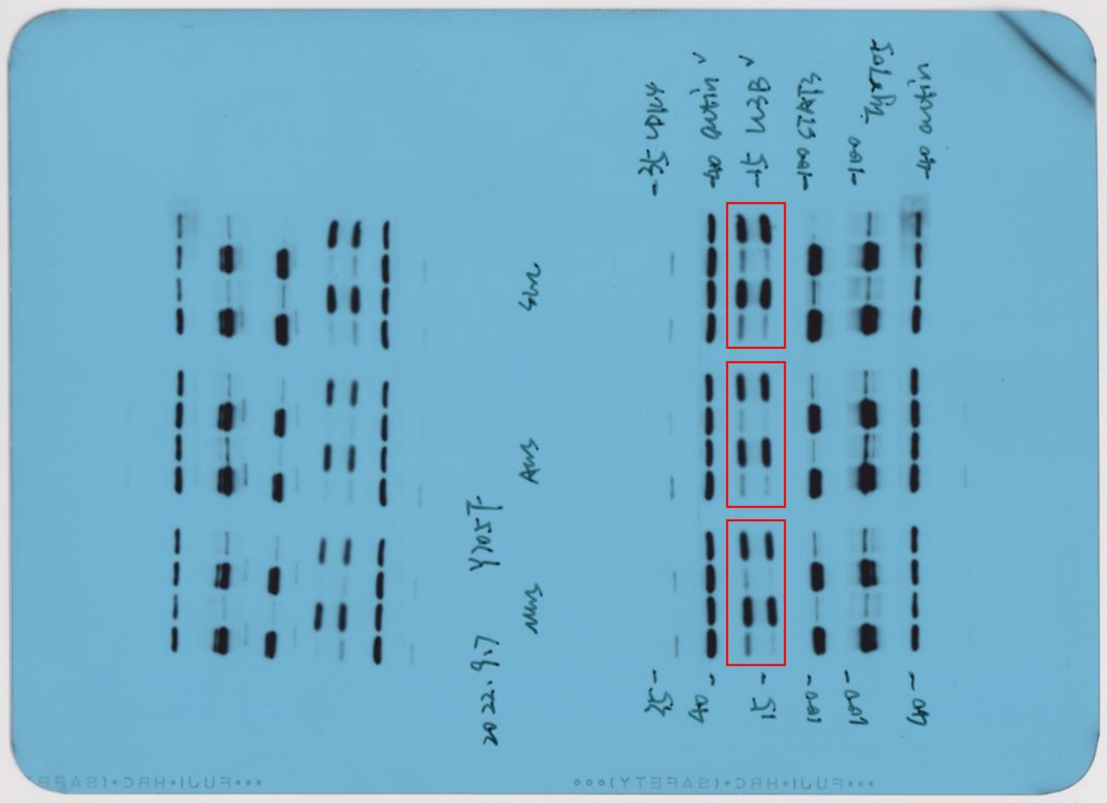

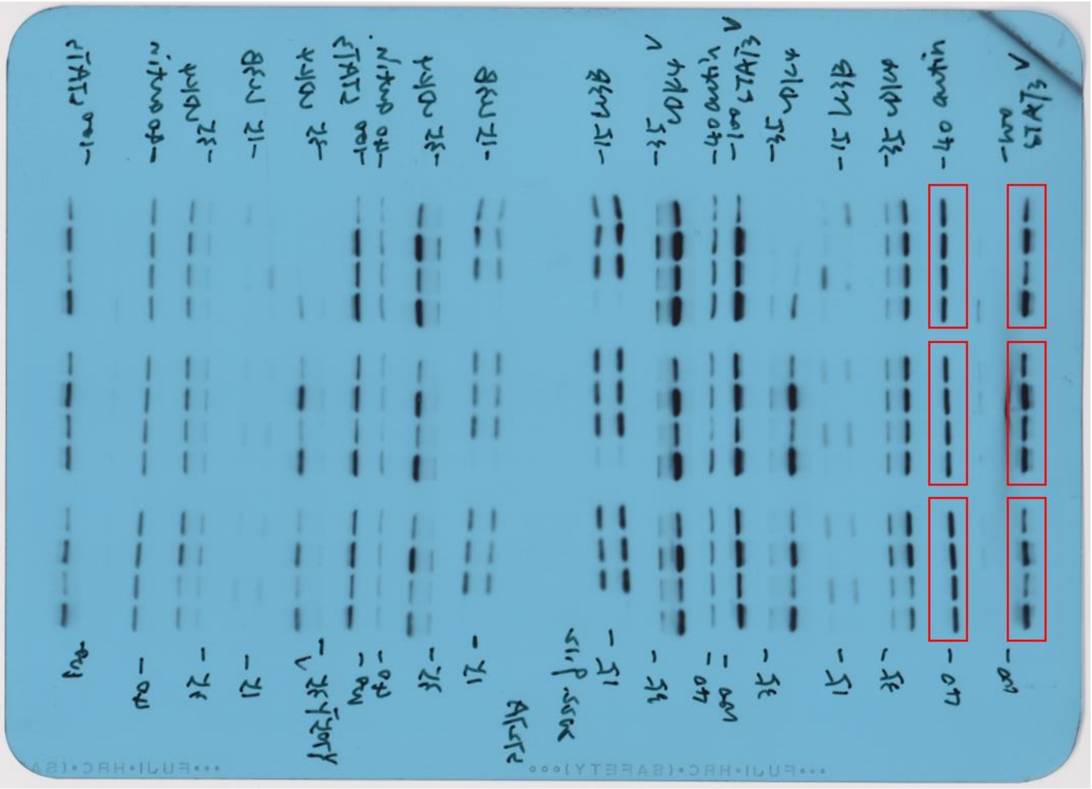

Figure. 5A

AGS cells

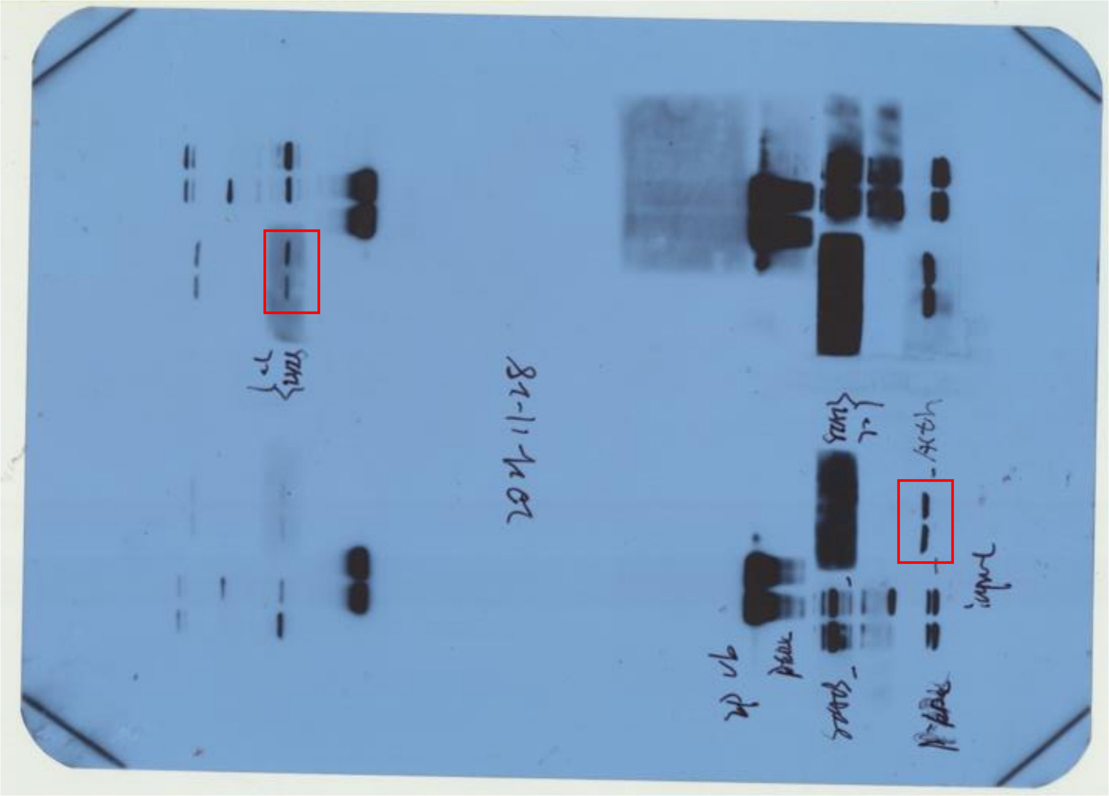

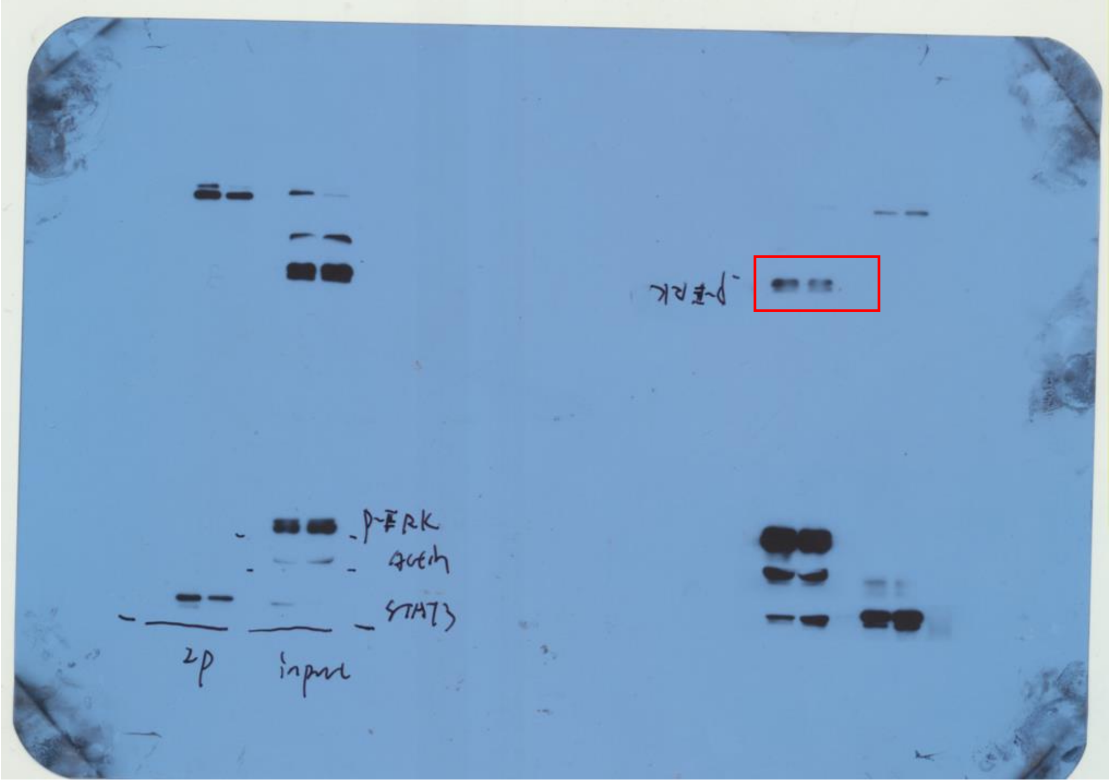

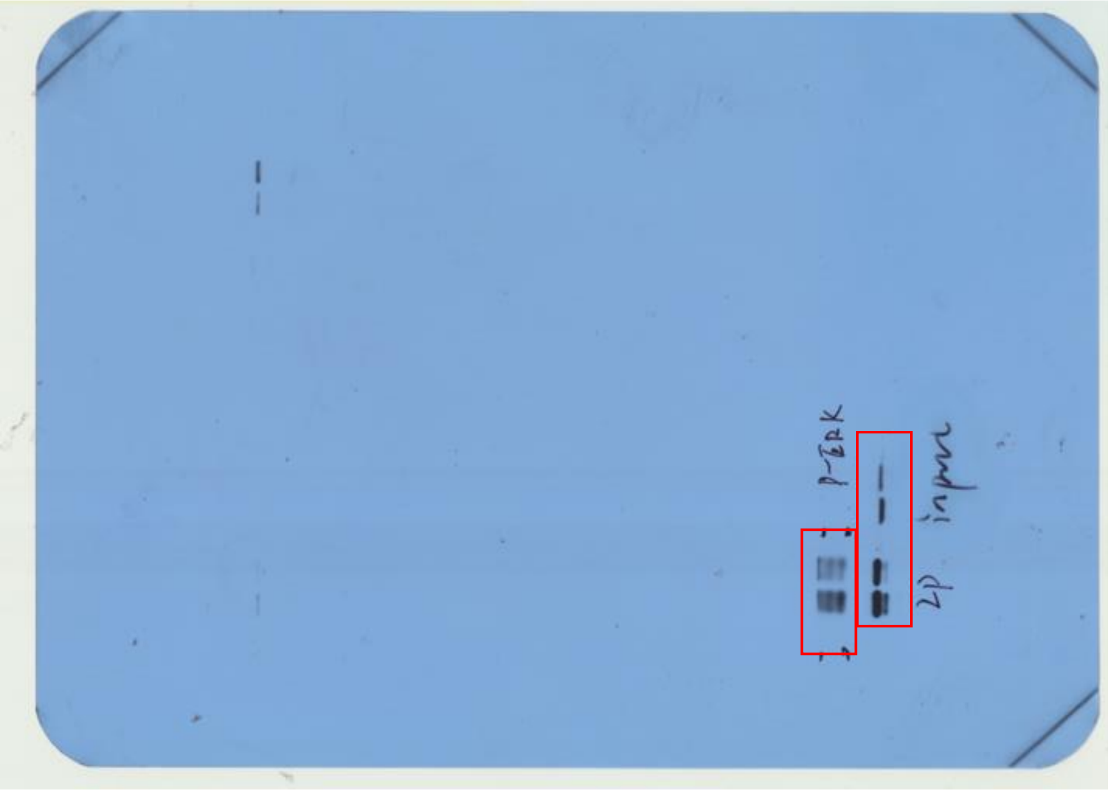
p-ERK
input
IP

MGC and SGC cells

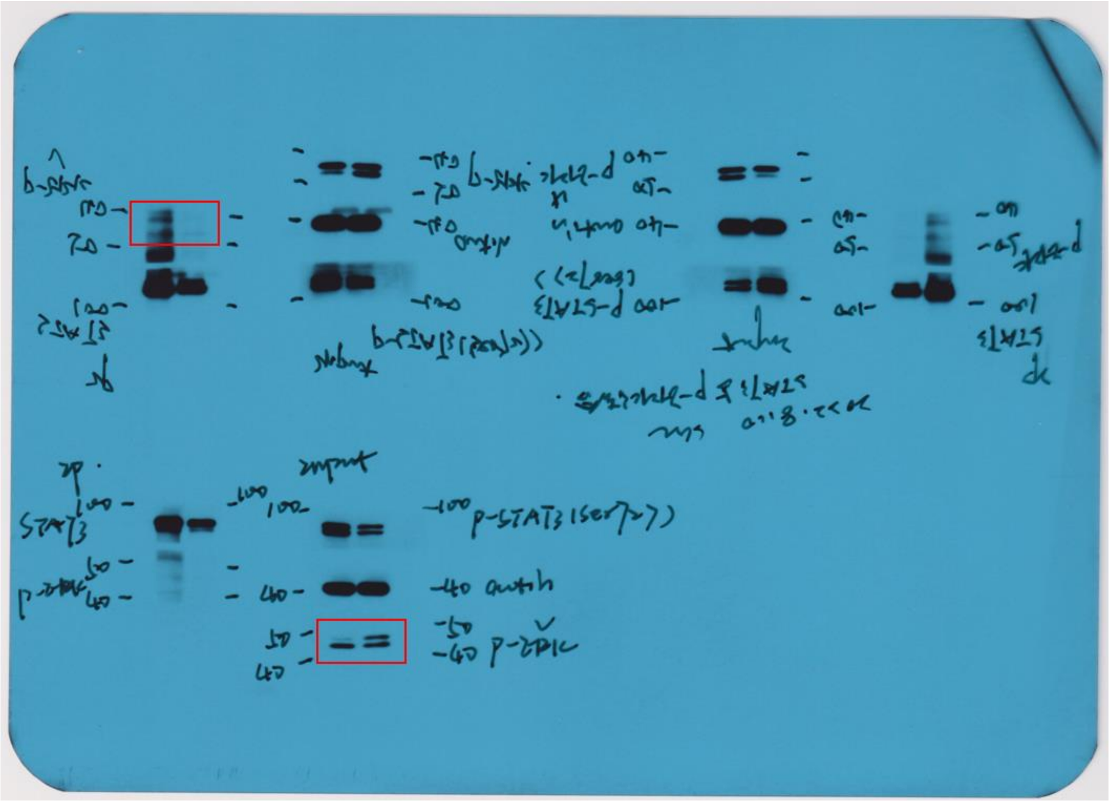

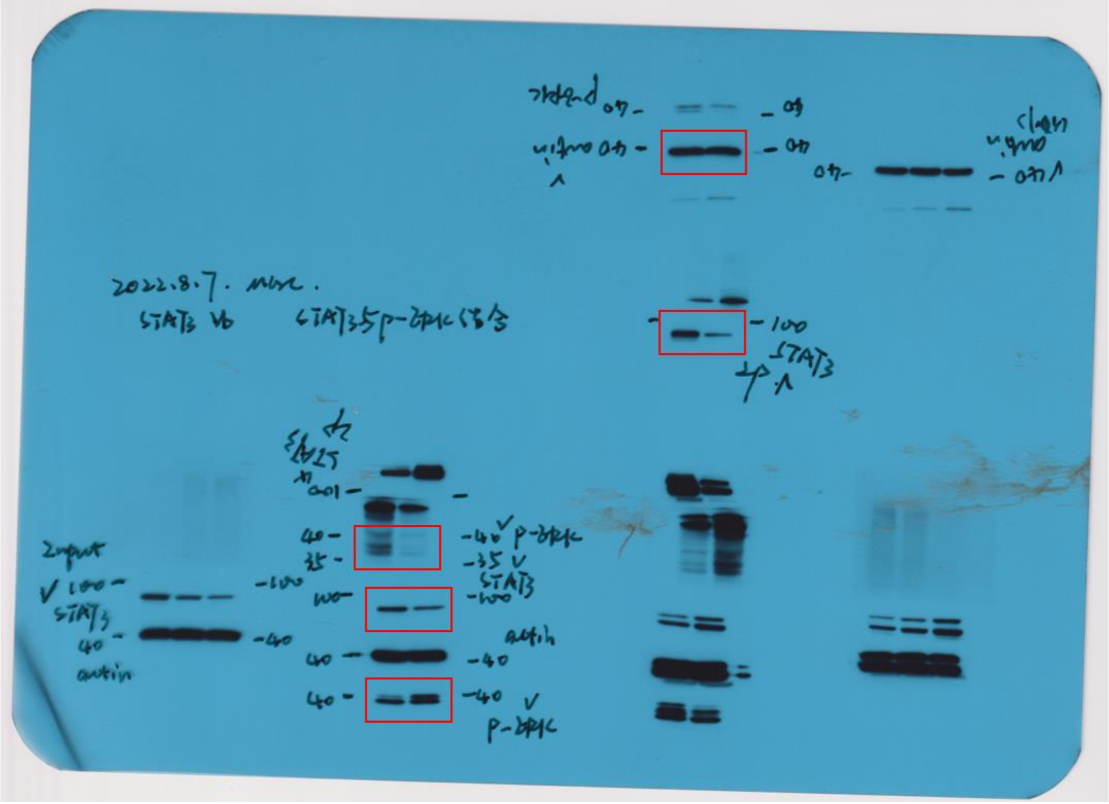

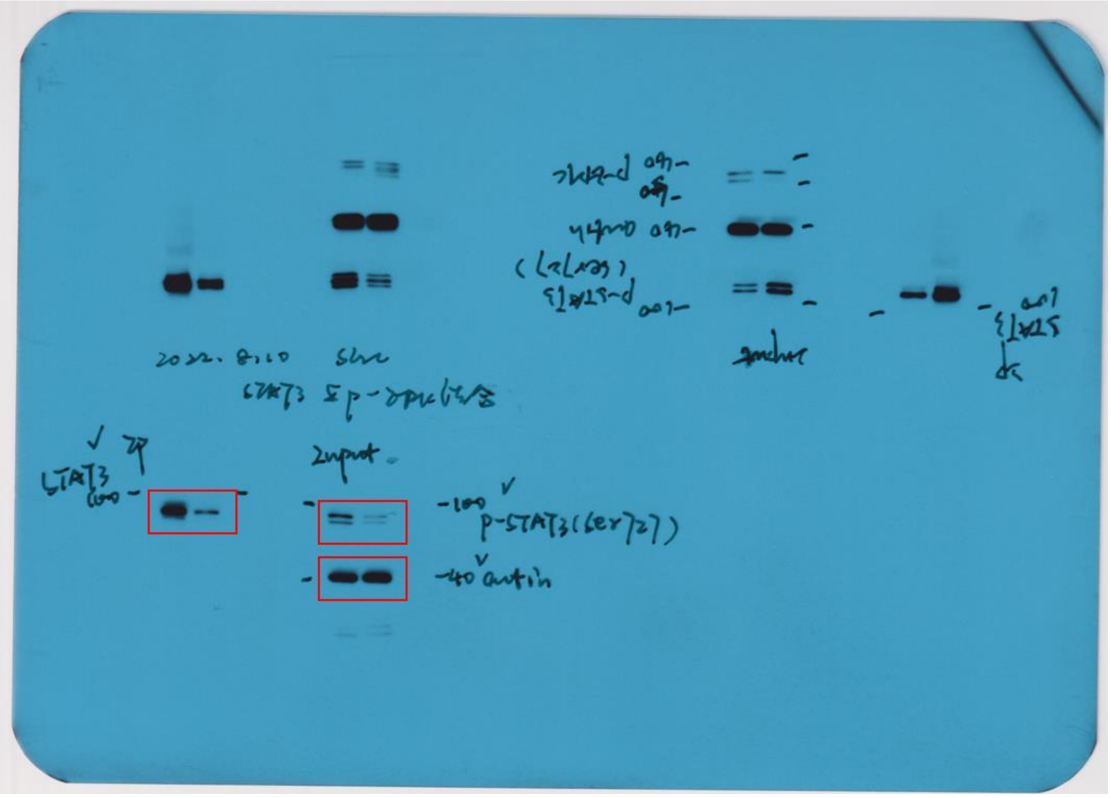
2022.8.10
STAT3
100
Input
-100 p-STAT3(Ser727)
-40 actin

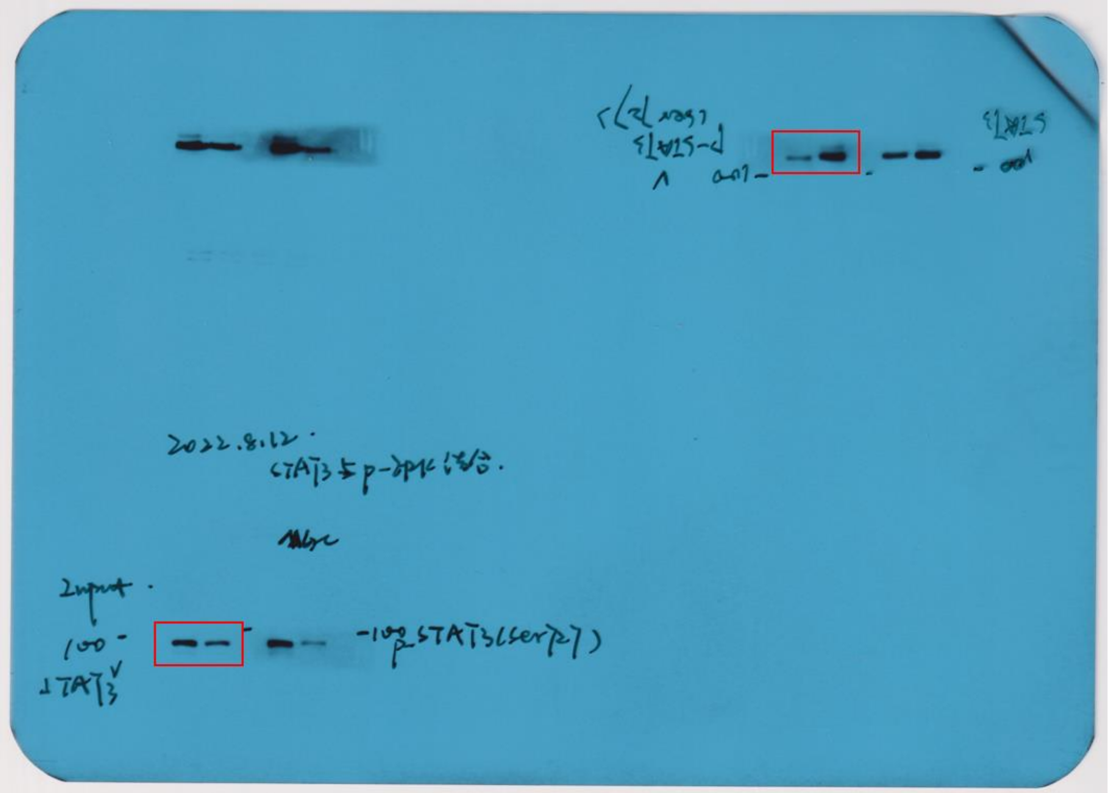
2022.8.12
Input
100
STAT3
-100 p-STAT3(Ser727)

Figure. 5B

MGC cells

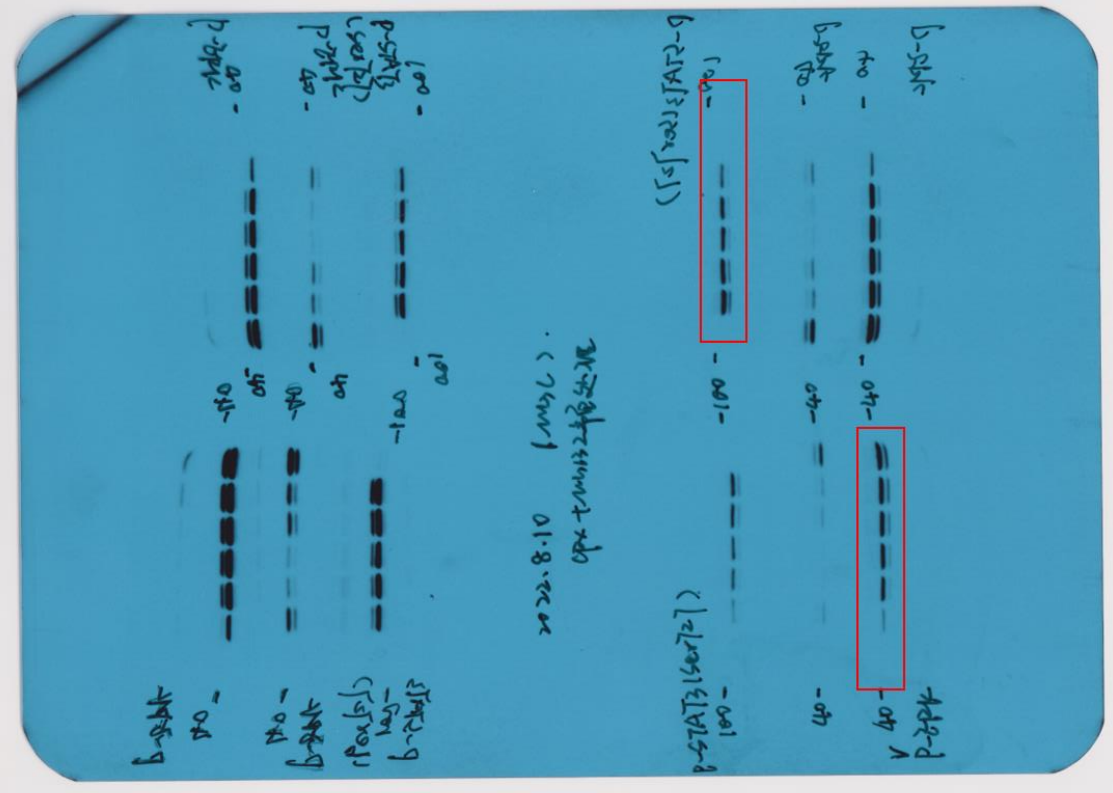

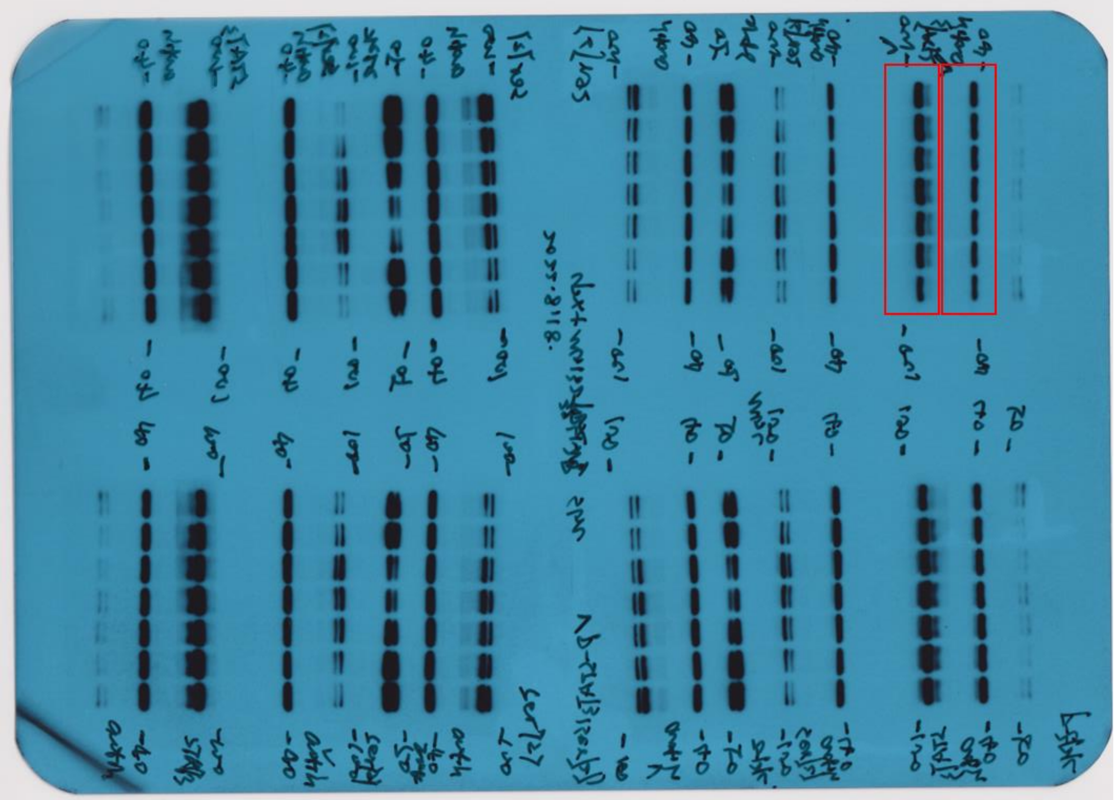

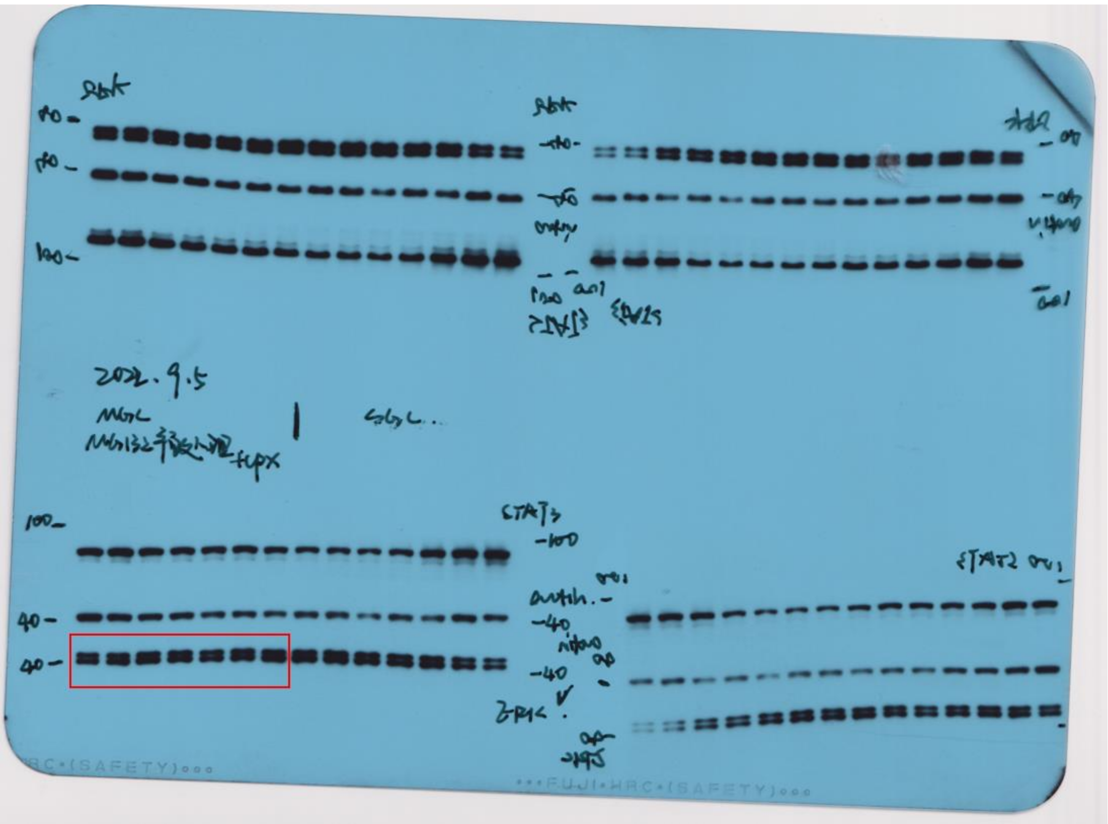

AGS cells

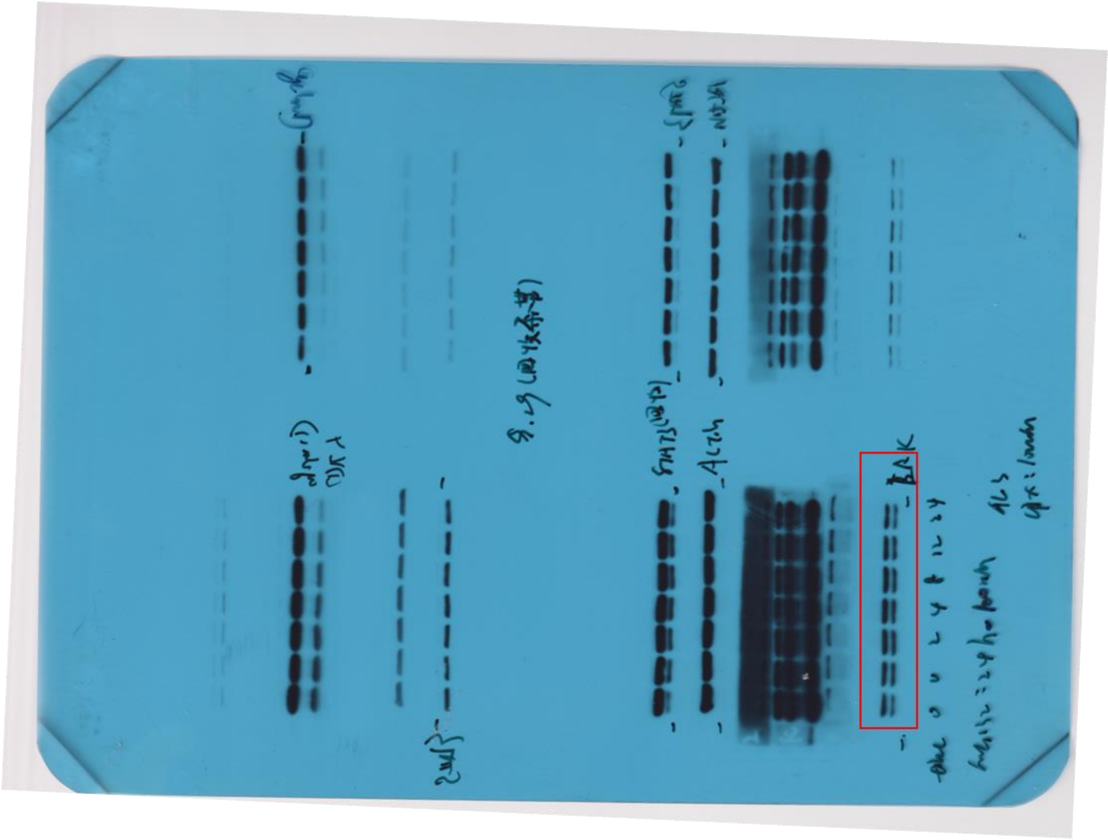

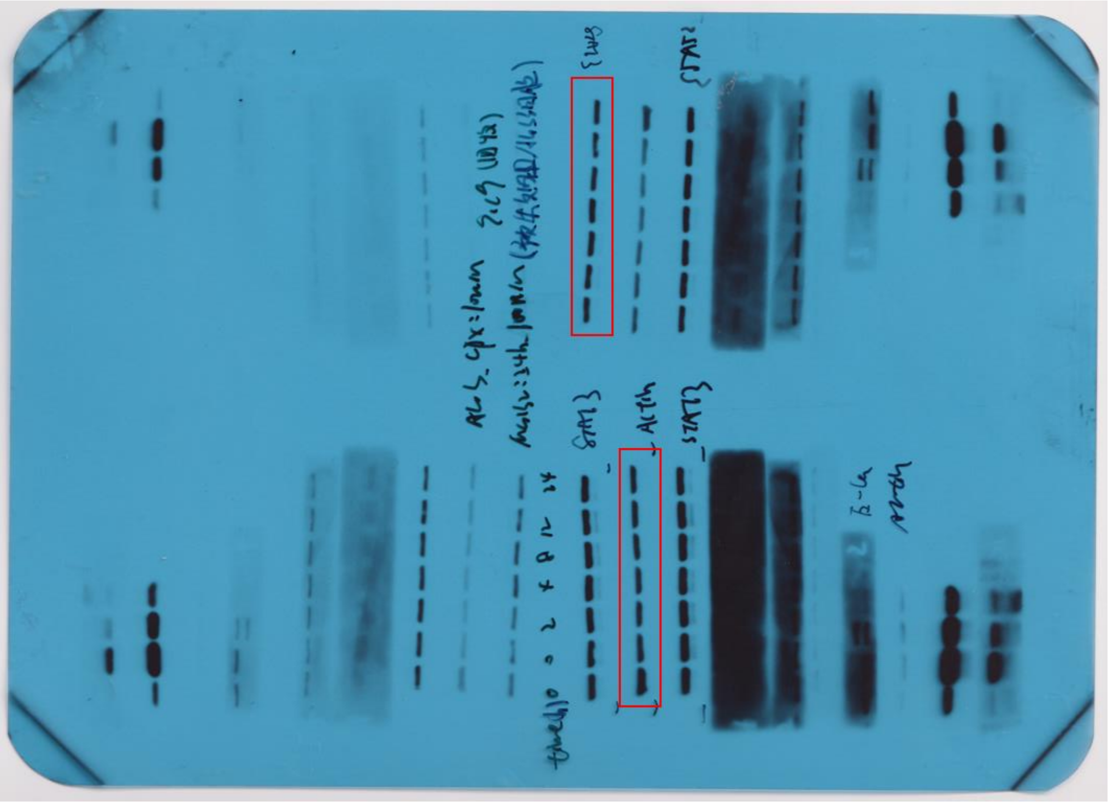

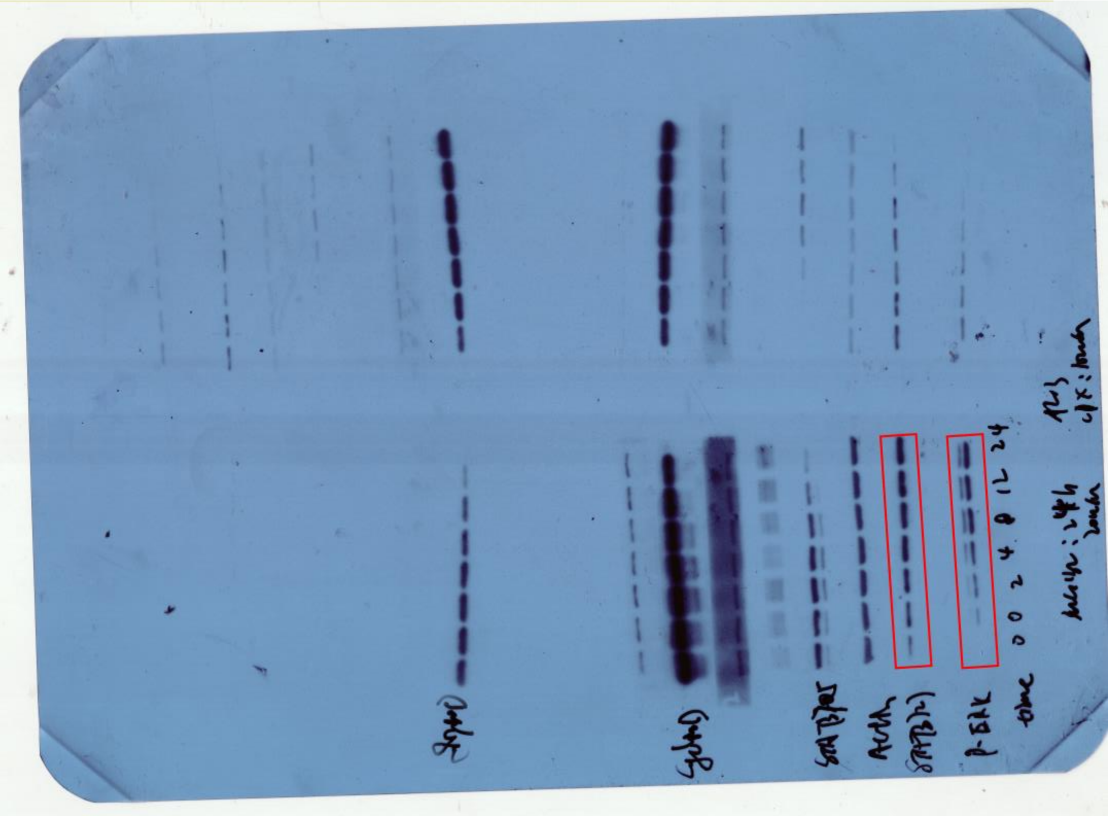

SGC cells

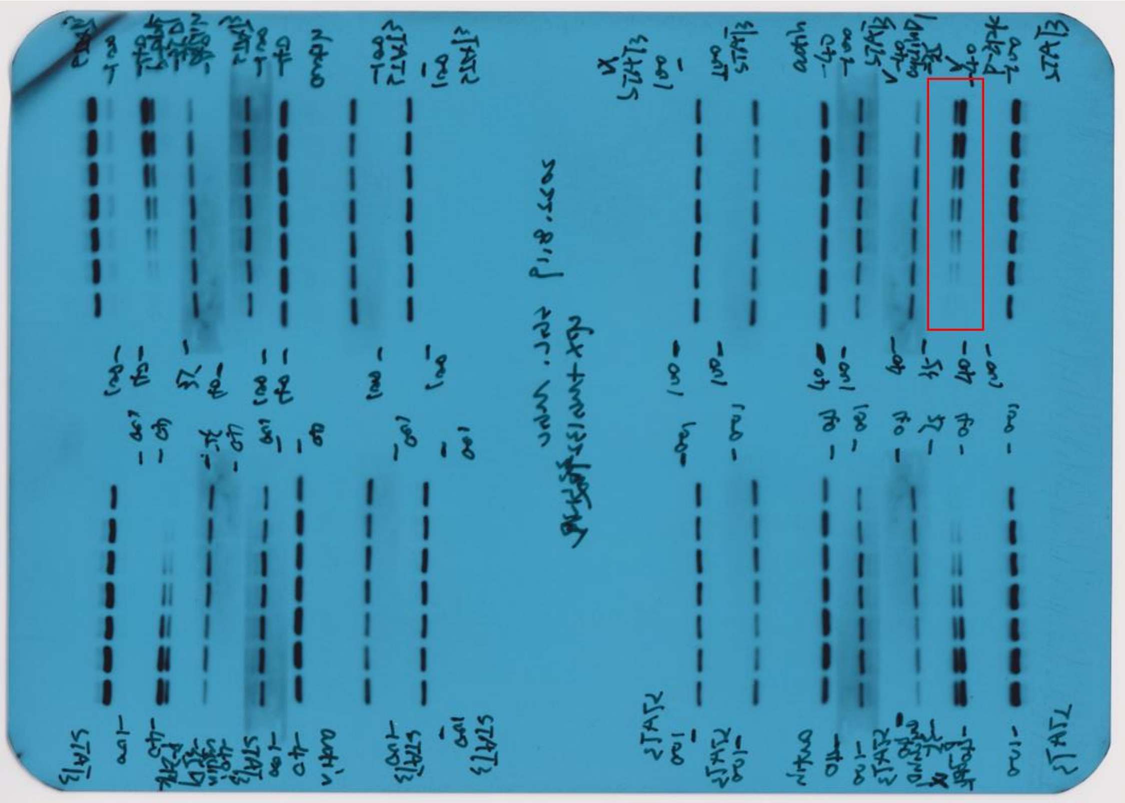

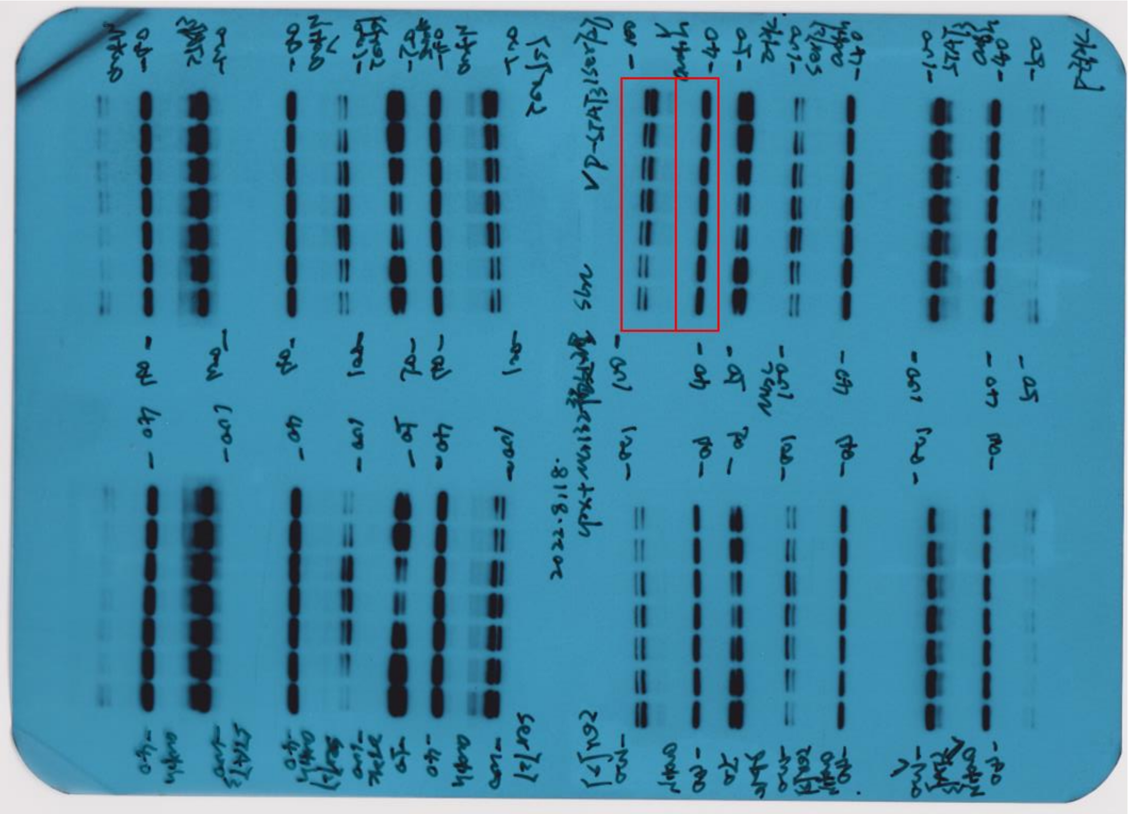

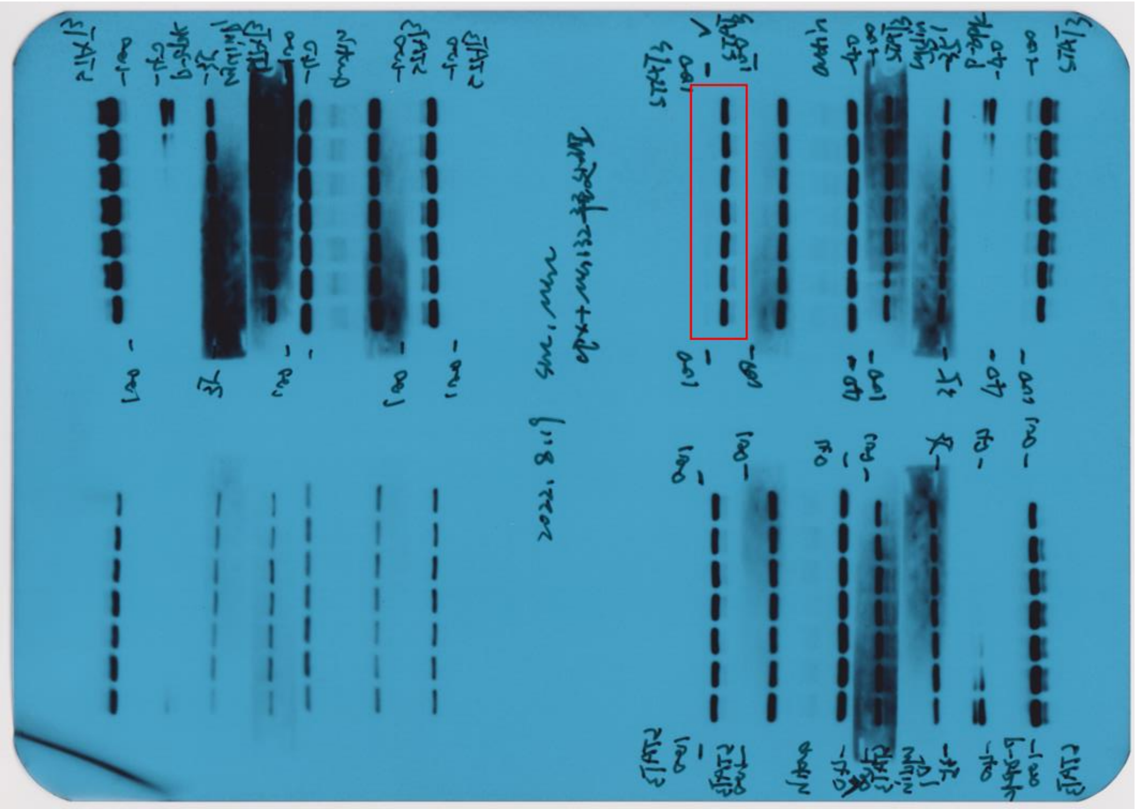

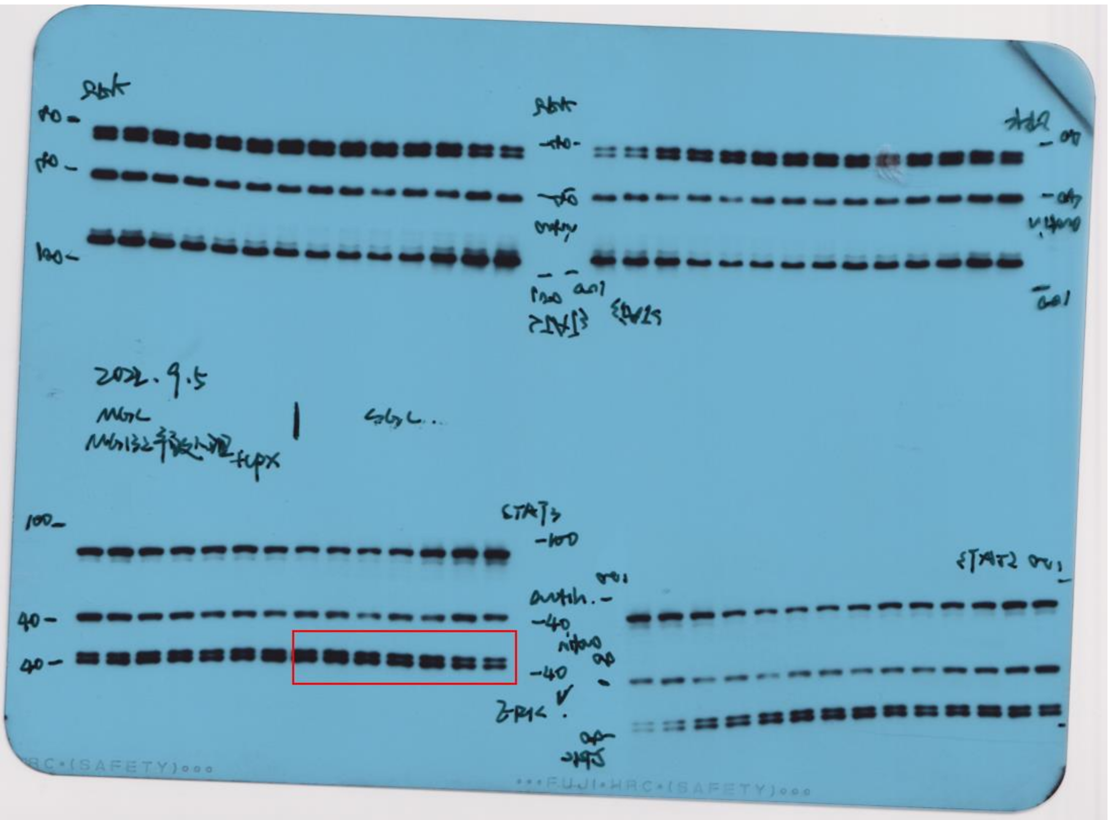

Figure. 5C

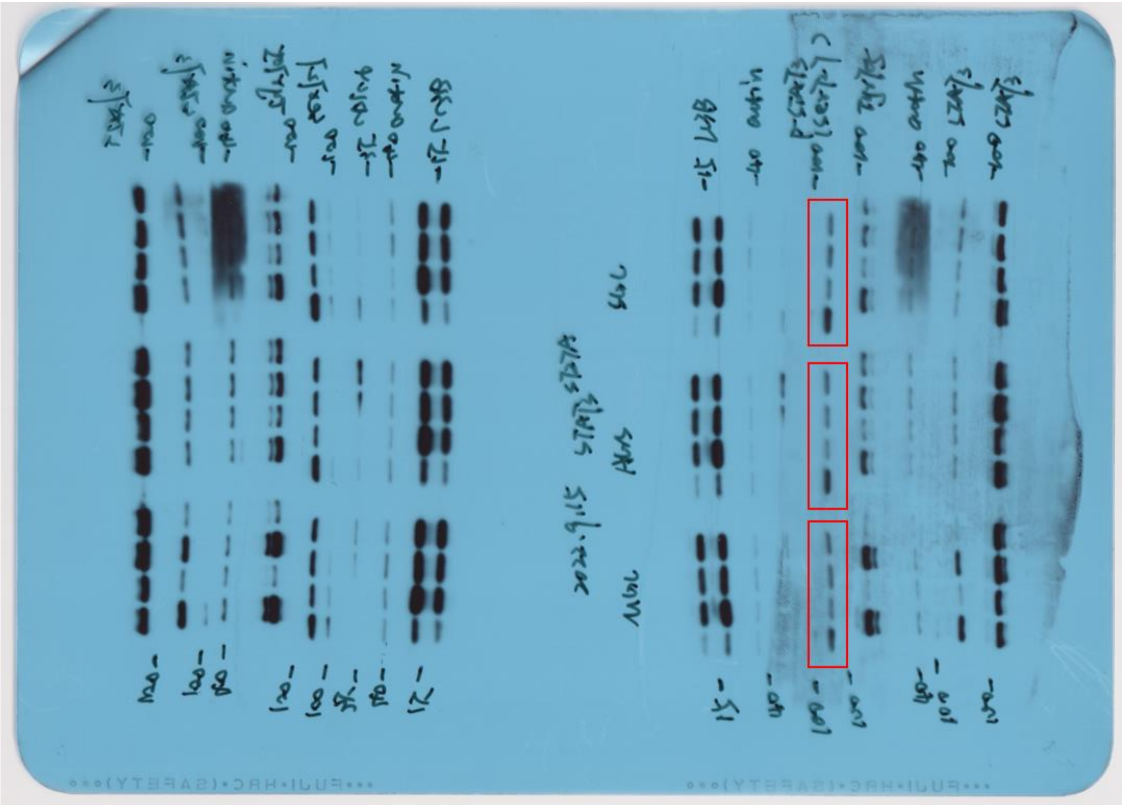

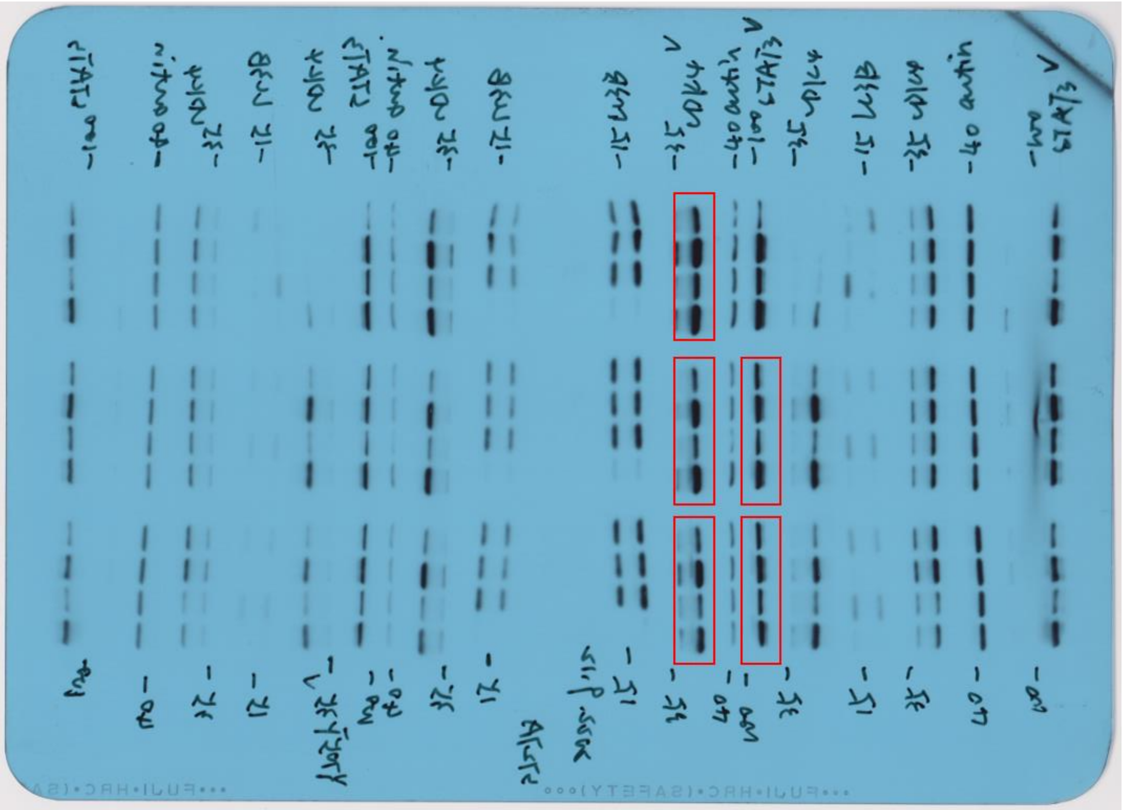

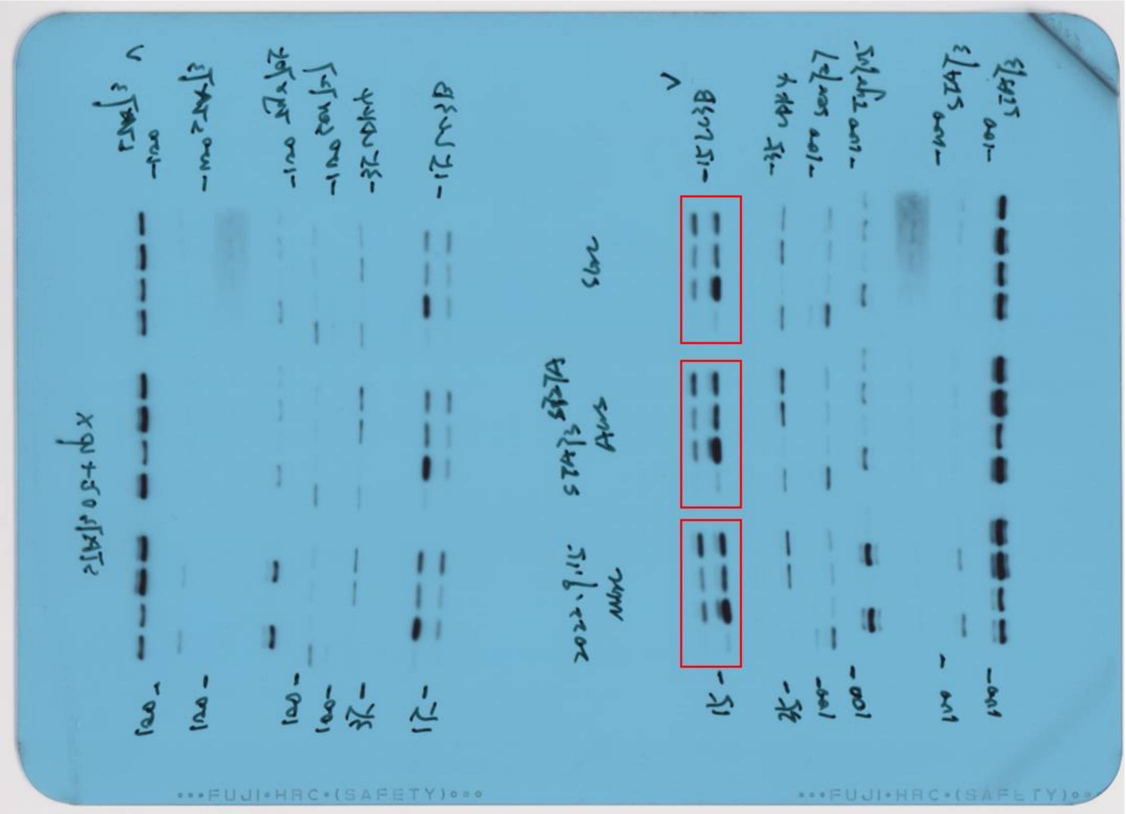

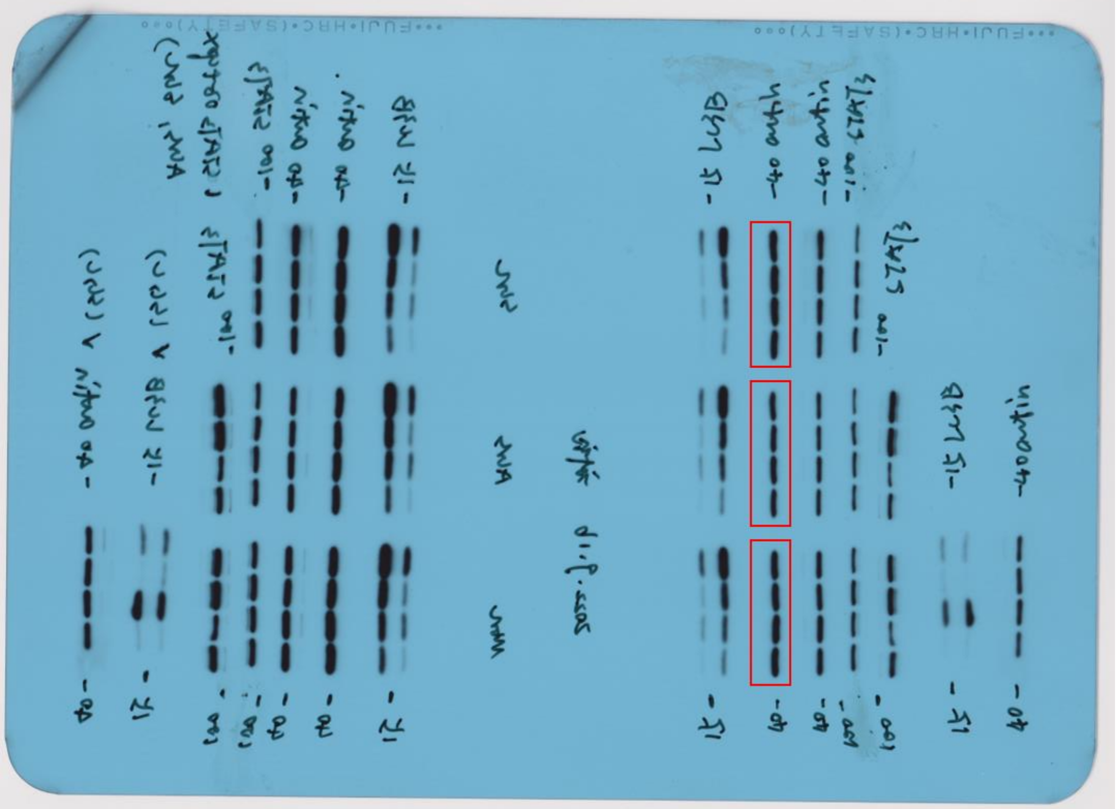

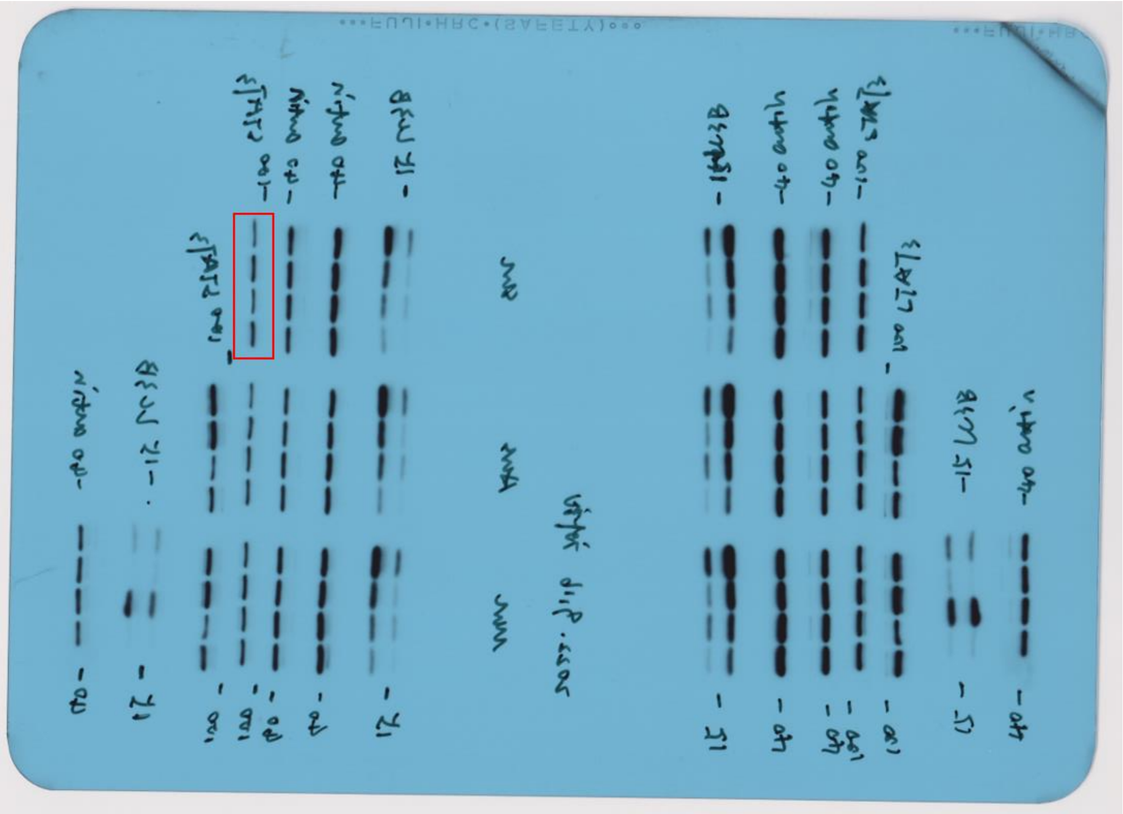

Figure S4a

MGC cells

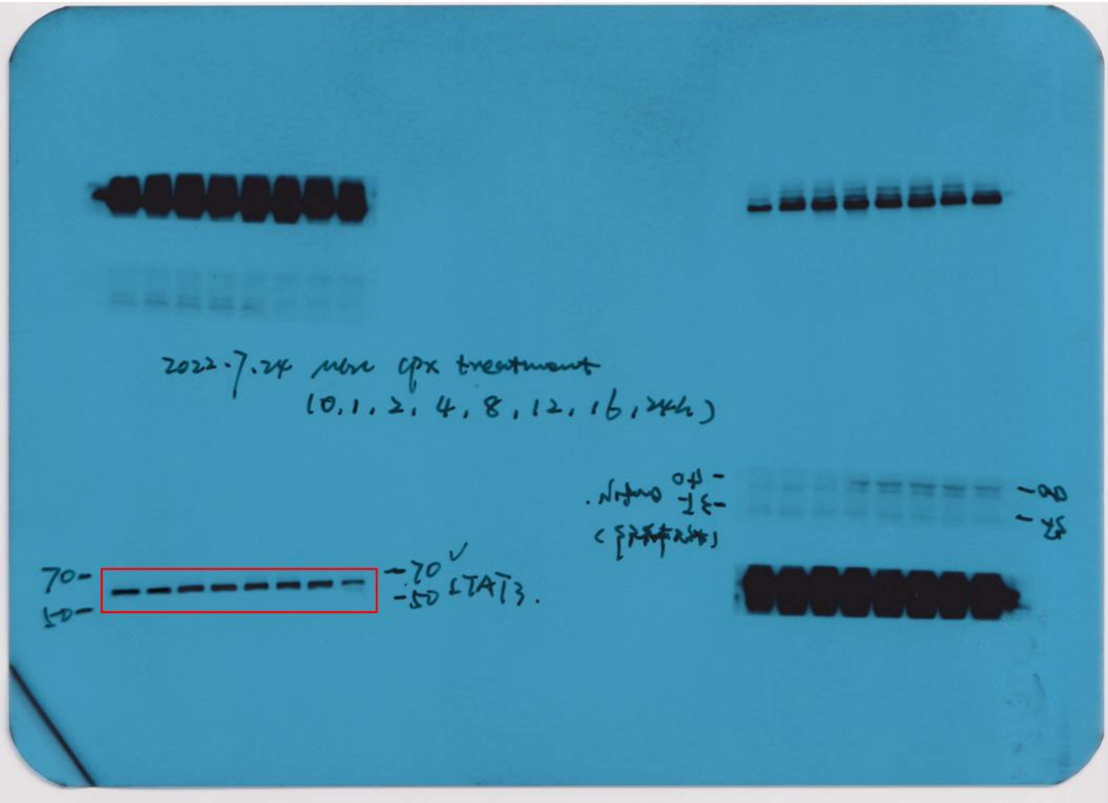

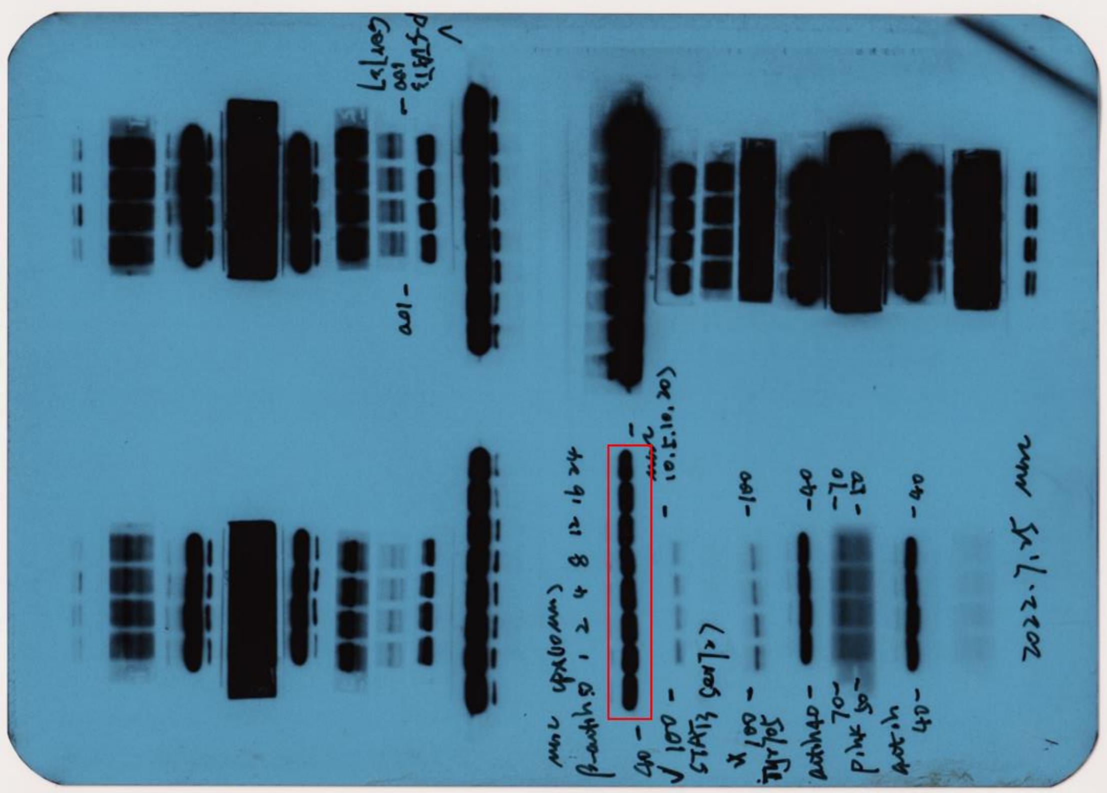

AGS and SGC cells

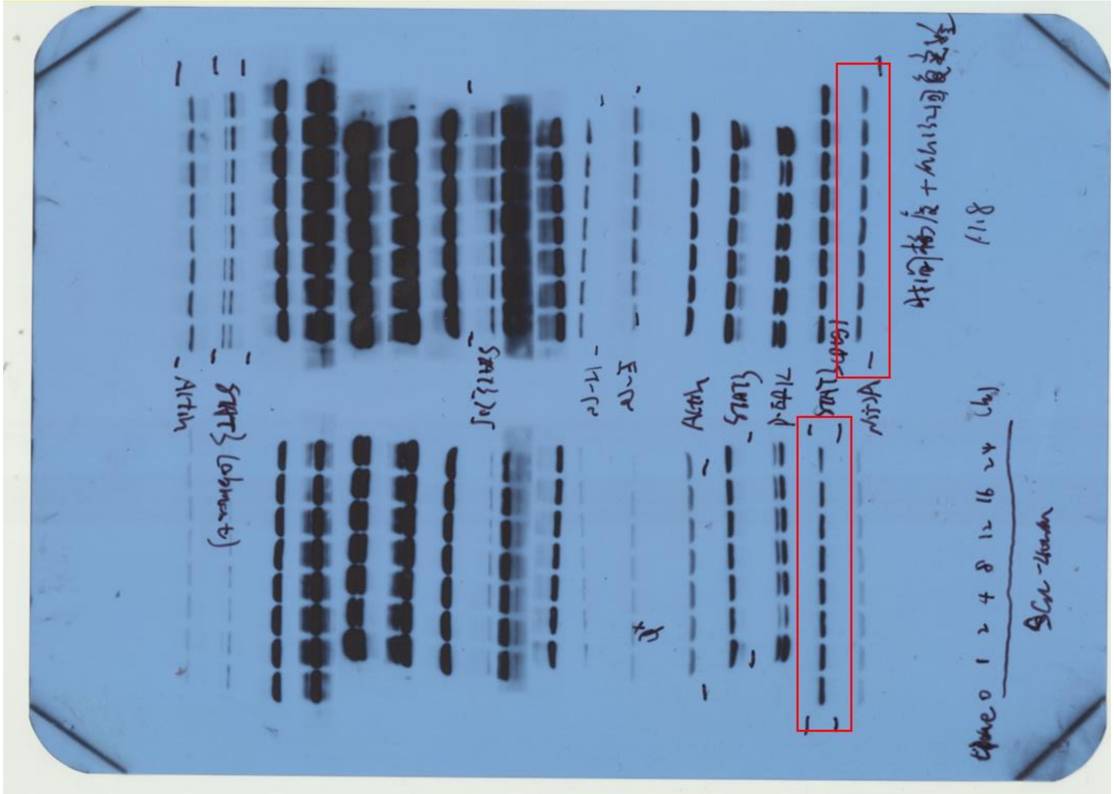

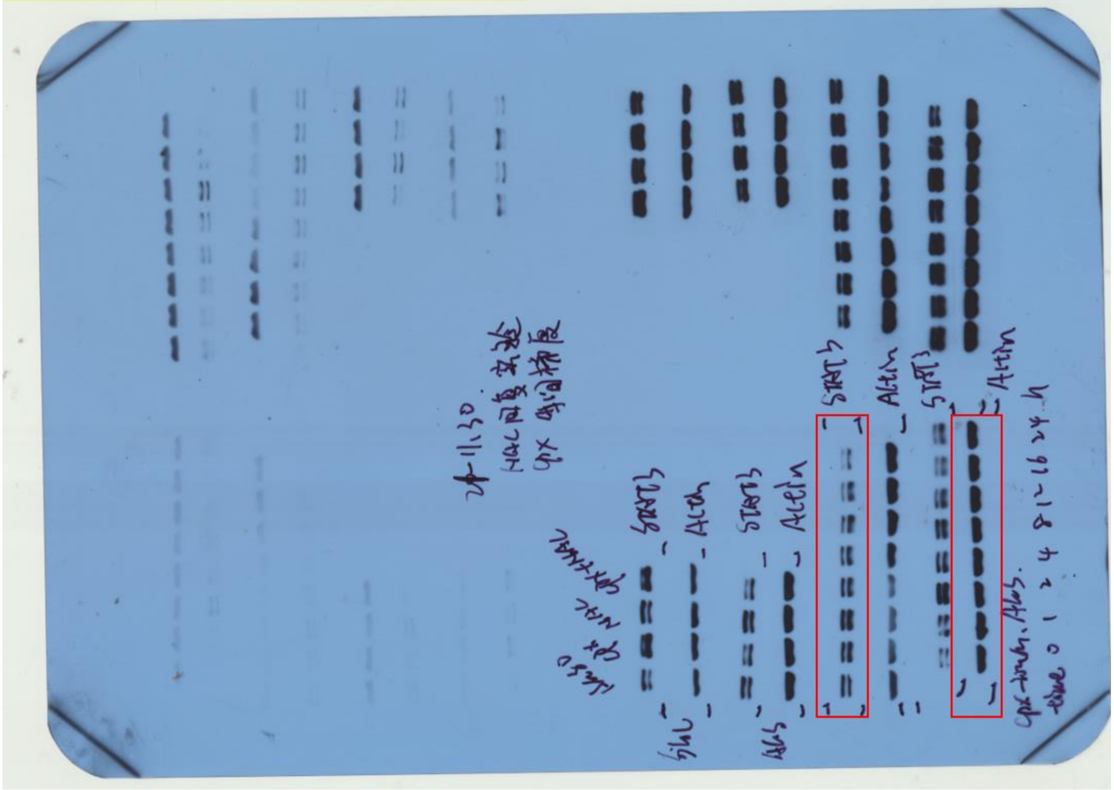

Figure S4b

MGC cells

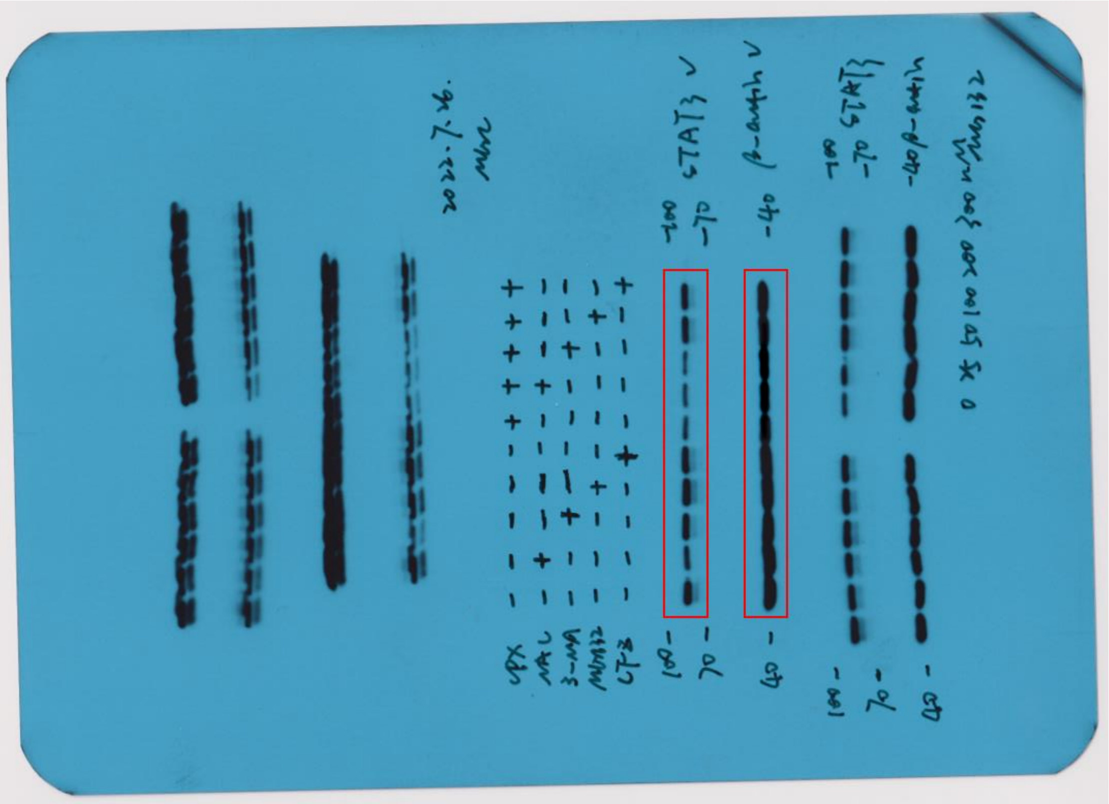

AGS and SGC cells

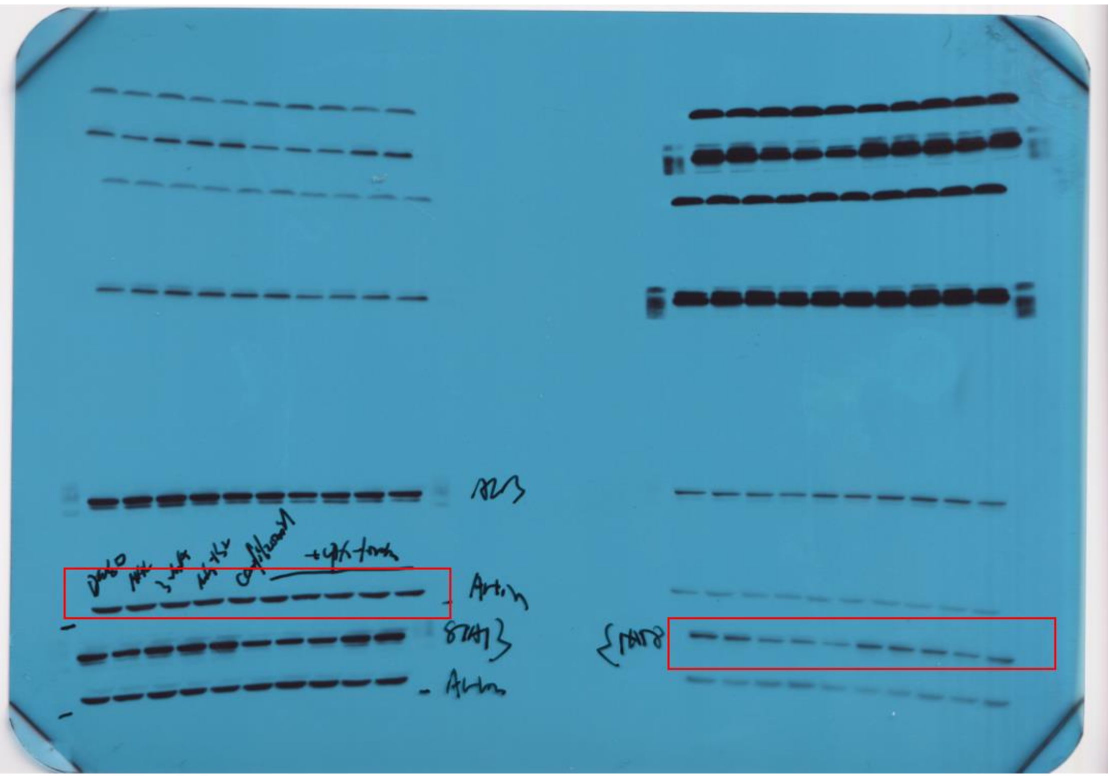

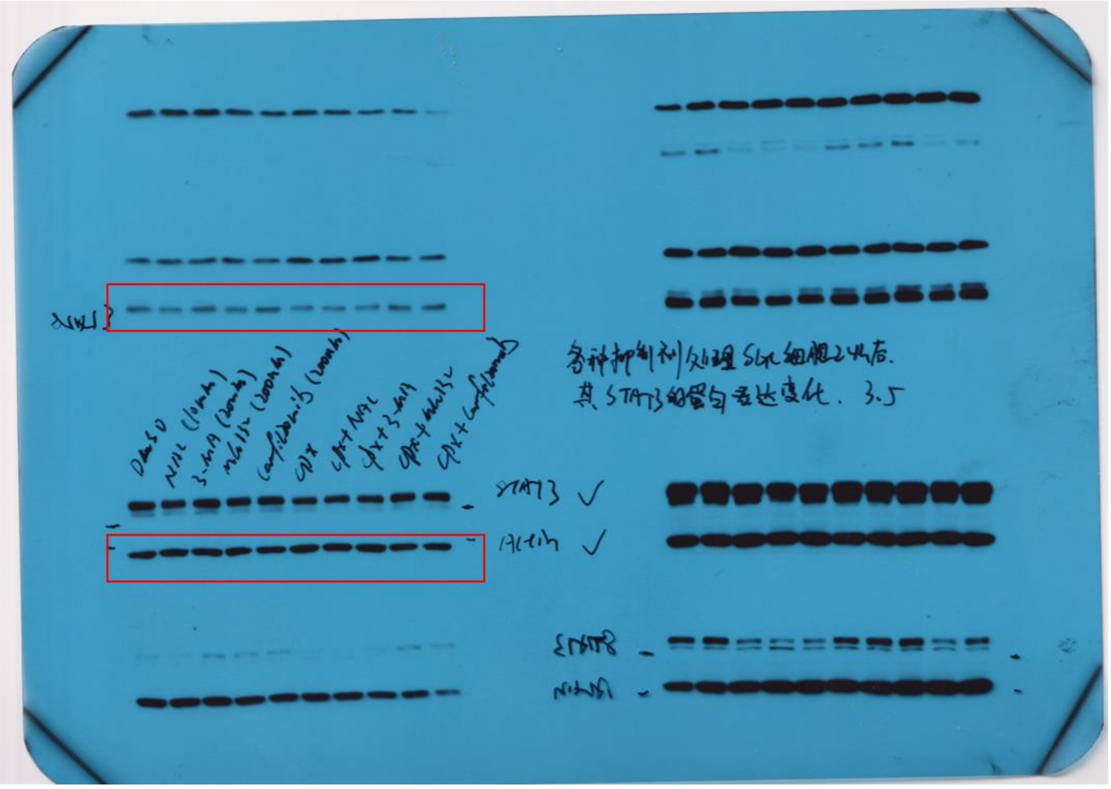

Figure S4c

MGC and AGS cells

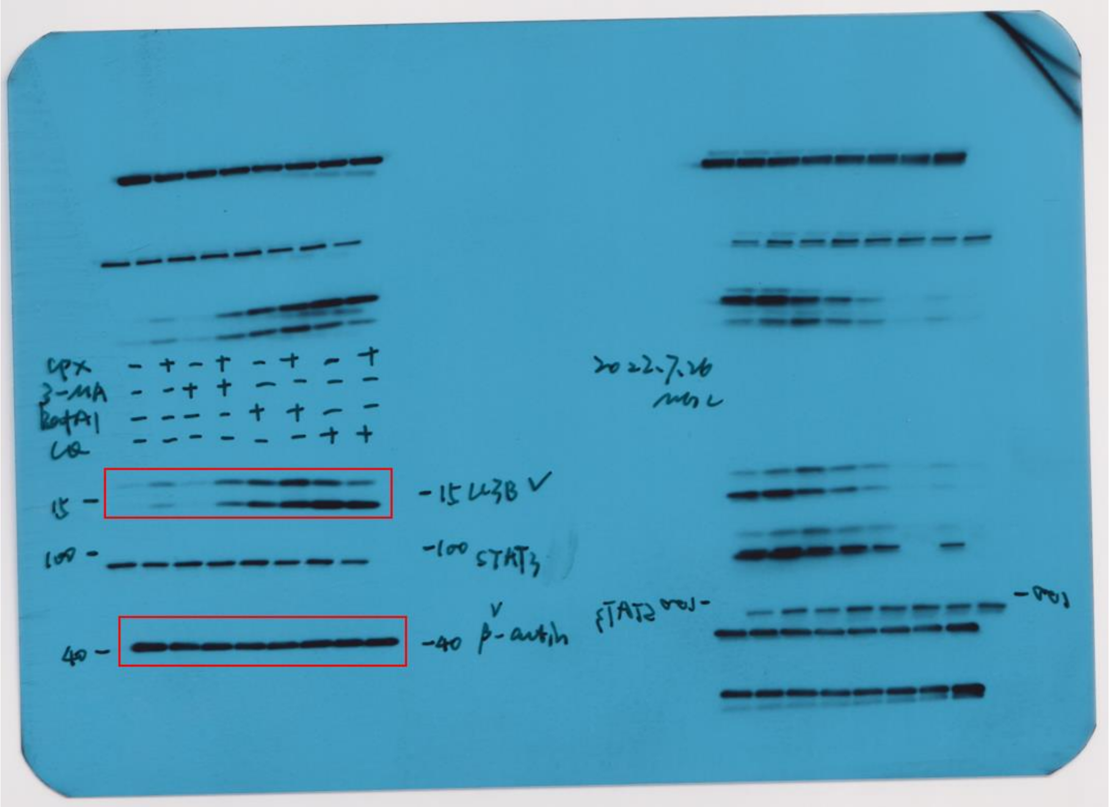

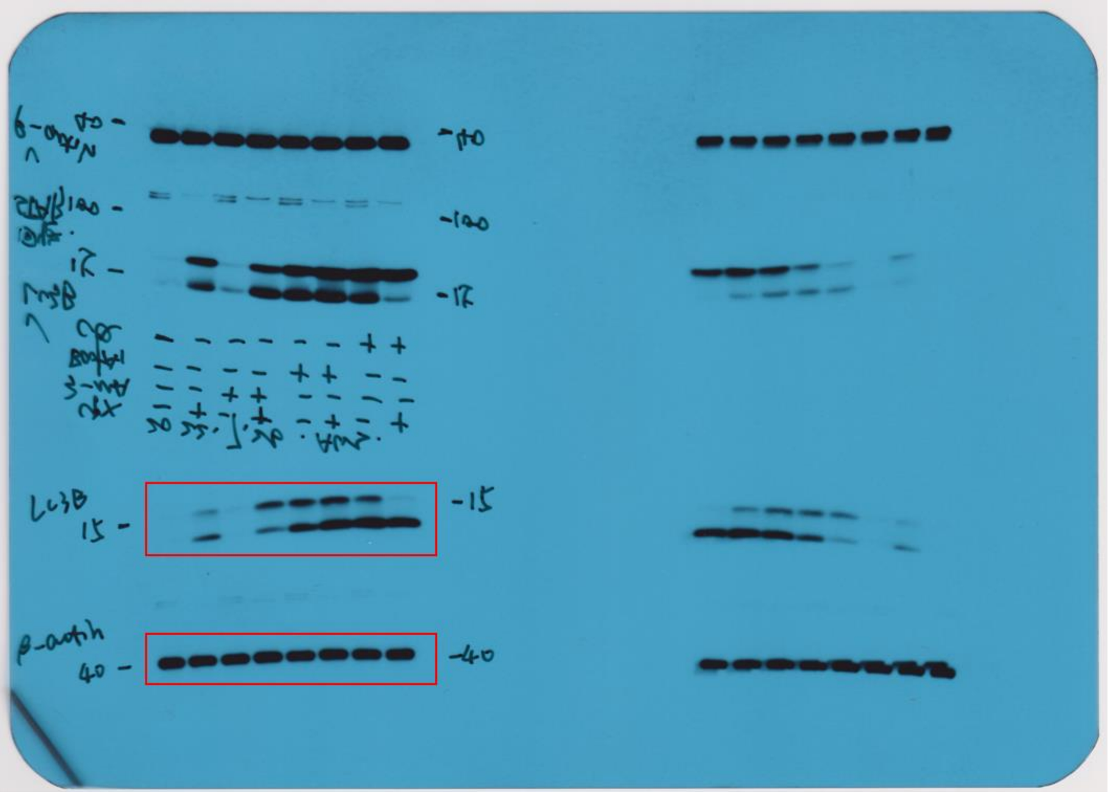
LC3B
15
β-actin
40

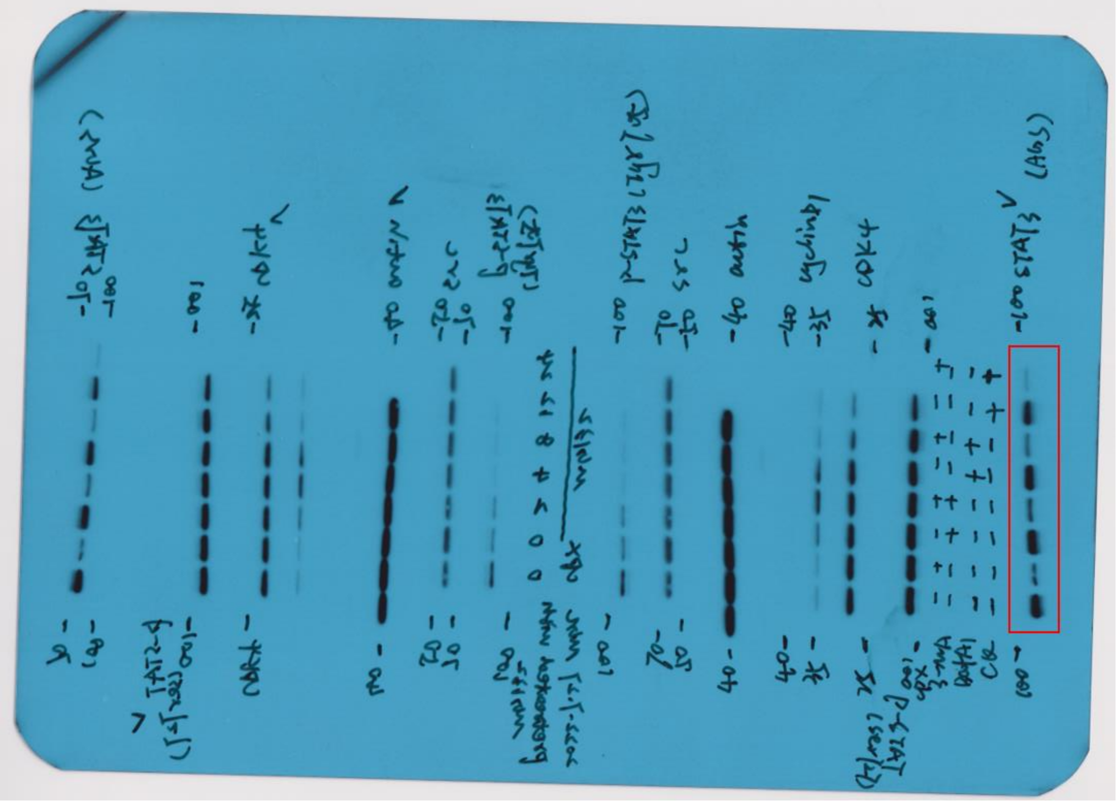

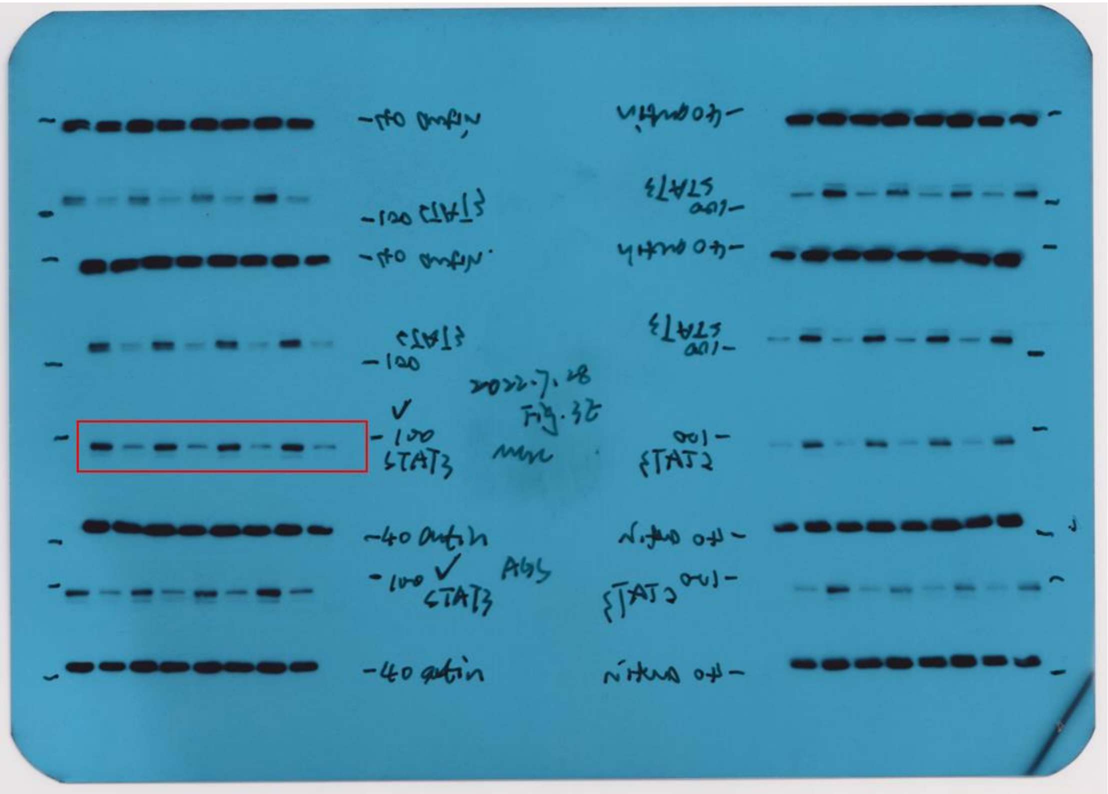

SGC cells

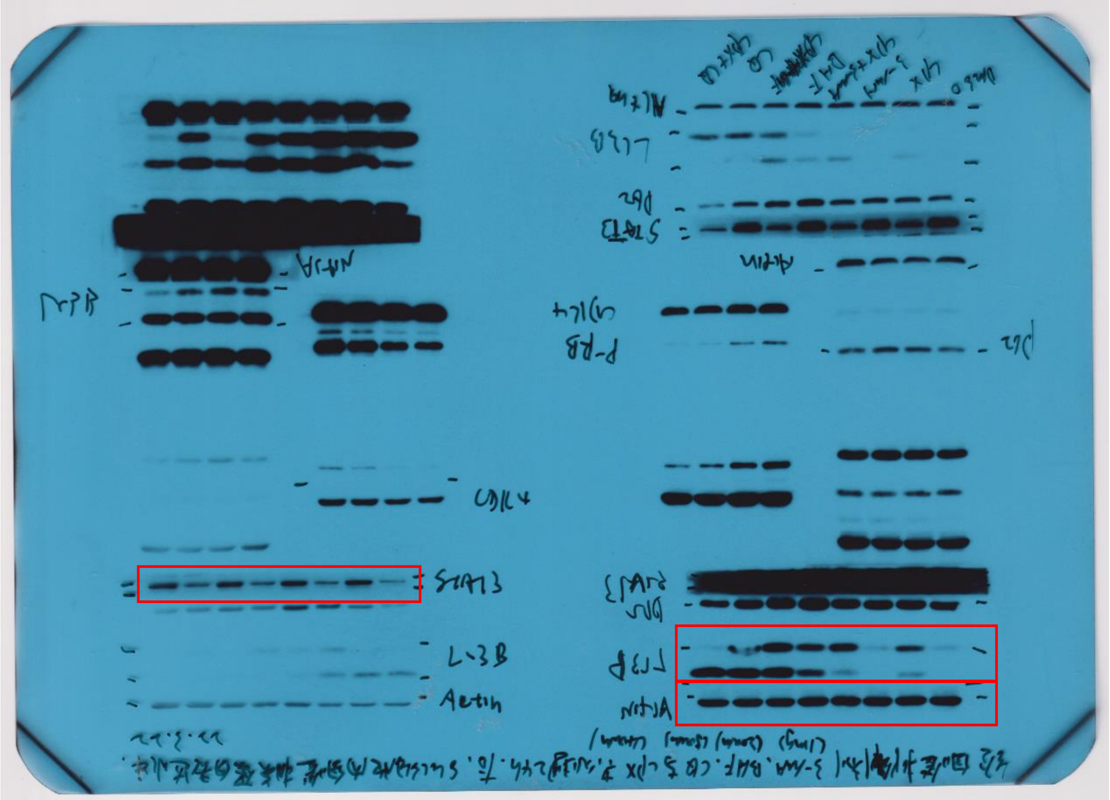

Figure S4d

MGC cells

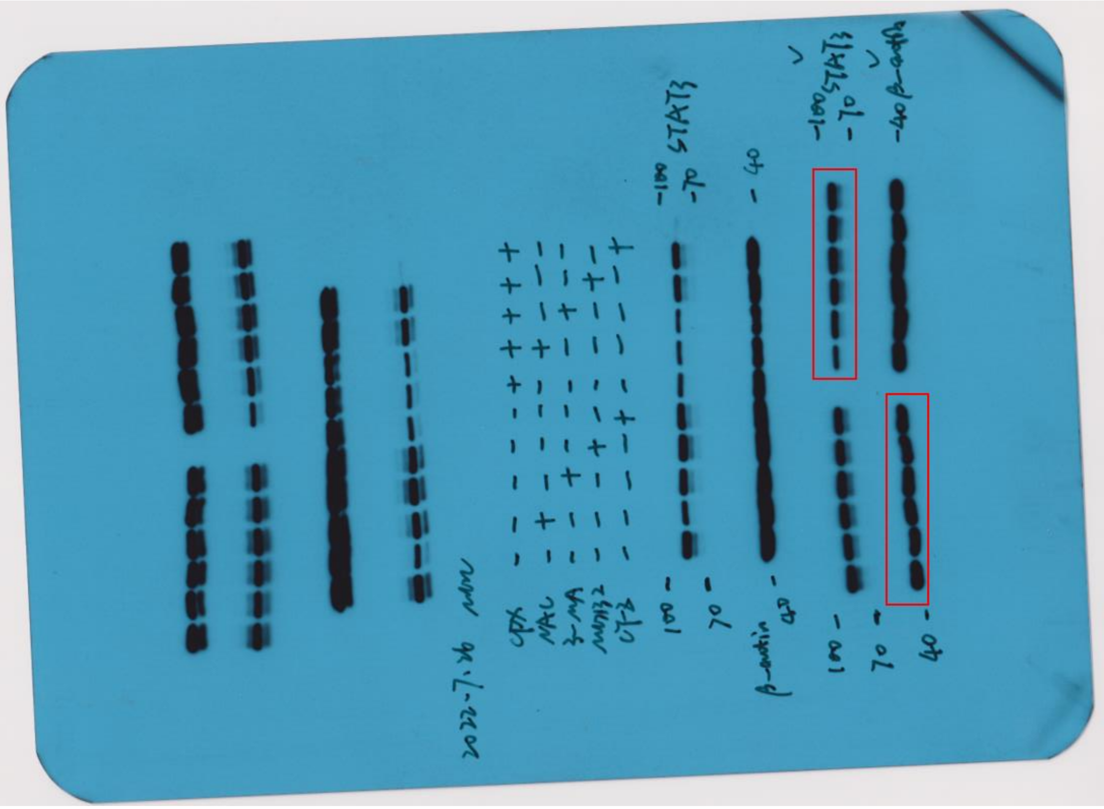

AGS and SGC cells

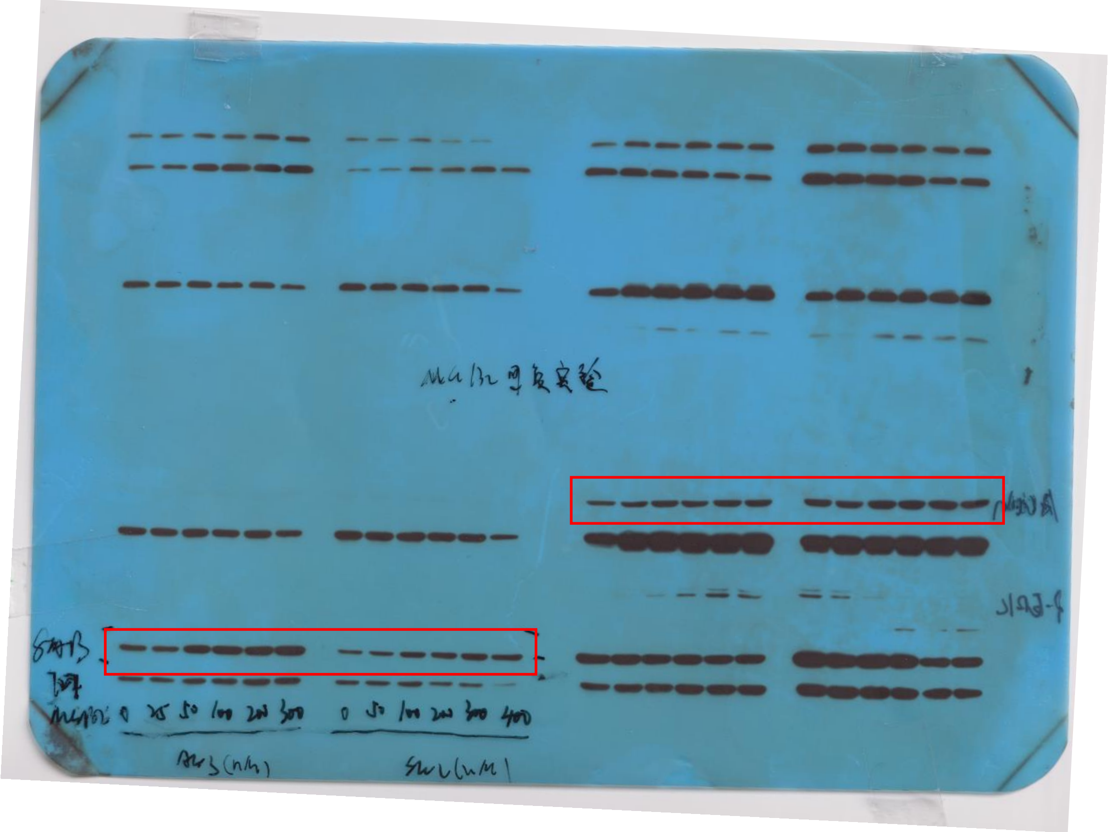

Figure S5a

MGC cells

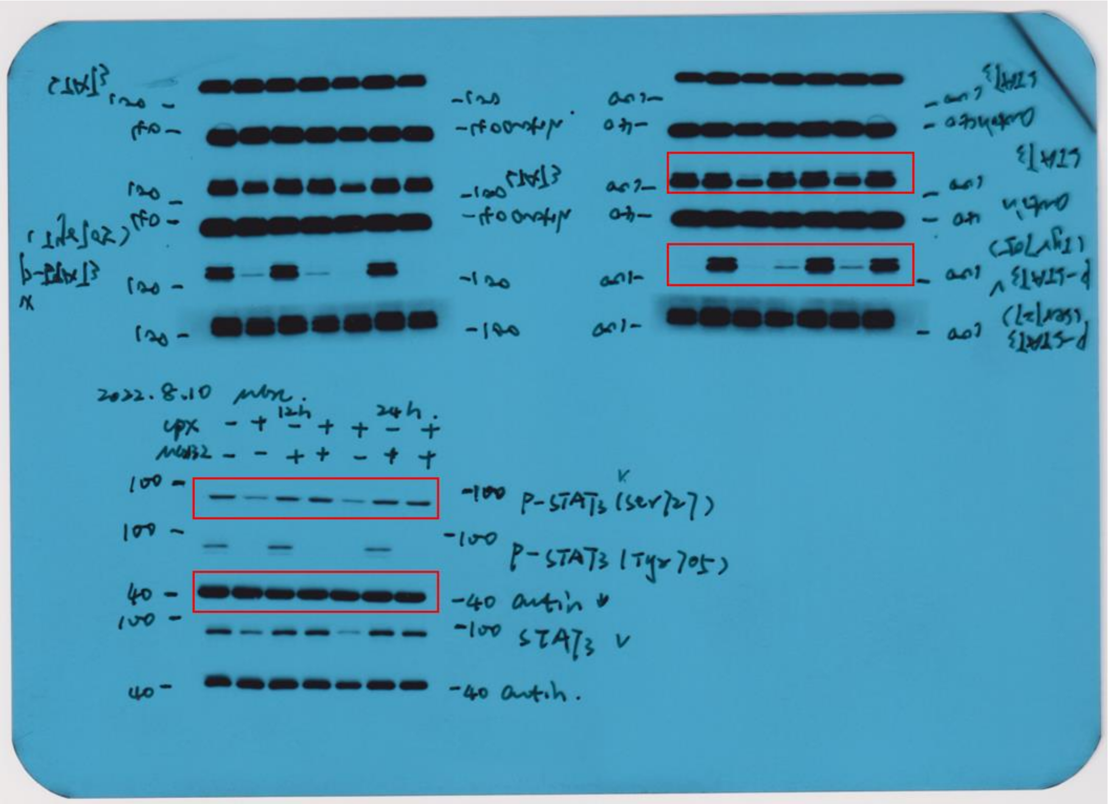

AGS cells

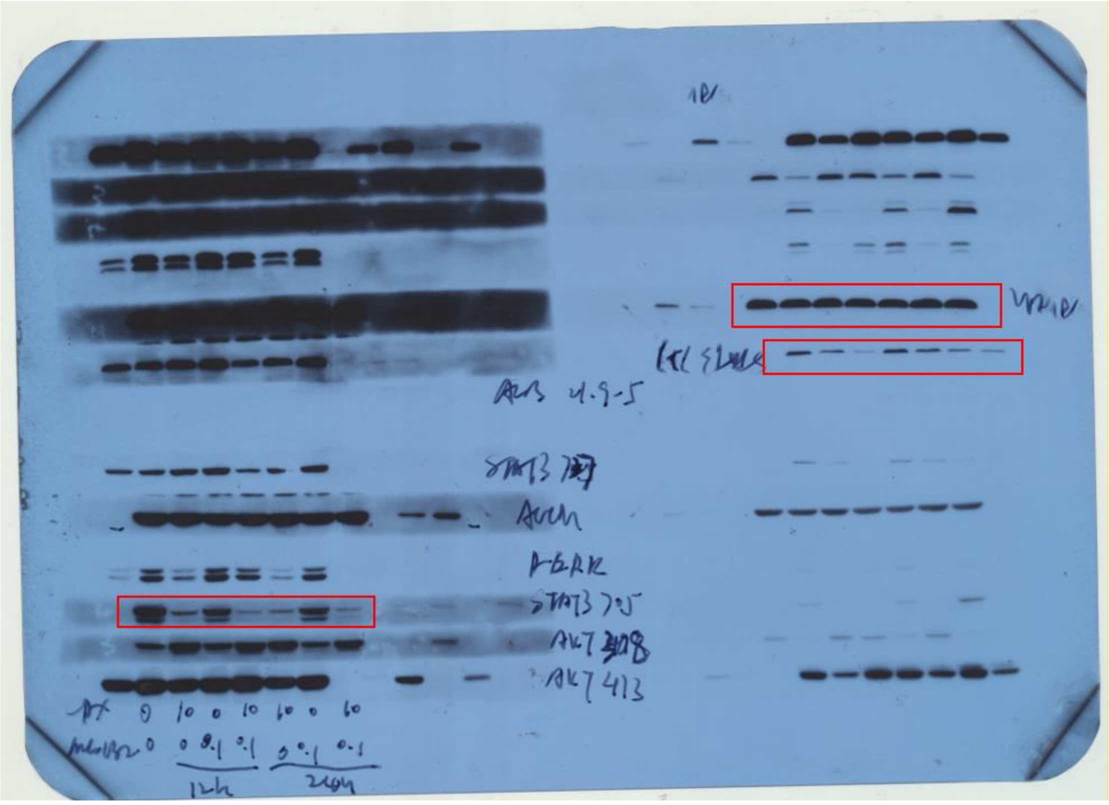

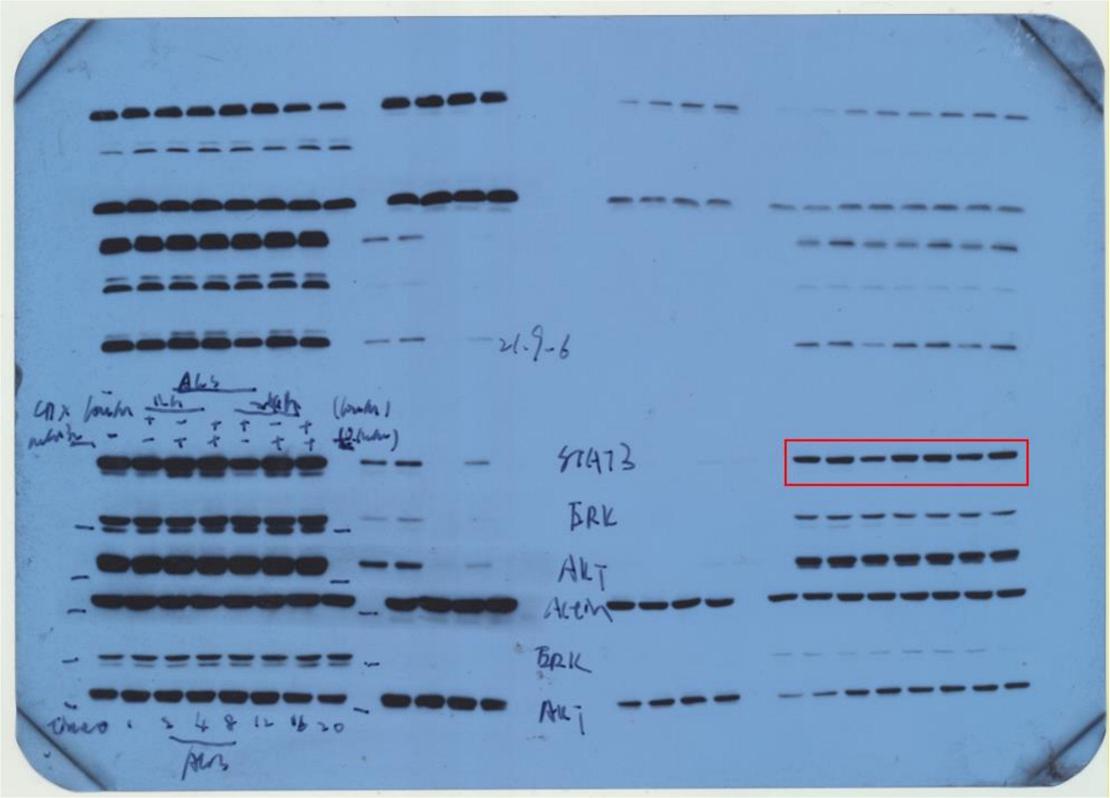

SGC cells

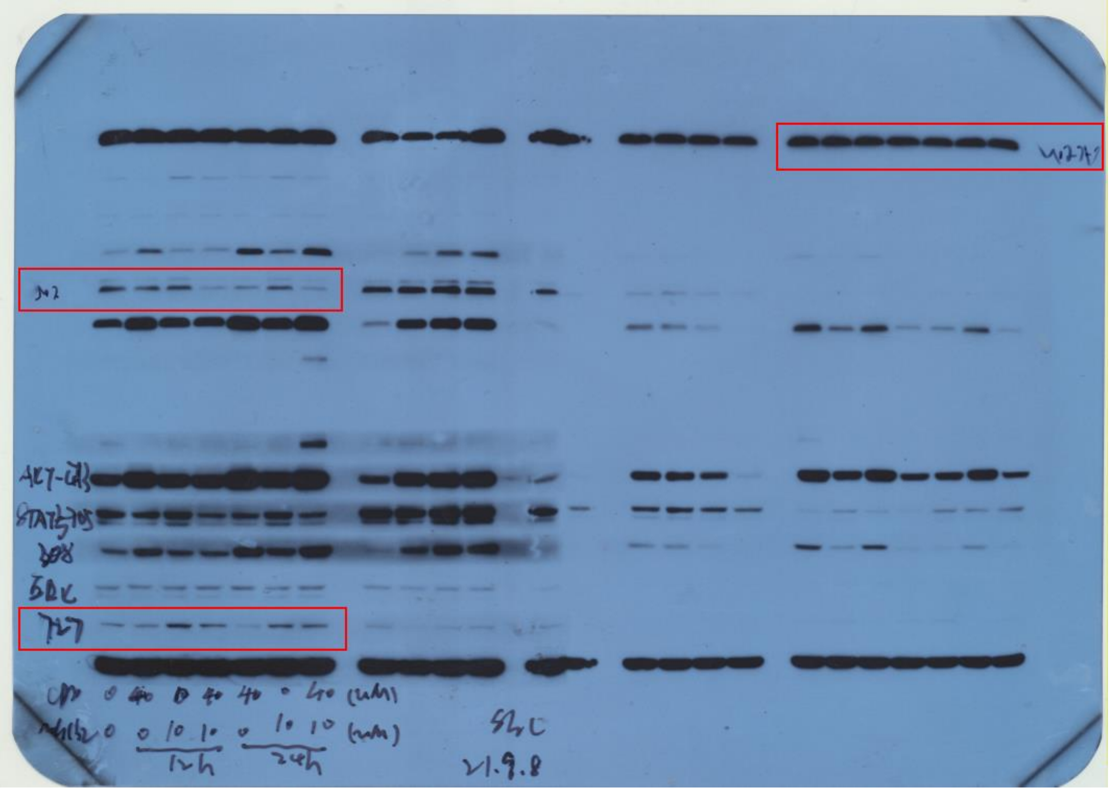

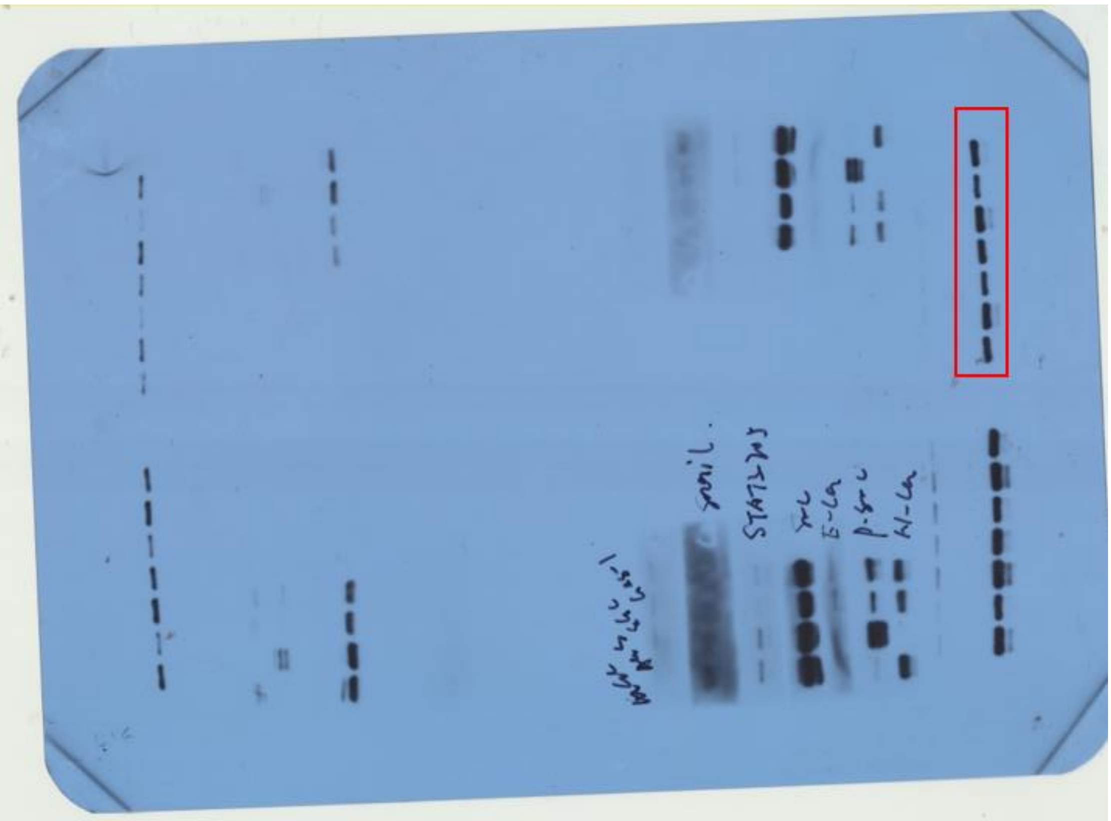

Figure S5b

AGS cells

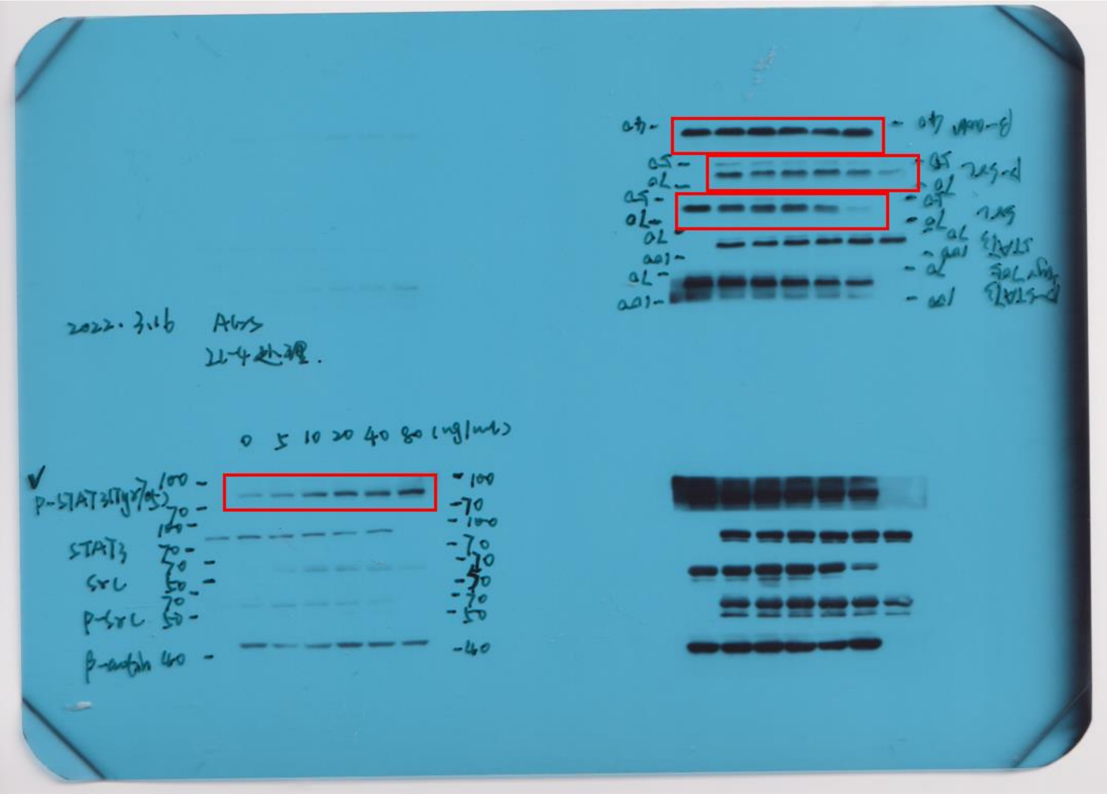

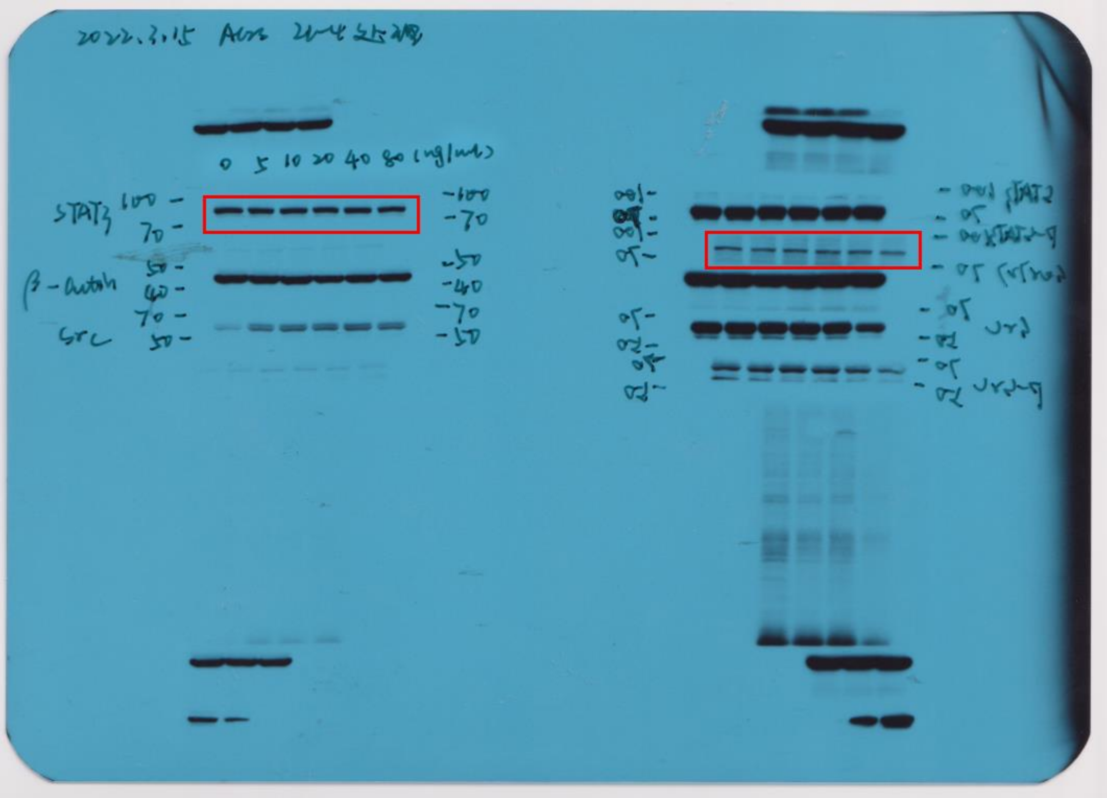

MGC and SGC cells

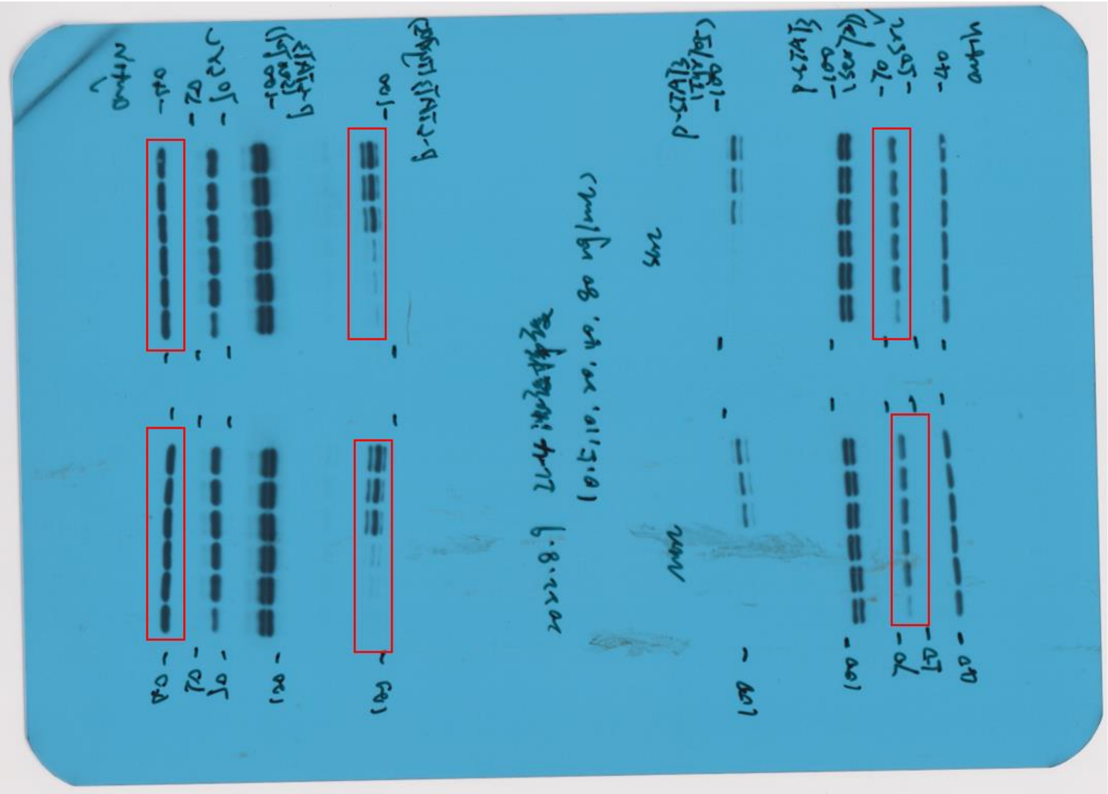

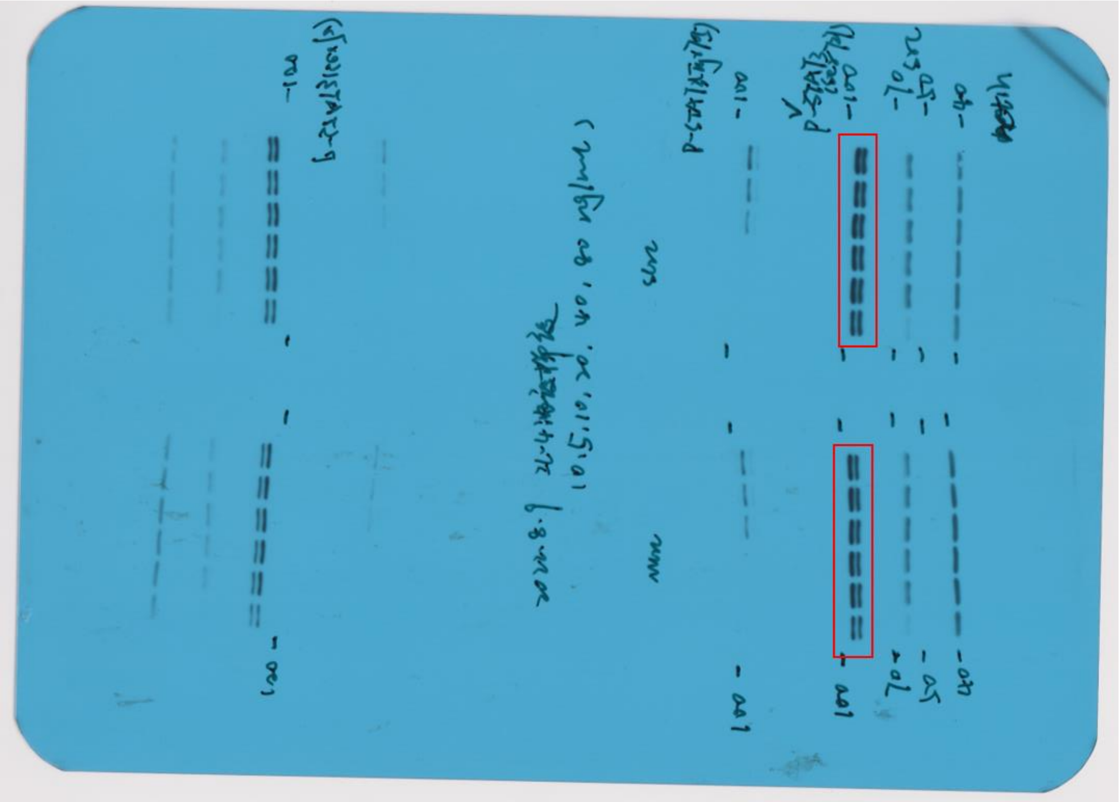

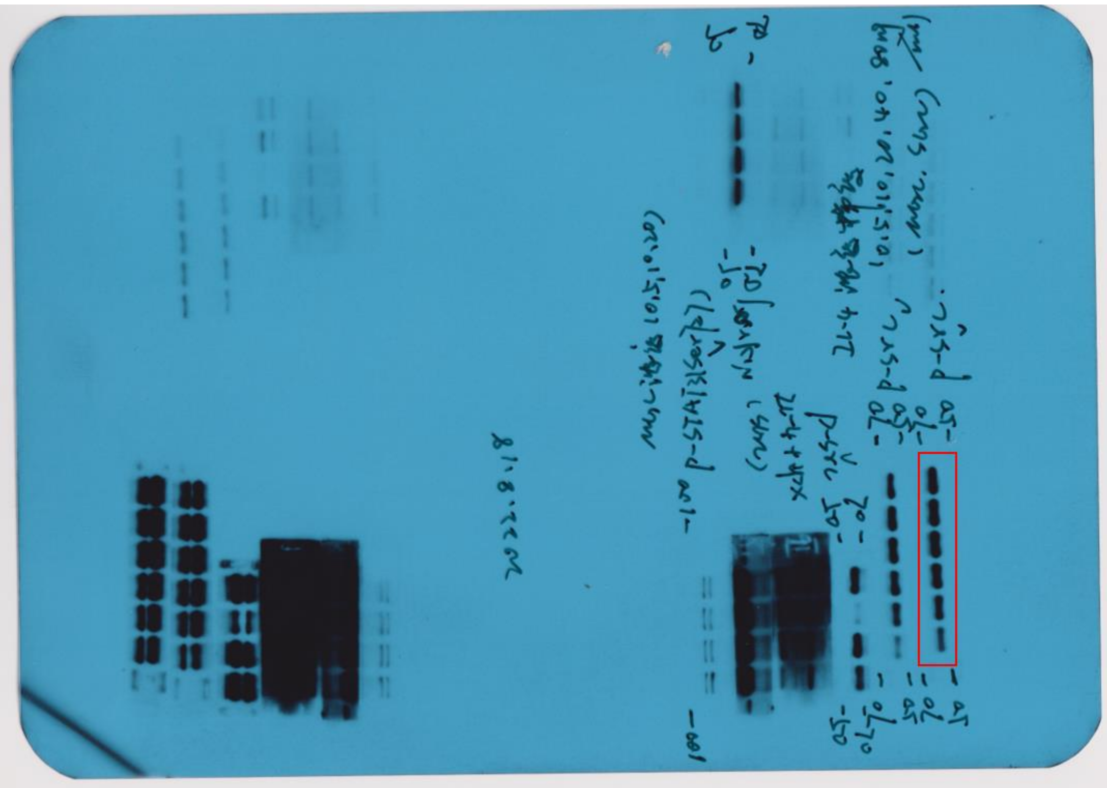

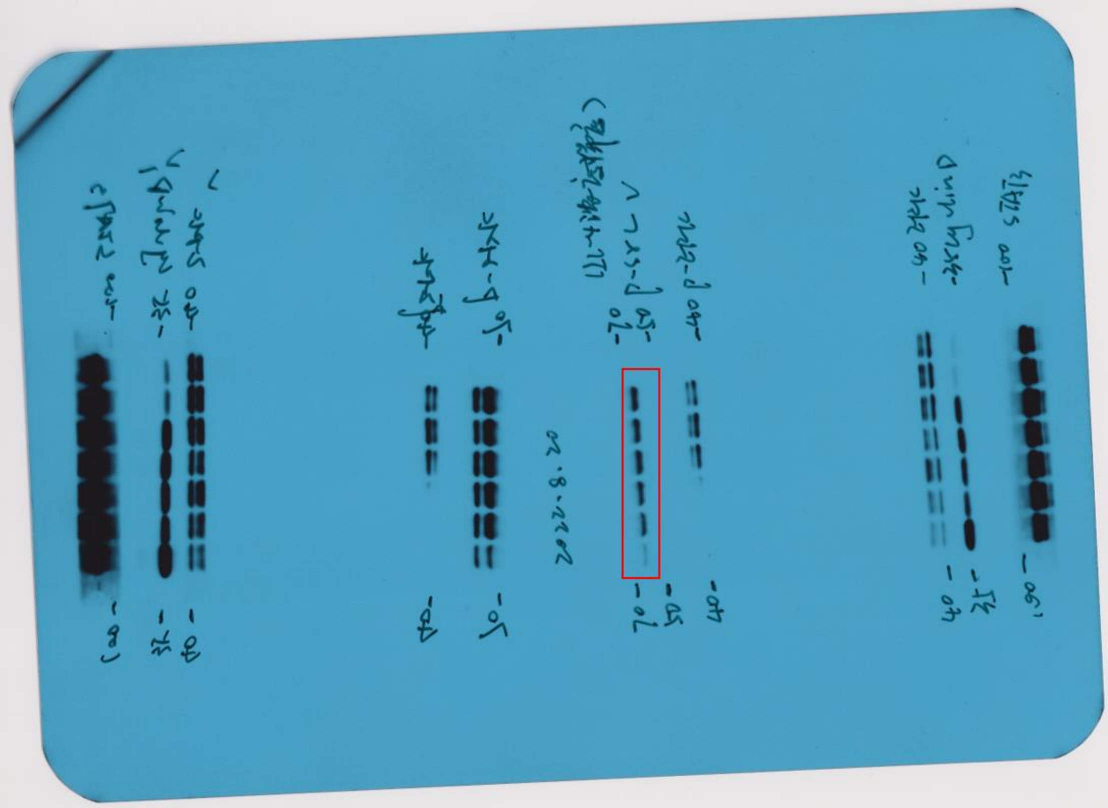

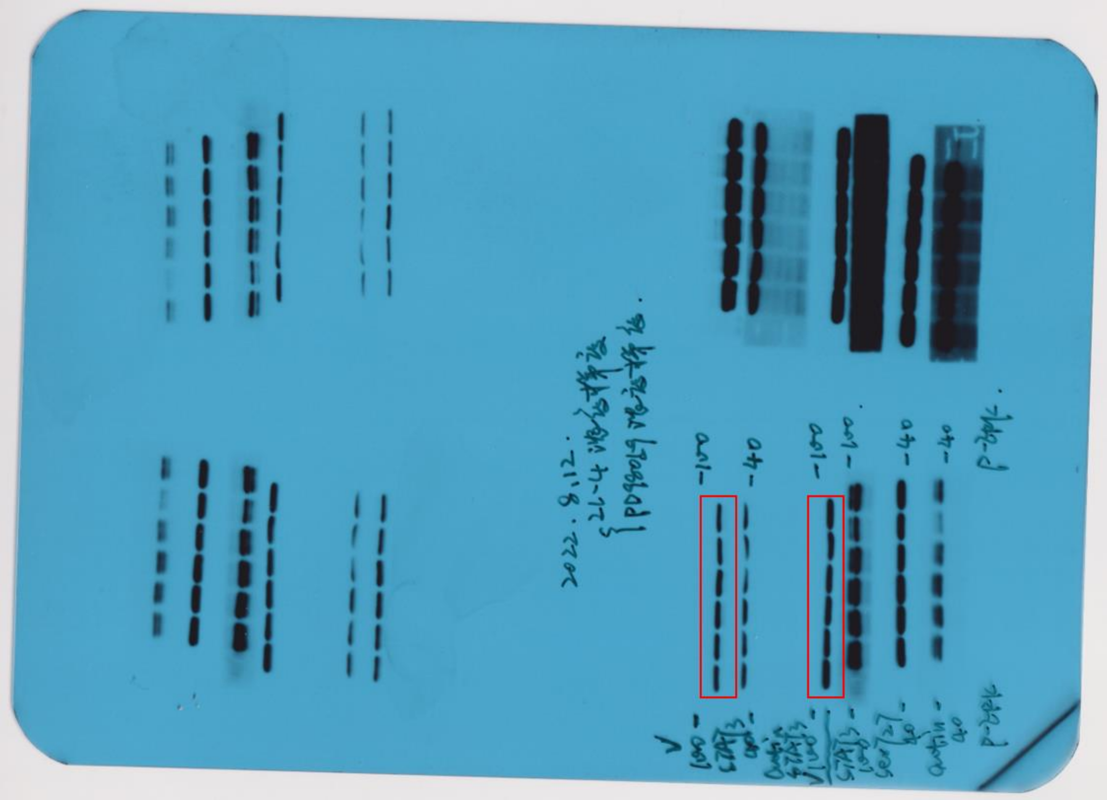

Figure S5c

AGS cells

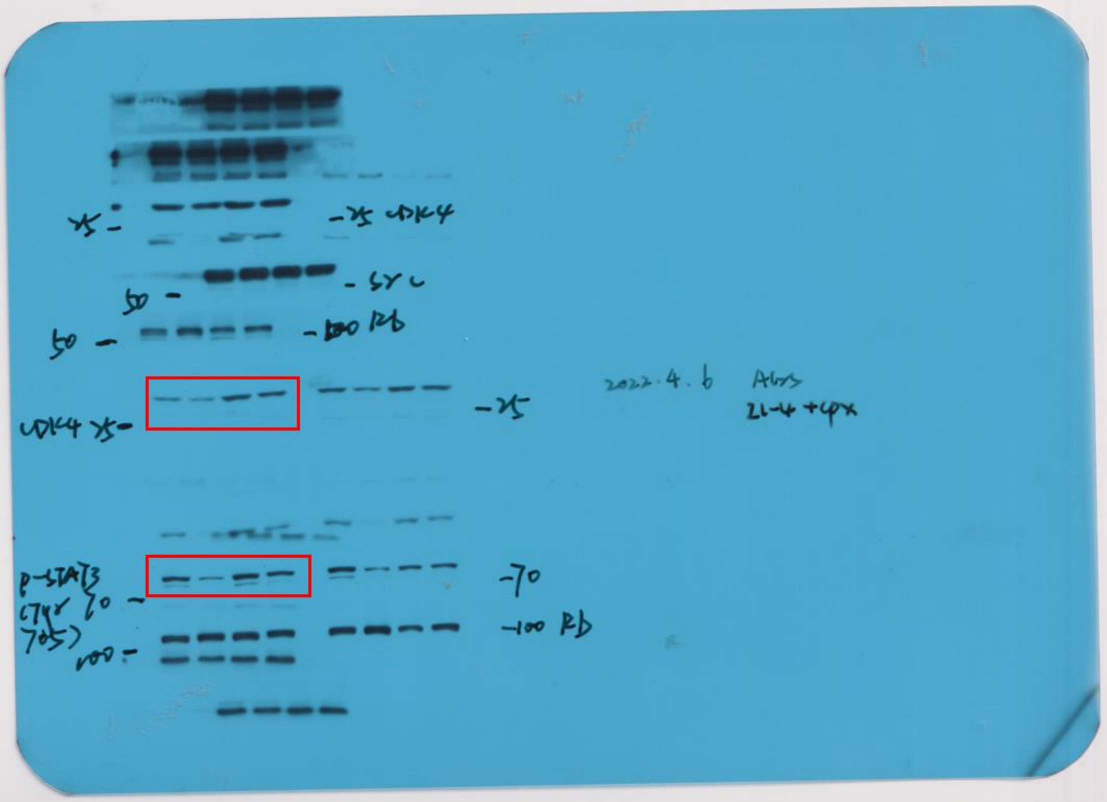

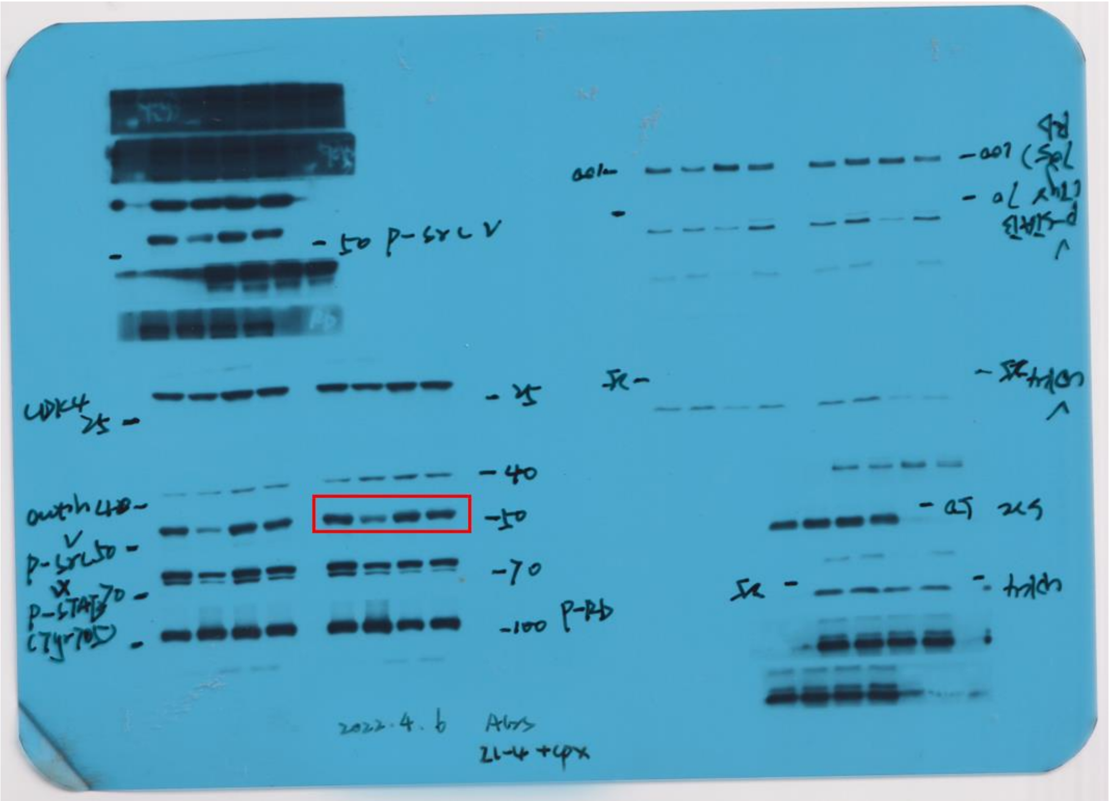
CDK4
25
-25
-40
-50
-70
-100 P-Rb
-50 P-src
2022.4.6
21-4 +cpx

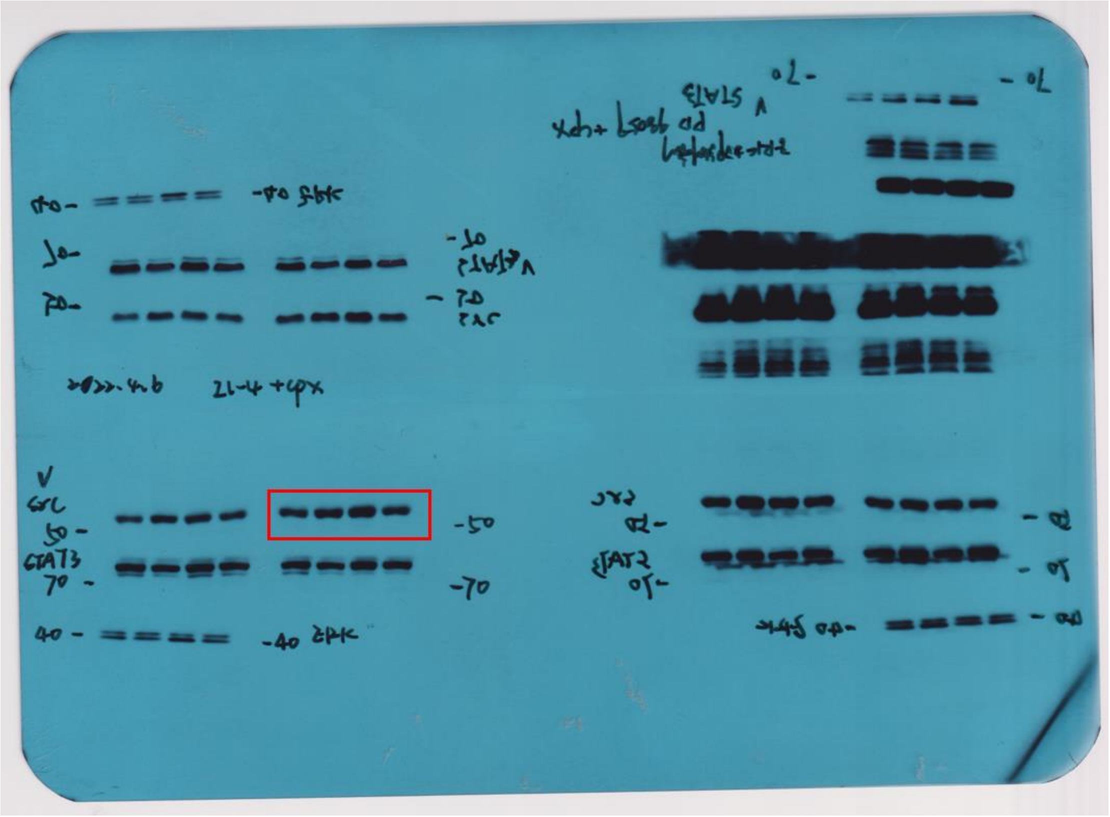
src
50
STAT3
70
40
-40 ERK
-50
-70
2022.4.6
21-4 +cpx

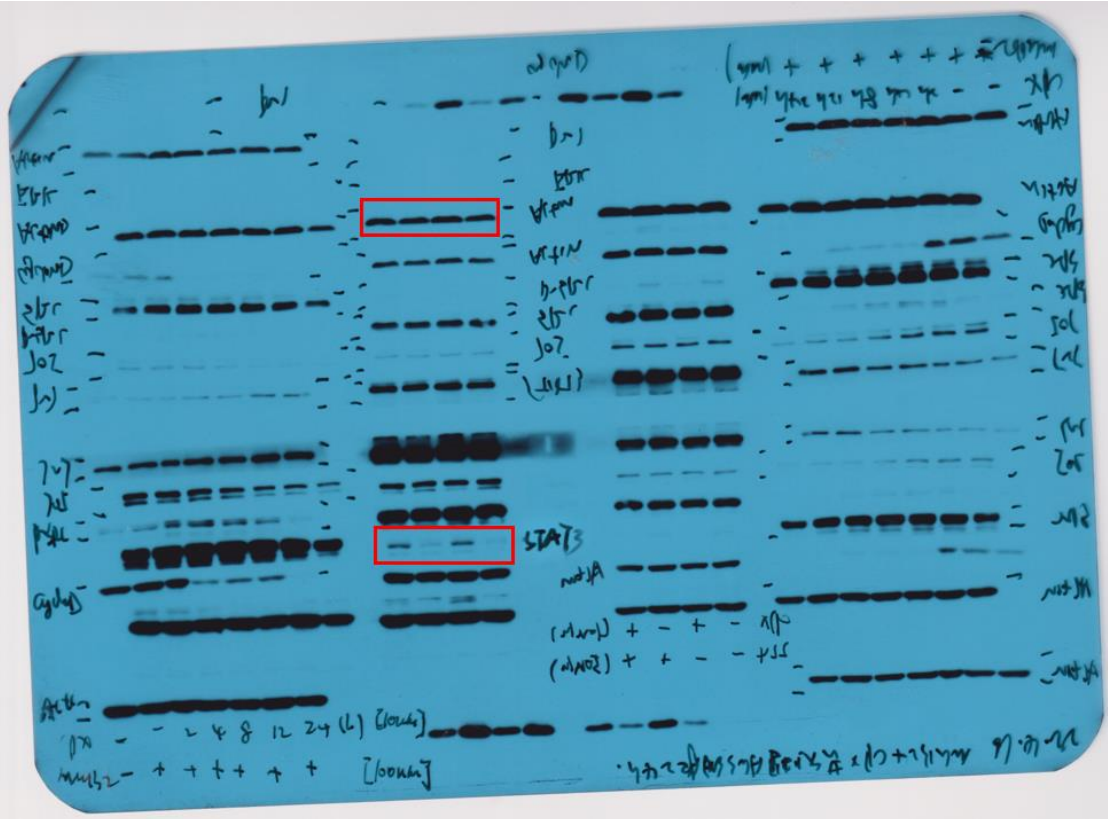

MGC and SGC cells

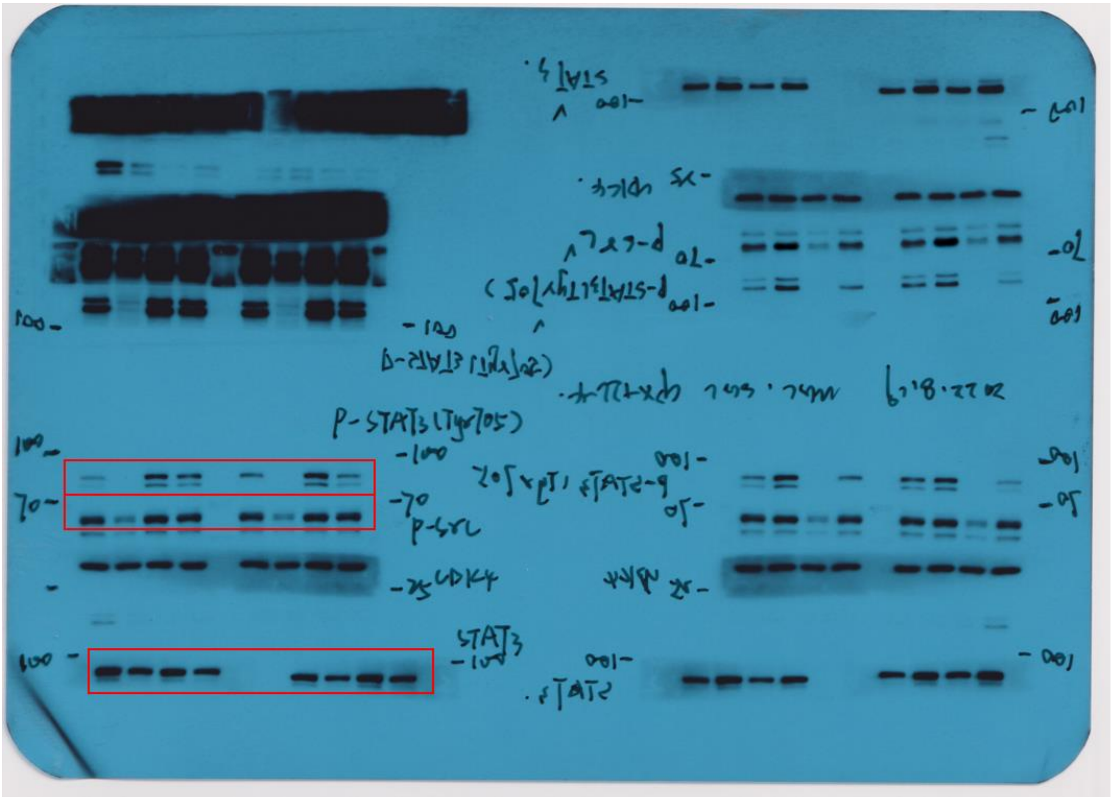

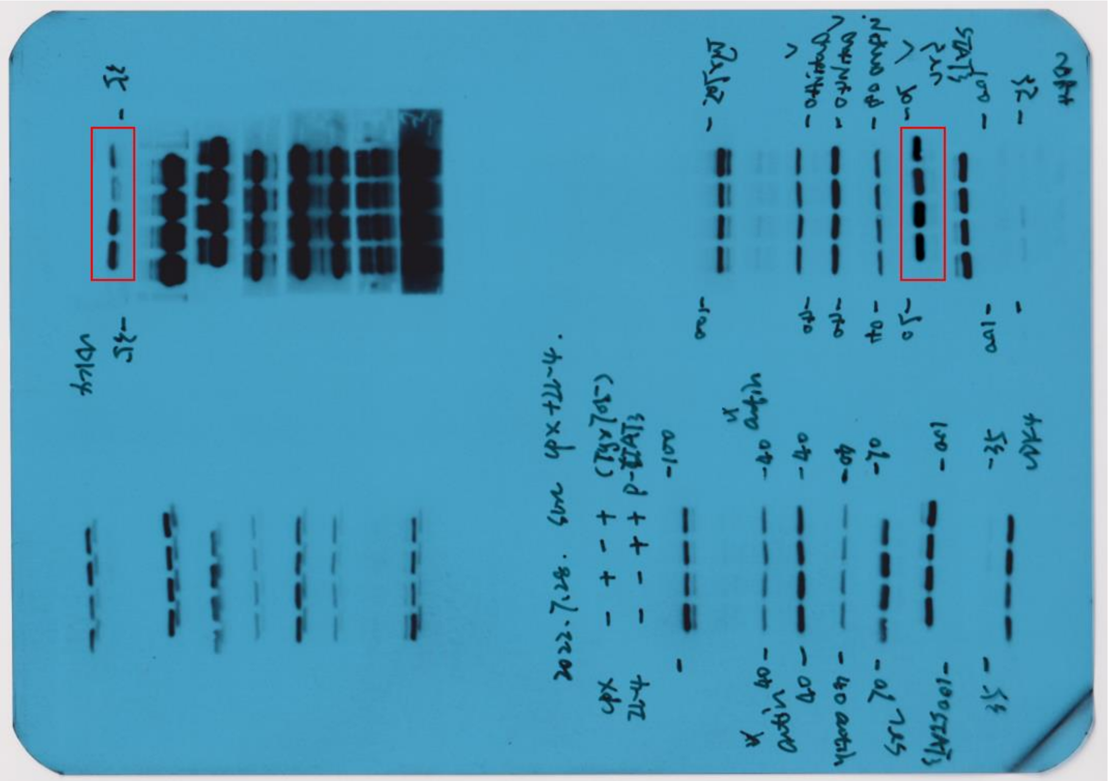

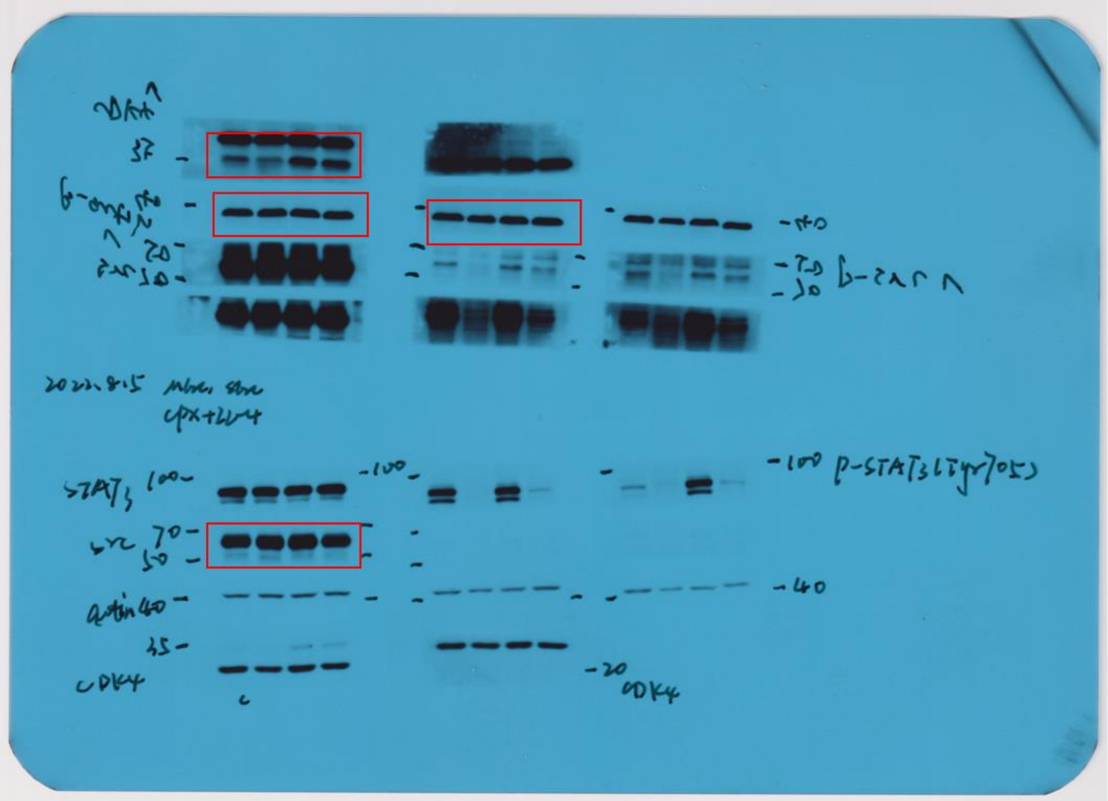
-100 p-STAT3(Tyr705)
STAT3 100
actin 40
-40
CDK4

Figure S6a

MGC and SGC cells

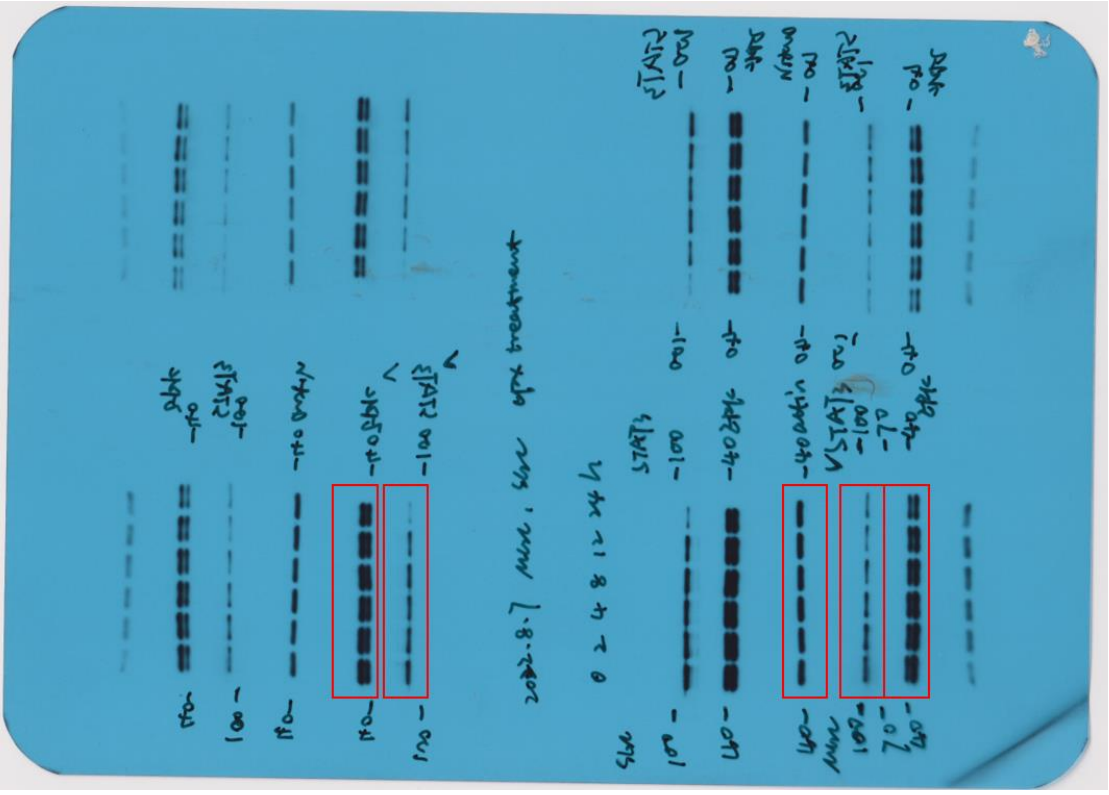

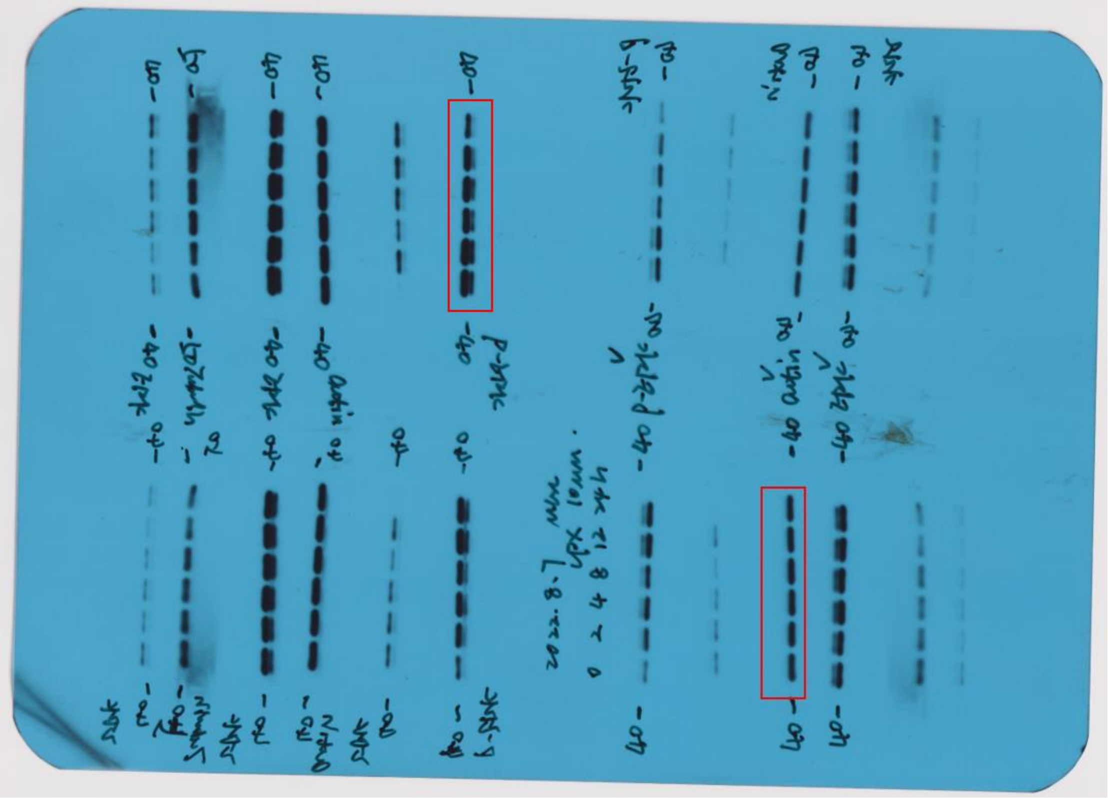

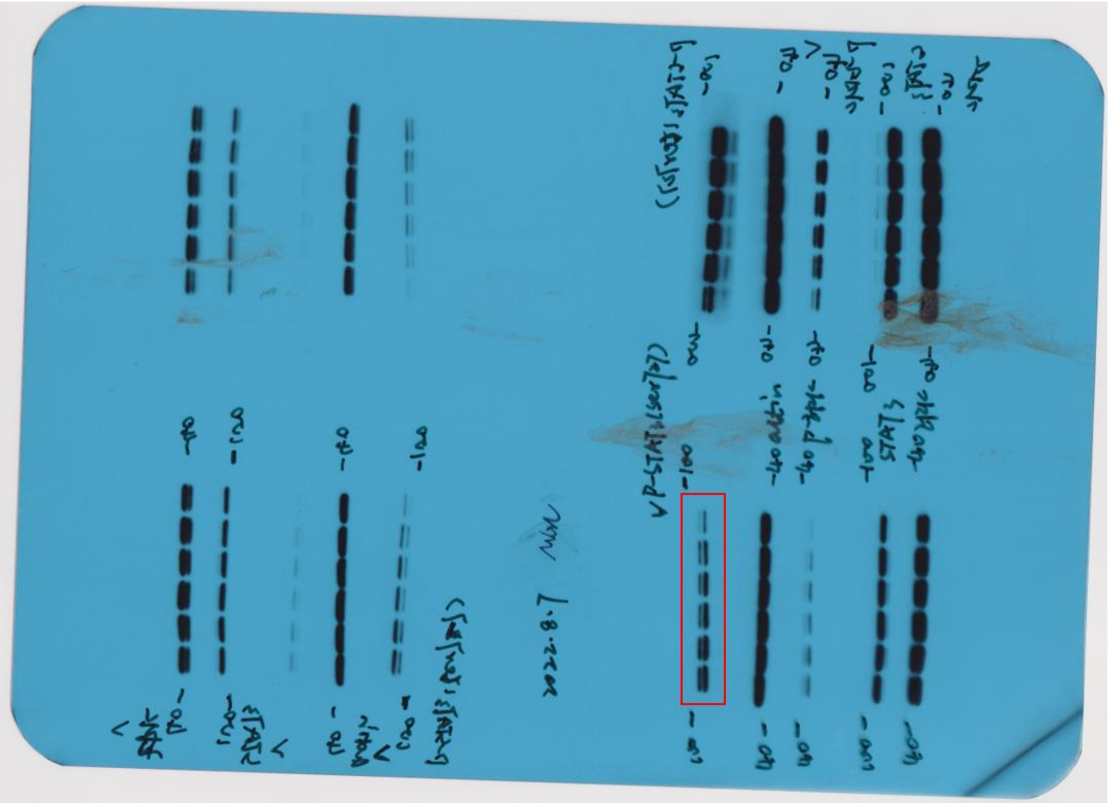

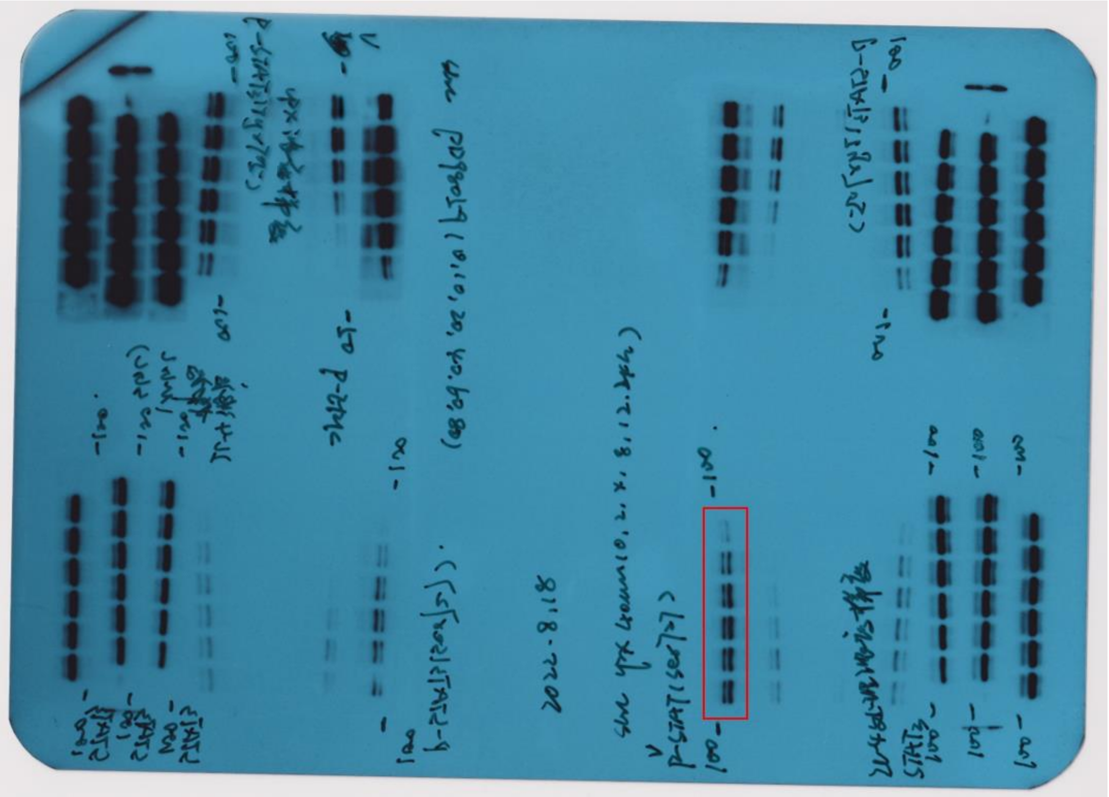

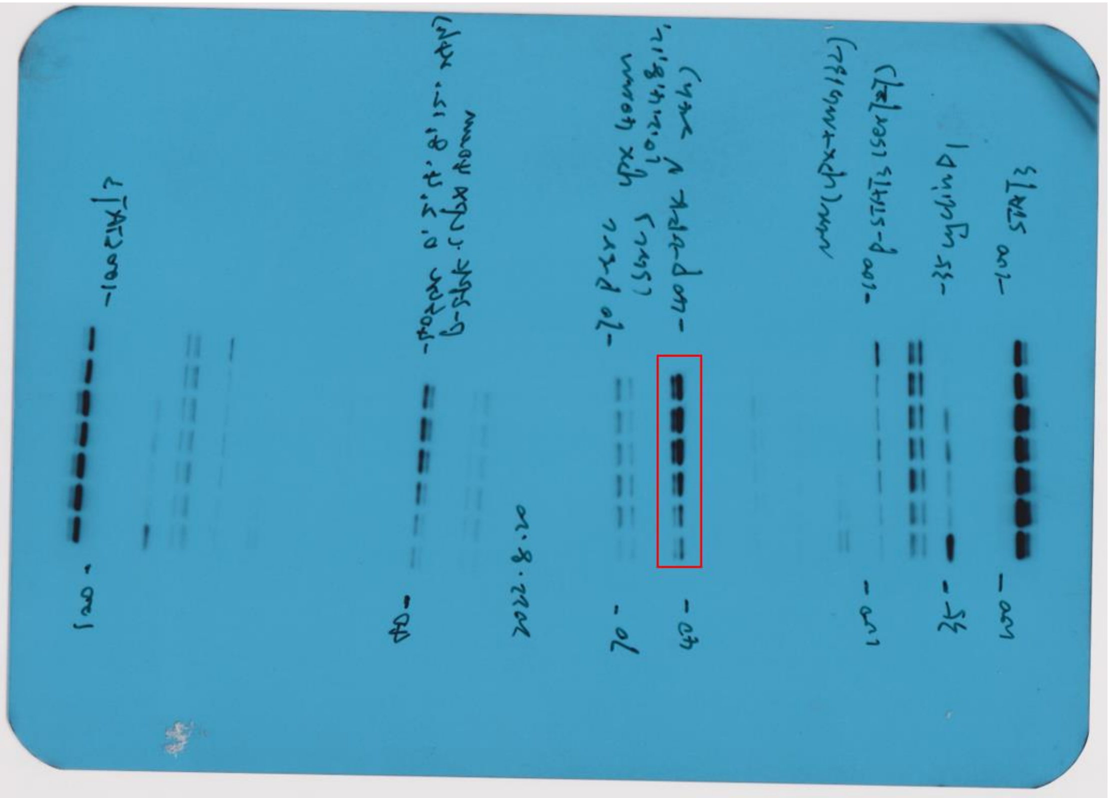

AGS cells

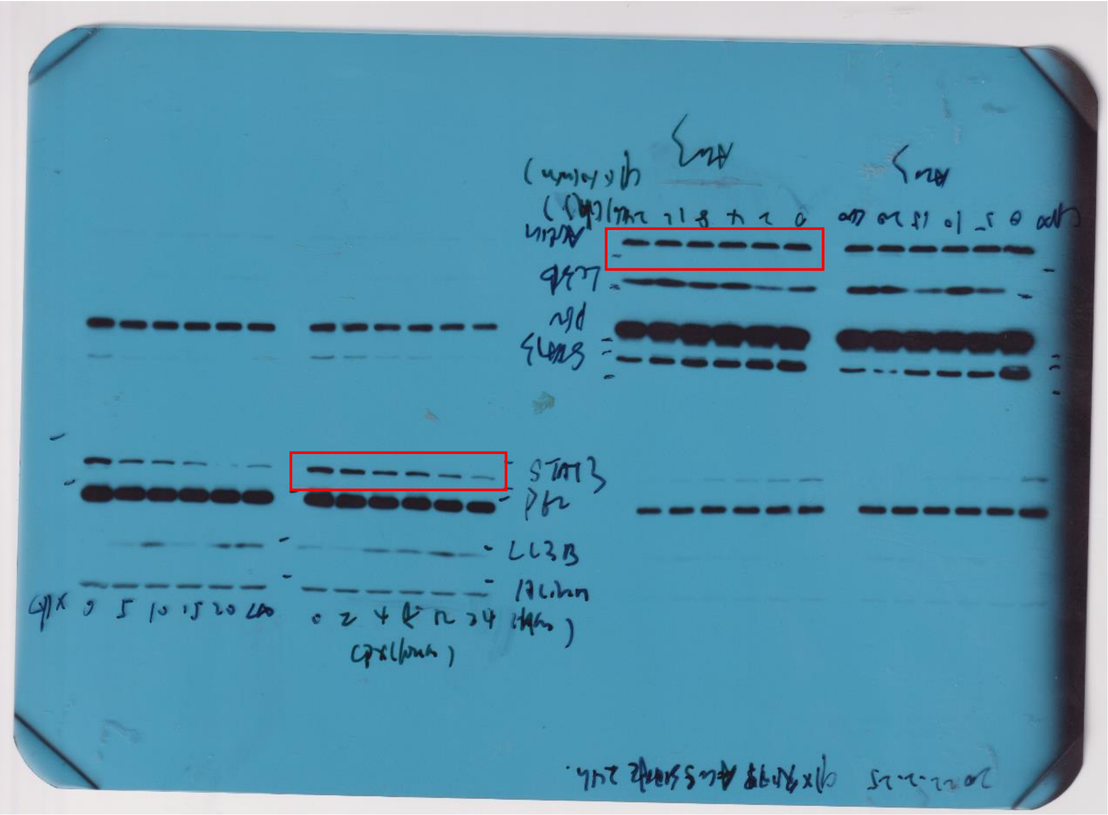

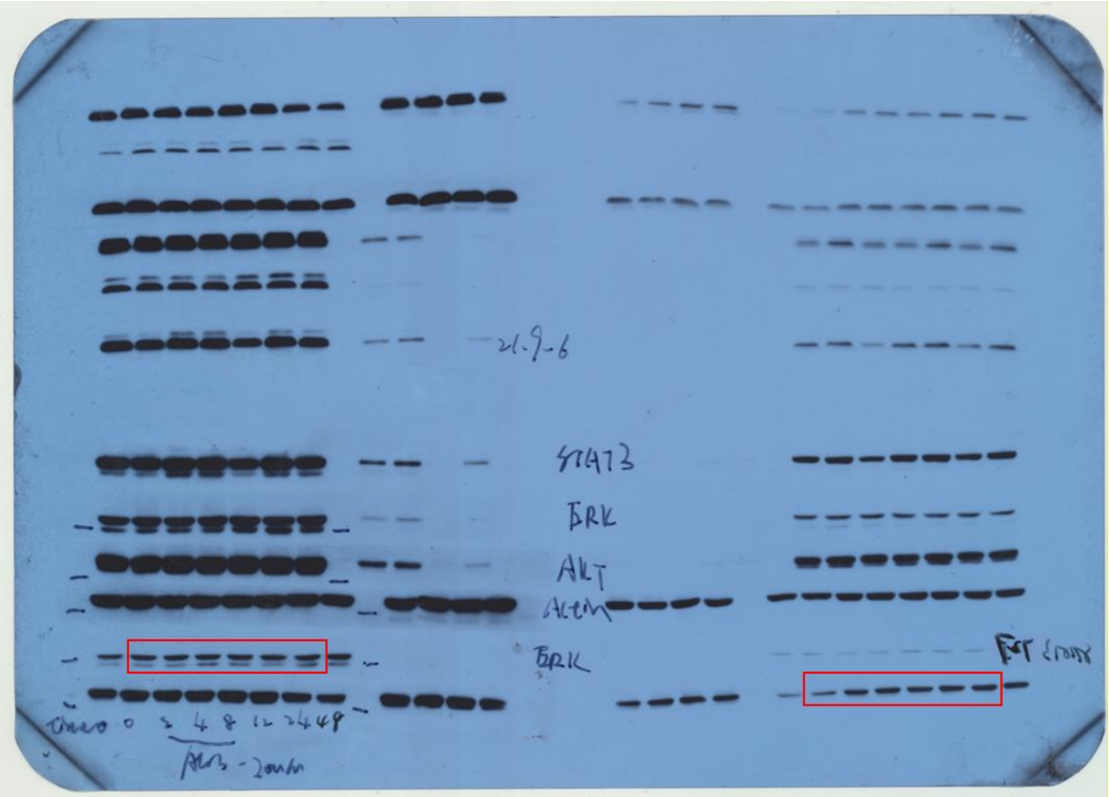
21.9.6
STAT3
ERK
AKT
Actin
ERK
0 2 4 8 12 24 48

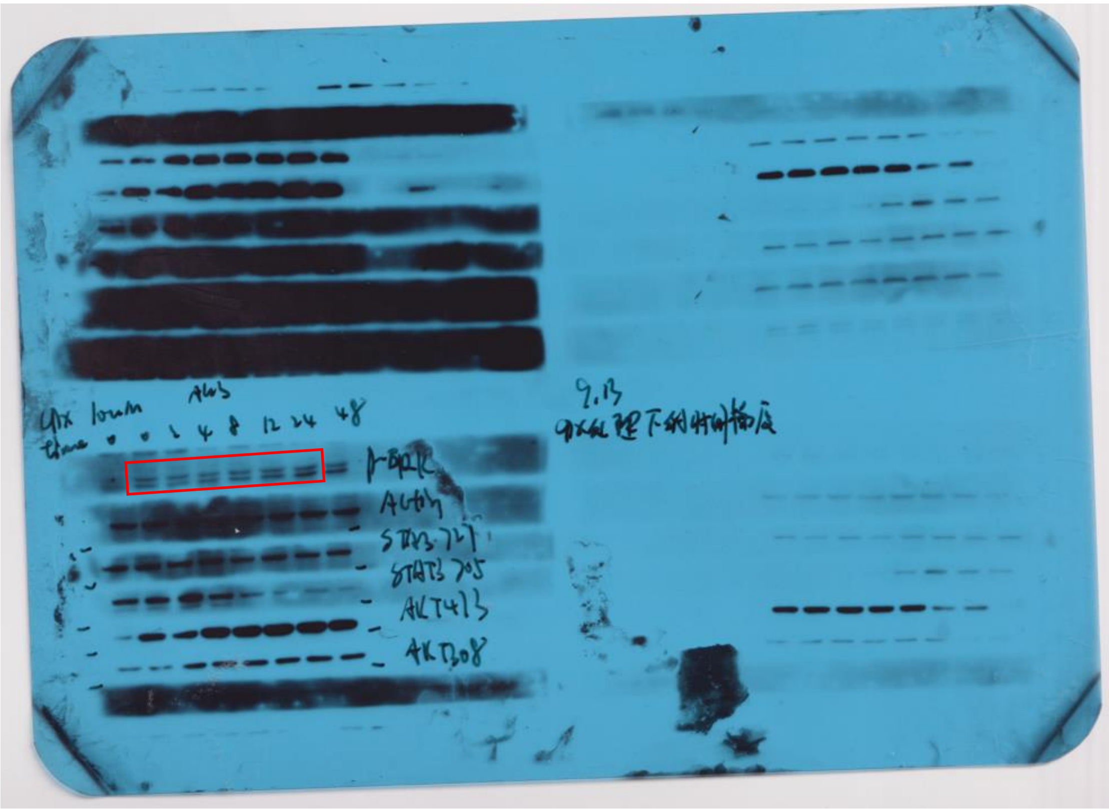
9.13
p-ERK
Actin
STAT3 727
STAT3 705
AKT473
AKT308
0 1 4 8 12 24 48

Figure S6b

MGC and SGC cells

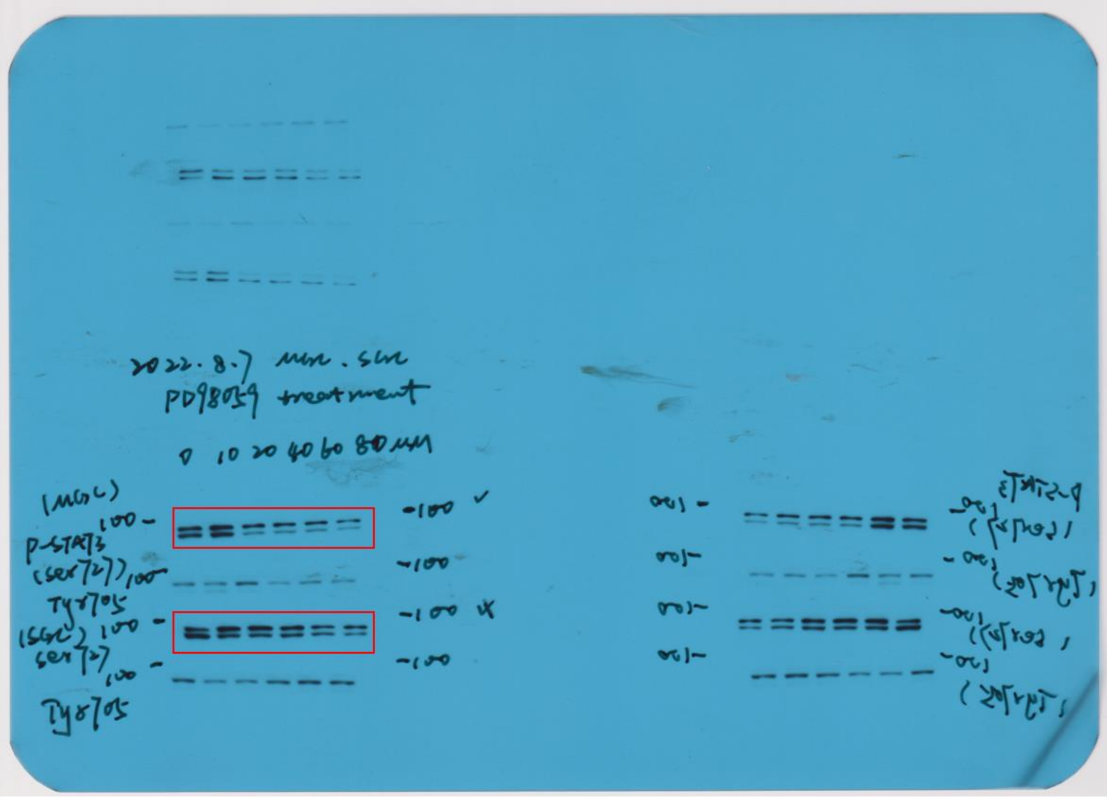

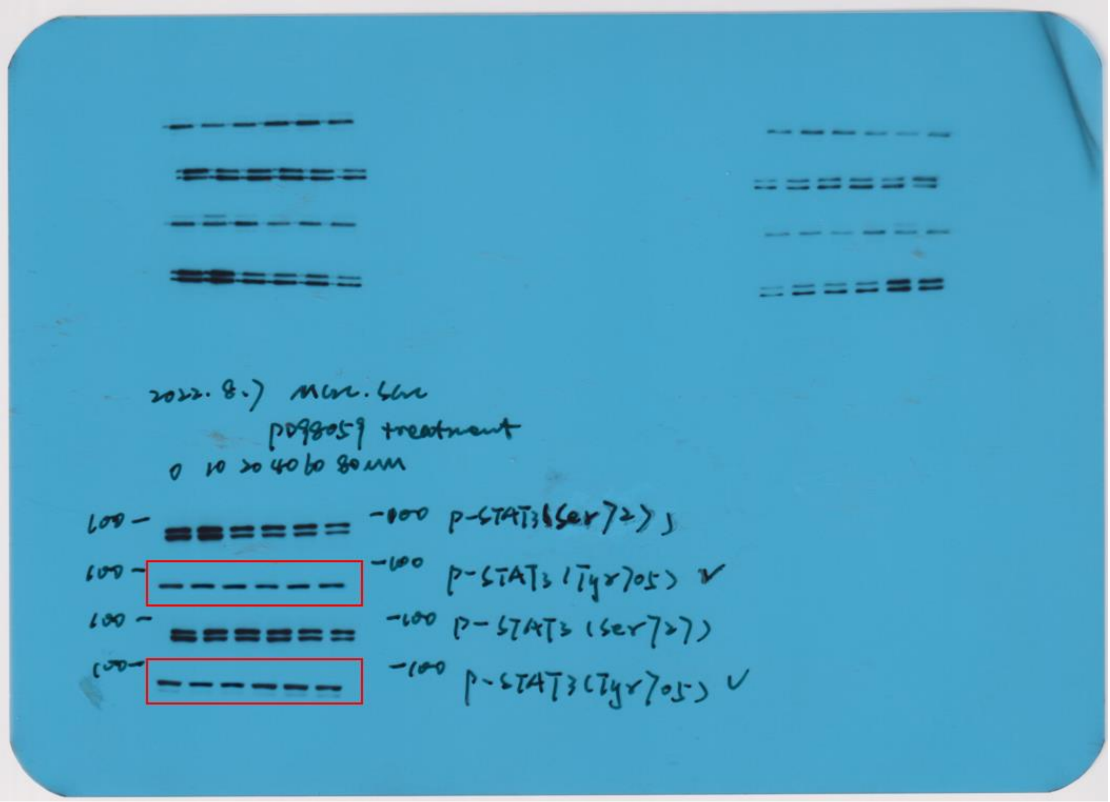

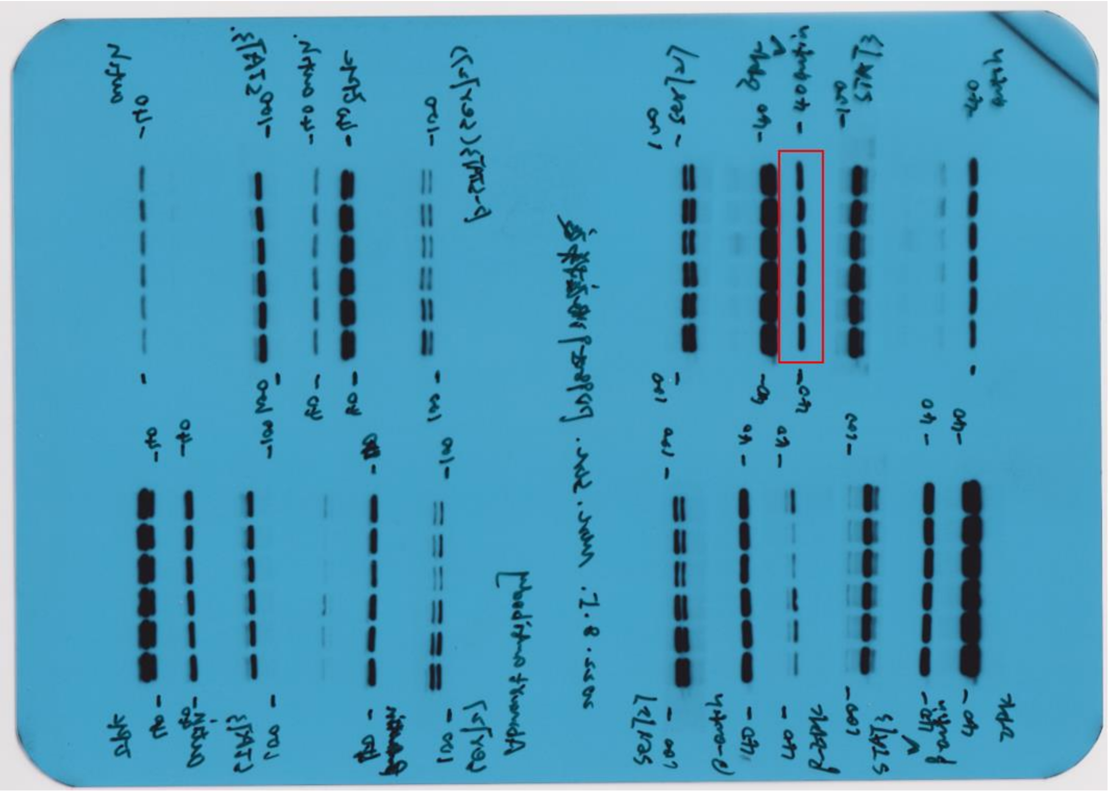

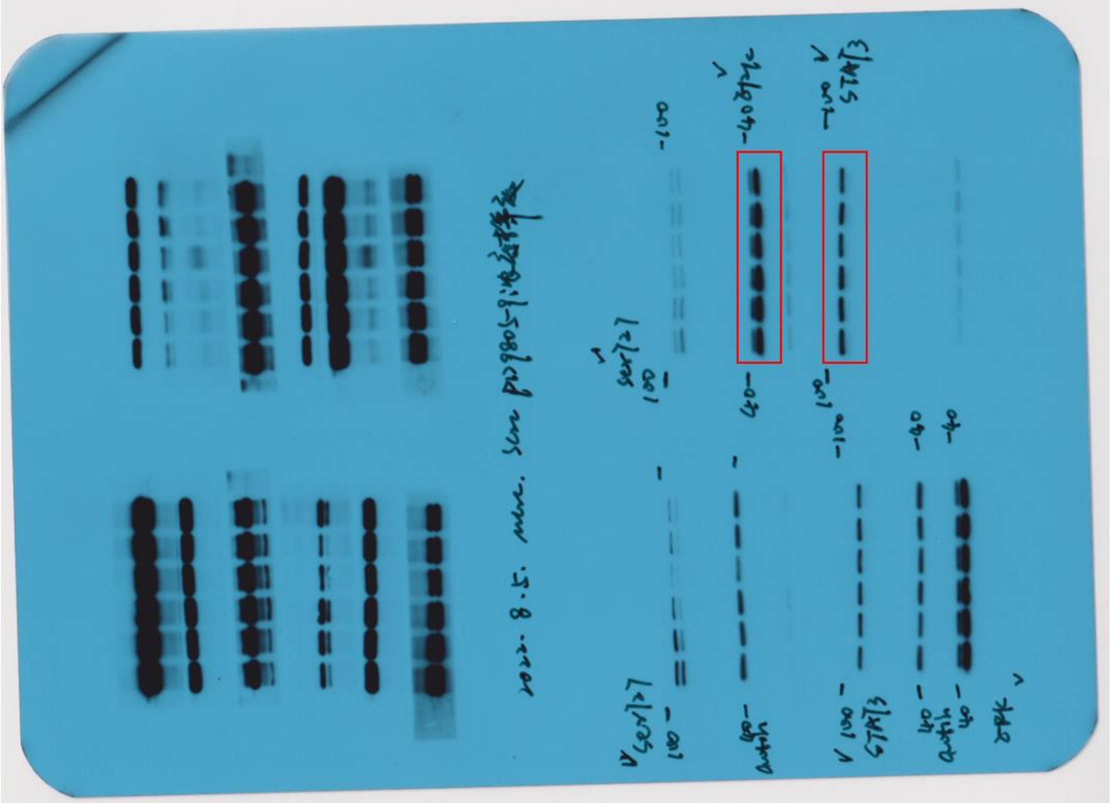

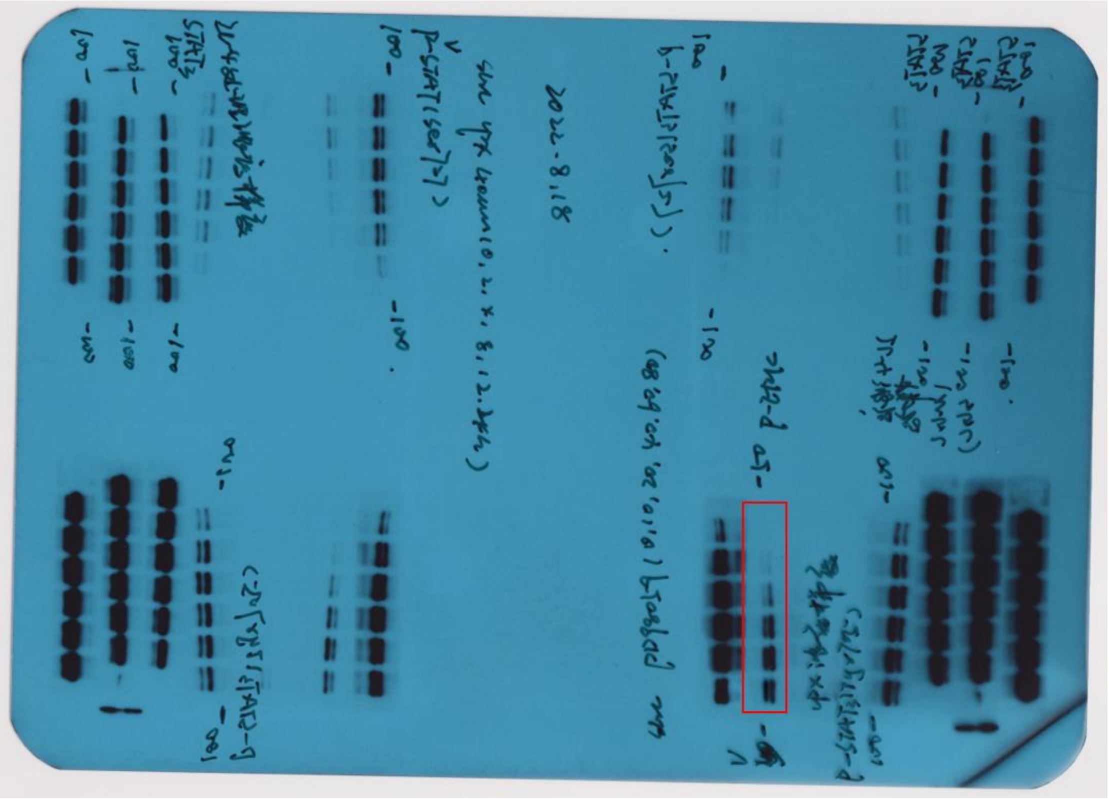
2022.8.18

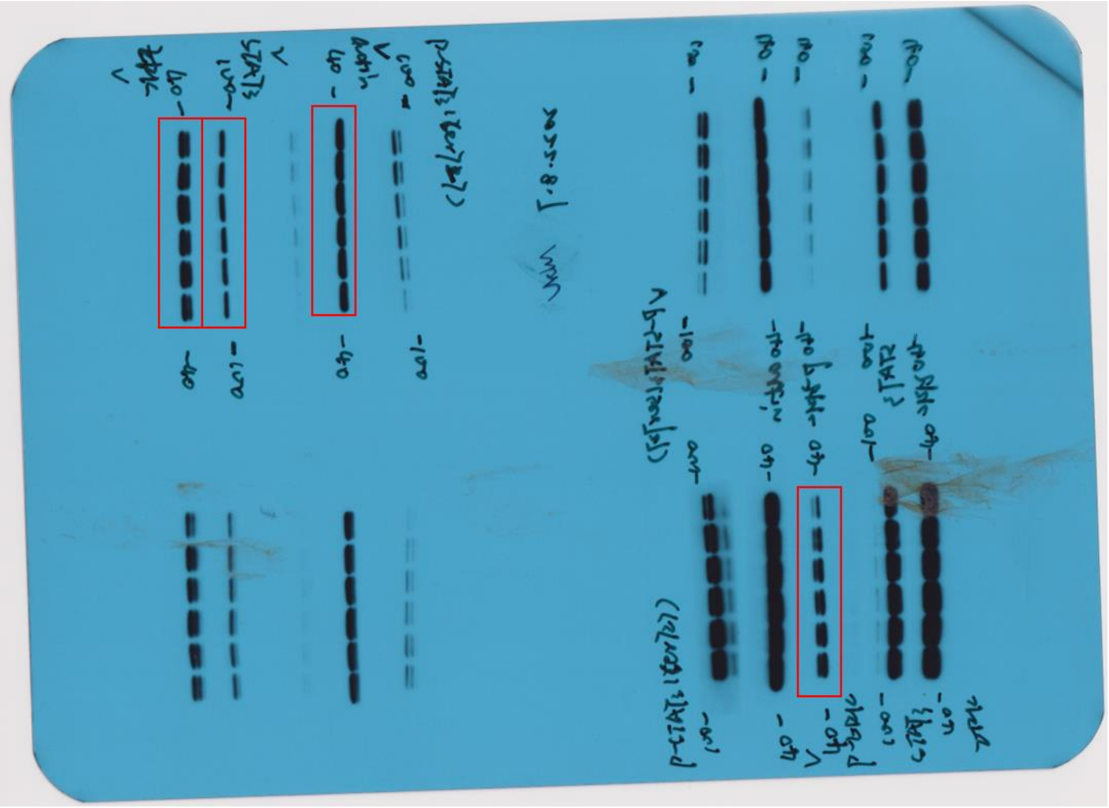

AGS cells

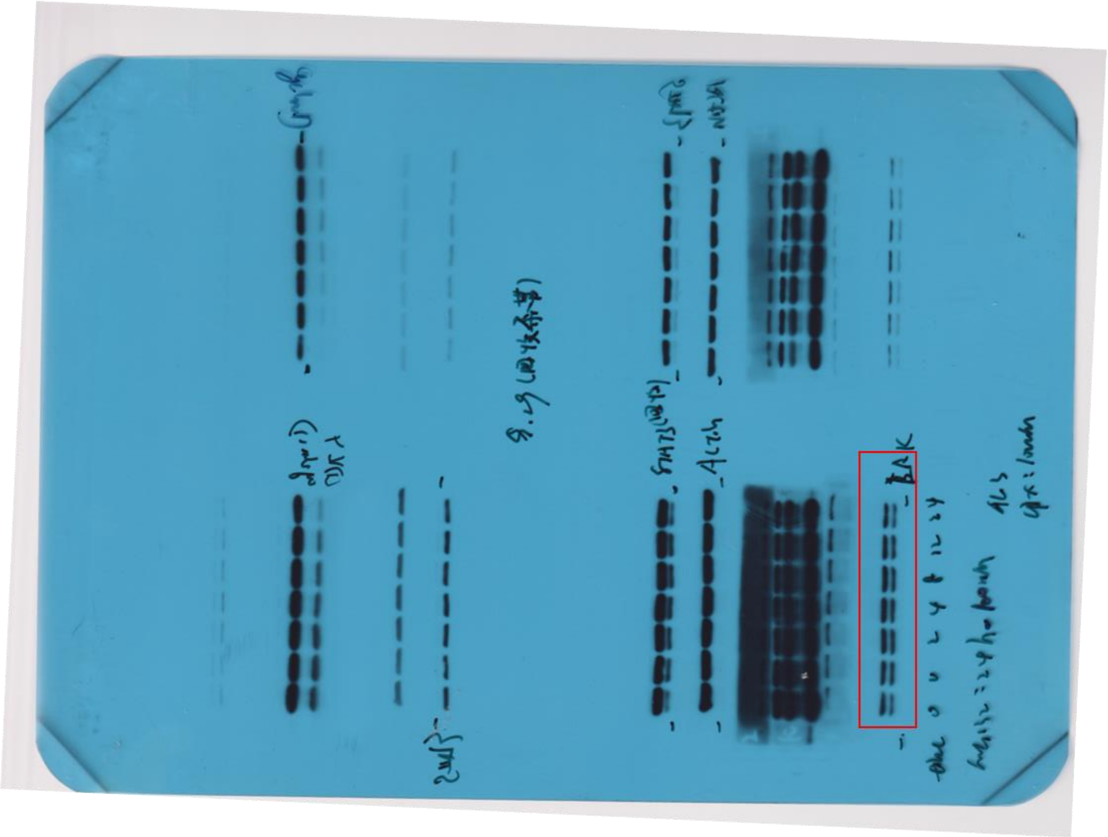

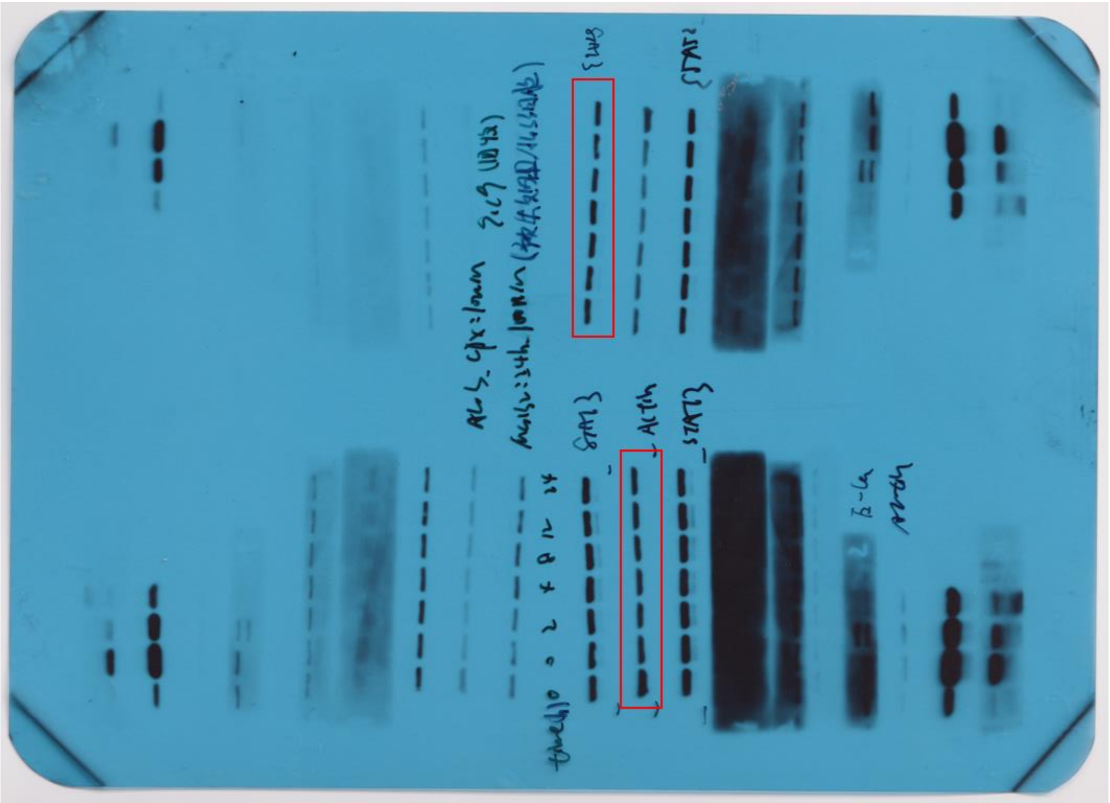

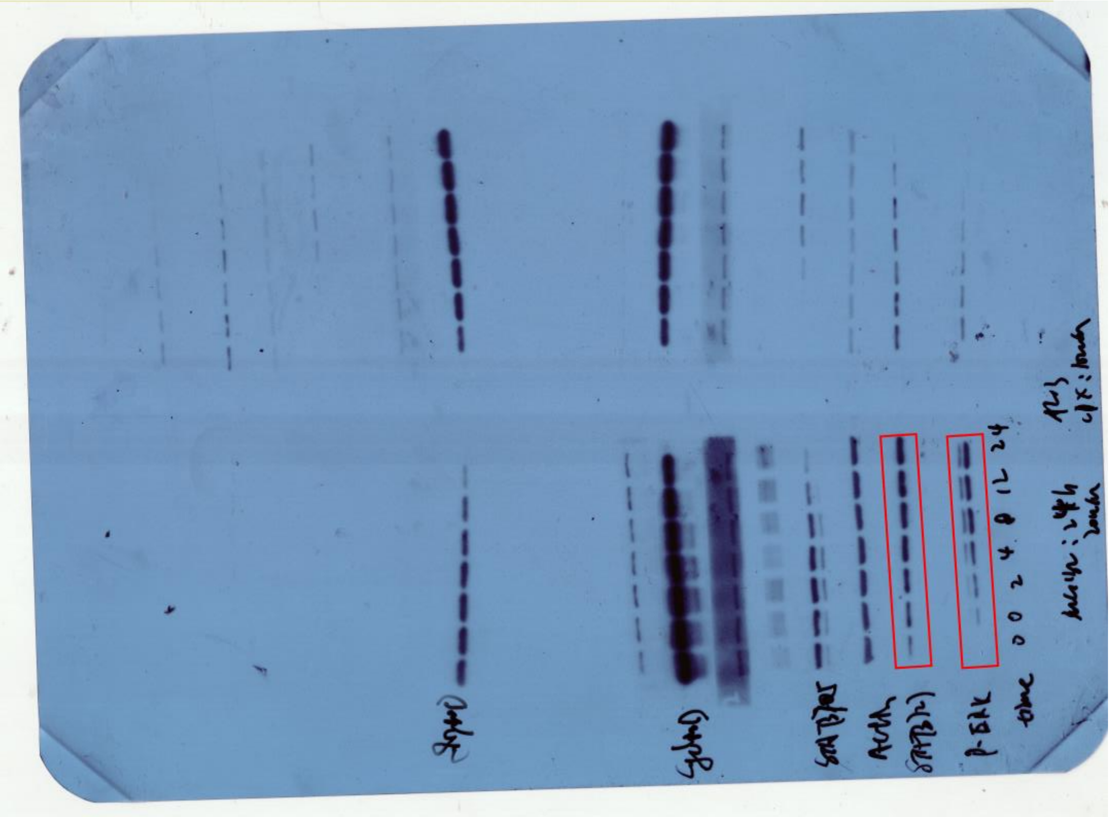

Figure S6c

MGC and SGC cells

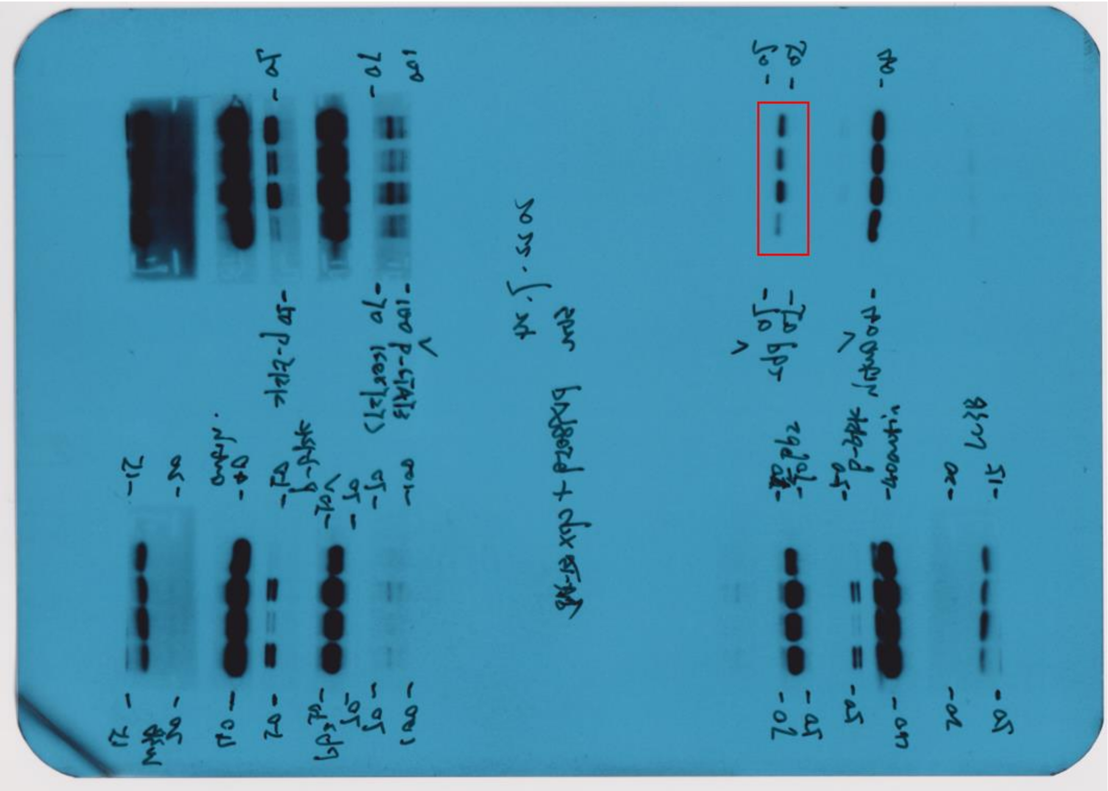

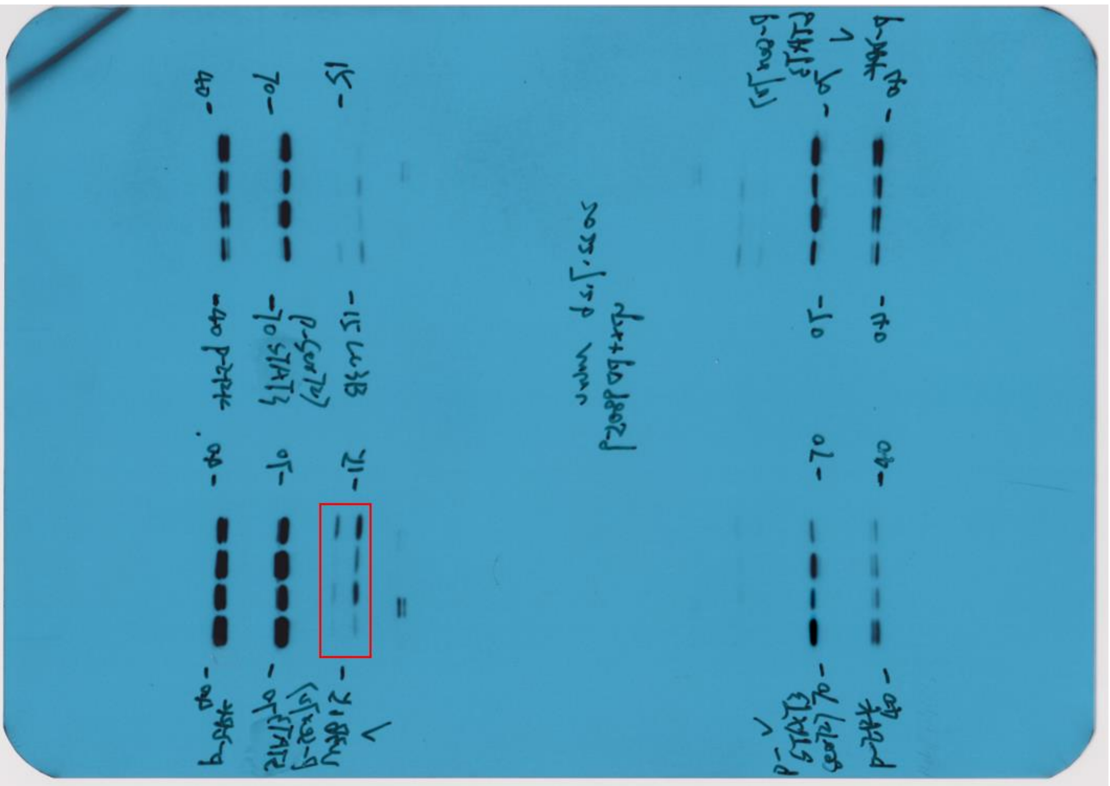

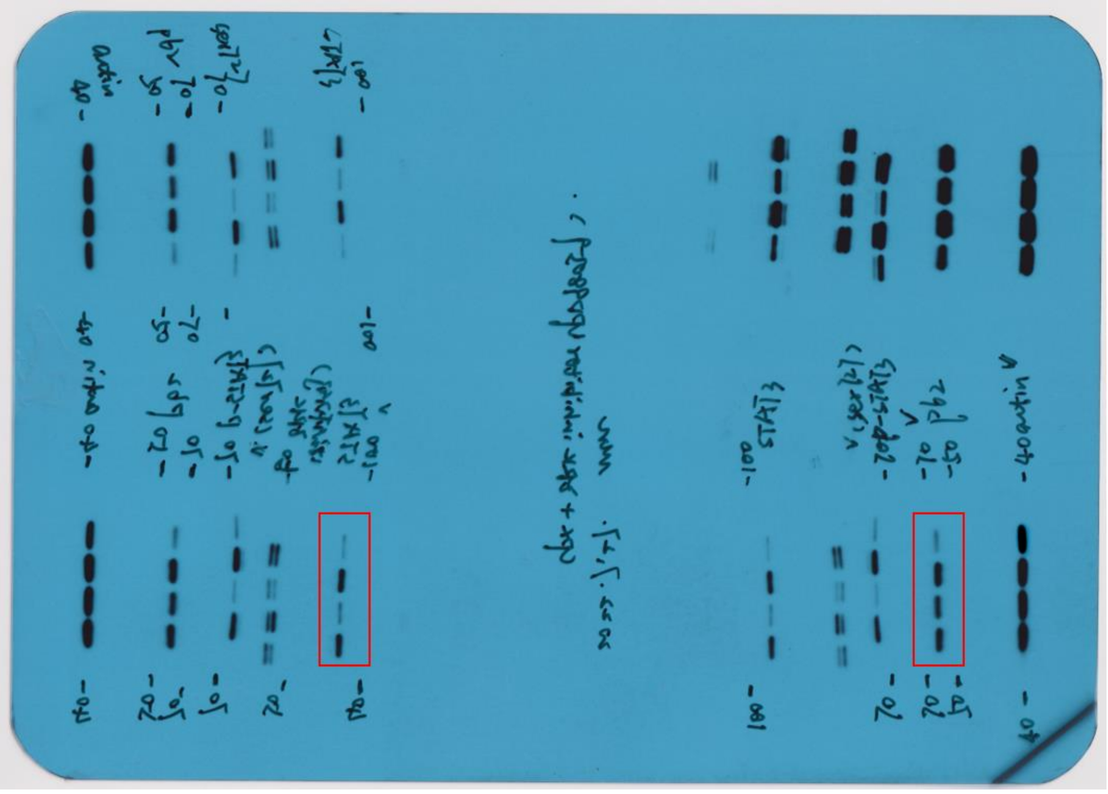

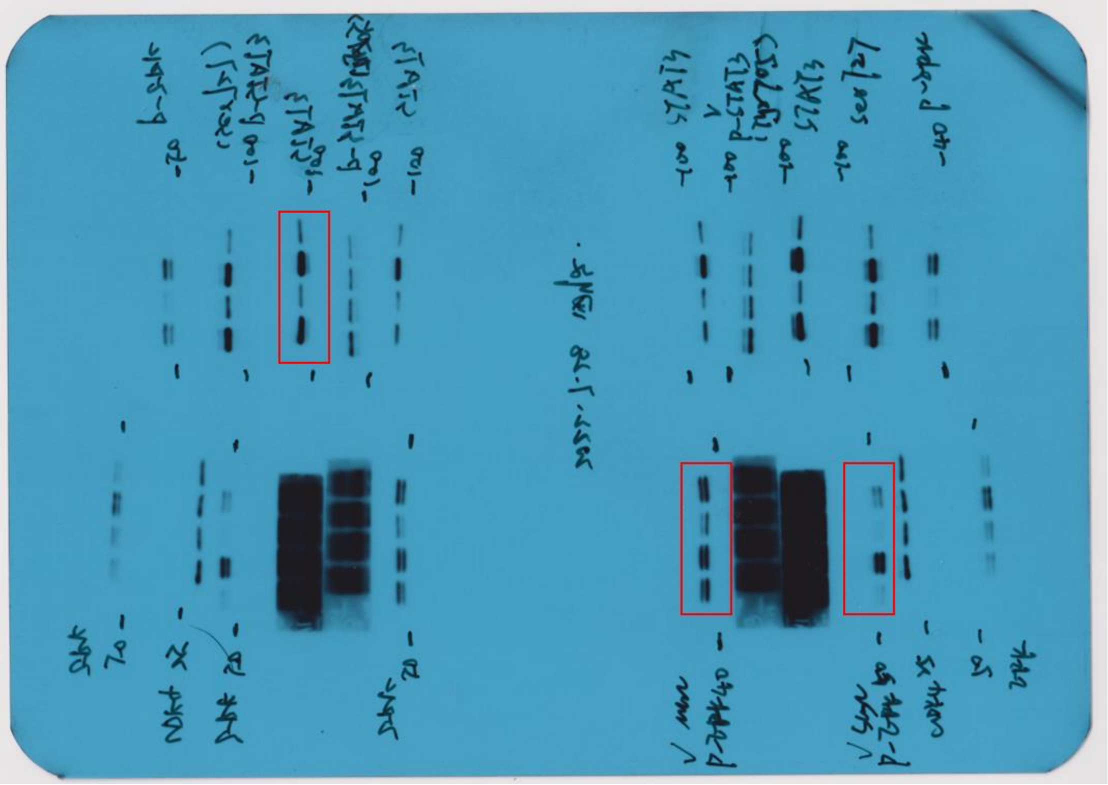

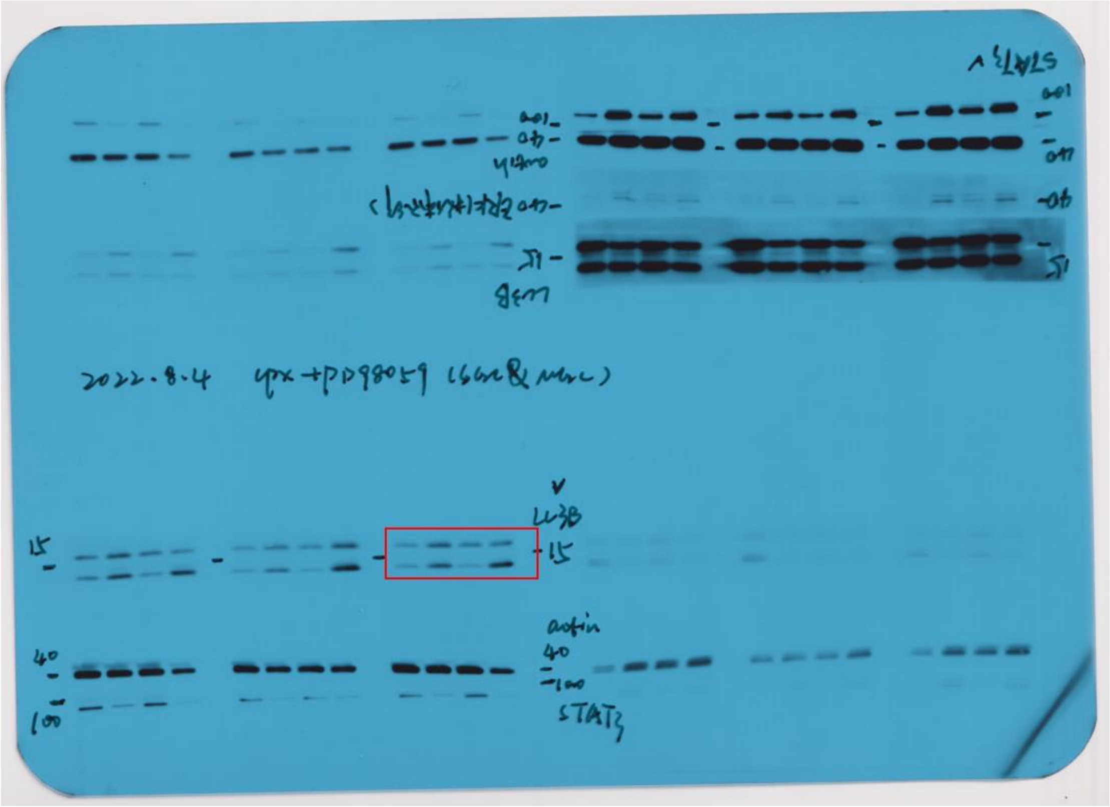
LC3B
actin
STAT3

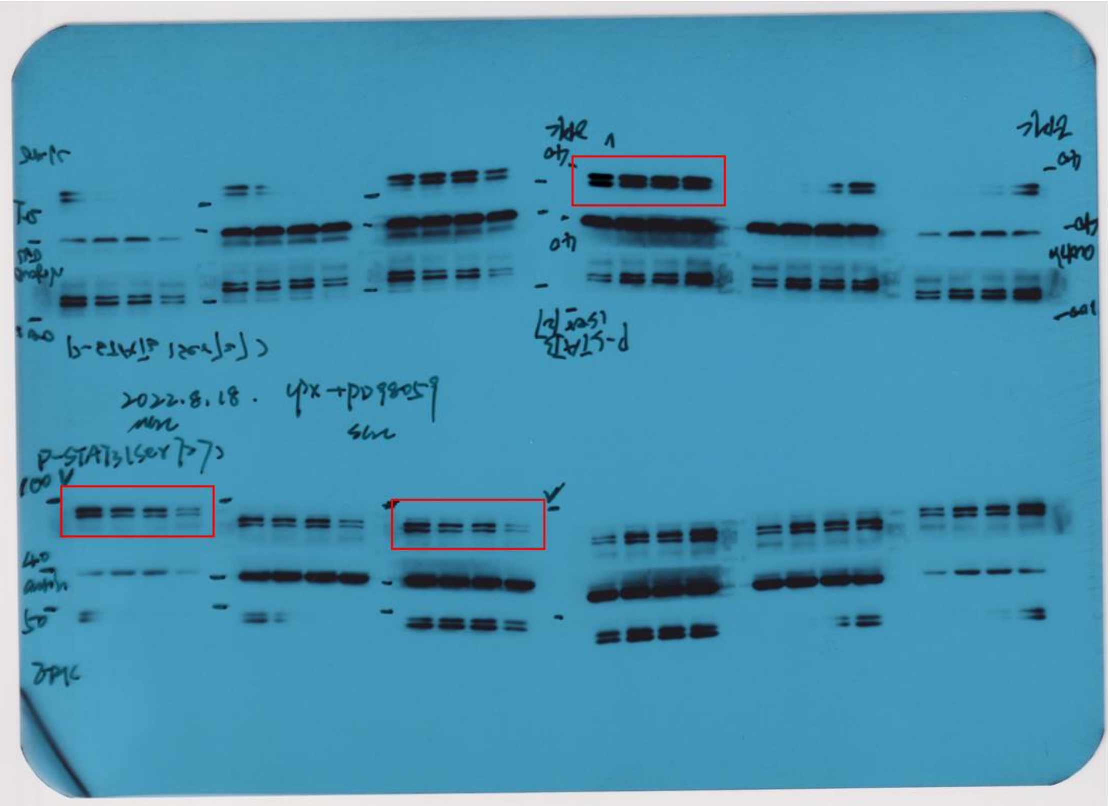
2022.8.18.
P-STAT3(ser727)

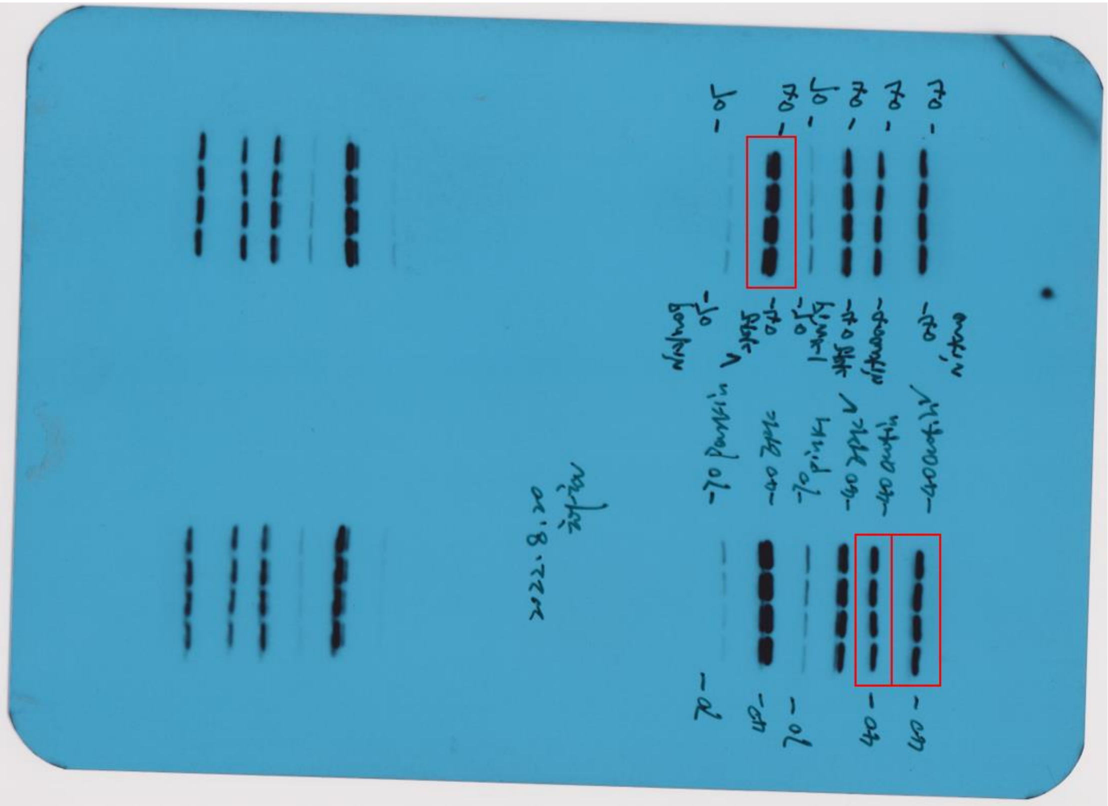

AGS cells

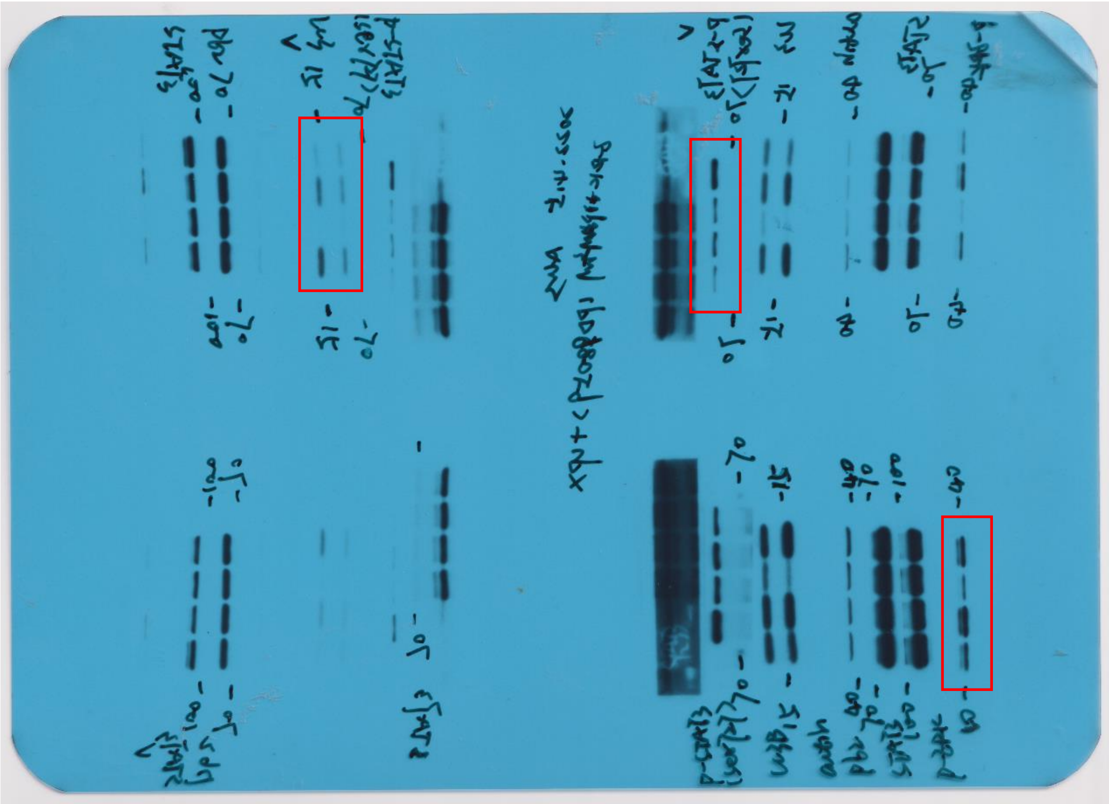

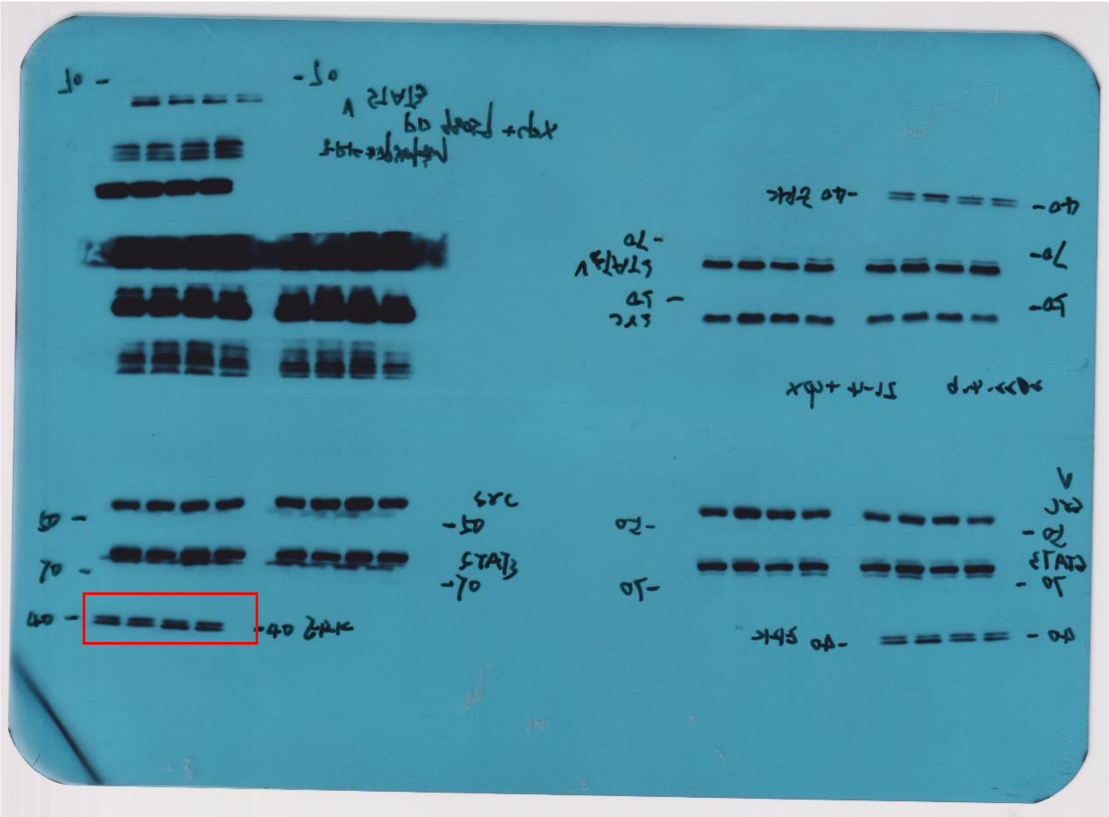

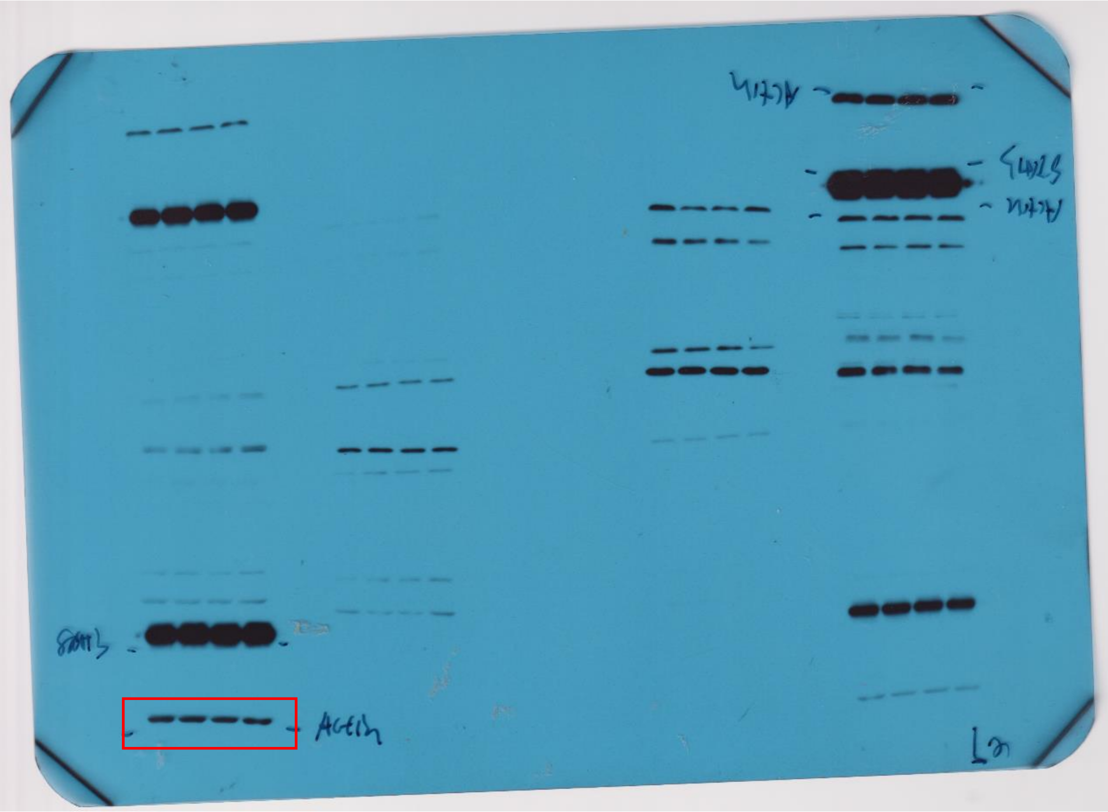
STAT3
Actin

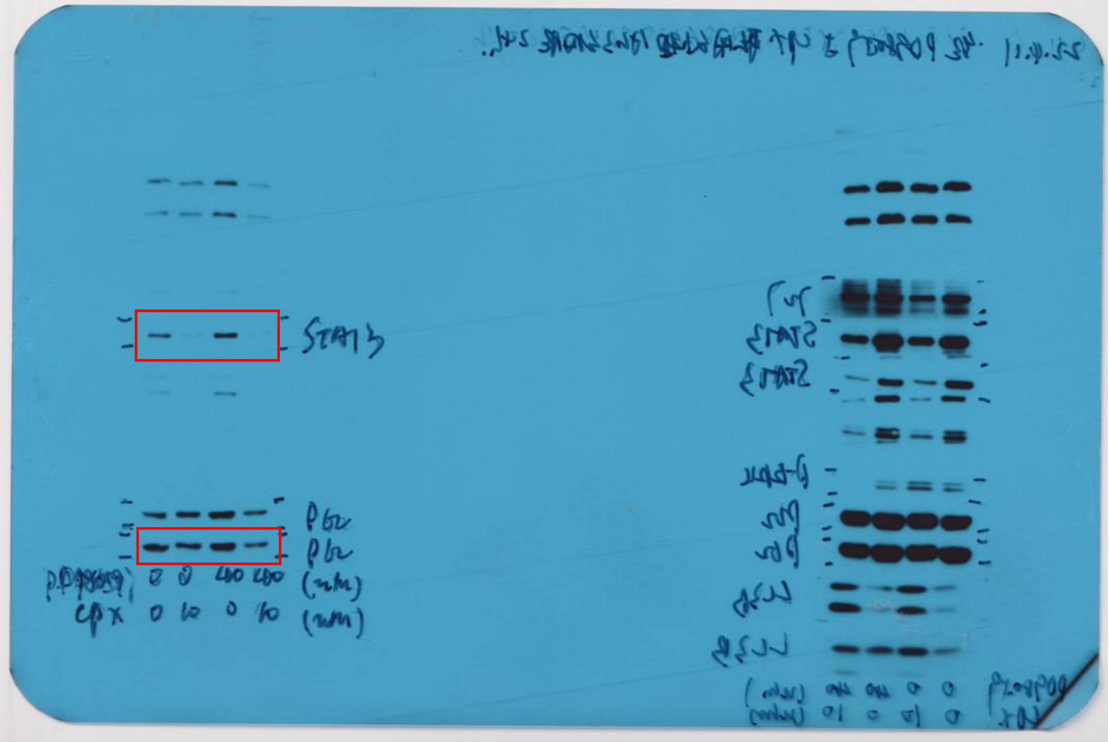
STAT3
p62
p62
(nM)
(uM)
