# Supplementary material for: Ciclopirox drives growth arrest and autophagic cell death through STAT3 in gastric cancer cells
Source: Cell Death Dis. 2022 Nov 28;13(11):1007. doi: 10.1038/s41419-022-05456-7 (PMC9705325; doi:10.1038/s41419-022-05456-7)
Supplement: Supplementary file 4 — Author-contribution-form [file 41419_2022_5456_MOESM4_ESM.pdf]

DECLARATION OF CONTRIBUTIONS TO ARTICLE

ADMC

Manuscript Number: CDDIS-22-2047R

Journal Name: *Cell Death & Disease* (the 'Journal')

Proposed Title of the Contribution:

Ciclopirox drives growth arrest and autophagic cell death through STAT3 in gastric cancer cells (the 'Contribution)

Author(s):

Lingyan Chen, Dejian Chen, Jiwei Li, Lipeng He, Ting Chen, Dandan Song, Shuang Shan, Jiaxin Wang, Xiaoang Lu, and Bin Lu (the 'Authors')

For all *CDDis* articles, each person named as an author in the published version must be able to show he or she has contributed substantially to the article.

Authorship credit should be based on 1) substantial contributions to conception and design, acquisition of data, or analysis and interpretation of data; 2) drafting the article or revising it critically for important intellectual content; and 3) final approval of the version to be published. Authors should meet conditions 1, 2 and 3.

Any person who cannot be shown to have made a substantial contribution to the article cannot be listed as an author in the final version. The name of any person who is deemed to have made a minor contribution can, however, appear in the Acknowledgments section of the article.

Please complete the table below to indicate the contributions of all named authors to the manuscript.

Author Full Name:	Specification of Contribution to the Manuscript:
Lingyan Chen	Conducted experiments, performed data analysis and wrote the manuscript
Dejian Chen	Conducted experiments, performed data analysis, assisted in imaging
Jiwei Li	Assisted in Western blot, performed data analysis
Lipeng He	Assisted in apoptosis assay and in vivo subcutaneous xenograft models
Ting Chen	Assisted in cell cycle assay and Western blot
Dandan Song	Assisted in immunofluorescence staining and imaging
Shuang Shan	Assisted in RT-qPCR and immunohistochemistry assay
Jiaxin Wang	Assisted in in vivo subcutaneous xenograft models
Xiaoang Lu	Assisted in constructing GC cell lines
Bin Lu	Conceived the project, participated in research design, performed data analysis, and wrote the manuscript

Please complete the table below to indicate the contributions of all named authors to the figures.

Figure 1:

LC generated the data and prepared panels A-D. TC generated the cell cycle data and prepared panel E. TC and DC generated Western blot data and prepared panel F. LC and BL assembled the figure.

Figure 2:

LC and DC generated the data and prepared panels A-F. LH generated with the cell death data and prepared panel G. LC and BL assembled the figure.

Figure 3:

LC and DC generated the Western blot data and prepared panel A. SS generated the data and prepared the panel B. LC and DC generated the data and prepared panel C-H. LC and BL assembled the figure.

Figure 4:

DC and JL generated the data and prepared the panel A. DS generated the IF data and prepared the panel B. LC and XL generated data and prepared the panel C-D. LC and BL assembled the figure.

Figure 5:

LC and DC generated the IP data and prepared panel A-B. LC, DC, and XL generated data and prepared the panel C-D. LC and BL assembled the figure.

Figure 6:

LC, DC, LH, and JW generated the xenograft tumor data and prepared panel A-D. DC and SS generated the IHC data and prepared panel E. LC and BL assembled the figure.

Signed for and on behalf of the Author(s):	Print Name:	Date:
[signature]	BIN LU	October 26, 2022
